# Non-parametric Regression Among Factor Scores: Motivation and Diagnostics for Nonlinear Structural Equation Models

**DOI:** 10.1007/s11336-024-09959-4

**Published:** 2024-04-23

**Authors:** Steffen Grønneberg, Julien Patrick Irmer

**Affiliations:** 1 Department of Economics, BI Norwegian Business School, Oslo, 0484 Norway; 2 Department of Psychology, Goethe University Frankfurt, Frankfurt am Main, Germany

**Keywords:** structural equation models, non-linear structural equation models, non-parametric estimation, identification, factor scores

## Abstract

We provide a framework for motivating and diagnosing the functional form in the structural part of nonlinear or linear structural equation models when the measurement model is a correctly specified linear confirmatory factor model. A mathematical population-based analysis provides asymptotic identification results for conditional expectations of a coordinate of an endogenous latent variable given exogenous and possibly other endogenous latent variables, and theoretically well-founded estimates of this conditional expectation are suggested. Simulation studies show that these estimators behave well compared to presently available alternatives. Practically, we recommend the estimator using Bartlett factor scores as input to classical non-parametric regression methods.

**Supplementary Information:**

The online version contains supplementary material available at 10.1007/s11336-024-09959-4.

## Introduction

Structural equation models (SEMs) describe how an endogenous latent random vector $\eta$ is influenced by an exogenous random vector $\xi$ as well as coordinates of $\eta$, where $(\xi ', \eta ')$ belong to a randomly chosen person in a population. Usually, both vectors are latent and continuous. The added complexity of this latency may explain the current sparsity of tools for motivating and diagnosing the functional form of this influence. This paper provides a population-based theoretical foundation for non-parametrically estimating the functional forms of the relationships between the coordinates of $(\xi ', \eta ')'$ that is based on Bartlett (1937) factor scores computed from the observables measuring $\eta$ and $\xi$. The population-based perspective of the paper means that we ignore sampling error for mathematical convenience, which correspond roughly to assuming that the sample size is large.

Even from a population perspective, the factor scores, say, $\ddot{\xi }$ and $\ddot{\eta }$, approximate the latent variables $\xi$ and $\eta$, respectively, with high precision only when the number of observable variables that measure them is sufficiently high (Krijnen, 2004, 2006a, b). For a low number of measurement variables, each individual factor score may still be a low precision approximation to the corresponding true latent variable. This is sometimes called factor indeterminacy (see, e.g.  Grice, 2001). Still, this paper shows that trend estimates for the effect of $\xi$ onto $\eta$ based on factor scores can work well in realistic conditions, and that what matters most for the quality of the trend estimate is the number of measurement variables $d_x$ of $\xi$. Loosely speaking, the reason for this is as follows: The trend estimate is based on averaging observations of $\ddot{\eta }$ for a given local range of observations of $\ddot{\xi }$. This approximates the true trend defined as averages of observations of $\eta$ for a given local range of observations of $\xi$. The averaging of $\ddot{\eta }$ cancels completely out the mean zero approximation error $\ddot{\eta }- \eta$, but the same effect is not present for the local range of observations of $\ddot{\xi }$ as an approximation to the local range of observations of $\xi$, which improves only as $d_x$ increases.

With the caveat that individual factor scores may be rough approximations to the latent variables, scatter plots of factor scores with trend estimates can still motivate and diagnose functional forms in SEMs in much the same way as scatter plots and superimposed trends are commonly used in applied regression analysis (see, e.g., Fox & Weisberg, 2011; Weisberg, 2005). While some specification tests or tests for quadratic and interaction terms for SEM exist (Nestler, 2015; Büchner and Klein, 2020), trend estimates of the functional form in SEM are useful also for linear SEM, as traditional covariance-based tools such as the Chi-square goodness-of-fit test and its robustified variants may have zero power toward non-linear alternatives (Mooijaart and Satorra, 2009).

In this paper, $\xi$ and $\eta$ are assumed to be latent and measured via a correctly specified linear factor model, as specified shortly. This means that we consider diagnostics or motivation of the measurement model as outside the scope of the present paper.

We will later assume that the error terms of the factor model and the factors are independent and that the factors are continuous variables. This can only happen if the observed variables are continuous (see Appendix G in the online supplementary material). While treating ordinal data as continuous is sometimes justified under additional assumptions (Foldnes and Grønneberg, 2022; Grønneberg and Foldnes, 2024), this paper only deals with continuous observations. Ordinal data models, such as item response theory or threshold models, are outside the scope of the present paper.

The trend estimates we consider are non-parametric regressions for the structural connections between the coordinates of $(\xi ', \eta ')'$. If $\mathbb {E} \eta$ exists, then the conditional expectation $\mathbb {E} [\eta |\xi ]$ exists (see Appendix K in the online supplementary material for a review of conditional expectations), which implies that

1 $$\begin{aligned} \eta = H(\xi ) + \zeta , \quad \zeta := \eta - \mathbb {E} [\eta |\xi ], \quad H(x)= \mathbb {E} [\eta |\xi =x], \quad \mathbb {E} [\zeta | \xi ] = 0. \end{aligned}$$

Recall that $\mathbb {E} [\zeta | \xi ] = 0$ implies ${\text {Cov}} \, (\varphi (\xi ), \zeta ) = 0$ for all integrable functions $\varphi$ (see Appendix K). This is stronger than merely assuming ${\text {Cov}} \, (\xi , \zeta ) = 0$, but weaker than independence between $\zeta , \xi$ as this is equivalent to ${\text {Cov}} \, (\varphi (\xi ), \varrho (\zeta )) = 0$ for any integrable functions $\varphi ,\varrho$.

In Eq. (1), we considered the total effect of $\xi$ onto $\eta$ (for an overview of linear mediation analysis see MacKinnon et al., 2007). By the same reasoning, we can consider each coordinate $\eta _j$ of $\eta$ separately, conditioning $\eta _j$ not just on $\xi$, but instead on both $\xi$ and the connections from $\eta$ substantive knowledge dictates influences $\eta _j$. If the substantive knowledge is correct, a proposition usually not fully identified from data alone (Bollen, 1989; Jöreskog et al., 2016), this non-parametrically estimates the trend of a full SEM. This approach, which we call the component-wise approach, is more fully described and exemplified in Appendix B in the online supplementary material.

Algorithmically, the only difference between the component-wise approach and the reduced form approach considered in Eq. (1) is the names of the variables involved. To reduce the notational burden of the paper, we will therefore focus the main text on estimating *H* in the reduced form representation of Eq. (1). While the component-wise approach is of higher practical interest in most cases, its mathematics is exactly the same as the reduced form approach if we re-label the variables.

To illustrate the difference between the component-wise and reduced form approaches, consider the simple system

$$\begin{aligned} \eta _1 = \xi _1^2 + \mathfrak {z}_1, \quad \eta _2 = \eta _1 + \xi _1 + \mathfrak {z}_2 = \xi _1 + \xi _1^2 + \mathfrak {z}_1 + \mathfrak {z}_2. \end{aligned}$$

For this illustration, assume that the error terms $\mathfrak {z}_1, \mathfrak {z}_2$ and the exogenous variable $\xi _1$ are zero mean and independent. We first consider $\eta _1$. In both the component-wise and the reduced form approaches, we consider $\mathbb {E} [\eta _1 | \xi _1] = \xi _1^2$, showing that $\mathfrak {z}_1$ is also the error term induced by the conditional expectation representation, i.e., $\mathfrak {z}_1 = \zeta _1:= \eta _1 - \mathbb {E} [\eta _1 | \xi _1]$. We then consider $\eta _2$. In the component-wise approach, we calculate $\mathbb {E} [\eta _2 | \eta _1, \xi _1] = \eta _1 + \xi _1$, which is linear. The error term $\mathfrak {z}_2$ is then the error term induced by this conditional expectation calculation, i.e., $\mathfrak {z}_2 = \eta _2 - \mathbb {E} [\eta _2 | \eta _1, \xi _1]$. From the expanded system shown at the end of the above display, we also deduce the reduced form trend $\mathbb {E} [\eta _2|\xi _1] = \xi _1 + \xi _1^2$, which is quadratic, with an induced error term $\zeta _2:= \mathfrak {z}_1 + \mathfrak {z}_2 = \eta _1 - \mathbb {E} [\eta _2|\xi _1]$. We see that in both cases, we detect a non-linear trend in the system. With structural knowledge, we are able to further detect that the non-linear trend affects only $\eta _1$ directly. More comprehensive examples and analytical examples are provided in Appendix B in the online supplementary material.

Our suggested empirical approach is based on plotting factor scores together with a non-parametric estimate of *H* to motivate or diagnose the functional form of a SEM. The non-parametric estimate will be rough and is in most cases best suited as a guide to model formulation and diagnostics—not as a standalone estimation technique. See Appendix A in the online supplementary material for a simple numerical illustration. Once an appropriate parametric model is identified, it is then estimated via standard techniques such as the classical linear approach, the latent moderated structural equations approach (LMS, Klein & Moosbrugger, 2000), or the unconstrained product indicator approach (UPI, Marsh et al., 2004; Kelava & Brandt, 2009). A literature review of available estimation methods is found in Appendix C in the online supplementary material. This approach follows common practice in the applied regression literature (see, e.g., Fox & Weisberg, 2011; Weisberg, 2005), where non-parametric estimates are used to guide parametric modeling.

Plotting factor scores for model motivation and diagnostics has roots going back to McDonald (1967) who worked with nonlinear factor models. In the context of SEM, Bauer et al. (2012) appear to be the first to suggest adding trend estimates to this plot, and Bauer et al. (2012) also showed through simulation that this gives reasonable results. Our paper provides the theoretical underpinnings of the method, as well as substantial simulation work to further assess the performance of the method.

Another approach to model diagnostics in SEM is residual analysis. Bollen and Arminger (1991) define residuals for linear SEM via factor score-based estimators of the error terms of the measurement model and the structural model. Raykov and Penev (2014) show via simulation that plotting coordinates of residuals from a structural model against each other can be used to detect unaccounted for structural trends. While a formal analysis of these procedures would be intimately connected to the contributions in the present paper, residual analysis is a complex topic, and we consider it outside the scope of the present paper.

For mathematical convenience, our analysis is limited to population quantities, and we deal only with the consistency of estimates of *H*. Inference for *H* is not considered in the paper, though standard bootstrap approaches may be applicable. Our paper also provides insights into what types of non-parametric regression methods should be used through a theoretical analysis and a comprehensive simulation study.

Our focus is on non-parametric estimators of *H* that make no parametric assumptions on *H* and no parametric assumptions on the distributions of $\eta$ and $\xi$. As reviewed in Appendix C in the online supplementary material, there are many ways to estimate *H*, but to the best of our knowledge, the only presently available non-parametric estimators for *H* are in the presently understudied papers of Kelava et al. (2017) and Kohler et al. (2015). Since no implementation of the estimator of Kohler et al. (2015) is available, we do not consider it in our paper.

We do, however, compare our suggested methods with the computationally demanding method of Kelava et al. (2017). Out of the methods we compare, our simulations indicate that inputting Bartlett scores into simple LOESS or spline methods work best, on average. This is computationally practically instantaneous.

As mentioned above, we assume $\xi$ and $\eta$ are measured through correctly specified linear factor models: Let $f=(\xi ', \eta ')'$ where ^′^ is vector transposition. We let the dimension of a random vector, say *V*, be denoted as $d_V$. We observe a sample of size *n* from the random vector $z = (x', y')'$ which follows the factor model

2 $$\begin{aligned} \tilde{z}:= z - \mu = (\tilde{x}',\tilde{y}')' = (x'-\mu _x',y'-\mu _y')' = \Lambda f + \varepsilon , \quad f = (\xi ', \eta ')', \quad \varepsilon = (\varepsilon _x', \varepsilon _y')', \end{aligned}$$

where $\mu = (\mu _x', \mu _y')'$ is the expectation of *z*, where $\Lambda$ is a non-random $(d_\xi +d_\eta )\times (d_x+d_y)$ matrix, and where $\varepsilon$ consists of measurement errors. Precise assumptions on the factor model will be given later.

Identifying a correct measurement model is a difficult, though standard problem. The assumption of a correctly specified and linear measurement model is made by all standard non-linear as well as linear structural equation models (see, e.g., Jöreskog, 1969; Kenny & Judd, 1984; Wall & Amemiya, 2000; Klein & Moosbrugger, 2000; Skrondal & Laake, 2001; Wall & Amemiya, 2001, 2003; Marsh et al., 2004; Lee et al., 2007; Kelava & Brandt, 2009; Mooijaart & Bentler, 2010; Mooijaart & Satorra, 2012; Croon, 2002; Kohler et al., 2015; Kelava et al., 2017; Devlieger & Rosseel, 2017; Brandt et al., 2018; Holst & Budtz-Jørgensen, 2020; Rosseel & Loh, 2022). In Appendix F in the online supplementary material, we show that the techniques presented in the present paper are also compatible with certain non-linear measurement models that can be rewritten as linear measurement models. Then, we can derive analytically how measurement model misspecification influences estimates of *H*, and that numerical experiments show that the proposed methodology is not overly sensitive to minor measurement model misspecification.

In this paper, we only consider additive measurement error, both in the structural and measurement part of the model. Our approach centers around approximating the conditional expectation function *H*, which enters in an additive relationship to $\zeta$. For distributions of $(\xi ', \eta ')'$ with errors entering non-additively, this need not be the right perspective for studying trends. See Appendix J in the online supplementary material for a simple example.

### Inputting Factor Scores to Non-parametric Regression Methods, a Literature Review and an Overview of our Theoretical Contributions

Traditional parametric regression methods among factor scores have been studied in several papers, among them Skrondal and Laake (2001); Devlieger et al. (2016); Devlieger and Rosseel (2017); Croon (2002); Hoshino and Bentler (2011) as well as the more recent SAM (structural after measurement) approach of Rosseel and Loh (2022). Also PLS-SEM and some of its variants (Sarstedt et al., 2021; Dijkstra and Henseler, 2015) are based on regression methods among factor scores (Yuan and Deng, 2021). In contrast, inputting factor scores into non-parametric regression methods is a far less well-studied problem. The first paper we have found on this is Bauer et al. (2012). Bauer et al. (2012) have two proposals for diagnostics and model formulation in NLSEM: The first proposal is to input factor scores to non-parametric regression estimators, which is the research area this paper continues. The second proposal is to consider structural equation mixture models, which we consider outside the scope of the present paper. While structural equation mixture models has its own literature, see, e.g., the references within Bauer et al. (2012), inputting classical scores, such as the Bartlett (1937) or Thurstone (1935, Thomson, 1934) factor scores, into non-parametric regression methods has as far as we know not been analyzed theoretically in the literature previously.

In Kelava et al. (2017) and Kohler et al. (2015), the authors propose to estimate *H* non-parametrically by a similar procedure as Bauer et al. (2012), except that instead of classical factor scores, they generate mathematically complex non-linear factor scores which are inputted into non-parametric regression procedures. Their papers include theoretical results proving that as the sample size *n* increase, these methods are consistent.

A foundational result for linear factor scores is that for its convergence in probability (and mean square) towards the true latent variables in addition to $n \rightarrow \infty$, also the number of easurement per latent variable are required to increase indefinitely (Guttman, 1955; Williams, 1978; Schneeweiss and Mathes, 1995; Krijnen, 2004, 2006a, b). This has the important implication that in general, non-parametric regression methods based on linear in contrast to nonlinear factor scores will not be consistent in estimating the true trend as $n \rightarrow \infty$, but will also require a sufficient number of measurements of the latent variables.

In the present paper, we show that under weak conditions, only $d_x$, the number of measurements of $\xi$, has to be sufficiently high to approximate *H*. We propose two kinds of theoretical approaches to the problem, both justified only for $d_x$ sufficiently high, though both are shown to work well in simulations also for small $d_x$, such as $d_x = 3$: We provide conditions so that the population versions of a class of factor scores fit into the problem of non-parametric regression with normal measurement error in the covariate. The normality of the measurement error is not based on parametric distributional assumptions on the variables in the model but is derived from a central limit theorem. We also provide conditions for when population versions of factor scores can be used to approximate *H* through a direct application of non-parametric regression estimates, such as the LOESS estimate (Cleveland, 1979, 1981) or smoothed splines (Chambers and Hastie, 1992). In our simulations, this second alternative, which is the computationally and mathematically simplest method of all considered, usually has best performance, also when taking into account the computationally complex method of Kelava et al. (2017).

In this paper, we do not consider the method proposed in Kohler et al. (2015), as no implementation of this method appears to be available. Kelava et al. (2017) provides a $\texttt {MATLAB}$ (The MathWorks Inc., 2023) implementation of their algorithm, which we use in our simulations. Their non-linear factor scores minimize a loss function defined in terms of unspecified constants called probability weights. The performance of their method depends on the choice of these probability weights as well as which non-parametric regression method is used in the second stage, where the provided implementation used B-splines (De Boor, 1978). We take the choice of probability weights as given in the implementation of Kelava et al. (2017). In the choice of a second stage non-parametric method, we consider both the B-splines method analyzed in Kelava et al. (2017) and LOESS or smoothed splines as implemented in R (R Core Team, 2023). The latter appears to give better performance than the B-spline option.

The asymptotic approach we consider is to let the number of items go to infinity, where we for simplicity consider an infinite sample size. A joint asymptotic analysis where the number of observations and items increases jointly is considered outside the scope of the present paper. Such an analysis would be mathematically considerably more complex than the analysis undertaken in the present paper.

An asymptotic approach with a growing number of items is standard in the related research field of factor panel data models. There, a common asymptotic approach is to let both the panel width and length increase. In this large literature, with contributions from econometrics, statistics and related fields, factor scores or its analogues are considered, see, e.g., Fan et al. (2023) and the references therein. As far as we know, non-parametric regression among factor scores has not been considered in that literature.

### The Structure of the Paper

This paper has four main contributions. First, we establish the conditions under which the conditional expectation function of $\eta$ given $\xi$, denoted as *H*, can be identified using population factor scores. Second, we prove some basic results on affine factor scores that are suitable for such an analysis. Third, we show new asymptotic results, which include the consistency of Bartlett scores in the mean square, the normality of the measurement error of factor scores as estimates to the factors, and conditions when conditional expectations based on factor scores with decreasing measurement error converge to the conditional expectation based on factors. These first three contributions are found in Sect. 2. Fourth, we suggest non-parametric methods based on Bartlett factor scores in Sect. 3, and in Sect. 4 we evaluate them together with the Kelava et al. (2017) procedure through a simulation study. Finally, we discuss the findings from the simulation study and give concluding remarks. More technical details and a fuller discussion of some conclusions from the simulation study are deferred to an online appendix. All proofs and source code for our numerical analysis are also found in the online supplementary material.

## Identification of *H* Based on Population Factor Scores

We here investigate when *H* is identified in the population, and we base our analysis on a class of factor scores.

Identification in this context means that under the stated assumptions, we are able to pin-point *H* based on the distribution of population versions of factor scores. The measurement part of the model in Eq. (2) is a confirmatory factor model, whose parameters are identified only up to unit of measurement transformations of the factors (for an overview and historical references, see Chapter 14.2 in Anderson, 2003). We will shortly assume that the parameters of the factor model in Eq. (2) are identified, which means that the unit of measurement is chosen, either by standardizing the factors, or fixing appropriate elements of $\Lambda$ to 1, or some combination thereof. As shown in the Appendix H in the online supplementary material, conditional expectations are well-behaved with regard to changes of the units of measurement, and therefore, standard practice for setting the units of measurement can be followed. The choice of unit of measurement will have some consequences for interpretation, and formulas for converting between choices are found in Appendix H in the online supplementary material. Without substantive knowledge leading to a preferred scaling method, we recommend standardizing the factors in an empirical investigation because this is an easily interpreted object, i.e., the conditional expectation of a standardized version of $\eta$ given a standardized version of $\xi$.

Conditions for the non-parametric identification of the parameters and distributions involved in a SEM using nonlinear factor scores are given in Lemma 1 in Kelava et al. (2017). These conditions are quite strong and include that all coordinates of $\varepsilon$ are independent, that $\varepsilon$ and *f* are independent, and that no cross loadings are present, meaning no observed variable measures two latent variables simultaneously. Lemma 1 in Kelava et al. (2017) then shows that the joint distribution of $\varepsilon$ and *f* is identified from the distribution of the observable variables. From this, we can compute the marginal distribution of $\xi$, the function $H(x) = \mathbb {E} [\eta | \xi = x]$, and the distribution of the error term $\zeta = \eta - H(\xi )$. Therefore, Lemma 1 in Kelava et al. (2017) identifies *H*.

In this section, we provide an alternative set of assumptions that asymptotically identifies *H*. More precisely, we will identify *H* under assumptions that hold only as $d_x$, the number of measurements of $\xi$, increases indefinitely, which can be called asymptotic identification. Showing asymptotic identification and not exact identification allows our results to be formulated under much weaker conditions compared to those of Kelava et al. (2017), whose first lemma shows exact identification for any $d_x, d_y \ge 3$. Our analyses focus on population versions of a class of factor scores, which we now introduce.

Affine factor scores are of the form $A z + a$, where *A* is a $d_f \times d_z$ matrix, and *a* is a $d_f$ dimensional vector. Usually, $a = - A \mu$ and *A* is chosen so that $A \tilde{z}$ is in an appropriate sense as close to *f* as possible. We will only consider such factor scores, and all references to factor scores mean affine factor scores.

Let $\Phi = {\text {Cov}} \, f$ and $\Sigma = {\text {Cov}} \, z$. The ’regression’ factor scores (Thurstone, 1935; Thomson, 1934), also known as Thurstone factor scores, are derived using $A = \Phi \Lambda ' \Sigma ^ {-1}$ and $a = - A \mu$. These factor scores are optimal in the mean square sense (Neudecker and Satorra, 2003), yet we will instead focus on the Bartlett factor score, for a theoretical reason we now explain. As sketched in the upcoming Remark 1, Thurstone factor scores can likely be included in an extension of the theoretical framework considered in the present paper.

As our focus is on using factor scores as input to non-parametric regression methods, we will only consider factor scores with the property that $A \tilde{z}$ equals *f* distorted by some uncorrelated, or more strongly, independent noise, as such factor scores will fit in with the general theory on non-parametric regression with measurement error. By addition and subtraction of *f*, we may define the $d_f$ dimensional error term $r_A = A \tilde{z} - f$ so that

3 $$\begin{aligned} A \tilde{z} = f + r_A. \end{aligned}$$

For this equation to be related to regression, we require at least $\mathbb {E} r_A = 0$ and ${\text {Cov}} \, (f,r_A) = 0$. In the upcoming technical conditions, we will require the additional assumption that *f* and $r_A$ are independent. Since independence cannot hold if the covariance is non-zero, we investigate this property more fully here.

The following lemma, which gathers several technical results that we need, shows that the requirement ${\text {Cov}} \, (f,r_A) = 0$ is equivalent to $A \Lambda = I_{d_f}$, i.e., that *A* is a left inverse of $\Lambda$. Interestingly, this is also a central requirement in the recently developed “structural after measurement” approach of Rosseel and Loh (2022). The lemma shows that Thurstone factor scores do not have an uncorrelated measurement error term, but Bartlett factor scores do. Since the Bartlett (1937) score is a generalized least squares estimate (GLS), it shares the standard optimality properties of GLS. The optimality of Bartlett scores in the least squares sense in the class of conditionally unbiased factor scores is well known. The following lemma shows that the class of conditionally unbiased factor scores is the same class as factor scores with uncorrelated measurement errors, and both are characterized by the previously mentioned left inverse property. We make the following standard assumptions, whose motivation is recalled in Appendix E.1 in the online supplementary material.

### Assumption 1

Suppose Eq. (2) holds and that $\eta$ and $\xi$ has at least two finite moments. Further suppose

1. $\mathbb {E} \varepsilon = 0$, and the cross-covariance matrix ${\text {Cov}} \, (f,\varepsilon ) = \mathbb {E} [(f - \mathbb {E} f) \varepsilon ']$ is zero.
2. $\Lambda$ has full column rank.
3. $\Phi = {\text {Cov}} \, (f)$ is positive definite.
4. $\Psi = {\text {Cov}} \, (\varepsilon )$ is positive definite.

Let $\mathcal {G}(\Lambda )$ be the set of all left-inverses of $\Lambda$. That is, $A\in \mathcal {G}(\Lambda )$ means $A \Lambda = I_{d_f}$.

### Lemma 1

Suppose given Assumption 1.

1. Let *A* be a deterministic matrix, and let $r_A = A \tilde{z} - f$. Then ${\text {Cov}} \, (f,r_A) = 0$ if and only if $A \in \mathcal {G}(\Lambda )$. This holds also if $\Psi$ is singular.
2. Let *A* be a deterministic matrix. If $\mathbb {E} [\varepsilon |f] = 0$, then $\mathbb {E} [A \tilde{z} | f] = f$ if and only if $A \in \mathcal {G}(\Lambda )$.
3. The transformation matrix $T = \Phi \Lambda ' \Sigma ^ {-1}$ used in the Thurstone factor score exists, but is not in $\mathcal {G}(\Lambda )$.
4. The Bartlett matrix $\Delta = (\Lambda ' \Psi ^ {-1} \Lambda )^ {-1} \Lambda ' \Psi ^ {-1}$ exists and is in $\mathcal {G}(\Lambda )$, and is such that for all $A \in \mathcal {G}(\Lambda )$ we have that ${\text {Cov}} \, (r_\Delta ) - {\text {Cov}} \, (r_A)$ is non-positive definite.

### Proof

See Section E.4.1. $$\square $$

The set of left inverses of $\Lambda$ is non-empty if and only if $\Lambda$ has full column rank (Harville, 1997, Lemma 8.1.1). Therefore, Assumption 1 (2) is foundational. Assumption 1 (4) can be avoided, see, e.g., Eq. (7) in Wall and Amemiya (2000) and Fuller (1987) for a Bartlett formula that avoids inverting $\Psi$. We will not consider singular $\Psi$ matrices in this paper. Further, since $\Lambda$ is assumed to have full column rank, the set of left inverses $\mathcal {G}(\Lambda )$ equals the set of generalized inverses of $\Lambda$ (Harville, 1997, Lemma 9.2.8). This set can be described constructively, see Theorem 9.2.7 in Harville (1997). Due to Lemma 1 (4), we single out the Bartlett factor score out of the elements from $\mathcal {G}(\Lambda )$ in most of our study.

In applications, the transformation matrix *A* has to be estimated. This introduces estimation error, as discussed in the upcoming Sect. 3. Taking this estimation error into account is outside the scope of this paper.

Let us now consider the regression representation in Eq. (3). For a given $A \in \mathcal {G}(\Lambda )$, such as the Bartlett score $A = \Delta$, we write $r = r_A$ and

4 $$\begin{aligned} \ddot{f} = (\ddot{\xi }', \ddot{\eta }')' = A \tilde{z} = A (\Lambda f + \varepsilon ) = f + r = (\xi ', \eta ')' + (r_\xi ', r_\eta ') \end{aligned}$$

where $r_\xi , r_\eta$ are, respectively, the first $d_\xi$ and last $d_\eta$ coordinates of *r*, and $\ddot{\xi }$, $\ddot{\eta }$ are respectively the first $d_\xi$ and last $d_\eta$ coordinates of $\ddot{f}$. From Eq. (4), we reach

5 $$\begin{aligned} \ddot{\xi }= \xi + r_\xi , \qquad \ddot{\eta }= \eta + r_\eta . \end{aligned}$$

Since $A\in \mathcal {G}(\Lambda )$, we have that $r=A\varepsilon$. Now, since $r_\eta$ is a linear transformation of *r*, we get $\mathbb {E} [\ddot{\eta }| \xi ] = \mathbb {E} [\eta | \xi ]$ as long as $\varepsilon$ is independent to *f*. We therefore make the following assumption.

### Assumption 2

Suppose $\varepsilon$ is independent to *f*.

In the classical literature on covariance models (see, e.g., the survey paper Shapiro, 2007), the strong Assumption 2 is not made. We need this assumption, and not merely the covariance Assumption 1 (1) to identify *H*. With only covariance restrictions the distribution of $f,\varepsilon$ is not identified (see Mardia et al., 1979, Exercise 9.2.2). Also Kelava et al. (2017) made this assumption to identify *H*.

### Lemma 2

Suppose given Assumption 1 and 2. For a given $A \in \mathcal {G}(\Lambda )$, we have that $H(x) = \mathbb {E} [ \eta | \xi = x] = \mathbb {E} [ \ddot{\eta }| \xi = x ]$ for $\ddot{\eta }$ given by Eq. (4) and Eq. (5).

### Proof

See Section E.4.2. $$\square $$

Under the following assumption, $\ddot{\xi }$ and $\ddot{\eta }$ are computable based on identifiable quantities. Therefore, we may suppose that we observe $\ddot{\xi }$ and $\ddot{\eta }$ directly when analyzing identification of *H*.

### Assumption 3

Suppose $\Lambda$, $\mu$, $\Psi$ are identified from the distribution of *z*.

The identification of these matrices is a classical problem, and the measurement model is usually only considered valid when they are identified up to scaling of the latent variables through the covariance matrix of *z* (Anderson, 2003; Bollen, 1989; Mardia et al., 1979). However, conditional expectations are well-behaved with regard to changes of the units of measurement (see Appendix H in the online supplementary material).

We are interested in identifying *H*(*x*) based on $\ddot{\xi }$ and $\ddot{\eta }$. Using Lemma 2, Eq. (5) has the same structure as a non-parametric regression problem with measurement noise, see, e.g., Delaigle et al. (2009); Delaigle (2014); Apanasovich and Liang (2021), or Huang and Zhou (2017). This appears to be noticed also by Wall & Amemiya (2000, see the discussion immediately following their Eq. (9)), but at the time that paper was written, no generally applicable non-parametric regression methods with measurement error were available. These approaches generally need a known distribution and independence conditions to hold for the measurement error $r_\xi$, which has to be independent noise. Therefore, we make the following additional assumptions.

### Assumption 4

Suppose

1. $r_\xi$ has a known distribution.
2. $r_\xi$ and $r_\eta$ are independent.

We will later consider approximating the measurement error $r_\xi$ by zero, meaning we ignore the measurement error, and will show that this approximation works as $d_x$ increases. In these arguments, we also use Assumption 4. When ignoring measurement errors, we conjecture that exact independence can be weakened to appropriate dependence bounds. We do not investigate this in the present paper.

The following result is the starting point of the literature on non-parametric regression with measurement error with some papers cited above. We state the result with our notation and provide its short proof for completeness.

### Proposition 1

Suppose given Assumption 1, 2, 3, and 4. Then *H* is identified.

### Proof

See Section E.4.3. $$\square $$

We now consider when Assumption 4 (2) can be justified. Assumption 4 (1) will be considered in the next subsection.

Since $(r_\xi ', r_\eta ')' = r = A \varepsilon$ we have $r_\eta = ( \varvec{0}_{d_\eta ,d_\xi }, I_{d_\eta } ) A \varepsilon$ and $r_\xi = ( I_{d_\xi }, \varvec{0}_{d_\xi , d_\eta }) A \varepsilon$, where $\varvec{0}_{a,b}$ is the $a\times b$ zero matrix. Unless strong distributional assumptions are made, $r_\xi$ and $r_\eta$ will not be independent unless firstly *A* is partitioned diagonal (thereby avoiding cross terms from $\varepsilon _x$ and $\varepsilon _y$), and secondly $\varepsilon _x$ is independent of $\varepsilon _y$. If this is the case, i.e., if

$$\begin{aligned} A = \begin{pmatrix} A_x &{} \varvec{0}_{d_\xi , d_y}\\ \varvec{0}_{d_\eta , d_x} &{} A_y \end{pmatrix}, \end{aligned}$$

then $r_\xi = A_x \varepsilon _x$ and $r_\eta = A_y \varepsilon _y$, and $r_\xi$ will be independent to $r_\eta$, as long as $\varepsilon _x$ and $\varepsilon _y$ are independent.

In general, we may write $\Lambda$ as a partitioned matrix

$$\begin{aligned} \Lambda = \begin{pmatrix} \Lambda _{x} &{} \Lambda _{x,y} \\ \Lambda _{y,x} &{} \Lambda _y \end{pmatrix} \end{aligned}$$

where $\Lambda _x$ is a $d_x \times d_\xi$ matrix, $\Lambda _y$ is a $d_y \times d_\eta$ matrix, $\Lambda _{x,y}$ is a $d_x \times d_\eta$ matrix, and $\Lambda _{y,x}$ is a $d_y \times d_\xi$ matrix. When *A* is a partition diagonal matrix with diagonal matrix entries $A_x, A_y$, we have

6 $$\begin{aligned} (\ddot{\xi }', \ddot{\eta }')' = A \tilde{z} = A (\Lambda f + \varepsilon ) = \begin{pmatrix} A_x \Lambda _x \xi + A_x \Lambda _{x,y} \eta + A_x \varepsilon _x \\ A_y \Lambda _y \eta + A_y \Lambda _{y,x} \xi + A_y \varepsilon _y \end{pmatrix}. \end{aligned}$$

The matrix $A_x$ will not in general be a generalized inverse of both $\Lambda _x$ and $\Lambda _{x,y}$. Therefore, the factor scores will contain residual dependency between $\xi$ and $\eta$ which distort the identification of *H* as then Assumption 4 (2) no longer holds, if used directly as input to non-parametric regression methods.

In order to fulfill Assumption 4 (2), we, therefore, do not allow cross-loadings or error correlations between endogenous and exogenous parts of the model, that is between $\xi$ and $\eta$. If such are part of the model, one would have to delete corresponding observed variables in order to directly apply our analysis. A less wasteful method might hopefully be derived as an extension of this work, though such an extension is outside the scope of the present paper. Within the measurement part of the endogenous and exogenous variables, cross-loadings or error correlations are allowed. Hence, we make the following assumptions.

### Assumption 5

Suppose $\varepsilon _x$ and $\varepsilon _y$ are independent, and that

$$\begin{aligned} \Psi = \begin{pmatrix} \Psi _{x} &{} \varvec{0}_{d_x, d_y}\\ \varvec{0}_{d_y, d_x} &{} \Psi _y \end{pmatrix}, \quad \Lambda = \begin{pmatrix} \Lambda _{x} &{} \varvec{0}_{d_x, d_\eta }\\ \varvec{0}_{d_y, d_\xi } &{} \Lambda _y \end{pmatrix}, \end{aligned}$$

where $\Lambda _{x}$ and $\Lambda _{y}$ have full column ranks.

Under Assumption 1 (4), $\Psi _x, \Psi _y$ are positive definite, as they are principle sub-matrices of a positive-definite matrix $\Psi$ (Horn & Johnson, 2013, Observation 7.1.2). Under Assumption 5, a direct calculation shows that if $A_x \in \mathcal {G}(\Lambda _x), A_y \in \mathcal {G}(\Lambda _y)$, then $$A = \begin{pmatrix} A_x &{} \varvec{0}_{d_\xi , d_y}\\ \varvec{0}_{d_\eta , d_x} &{} A_y \end{pmatrix} \in \mathcal {G}(\Lambda )$$. While there are also elements in $\mathcal {G}(\Lambda )$ of different forms (Harville, 1997, Exercise 9.7), partitioned diagonal generalized inverses of $\Lambda$ imply that Assumption 4 (2) holds.

### Lemma 3

Suppose given Assumption 1 and 5. Suppose that $$A = \begin{pmatrix} A_x &{} \varvec{0}_{d_\xi , d_y}\\ \varvec{0}_{d_\eta , d_x} &{} A_y \end{pmatrix}$$ where $A_x \in \mathcal {G}(\Lambda _x)$ and $A_y \in \mathcal {G}(\Lambda _y)$. Then $r_\xi$ and $r_\eta$ are independent, i.e., Assumption 4 (2) holds.

### Proof

See Section E.4.4. $$\square $$

Under Assumption 5, Bartlett (1937) factor scores are partitioned diagonal as shown in the following result, and hence using Bartlett scores under Assumption 5 leads to Assumption 4 (2).

### Proposition 2

Suppose given Assumption 1 and 5. Then,

$$\begin{aligned} \Delta = \begin{pmatrix} \Delta _x &{} \varvec{0}_{d_\xi , d_y}\\ \varvec{0}_{d_\eta , d_x} &{} \Delta _y \end{pmatrix}, \end{aligned}$$

with $\Delta _x:= \left( \Lambda _x'\Psi _{x}^ {-1} \Lambda _x\right) ^ {-1} \Lambda _x'\Psi _x^ {-1}$ and $\Delta _y:= \left( \Lambda _y'\Psi _y^ {-1} \Lambda _y\right) ^ {-1} \Lambda _y'\Psi _y^ {-1}$ both existing. Additionally,

$$\begin{aligned} {\text {Cov}} \, r=\begin{pmatrix} \left( \Lambda _x'\Psi _{x}^ {-1} \Lambda _x\right) ^ {-1} &{} \varvec{0}_{d_\xi , d_\eta }\\ \varvec{0}_{d_\eta , d_\xi } &{} \left( \Lambda _y'\Psi _y^ {-1} \Lambda _y\right) ^ {-1} \end{pmatrix}, \end{aligned}$$

which is positive definite, and whose diagonal partitions are positive-definite matrices.

### Proof

See Section E.4.5. $$\square $$

We now consider Assumption 4 (1). We examine two approximations for sufficiently large $d_x$: Firstly, that $r_\xi$ is approximately zero, and secondly that $r_\xi$ is approximately normal. Since $r_\xi$ will go to zero as $d_x$ increases under weak assumptions, asymptotic normality is closely connected to techniques that treat $r_\xi$ as zero, and a normality approximation can potentially improve approximations of *H*. This issue will be further discussed though not resolved at the end of Sect. 2.2.

### Distributional Approximations of $r_\xi$ as $d_x$ Increases, Part 1: Approximating $r_\xi$ by a Constant Zero Vector

We here consider Assumption 4 (1). For fixed $d_x$, the distribution of $r_\xi$ is not identified, but under weak conditions, the distribution of $r_\xi$ will go to zero in mean square. This motivates approximating $r_\xi$ by a zero vector.

Mean square convergence of factor scores has been investigated by several previous authors, e.g., Guttman (1955), Schneeweiss and Mathes (1995), Krijnen (2004), Krijnen (2006b), Krijnen (2006a), or Williams (1978). To the best of our knowledge, previous papers either assume a particularly simple structure for the factor model, or used what may be termed abstract assumptions, such as limiting consideration of a certain eigenvalue for a matrix which is difficult to interpret. We, therefore, provide this conclusion based on alternative assumptions that have a more direct asymptotic interpretation as $d_x \rightarrow \infty$. We only consider the Bartlett (1937) factor scores.

Let $(M)_{\cdot , i}$ be the *i*’th column of a matrix *M*, and $(M)_{j,i}$ be the *j*, *i*’th element of a matrix *M*. Also, $\lambda _{\text {max}}(M)$ and $\lambda _{\text {min}}(M)$ are the, respectively, largest and smallest eigenvalues of a matrix *M*.

#### Assumption 6

Suppose

1. for all $d_x$, there are numbers $m_{\Psi _x},M_{\Psi _x} > 0$ such that $m_{\Psi _x}<\lambda _{\text {min}}(\Psi _x)\le \lambda _{\text {max}}(\Psi _x) < M_{\Psi _x}$.
2. for all $d_x$, there are numbers $m_{\Lambda _x}, M_{\Lambda _x} > 0$ such that for all indices $1 \le i \le d_\xi$ and all $1\le k$ where $(\Lambda _x)_{k,i} \ne 0$, we have, that $m_{\Lambda _x}< |(\Lambda _x)_{k,i}| < M_{\Lambda _x}$.
3. for $N_i$ being the number of non-zero elements in $(\Lambda _x)_{\cdot ,i}$ that $\lim _{d_x \rightarrow \infty } N_i = \infty$ for $1 \le i \le d_\xi$.
4. for $C_{i,j}$ for $1 \le i,j \le d_\xi$ with $i \ne j$ being the number of non-zero elements in $(|(\Lambda _x)_{k,i} (\Lambda _x)_{l,j} (\Psi _x^ {-1} )_{k,l}|)_{1 \le k,l \le d_x}$, that $\lim _{d_x \rightarrow \infty } \frac{1}{N_i} \sum _{1 \le j \le d_\xi , j \ne i} C_{i,j} = 0$.

Assumption 6 (1) extends Assumption 1 (4) to the asymptotic case and can be interpreted using the classical result that for a vector *x* with $\Vert x \Vert = 1$, we have $\lambda _{\text {min}}(\Psi _x) \le x' \Psi _x x \le \lambda _{\text {max}}(\Psi _x)$. Assumption 6 (1), therefore, dictates that no linear combination with a unit squared coordinate sum has a variance that diverges or converges to zero. This assumption requires that the variances of $\varepsilon _x$ are within a bounded interval and bounded away from zero. It, further, places restrictions on the correlations between the elements in $\varepsilon _x$. For a familiar example, let $x = (1, \ldots , 1)'/\sqrt{d_x}$, which is such that $\sum _{i=1}^{d_x} x_i^2 = 1$, giving $x' \varepsilon _x = \sqrt{d_x} \bar{\varepsilon }_x$ whose variance can neither diverge nor converge to zero if, for example, the effect of the central limit theorem for $\bar{\varepsilon }_x$ is to occur. Assumption 6 (2) says that the loadings of the measurement of each coordinate of $\xi$ (i.e., those that are non-zero) must neither vanish nor explode. Assumption 6 (3) says that the number of measurements of each coordinate of $\xi$ are continually increasing, thereby giving more and more information on $\xi$. Assumption 6 (4) places restrictions on the increase of the number of cross-loadings and cross correlations in relation to the number of direct loadings.

#### Proposition 3

Suppose Assumption 1, 2, and 5 hold, and let $A = \Delta$.

1. Suppose Assumption 6 (1) and (2) hold and let $N_i$ and $C_{i,j}$ be defined as in Assumption 6 (3) and (4), respectively, then
  $$\begin{aligned} \max _{1 \le i,j \le d_\xi } |({\text {Cov}} \, r_\xi )_{i,j}| \le \left[ \min _{1 \le i \le d_\xi } N_i \left( \frac{m_{\Lambda _x}^2}{M_{\Psi _x}} - \frac{M_{\Lambda _x}^2}{m_{\Psi _x}} \frac{1}{N_i} \sum _{1 \le j \le d_\xi , j \ne i} C_{i,j} \right) \right] ^ {-1} . \end{aligned}$$
2. Suppose Assumption 6 holds, then $\lim _{d_x \rightarrow \infty } \max _{1 \le i,j \le d_\xi } ({\text {Cov}} \, r_\xi )_{i,j} = 0$.

#### Proof

See Section E.4.6. $$\square $$

We now consider convergence of the conditional expectation of the population Bartlett factor score $\ddot{\eta }$ given the population Bartlett factor score $\ddot{\xi }$. When inputting samples of these into non-parametric regression methods, the methods consistently estimate $H_{d_x}(x) = \mathbb {E} [\ddot{\eta }| \ddot{\xi }= x]$ which will not equal $H(x) = \mathbb {E} [\eta | \xi = x]$ for fixed $d_x$. We here show that $H_{d_x}$ converges to *H* uniformly over an appropriately chosen subset. The implication of this is that non-parametric estimators based on population Bartlett factor scores will converge to *H* as the number of measurements $d_x$ increases over the chosen set.

Since conditional expectation of a vector is defined coordinate-wise, so that e.g., $\mathbb {E} [\eta | \xi ] = ( \mathbb {E} [\eta _1 | \xi ], \ldots , \mathbb {E} [\eta _{d_\eta } | \xi ])'$, we may without loss of generality assume that $d_\eta = 1$, since all norms on $\mathbb {R}^{d_\eta }$ are equivalent and $d_\eta$ is fixed.

We have not managed to find a result that implies the appropriate convergence of these conditional expectations and have therefore produced the following result. It seems plausible that relevant, and possibly stronger results could be available in the technical probabilistic literature. As $d_x$ increase, $\ddot{\xi }$ will under natural conditions be close enough to $\xi$ for $\mathbb {E} [\ddot{\eta }| \ddot{\xi }= x]$ and $H(x) = \mathbb {E} [\eta | \xi = x]$ to be very close, likely also under much weaker conditions than we identify in the upcoming result, which is based on classical approximations.

Our result requires *f* to have a density and poses several regularity conditions on the density of $\xi$, as well as some boundedness and smoothness conditions on *H*. Additionally, it requires that $r_\xi$ converges to zero in probability, which is implied by Proposition 3 and Markov’s inequality.

Let $\Vert a \Vert _2$ be the Euclidean norm of a vector *a*.

#### Assumption 7

Suppose

1. $d_\eta = 1$, and that $f = (\xi ', \eta ')'$ and $r_\xi$ have densities with respect to Lebesgue measure given by $f_{\xi , \eta }$ and $f_{r_\xi }$, respectively.
2. $\sup _{x \in \mathbb {R}^{d_\xi }} f_{\xi }(x) < \infty$, where $f_\xi$ is the marginal density of $\xi$.
3. there is a set $\mathcal {S} \subseteq \mathbb {R}^{d_\xi }$ such that for $\mathcal {S}^\rho = \{ x + \alpha (x - v): x \in \mathcal {S}, v \in \mathbb {R}^{d_\xi }, \Vert v\Vert _2 < \rho , \alpha \in [0,1] \}$ for an $\rho > 0$ we have that
  - $$\sup _{x \in \mathcal {S}^\rho } | \mathbb {E} \omega (x,r_\xi )| \rightarrow 0$$ as $$d_x \rightarrow \infty $$, where $$\omega (x,h) = H(x-h)-H(x)$$.
  - $$\sup _{x \in \mathcal {S}^\rho } |H(x)| < \infty $$
  - $$\inf _{x \in \mathcal {S}^\rho } f_{\xi }(x) > 0$$,
  - $f_\xi$ is continuously differentiable in $\mathcal {S}^\rho$, and $\sup _{x \in \mathcal {S}^\rho } \Vert f_\xi '(x)\Vert _2 < \infty$.
4. $r_\xi$ converges in probability to zero as $d_x$ increases.

Assumptions 7 (1) and (2) suppose the desired densities. Assumption 7 (3) is the most complex assumption and is given in terms of a kind of modulus of continuity of *H*. A verification of this assumption for a specific class of *H* functions requires taking the structure of this class into account. The assumption itself can be justified as a kind of smoothness assumption on *H*. To illustrate that the assumption is reasonable, we verify it for the class of univariate polynomials in Appendix E.2 in the online supplementary material. Assumption 7 (4) is implied, for example, by Proposition 3. Consequently, Assumption 7 allows the proof of the following proposition considering the convergence of $H_{d_x}$ to *H* for increasing $d_x$. Finally, Proposition 3 implies Assumption 7 (4).

#### Proposition 4

Suppose given Assumption 1, 2, 4 and 7. Let $H_{d_x}(x) = \mathbb {E} [\ddot{\eta }| \ddot{\xi }= x]$ and $H(x) = \mathbb {E} [\eta | \xi = x]$. Let $|\cdot |$ be any norm on the relevant Euclidean space. Then $\sup _{x \in \mathcal {S}^\rho } | H_{d_x}(x) - H(x)| \rightarrow 0$ as $d_x \rightarrow \infty$.

#### Proof

See Section E.4.7. $$\square $$

#### Remark 1

Let us re-visit the Thurstone transformation *T*. From Lemma 1, $T \notin \mathcal {G}(\Lambda )$. However, we have, say $\breve{f}:= (\breve{\xi }', \breve{\eta }')':= T \tilde{z} = (T \Lambda ) f + T \varepsilon$. From Assumption 1 and 2 we have that $T \varepsilon$ is still mean zero and independent to *f*. Therefore, $\mathbb {E} [\breve{\eta }| \breve{\xi }] = T_y \Lambda _y \mathbb {E} [\eta |\breve{\xi }]$ where $T_y$ is defined analogously as $\Delta _x$. Since the Thurstone factor scores converge in probability (and mean square) toward the true latent variables under weak assumptions (see, e.g., Krijnen, 2006a; b), we get that $T \Lambda \rightarrow I$ as $d_y$ increases, and $\breve{\xi }\rightarrow \xi$ as $d_x$ increases. We see that the additional term $T_y \Lambda _y$ is due to *T* not fulfilling the regression equation Eq. (3) with an uncorrelated error term. Therefore, consistency requires $d_y \rightarrow \infty$, in contrast to the present analysis that requires only $d_x \rightarrow \infty$.

### Distributional Approximations of $r_\xi$ as $d_x$ Increases, Part 2: Approximating $r_\xi$ by a Normal

We now consider approximating $r_\xi$ by a normal, using a central limit theorem. In this section, we make strong assumptions to simplify the normality argument. Under approximate normality, de-convolution methods can be used that take into account the distribution of the noise in $\ddot{\xi }$ as an approximation to $\xi$. The strong assumptions of the present section are not needed for our justification of direct non-parametric estimates of $\mathbb {E} [\ddot{\eta }|\ddot{\xi }= x]$ as an approximation to $\mathbb {E} [\ddot{\eta }| \xi = x]$ as considered in the previous subsection, but they are needed in our justification of non-parametric de-convolution based methods, one of which we will consider in the main simulation study of the paper (the HZCV method).

For a partitioned diagonal $A \in \mathcal {G}(\Lambda )$, we have that $r_\xi = A_x \varepsilon _x$. For sufficiently large $d_x$, we can expect central limit effects to justify the approximation $r_\xi \overset{a}{\sim }\ N(0, A_x \Psi A_x')$. As $d_x$ increases, $r_\xi$ will typically converge to zero in the mean square, so that $A_x \Psi A_x'$ will tend to zero. Therefore, a re-scaling is required to prove a formal limiting result, as is the case for standard averages.

Let $A_{i, \cdot }$ be the *i*’th row of *A*. Let $r_i$ be the *i*’th coordinate of $r_\xi$. We have $r_i = A_{i, \cdot } \varepsilon _x = \sum _{j=1}^{d_x} a_{i,j} \varepsilon _{x, j}$. When $\varepsilon _x$ have independent components, the normality of $r_i$ can be analyzed via the Lindeberg–Feller or Lyapunov central limit theorems (see e.g. Billingsley, 1995, Section 27). In order to do this, detailed assumptions have to be made on the entries of *A*. To get concrete and simple assumptions, we provide a verification of the details of this argument only for the Bartlett factor score when the measurement model of $\xi$ has the following simplified structure. The following results can be generalized in many directions, and the approximate normality of $r_\xi$ holds also well outside these conditions, and will hold in most cases of practical interest.

#### Assumption 8

Suppose

1. $\varepsilon _x$ has independent components and $\Psi _x$ is a diagonal matrix.
2. $\Lambda _x$ has only one non-zero element per row. Without loss of generality, we further assume that the coordinates of $\tilde{x}$ are re-arranged in such a way that $\Lambda _x$ is partitioned diagonal.

Let $\mathcal {I}_j$ be the coordinates of *x* which measures the *j*’th coordinate number of $\xi$. Under Assumption 8, $|\mathcal {I}_j|$ is the number of non-zero rows in the *j*’th column of $\Lambda _x$, and $(\mathcal {I}_j)_j$ forms a disjoint sequence. In the result, recall that the upper left elements of $\Lambda$ equal $\Lambda _x$, as is also the case for $\Psi$ and $\Psi _x$.

#### Lemma 4

Suppose Assumptions 1, 2, and 8. Then

1. $$\Delta _x = \left( \frac{\lambda _{ji}}{\psi _{jj}\sum _{k=1}^{d_x}\frac{\lambda _{ki}^2}{\psi _{kk}}}\right) _{i,j, i=1,\dots ,d_\xi ,j=1,\dots ,d_x}$$.
2. The *j*’th coordinate of $r_\xi$ fulfills $r_j = \sum _{i\in \mathcal {I}_j}\frac{\lambda _{ij}}{\psi _{ii}\sum _{k=1}^{d_x}\frac{\lambda _{kj}^2}{\psi _{kk}}} \varepsilon _i$, for $j=1,\dots ,d_\xi$.
3. We have that ${\text {Cov}} \, r_\xi$ is the diagonal matrix with elements $d_{ii}:= \left( \frac{1}{\sum _{k=1}^{d_x}\frac{\lambda _{ki}^2}{\psi _{kk}}}\right)$ for $i=1,\dots ,d_\xi$.

#### Proof

See Section E.4.8. $$\square $$

The following assumptions provide enough regularity to use the Lyapunov central limit theorem.

#### Assumption 9

Suppose

1. for a $\delta > 0$ we have
  $$\begin{aligned} \sup _{j \ge 1} \mathbb {E} \left| \frac{\varepsilon _{x,j}}{\sqrt{\psi _{jj}}} \right| ^{2 + \delta } < \infty . \end{aligned}$$
2. there are finite numbers $0<m_{\lambda /\psi } \le M_{\lambda /\psi } < \infty$ such that $\left( \frac{\lambda _{ji}^2}{\psi _{jj}} \right) _{1 \le i \le d_\xi , 1 \le j \le d_x} \subset [m_{\lambda /\psi },M_{\lambda /\psi }]$.
3. as $$d_x \rightarrow \infty $$, $$|\mathcal {I}_j| \rightarrow \infty $$, for $$j=1,\dots ,d_\xi $$.

Assumption 9 (2) places restrictions on asymptotic behavior of the coefficients in front of $\varepsilon _i$ in the expression for $r_j$ in Lemma 4. Assumption 9 (3) means that we get more and more measurements for all coordinates of $\xi$.

Notice that under the simplified variance expression in Lemma 4 (3), conditions for mean square convergence of $r_\xi$ are implied by Assumption 9 (2), as the variance converges to zero as $d_x$ increases since the sum in the expression is greater than $d_x m_{\lambda /\psi }$.

We now formalize the aforementioned central limit theorem-based approximation.

#### Proposition 5

Under Assumption 1, 2, Assumption 8, and 9, we have

$$\begin{aligned} c_{d_x}' r_{\xi } \xrightarrow [{d_x} \rightarrow \infty ]{d} N(0,I). \end{aligned}$$

where $c_{d_x}' = (\sqrt{n_{d_x}(1)}, \ldots , \sqrt{n_{d_x}(d_\xi )})$ in which $n_{d_x}(i) = \sum _{j=1}^{d_x} \frac{\lambda _{ji}^2}{\psi _{jj}}$ for $i = 1, 2, \ldots , d_\xi$.

#### Proof

See Section E.4.9. $$\square $$

This result does not have implications for identification, as Proposition 5 also implies that $r_\xi$ converges to zero in probability. It may be, however, that using the approximate normality of $r_\xi$ improves approximations of *H* based on the distribution of the population factor scores. While we have been unable to prove this, this topic is further discussed in more technical detail in Appendix E.3 in the online supplementary material.

## Empirical Estimation Strategies

Section 2 treats the foundational topic of identification. We now consider empirical estimates by following a plug-in procedure where $\Delta z$ is replaced by $\hat{\Delta }\hat{z}$, where $\hat{z} = z - \hat{\mu }$ replaces all unknown parameters with parameter estimates from considering the measurement model in Eq. (2) as a confirmatory factor analysis model (CFA). This is a linear transformation of $(z', 1)'$ where the estimation error of the standard CFA estimators is of order $O_P(n^{-1/2})$ where *n* is the sample size (see, e.g., Satorra, 1989). Therefore, the empirical Bartlett factor score has the same structure as a residual in standard regression problems. The mathematics behind a full asymptotic analysis of non-parametric regression methods where the covariates have measurement error with a known distribution is highly technical already with independent and identically distributed data (see, Delaigle et al., 2009, Section 1). In our case, we are inputting empirical factor scores, which as mentioned above have the same mathematical structure as regression residuals. Therefore, taking the estimation error of approximating $\Delta$ with $\hat{\Delta }$ properly into account is similar to using residuals in statistical methods, which can be mathematically complex (see, e.g., Grønneberg & Holcblat, 2019, and references therein). We consider an analysis of this problem outside the scope of the present paper.

Next to uncertainty in the estimation of $\hat{\Delta }$, the choice of the non-parametric regression method utilizing the computed factor scores will have an influence on the overall performance in approximating *H* in small samples. As there are many possible methods, we restrict attention to the most widely used methods and consider a more detailed examination outside the scope of the current article. We discuss the properties of the Bartlett factor scores derived in the previous section with regard to the choice in estimating *H* in the following.

The theoretical results of the previous sections imply that the residual *r* of the Bartlett factor score is close to normal (see Sect. 2.2) for sufficiently large $d_z$ and converges to zero in probability (see Sect. 2.1 and specifically Proposition 3). Further, the conditional expectation of the underlying latent variables is identifiable using the Bartlett factor scores (see Proposition 1, when independence assumptions among $r_\xi$ and $r_\eta$ hold and the distribution of $r_\xi$ is known). Most of these results depend on convergence dependent on *n* (the sample size) or on the number of measurements of $\xi$ ($d_x$) or both. In the next section, we use simulation to study the finite sample properties of several non-parametric regression methods where we use the Bartlett factor scores as inputs. We compare the performance of these methods using the Bartlett factor scores with the performance of three methods using the nonlinear factor scores proposed by Kelava et al. (2017) as inputs.

For a finite sample, both *n* and $d_z$ are finite and $d_x < d_z \ll n$. Therefore, the Bartlett score $\ddot{f}=f+r$ ought to have significant residual variance ${\text {Var}} \, [r]>0$. In this scenario, the usage of the Bartlett score is closely related to non-parametric regression estimation with measurement error (Delaigle et al., 2009; Delaigle, 2014; Huang and Zhou, 2017), where the independent variable (here $\ddot{\xi }$) is allowed to have a residual (here $r_\xi$). Such methods require additional assumptions. Similarly to the arguments underlying Proposition 1, the distribution of $r_\xi$ is required to be known. However, from Proposition 5 we have that the distribution of $r_\xi$ is only approximately known as it is asymptotically normal. Unfortunately, there are no current methods available that enable an examination of the sample distribution of the measurement errors and $r_\xi$ (see Appendix I in the online supplementary material for a discussion). We, therefore, are interested in the performance of such a method using an approximate distribution for $r_\xi$ and focus on an adaption of the local polynomial estimator by Delaigle et al. (2009, DFC-estimator) proposed by Huang and Zhou (2017): the HZ-estimator (HZ for local linear estimators for solving errors-in-variables problems, see Appendix D.3 in the online supplementary material for more details). The HZ-estimator is less biased and more computationally stable compared to the originally proposed DFC-estimator as also suggested by some of our preliminary analyses.

Since for increasing numbers of measurements in the exogenous part of the model, the variance of $r_\xi$ decreases, measurement error in the factor scores can be ignored. There is a variety of methods that could be used to estimate trends within data non-parametrically that do not take measurement error into account. All results in the next sections could be influenced by these choices. We employed two commonly used methods to estimate non-parametric trends, namely the locally estimated scatter plot smoothing (LOESS) originating from its weighted version (LOWESS, Cleveland et al., 1992) proposed by Cleveland (1979, 1981) and a cubic smoothing spline function (Chambers and Hastie, 1992). Both methods were also used to model the nonlinear factor scores of Kelava et al. (2017) complemented by their implementation of a specific BSpline (De Boor, 1978) method in order to enable a fair comparison between the methods and rule out any performance influences induced by the non-parametric regression method. We do not examine the BSpline method based on Bartlett factor scores since there is no readily available implementation except for the script of Kelava et al. (2017). We did include it for the factor scores of Kelava et al. (2017) since it was their suggested method to estimate the conditional expectation.

Table 1 provides a high-level summary of the most important assumptions for the empirical estimators considered. Using Bartlett factor scores together with either LOESS or spline estimates (BFS in the table) is our recommended approach and the one with the least assumptions on the measurement model.

**Table 1:**
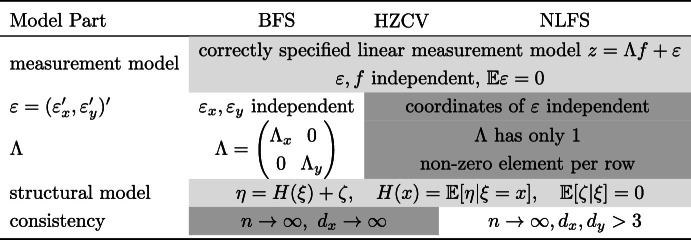
Assumptions used

BFS = Bartlett factor scores inputted into a general non-parametric trend estimate, HZCV = cross-validated HZ estimator, NLFS = nonlinear factor scores. The light gray area shows assumptions shared by all methods, the white region pertains to BFS, the dark gray region pertains to assumptions shared by two methods.

## Numerical Illustrations

All empirical analyses were done in R (R Core Team, 2023), except the nonlinear factor scores proposed by Kelava et al. (2017) which were estimated with a modified version of their MATLAB (The MathWorks Inc., 2023) scripts called from R including their used BSpline method. Appendix D.2 in the online supplementary material gives detailed information on the implementation, including the use of R-packages. A simple and practically minded numerical example is provided in Appendix A in the online supplementary material. All code and data used in the paper are available at the OSF-repository: https://osf.io/2xfh8/.

Within the following sections, we abbreviate the Bartlett factor scores (Bartlett, 1937) by BFS and the nonlinear factor scores by NLFS. None of the simulations are exhaustive due to the high computational cost of the cross-validated HZ-estimator and the estimation of the NLFS. Running the simulations of Sects. 4.3 and 4.4 on a 30 core cluster^1^ took about 26 days to complete even when limiting the number of replications to 200 per condition.

### The Distribution of $r_\xi$

**Fig. 1:**
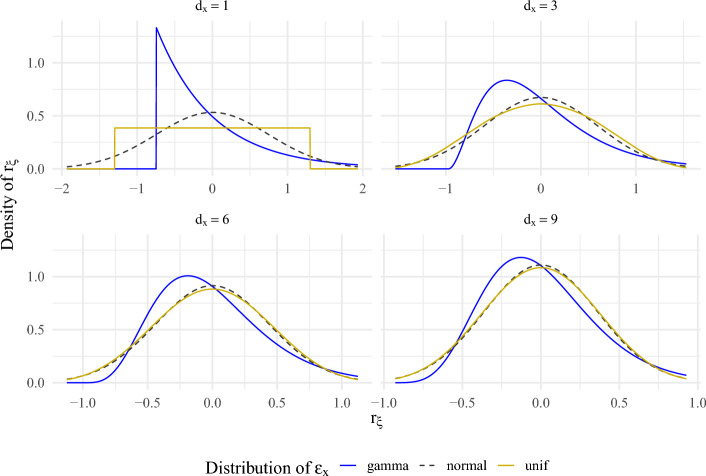
A comparison of the exact densities of $r_\xi$ resulting from the corresponding distribution of $\varepsilon _x$ with the relevant normal distribution suggested as an approximation.

We here illustrate the quality of the normal approximation of $r_\xi$. The normal approximation follows from the central limit theorem, and numerical illustrations of this effect are, therefore, well known. Hence, we only consider error distributions used in the proceeding simulation studies. We let $\varepsilon _x$ have independent and identically distributed coordinates and have marginal distributions given either by a standardized uniform or a standardized Gamma(1, 1). The exact distribution of $r_\xi$ is then known analytically (Moschopoulos, 1985; Kamgar-Parsi et al., 1995). Using these results, we produced Fig. 1. In it, we see that $r_\xi$ is close to normal in the uniform case for as few as 3 measurements, while more measurements are necessary for skewed gamma distributions, where deviations are easily seen even with 6 or 9 measurements. This indicates that approximating $r_\xi$ with a normal distribution is expected to work better for uniform errors than for gamma errors. Further, the plot also depicts the decreasing variance of $r_\xi$ with increasing $d_x$, as indicated by narrower distributions.

### A Visual Comparison of Approximations to *H*

In this section, we present average approximations to the conditional expectation $H(x)= \mathbb {E} [\eta | \xi =x]$ by the use of different methods. In order to examine the small sample and finite measurement properties of the methods, we simulated four different trends to be estimated non-parametrically for $d_\xi = d_\eta = 1$; quadratic, cubic, logit and piecewise linear. We chose $n=1000$, $d_x$ as 3 and 9 and all model parameters to coincide with the assumptions needed for Lemma 4, that is, there are no cross-loadings or residual covariances and all residuals are independent. All coefficients are chosen so that $\xi$ and $\eta$ have zero mean and unit variance. Further, we fixed the first factor loading within $\Lambda _x$ and $\Lambda _y$ to 1 (in the population and in the analyses) and the corresponding residual variances in $\Psi$ to .5625 to ensure that the corresponding reliability is .64. The remaining factor loadings per latent variable and the corresponding residual variances were chosen to have reliabilities that are equidistant between .64 and .25. These item-wise reliabilities are rather low, but realistic. We wanted to choose conditions under which there is substantial noise in the data. As this is a condition also analyzed in the following simulation study we refer to Appendix D.1 in the online supplementary material for additional information.

**Fig. 2:**
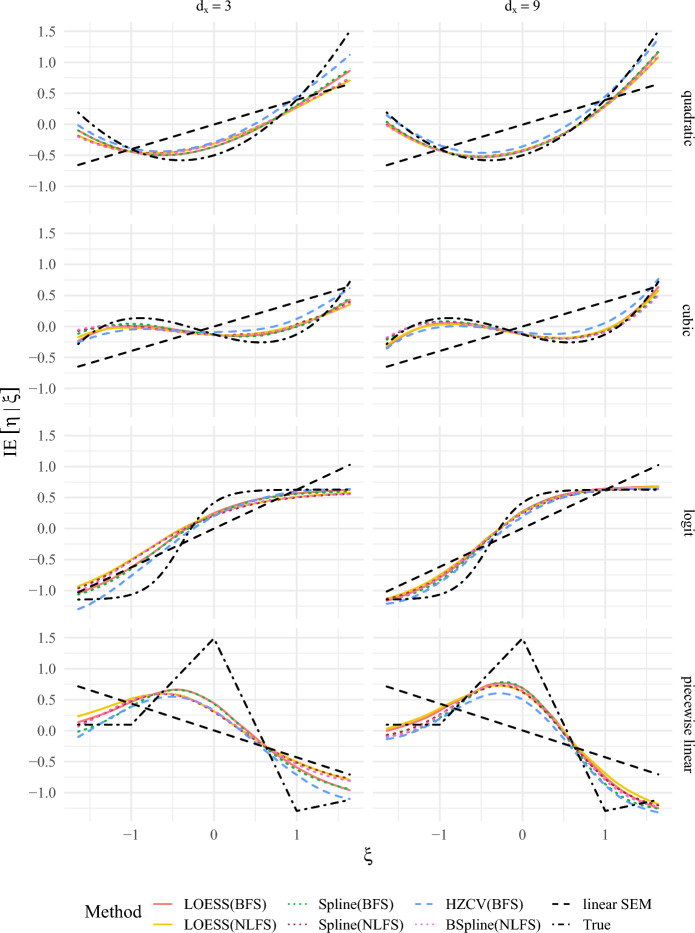
A comparison of nonparametric estimation for $\mathbb {E} [\eta | \xi ]$ averaged across 200 replications with $n=1000$ for the LOESS and the smoothed spline methods based on BFS and the NLFS, the HZ-estimator, the BSpline estimator based on NLFS compared to the true trend and a linear SEM estimation with different true trends (quadratic, cubic, logit and piecewise linear) and dimensions $d_x$ with normal $\xi$ and gamma distributed errors $\varepsilon$.

Figure 2 shows the average nonparametric estimation of *H* using either BFS or NLFS as inputs compared with the true trend and to a linear SEM estimation averaged across 200 replications for normal $\xi$ and gamma $\varepsilon$.

Figure 11 in Appendix D.4 in the online supplementary material extends Fig. 2 by coverage intervals. Both figures depict the convergence toward the true trend for increasing $d_x$ and make differences with regard to the trends evident. That is, smoother trends, such as the quadratic trend with a linear first derivative, are approximated with more precision from all methods compared with the other trends, which have stronger differences with regard to their rate of change, i.e., nonlinear first derivatives. All methods appear to be less precise at the edges of the support, which is expected as there are fewer data points present. Further, Fig. 2, and 11 in Appendix D.4 in the online supplementary material suggest that there are differences among the methods with the methods relying on the BFS slightly outperforming the NLFS.

In order to compare computational costs, we benchmarked the methods used within Fig. 2 for a cubic trend, see Appendix D.4 in the online supplementary material, Table 7. On a standard laptop, the LOESS and spline method based on BFS are extremely quick compared to all other methods, taking much less than 1 s. The HZ-estimator using simulation-based cross-validated bandwidth took more than 24 min and the methods based on NLFS took more than 35 min on average.

The nonparametric method of Kelava et al. (2017) sets the first factor loading per latent variable to one. This is done in the following simulations to make estimates comparable. See Appendix H in the online supplementary material for theoretical information on the scaling issue.

### Simulation Study Based on Mean Integrated Square Error for $d_\xi = 1$

We now consider a more systematic simulation-based comparison of the performance of nonparametric estimation methods based on BFS and NLFS. We evaluate the scenarios in the previous section in more detail and aggregate the performance by using the mean integrated squared error:

$$\begin{aligned} \text {MISE} = \mathbb {E} \Vert H - \hat{\varphi }\Vert _{\mathcal {A}_{\alpha ^\star }, 2}^2, \quad \text {where} \quad \Vert H - \varphi \Vert _{\mathcal {A}_{\alpha ^\star }, 2}^2 = \int _{\mathcal {A}_{\alpha ^\star }} \big [H(x) - \varphi (x)\big ]^2 dx, \end{aligned}$$

where the expectation is approximated by the empirical expectation over the number of replications. The integration area is limited, as lack of data near edges inflates the mean squared error but is of limited practical interest. We set $\mathcal {A}_{\alpha ^\star }$ as level sets $\{x: f_{\xi }(x) > c_\alpha ^\star \}$ where $c_\alpha ^\star$ is such that $P(\xi \in \mathcal {A}_{\alpha ^\star }) = 1 - \alpha ^\star$, where $\alpha ^\star = 10\%$.

For our simulation study using $d_\xi =1$, we extended the conditions of Sect. 4.2 by a crossed design for which we manipulated the number of items $d_x$ (3, 6, 9), the distribution of $\xi$ (normal or uniform with mean zero and unit variance), the distribution of $\varepsilon$ (centered normal, centered uniform, centered gamma, see Sect. 4.1), and the true trends (quadratic, cubic, logistic, piecewise linear). This resulted in a total of 72 conditions. 200 replications were used, with a sample size of $n=$ 1000. For a more detailed description of the simulation conditions and the data generating process, see Appendix D.1 in the online supplementary material. All conditions were analyzed using the following methods: linear SEM, LOESS using BFS and NLFS, smoothed splines using BFS and NLFS, the BSpline method using NLFS proposed by Kelava et al. (2017), and the cross-validated HZ-estimator using the BFS. In order to compare all results with a best case scenario, we also included LOESS and smoothed spline estimation using the true latent variables $f=(\xi , \eta )'$ as inputs.

**Fig. 3:**
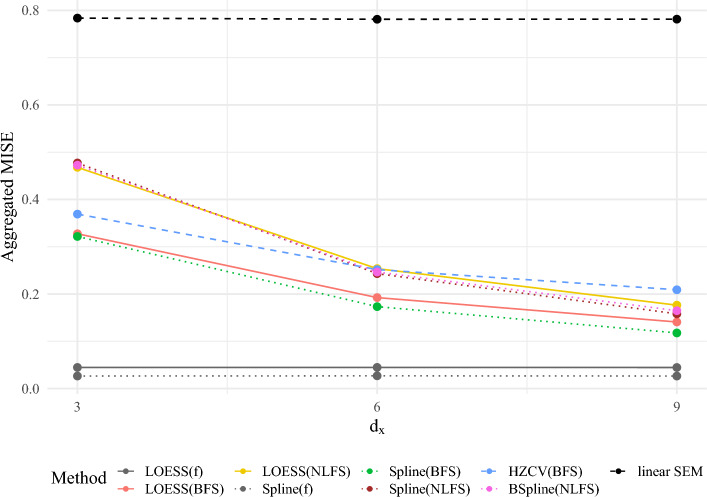
A comparison of the average MISE across 200 replications with $n=1000$ for different procedures [(B)Splines vs. LOESS vs. HZ/others] based on different inputs (BFS $\ddot{f}$, NLFS, the linear SEM, and the true latent variables *f* for comparison) for different dimensions $d_x$ aggregated across all distributions and trends used in the simulation study.

Figure 3 depicts the performance of the methods aggregated across all distributional conditions and all trends. As can be expected, the MISE for the linear SEM and the true latent variables *f* are not affected by $d_x$. Here, the linear SEM shows the highest and the methods based on *f* show the lowest MISE for all $d_x$ averaged across all distributional conditions and trends. All other methods show a decrease in MISE for increasing $d_x$. However, even for $d_x=9$ the MISE of all methods is considerably higher compared with using the true latents *f* as inputs for the nonparametric methods. This deviation quantifies approximation error arising from measurement and finite sample error. Concerning the methods of interest, for all $d_x$ the smoothed spline using the BFS as input showed the lowest MISE followed by the LOESS based on the BFS. Averaged across all distributional conditions and trends for $d_x=3$ the HZ-estimator outperformed the methods based on NLFS. These differences disappear for $d_x=6$ and reverse for $d_x=9$ indicating that for smaller variance of $r_\xi$ the HZ-estimator is less useful compared to the other methods. Within the methods based on NLFS, Figs. 3 suggests that the spline and BSpline approaches have smaller MISE compared to the LOESS. However, these differences are rather small. For $d_x=9$ the differences between the methods using BFS and the methods using NLFS appear to be considerably smaller than for $d_x=3$.

**Fig. 4:**
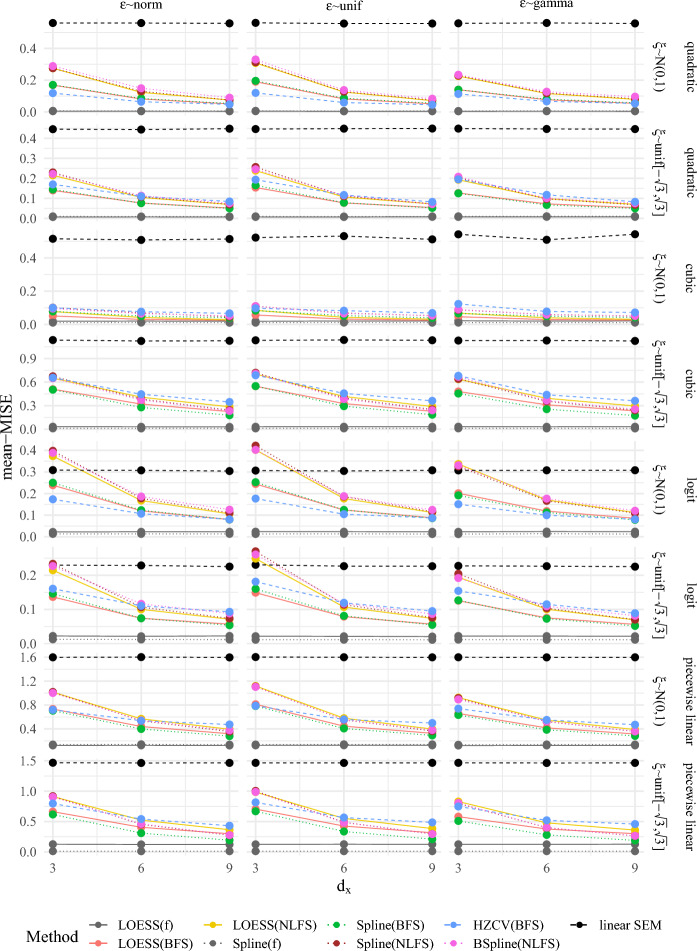
A comparison of the averaged MISE across 200 replications with $n=1000$ for different procedures [(B)Splines vs. LOESS vs. HZ/others] based on different inputs (BFS, NLFS, linear SEM, and true latent variables *f* for comparison) for four models with different true trends (quadratic, cubic, logit and piecewise linear) and dimensions $d_x$. See Table 8 and 9 in Appendix D.4 in the online supplementary material for numerical values.

Figure 4 shows the MISE across all conditions. Table 8 and 9 in Appendix D.4 in the online supplementary material displays the corresponding numerical values depicted in Fig. 4. These supplement Fig. 3 of the article by visualizing all MISE for all simulated conditions. For instance, it is evident for some conditions with a logit trend for $d_x=3$, that the MISE for methods based on NLFS was in fact higher than that of the linear SEM, indicating that the linear approximation was closer to the true trend than the non-parametric one based on NLFS. With increasing $d_x$, all methods showed lower MISE compared to the linear SEM. The distribution of $\xi$ also influences the performance of the methods. For instance, for normal $\xi$ and logit trend the HZ-estimator resulted in lower MISE compared to all other methods based on factor scores. For a cubic trend, this is reversed and all methods except for the linear SEM show a lower MISE compared to the HZ-estimator. All in all, for all scenarios and all $d_x$ the methods using the NLFS never had the smallest MISE, with the LOESS, the BSpline, and the smoothed spline method based on NLFS showing comparable MISE in almost all conditions. In most cases, spline and LOESS based on BFS showed the lowest MISE as already suggested by the aggregated results in Fig. 3. We note that the differences between the methods based on BFS and NLFS are small but consistent.

Interestingly, there are conditions for which LOESS showed lower MISE for the methods based on factor scores than the spline method, while the LOESS based on the true latent variables showed higher MISE than using splines in all conditions. Therefore, we cannot draw any conclusions from the performance of the methods using the true latents *f* as inputs, and they should only be used as a best case scenario and serve as an anchor for an expected smallest possible MISE (as $\ddot{f} \rightarrow f$ for $d_z \rightarrow \infty$; hence, the MISE cannot be smaller than that using *f*). For ease of comparison, Figure 12 in Appendix D.4 in the online supplementary material depicts the relative improvement of MISE in comparison with the linear SEM approximation (see also Table 10 and 11). These relative improvements show that the logit trend was closest to linearity since the improvement was the smallest. The cubic trend for normal $\xi$ showed the largest improvement, while for uniform $\xi$ for cubic trends the improvement was comparable to that of quadratic or piecewise-linear trends.

In order to visualize variation among the trends, Figure 13 in Appendix D.4 in the online supplementary material depicts box plots summarizing the MISE across all distributional conditions for each $d_x$ and each trend. The variation among MISE decreases with increasing $d_x$ and is comparable among the methods based on factor scores. The methods based on the true latent variables on average show the smallest variation. The cubic trend has the largest variation among the MISE across the distributional conditions, but the piecewise linear trend resulted in the largest average MISE.

To summarize, from our limited conditions within the simulation we may conclude that using BFS either with LOESS or smoothed splines will result in the smallest MISE and, therefore, in the best approximation of the true trend, while also being the cheapest with regard to computation time. The HZ-estimator was only beneficial in limited conditions, while the NLFS always showed higher MISE compared to LOESS or splines based on BFS.

### Simulation Study Based on Mean Integrated Square Error for $d_\xi = 2$

In this section, we extend the previous simulation results to models with $d_\xi =2$. As a multivariate implementation of the HZ-estimator is still missing, we did not include it in the simulation. Further, there are no simple multivariate extensions of the smoothed spline method, and we discarded it within the $d_\xi =2$ simulation study. This simulation study therefore only considers LOESS estimates of the trend as well as the BSpline approach as implemented by Kelava et al. (2017) for their NLFS.

We used a crossed design for which we manipulated the number of items per latent exogenous variable $\xi =(\xi _1,\xi _2)'$, the number of measurements per latent variable $d_{x_j}$ (3, 6, 9) for $j=1,2$, the distribution of $\xi$ (multivariate standard normal or normal copula with uniform marginals with mean zero, variance 1, and correlation .5), the distribution of $\varepsilon$ (centered normal, centered uniform, centered gamma), and the true trends (quadratic, cubic). Further, we manipulated the model specification, that is whether cross-relations, i.e., cross-loadings and residual covariances, among the measurements of $\xi$ or within the measurements of $\eta$ are present (uncrossed, crossed). This resulted in a total of 72 conditions. We used 200 replications, and a sample size of $n=$ 1000. For a more detailed description of the simulation conditions and the data generating process, see Appendix D.1 in the online supplementary material. We compared the following methods: linear SEM, LOESS using BFS or NLFS, and the BSpline method using NLFS proposed by Kelava et al. (2017). In order to compare all results with a best case scenario, we, again, included LOESS estimation based on the true latent variables $f=(\xi ', \eta )'$ as inputs.

The NLFS proposed by Kelava et al. (2017) assumes a linear factor model without cross-loadings or residual covariances among the items (recall Table 1). In order to examine the effect of a misspecified measurement model used to compute factor scores, we added Bartlett scores estimated without cross-relations as an additional condition, to have a fair comparison between the methods, as they are then both misspecified in these conditions. For a discussion and examples on the misspecification of the functional form of the factor models (i.e., nonlinear factor models) see Appendix F in the online supplement, which show that misspecification is not very sensitive for minor deviations from linearity. We call BFS_uc_ the Bartlett scores estimated using no cross-relations in the factor model.

**Fig. 5:**
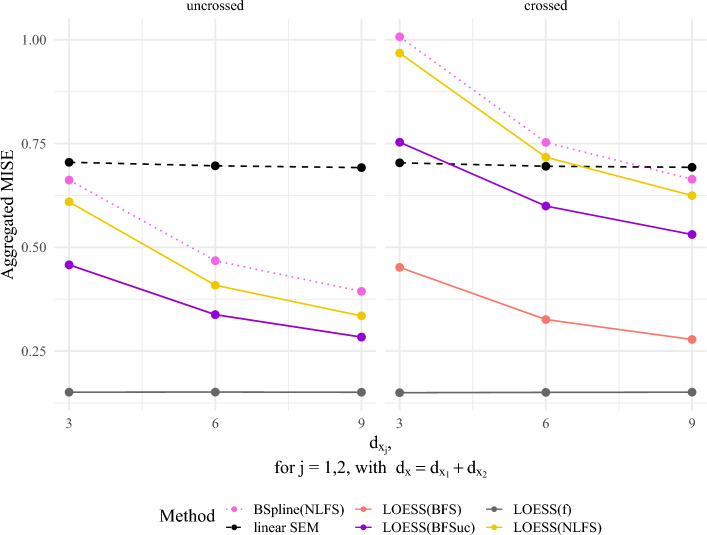
A comparison of the averaged MISE across 200 replications with $n=1000$ for different procedures [(B)Splines vs. LOESS vs. HZ/others] based on different inputs (BFS $\ddot{f}$, NLFS, the linear SEM, and the true latent variables *f* for comparison) for different dimensions $d_x$ aggregated across all distributions and trends used in the simulation study separated for conditions without (uncrossed) and including cross-loadings and cross correlations in $\Lambda _x, \Psi _x,$ and $\Psi _y$. BFS and BFS_uc_ are equivalent for uncrossed data.

Figure 5 shows aggregated MISE results across all distributional conditions, trends, and model specifications. Similarly to the $d_\xi =1$ case, MISE decreases for all methods based on factor scores for increasing $d_{x_j}$, while LOESS based on the true latents *f* and the linear SEM are not affected by $d_{x_j}$. It stands out that LOESS based on BFS using the correct model is not influenced by cross relations, while the NLFS as well as the BFS without these cross-relations show largely inflated MISEs for conditions where there are in fact cross-relations present. Still, the wrongly specified BFS_uc_ resulted in lower MISE compared to the methods based on the NLFS for conditions with present cross-relations. For conditions without cross-relations the LOESS based on BFS with and without are identical and, hence, overlap completely. The LOESS based on BFS outperforms the methods based on NLFS under all presented conditions.

**Fig. 6:**
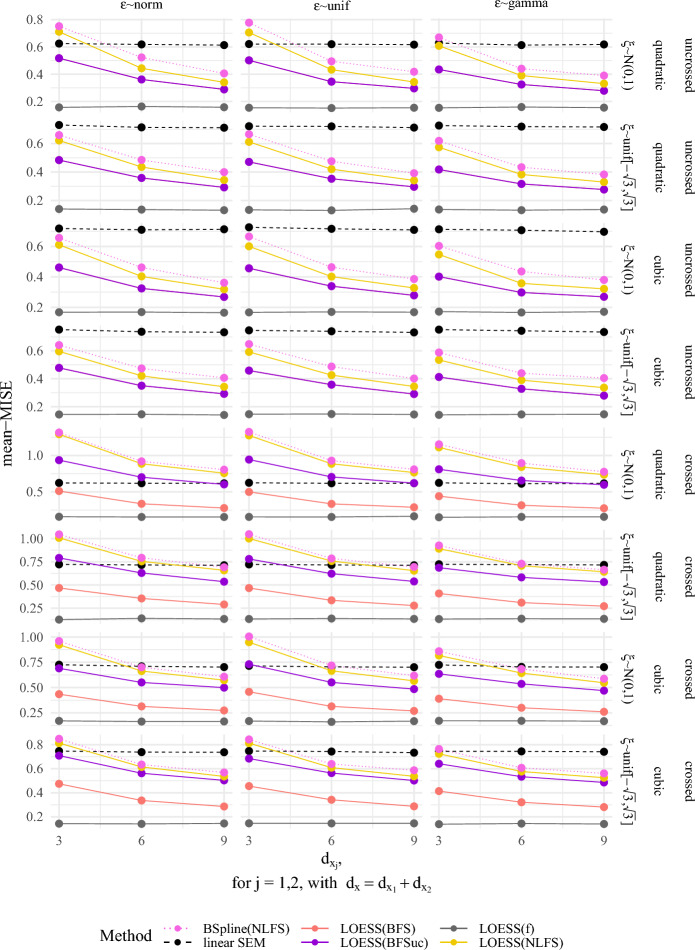
A comparison of the averaged MISE across 200 replications with $n=1000$ for different procedures [(B)Splines vs. LOESS vs. HZ/others] based on different inputs (BFS, NLFS, linear SEM, and true latent variables *f* for comparison) for two models with different true trends (quadratic and cubic), dimensions $d_{x_j}$, and inclusion of cross-relations (cross-loadings and cross-correlations in $\Lambda _x, \Psi _x,$ and $\Psi _y$) and distributions (row and column names refer to marginal distributions) used in the simulation study for $d_\xi = 2$. See Table 12 and 13 in Appendix D.4 in the online supplementary material for numerical values.

Figure 6 shows all average MISE across the 200 replications for all used conditions (see also Table 12 and 13 in Appendix D.4 in the online supplementary material for numerical values). From Fig. 6, it is evident that there are differences in the degree of poor performance of the method with regard to the distributions. The MISE did decrease for all methods based on factor scores with increasing $d_{x_j}$, but there are conditions where the MISE for methods using factor scores was considerably higher than that of the linear SEM. Compared to the linear SEM, the MISE in conditions with cross-relations was larger for BFS_uc_ and the methods based on NLFS for quadratic trends and especially for normal $\xi$ with the MISE being larger than that of the linear SEM even for $d_{x_j}=9$ for quadratic trends with normal $\xi$. Interestingly, with regard to measurement errors, gamma $\varepsilon$ resulted in the lowest MISEs. In the conditions without cross-relations still in all conditions LOESS based on NLFS outperformed the BSpline method. Further, LOESS based on BFS is considerably lower in MISE in all conditions without cross-relations compared to methods based on NLFS. These differences appear the smallest for cubic trends with marginally uniform $\xi$ with normal copula. The MISE of the methods based on factor scores was considerably higher than that of the LOESS based on *f*. Identically to the simulation with $d_\xi =1$ the MISE for the linear SEM or for the LOESS based on *f* was not related to $d_{x_j}$. This, of course, can be expected as for these objects $d_{x_j}$ has minimal influence on the estimated parameters.

Further, Figure 16 in Appendix D.4 in the online supplementary material emphasizes that the LOESS based on BFS is much more homogeneous in the MISE and, hence, in the performance in approximating the true trend. Additional information on the relative improvement in comparison with the linear SEM approximation is further given in this appendix stretching the importance of a correctly specified (linear) measurement model (see Figure 15 and Tables 14 and 15).

To summarize, from our limited conditions within the simulation we underline the results of the previous simulation study for $d_\xi =1$ with even stronger evidence in favor of the LOESS method based on the BFS as it showed the smallest MISE in all conditions, opens the opportunity to check whether the confirmatory factor analysis model used to extract the factor scores fits the data, is flexible with regard to cross-relations and has an extremely short runtime. We note that the differences between the methods based on BFS and NLFS are small but consistent, when all assumptions for NLFS are fulfilled. The differences between the methods decline for increasing $d_{x_j}$, but are never close to the performance of the LOESS based on *f* or to each other. Measurement model misspecification negatively affects all methods.

## Concluding Remarks

We may combine our foundational equations (1) and (2) to see that the full model is a multivariate non-parametric regression problem where both the dependent variable $\eta$ and independent variable $\xi$ are observed with measurement error. In theory, this non-parametric formulation can be worked with directly. However, the distributions of the error terms would be unknown and would neither be asymptotically normal nor vanishing as the number of measurements increases. With stronger assumptions the distributions of these measurement errors are identified and can be non-parametrically estimated, which would lead to methodology such as that suggested in Kelava et al. (2017) and Kohler et al. (2015).

Our approach avoids such estimation or a-priori specification of the error distribution through our use of factor scores. That is, we use a linearly optimal dimensionality reduction which has the advantage that the measurement error distribution is asymptotically known, thereby avoiding their estimation in order to non-parametrically estimate *H*.

Our simulation study has demonstrated that using Bartlett (1937) factor scores as inputs in non-parametric regression method is well-working and computationally efficient for non-parametric estimation in NLSEM. Specifically, employing LOESS or spline approaches based on Bartlett factor scores outperformed the other methods in nearly all conditions in our simulation study.

Our analyses have several limitations. In the theoretical contribution, the most striking limitations are that we only study population quantities, and that we only used linear factor scores taken from an assumed correctly specified linear factor model. Also, the assumptions of the asymptotic results can be weakened, and the assumptions we made on the factor scores can likely also be weakened to, for example, allow the use of Thurstone factor scores.

In the simulations, we limited attention to non-parametric estimators of *H*, and excluded the semi-parametric alternatives reviewed in Appendix C in the online supplementary material. Bauer et al. (2012) compared the performance of estimating trends using the semiparametric latent class approach of Bauer (2005) with the approach of inputting factor scores into non-parametric regression methods as dealt with in this paper and concluded that the latent class approach performed best in many settings. In further research, one could analyze the scope of the semi-parametric methods, i.e., identify which types of trends and distributional forms are supported in common situations (in the latent class situation this could be a small to moderate number of latent classes and within each $(\xi ', \eta ')'$ follows a linear and normal SEM), and compare the non-parametric approaches considered in the present paper with the semi-parametric methods both within and outside their scope.

Further limitations of our simulation study are that we only use a sample size of $n=1000$ observations with 200 replications. Expanding the sample size conditions could provide further insights into the performance of the methods. Expanding the replication number would sharpen our approximations. Furthermore, our simulation study solely considers symmetric distributions for the latent exogenous variable $\xi$, and we have not varied the number of measurements for the latent endogenous variable $\eta$.

## Supplementary Information

Below is the link to the electronic supplementary material.

Supplementary file 1 (pdf 4952 KB)

## Footnotes

1 For our simulation, we used 30 processes on a shared computer cluster with 2 sockets of AMD EPYC 7452 32-core Processors @2.35 GHz (64 physical cores and 128 logical cores) and 128GB RAM.

## Declarations

### Data Availability

All source code and data are included in the supplementary material at https://osf.io/2xfh8/
