# Supplementary material for: Non-parametric Regression Among Factor Scores: Motivation and Diagnostics for Nonlinear Structural Equation Models
Source: Psychometrika. 2024 Apr 23;89(3):822–50. doi: 10.1007/s11336-024-09959-4 (PMC11458680; doi:10.1007/s11336-024-09959-4)
Supplement: Supplementary file 1 — (pdf 4952 KB) [file 11336_2024_9959_MOESM1_ESM.pdf]

# ONLINE SUPPLEMENTARY MATERIAL: APPENDIX

By Steffen Grønneberg steffeng@gmail.com and Julien Patrick Irmer jirmer@psych.uni-frankfurt.de (shared first authorship).

Throughout the appendix we use several properties of conditional expectations and measure-theory, which are stated in Appendix K for completeness.

## Appendix A. A Simple and Practically Oriented Numerical Illustration

In this section, we consider a simple simulation-based illustration using a very simple model summarized in Figure 7. Example code in how to estimate the nonlinear trend using Bartlett factor scores utilizing LOESS is given at the end of this section.

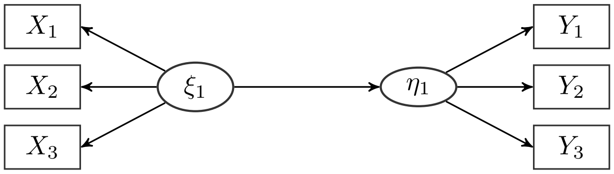


FIGURE 7. An example SEM path diagram, where arrows between latent variables and manifest variables indicate linear relationships, while arrows among latent variable indicate possible nonlinear relations. Residuals and measurement errors are not shown for simplicity, but are present in the model.

The arrow between $\xi_1$ and $\eta_1$ denote an influence, and it may be non-linear. Its equation is

$$\eta_1 = H(\xi_1) + \zeta_1, \qquad \xi_1, \sim \mathcal{N}(0,1), \zeta_1 \sim \mathcal{N}(0,.34) \text{ and independent of each other.}$$

Linear SEM assumes $H(x) = \gamma_1 x$. Instead, we will assume

$$H(x) = -0.5 + 0.4x + 0.5 * x^2$$

which is a clearly non-linear quadratic trend. The chosen parameters further imply Var $\eta = 1$.

The measurement model is linear, and given by

$$x_i = \lambda_{x,i}\xi_1 + \varepsilon_{x,i}, \quad y_i = \lambda_{y,i}\eta_1 + \varepsilon_{y,i}, \quad i = 1,2,3.$$

We let $\lambda_{x,1} = \lambda_{y,1} = 1$ fixed for identification and let $\lambda_{x,2} = \lambda_{y,2} = .65$ and $\lambda_{x,3} = \lambda_{y,3} = .5$. Further let $\varepsilon_x \sim \mathcal{N}(0, \Psi_x)$ and $\varepsilon_y \sim \mathcal{N}(0, \Psi_y)$, with Cov $\varepsilon_x = \Psi_x =$ Cov $\varepsilon_y = \Psi_y = \text{diag}(.5625, .5775, .75)$, where diag stacks the vector onto the diagonal of a corresponding square matrix. This is the same model setting as chosen for the simulation study, further described in Section 4 and Appendix D.

We drew a sample with sample-size $n = 200$. The simulated values of $(\eta_1, \xi_1)$ are shown in Figure 8. Standard linear SEM goodness of fit measures report $\chi^2_{df=8} = 10.11, p = 0.257$, $RMSEA = 0.036$, $SRMR = 0.030$, $CFI = 0.990$, indicating an appropriate fit. This failure of standard linear SEM estimations to detect non-linear deviations from the model is well-known, see e.g. Mooijaart and Satorra (2009). The trend we have chosen for the illustration is of a simple quadratic kind. There are

available tools to detect missing quadratic or interaction terms in SEM, such as the specification test of Nestler (2015) or significance tests for non-linear SEM (Büchner & Klein, 2020). We here illustrate how the non-linear trend can be detected using trend estimates based on factor scores.

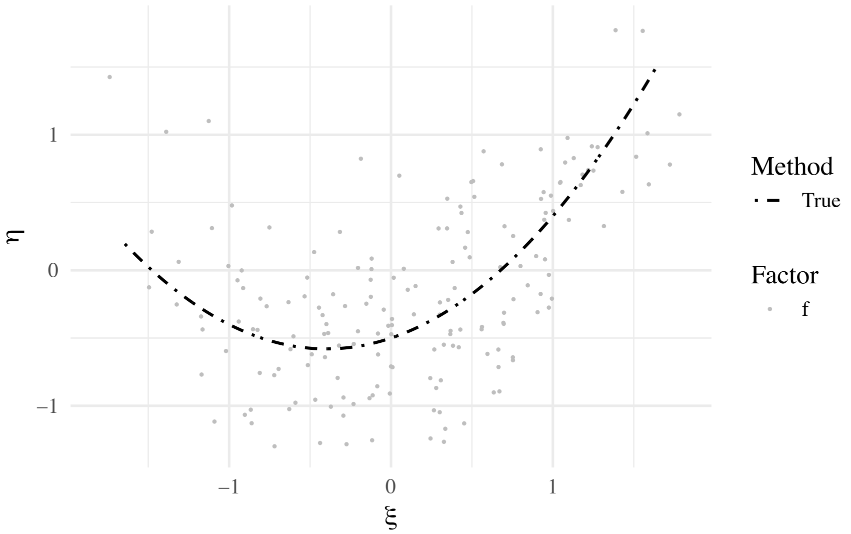


FIGURE 8. Simulated values of $(\eta_1, \xi_1)$ with the true trend $H$.

The data plotted in Figure 8 will never be known to us, and we need to use the manifest variables $x_1, x_2, x_3, y_1, y_2, y_3$ to approximate the latent variables. In Figure 9, we have plotted the Bartlett factor scores with trend estimates using the locally estimated scatterplot smoothing (LOESS) originating from its weighted version (LOWESS, Cleveland et al., 1992) proposed by Cleveland (1979, Cleveland, 1981), the cross-validated adaption of the local polynomial estimator by Delaigle et al. (2009, DFC-estimator) proposed by Huang and Zhou (2017): the HZ-estimator (HZ for local linear estimators for solving errors-in-variables problems, see Appendix D.3 for more details) specifically tailored for Bartlett factor scores assuming normality of the prediction residual of the score (see Section 3 for further information), and the nonlinear factor scores of Kelava et al. (2017) complemented by their implementation of a specific BSpline (De Boor, 1978) method. In this particular simulation, the LOESS(BFS) has the least mean integrated square error to the true trend line $H$, then HZCV(BFS), and finally BSpline(NLFS).

The plotted points of Figure 9 will only be an approximation to the true latent variables $f$ in Figure 8 due to the relation $\ddot{f} = f + r$ for the BFS. Individual realizations of the factors are not possible to re-gain exactly (for an overview of factor score indeterminacy see, e.g., Grice, 2001), even in the population. We caution against taking the individual factor scores as equal to the factors. The observed differences between Figures 8 and 9 illustrate the type of difference one might expect in an empirical study.

When studying the difference between the latent variables (Figure 8) and their approximation (Figure 9), it is clear that with a low sample size ($n = 200$) and a low number of measurement variables (three per latent variable), there is a large degree of approximation error. Yet both LOESS(BFS) and HZCV(BFS) clearly indicate that a non-linear trend appears needed, and that a quadratic trend

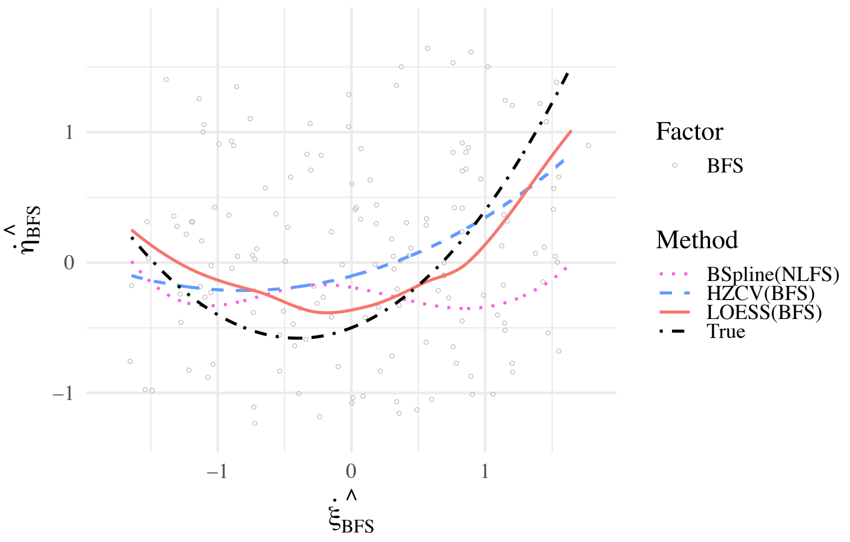


FIGURE 9. *Estimated values* $(\ddot{\eta}_1, \ddot{\xi}_1)$ *using Bartlett factor scores with the true trend H (True) and estimated trends using LOESS based on BFS (LOESS(BFS)), BSpline estimator based on NLFS (BSpline(NLFS)), and the cross-validated HZ-estimator based on BFS (HZCV(BFS)) for* $n = 200$.

appears reasonable. This is less apparent based on BSpline(NLFS), which did not work well in this particular simulation.

The following `R` code uses `lavaan` (Rosseel, 2012) to estimate LOESS(BFS) for this two factor model, where $\xi_1$ influences $\eta_1$, all measured by three observations as represented by Figure 7. As per default in `lavaan`, the latent mean per latent variable is fixed to zero, we manually overwrite this by fixing the first manifest mean per latent variable to zero and freely estimating the latent means. This ensures that the BFS are allowed to have means which is necessary for the nonparametric trend to converge towards the population trend and not a linear combination thereof. The code to estimate all other trends as well as the code resulting in the figures and the data of this section are given in the online supplementary materials.

```
# fit model
model <-   "
# measurement model formulation
Xi1  =~ 1*x1 + x2 + x3
Eta1 =~ 1*y1 + y2 + y3
Xi1  ~~ Eta1

# fix first intercept per latent to zero for scaling
x1 ~0
y1 ~0

# estimate latent means freely
```

```
Xi1  ~1
Eta1 ~1
"
fit <- lavaan::sem(model, data)
BFS <- as.data.frame(lavaan::lavPredict(fit, method = "Bartlett"))

# fit LOESS(BFS)
fitLOESS <- loess(Eta1 ~ Xi1, data = BFS)
LOESS_BFS <- predict(fitLOESS)

# plot data
df <- data.frame(LOESS_BFS, BFS)
library(ggplot2)
ggplot(data = df, mapping = aes(x = Xi1, y = LOESS_BFS)) +
     geom_line()+
     geom_point(mapping = aes(x = Xi1, y = Eta1))
```

## Appendix B. Non-parametric regression among factor scores for a full SEM: a component-wise approach

For a given SEM, the non-parametric estimation methodology developed in this paper can be used to produce component-wise estimates of the influences onto each endogenous variable in the model. This can be achieved by taking each endogenous component of the model, and estimating non-parametrically its regression function using all variables that influence it as explanatory variables. Since this may include variables that are endogenous in the full system, the explanatory variables of each step in the component-wise estimates may be a mixture of both exogenous and endogenous variables.

In this section, we consider this procedure via illustrations following the SEM given in Figure 10. We will illustrate the differences and similarities between considering the reduced form of the SEM and a component-wise perspective through some example calculations.

In this example model we have one exogenous variable $\xi = \xi_1$ and three endogenous variables $\eta = (\eta_1, \eta_2, \eta_3)'$ in the full system. The reduced form representation of the whole system is the conditional expectation of all endogenous variables given the exogenous variable $\xi_1$ distorted by noise $\zeta = (\zeta_1, \zeta_2, \zeta_3)'$

$$\eta = (\eta_1, \eta_2, \eta_3)' = H(\xi_1) + \zeta, \quad \mathbb{E}[\zeta|\xi_1] = 0,$$

where $H : \mathbb{R} \mapsto \mathbb{R}^3$ is $H(x) = \mathbb{E}[\eta|\xi_1 = x]$. This reduced form representation considers how $\xi = \xi_1$ influences $\eta$.

In contrast, we may use the structural model from Figure 10. By the existence of the conditional expectations, we have that there exists functions $\tilde{H}_1, \tilde{H}_2, \tilde{H}_3$ with

$$\begin{aligned}
\eta_1 &= \tilde{H}_1(\xi_1) + \zeta_1, \quad \mathbb{E}[\zeta_1|\xi_1] = 0,\\
\eta_2 &= \tilde{H}_2(\xi_1, \eta_1) + \zeta_2, \quad \mathbb{E}[\zeta_2|\xi_1, \eta_1] = 0,\\
\eta_3 &= \tilde{H}_3(\xi_1, \eta_1, \eta_2) + \zeta_3 \quad \mathbb{E}[\zeta_3|\xi_1, \eta_1, \eta_2] = 0,
\end{aligned}$$

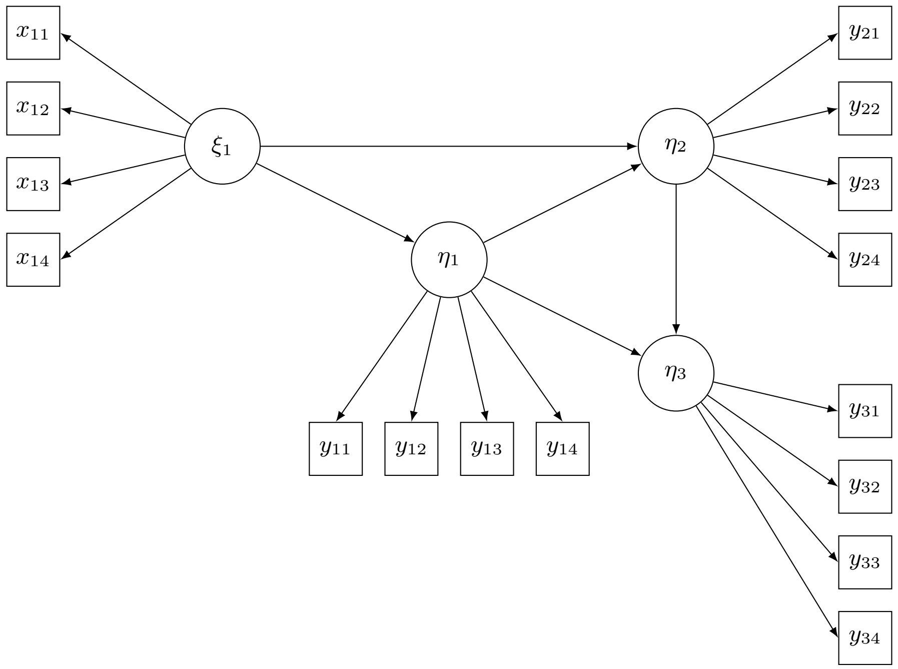


FIGURE 10. An example SEM path diagram, where arrows between latent variables and manifest variables indicate linear relationships, while arrows among latent variable indicate possible nonlinear relations. Residuals and measurement errors are not shown for simplicity, but are present in the model.

so that

$$\begin{aligned}\tilde{H}_1(x_1) &= \mathbb{E}[\eta_1|\xi_1 = x_1] \\ \tilde{H}_2(x_1, y_1) &= \mathbb{E}[\eta_2|\xi_1 = x_1, \eta_1 = y_1] \\ \tilde{H}_3(x_1, y_1, y_2) &= \mathbb{E}[\eta_3|\xi_1 = x_1, \eta_1 = y_1, \eta_2 = y_2].\end{aligned}$$

If we assume that all drawn errors in the path diagram indicate dependence, and missing errors denote independence among variables, the conditional expectation of $\eta_3$ further simplifies to $\mathbb{E}[\eta_3|\eta_1 = y_1, \eta_2 = y_2]$.

In general, the coordinate functions of $H$ will not coincide with $\tilde{H}_1, \tilde{H}_2, \tilde{H}_3$, both because these functions depend on other inputs than $\xi_1$, but also because the reduced form equation including $H_2$ does not take into account for example the influence from $\eta_1$ to $\eta_2$, which is accounted for in $\tilde{H}_2$.

In NLSEM, traditional estimators make stronger assumptions on the error terms $\zeta_j$ than the conditional zero expectation property stated in the above display. Also independence to the variables influencing each coordinate of $\eta$ as well as other error terms are explicitly made (see, e.g., Holst & Budtz-Jørgensen, 2020; Lee et al., 2007; Mooijaart & Bentler, 2010; Mooijaart & Satorra, 2012; Wall & Amemiya, 2000, 2001, 2003), or implicitly made via distributional assumption, such as multivariate normality (see, e.g., Brandt et al., 2018; Kelava & Brandt, 2009; Kenny & Judd, 1984; Klein & Moosbrugger, 2000; Marsh et al., 2004). In this section we will assume that the regression errors $\zeta_1, \zeta_2, \zeta_3$

are independent to what is conditioned on for ease of computation. The rational of our paper is general (see also discussion on non-additive errors in Appendix J). Since these independence assumptions imply that the above stated conditional expectations are zero, we may non-parametrically estimate $\tilde{H}_1, \tilde{H}_2, \tilde{H}_3$ using the techniques of the present paper.

Concretely, to estimate $\tilde{H}_1$, we input $\xi_1$ as the explanatory variable for $\eta_1$. To estimate $\tilde{H}_2$, we input $\xi_1$ and $\eta_1$ as explanatory variables for $\eta_2$. To estimate $\tilde{H}_3$, we input $\xi_1, \eta_1$, and $\eta_2$ as explanatory variables for $\eta_3$.

In each step, the assumptions of the paper have to be fulfilled. In most cases, this is the case if it holds globally. In a few cases, we may loose identification of the covariance parameters when considering a measurement model for a reduced equation set. We do not consider this topic systematically here.

For simplicity, the structural part of Figure 10 is recursive, hence, there are no loops and no correlated error terms. Loops are unproblematic to take into account when considering the model component-wise: Say there would be an arrow also from $\eta_3$ to $\eta_2$. Then the equation for $\eta_2$ would need to include $\eta_3$, giving

$$\eta_2 = H_2(\xi_1, \eta_1, \eta_3) + \zeta_2, \quad \mathbb{E}[\zeta_2|\xi_1, \eta_1, \eta_3] = 0.$$

As for correlated errors in the structural part, we first recall why error terms defined through conditional expectation requirements are uncorrelated with what is conditioned on. That is, recall that $\zeta_1, \zeta_2, \zeta_3$ are defined by tautology through

$$\begin{aligned}
\zeta_1 &= \eta_1 - \mathbb{E}[\eta_1|\xi_1] \\
\zeta_2 &= \eta_2 - \mathbb{E}[\eta_2|\xi_1, \eta_1] \\
\zeta_3 &= \eta_3 - \mathbb{E}[\eta_3|\xi_1, \eta_1, \eta_2].
\end{aligned}$$

Now firstly, we recall that e.g. $\mathbb{E}\zeta_3 = \mathbb{E}[\mathbb{E}[\zeta_3|\xi_1, \eta_1, \eta_2]] = \mathbb{E}0 = 0$, and similarly $\mathbb{E}\zeta_j = 0$ for $j = 1, 2$. Since the error terms have zero mean, we get e.g. that

$$\text{Cov}\,(\zeta_3, \eta_1) = \mathbb{E}\zeta_3\eta_1 = \mathbb{E}[\mathbb{E}[\zeta_3\eta_1|\xi_1, \xi_2, \eta_1, \eta_2]]$$

where here $\eta_1$ is conditioned on, and can therefore be taken outside the inner expectation, giving

$$\text{Cov}\,(\zeta_3, \eta_1) = \mathbb{E}[\eta_1\mathbb{E}[\zeta_3|\xi_1, \eta_1, \eta_2]] = 0.$$

Similarly, all error terms are uncorrelated with the explanatory variables within each equation.

The definition of terms in $\zeta = (\zeta_1, \zeta_2, \zeta_3)'$ does not imply that they are independent nor uncorrelated. Consider the data generating mechanism to imply a correlation among $\zeta$ and assume that the assumptions of the error terms hold. This then implies that the conditional expectation of the error terms when conditioning on the same variables as when defining $\zeta_1, \zeta_2, \zeta_3$, is equivalent to the error terms $\zeta_1, \zeta_2, \zeta_3$ because the conditional expectation is almost surely unique. Therefore, since it is possible to have data generating mechanisms where the error terms in the structural part have correlation, also the error terms $\zeta_1, \zeta_2, \zeta_3$ may be correlated. Therefore, the possibility of correlated errors is embedded within the framework we work with, and cannot be specified to be the case nor chosen away, as we are simply estimating a conditional expectation and its implied residue $\zeta_1, \zeta_2, \zeta_3$.

Consequently, residual covariation among endogenous variables can be estimated using estimates for the residual $\zeta$ by applying its formula. We note the possible influence of approximation error and do not consider this topic systematically here.

We now consider a series of examples, first under model conditions, and then in the upcoming sub-section under structural misspecification. In Section F, we consider similar issues, though under measurement misspecification or non-linear measurement models.

**Example 1.** Consider the linear SEM resulting from the model in Figure 10 by setting all relations among latent variables as linear. Hence, we get

$$\begin{aligned}\eta_1 &= \alpha_1 + \gamma_{1,1,1}\xi_1 + \zeta_1,\\ \eta_2 &= \alpha_2 + \gamma_{1,2,1}\xi_1 + \beta_{1,2,1}\eta_1 + \zeta_2\\ \eta_3 &= \alpha_3 + \beta_{1,3,1}\eta_1 + \beta_{1,3,2}\eta_2 + \zeta_3,\end{aligned}$$

where we use $\gamma$ for the effects of $\xi$ to $\eta$ and $\beta$ for the effects among $\eta$. Further, the first index in $\gamma$ and $\beta$ refers to the order of the effect. Here, only linear effects are present, hence, $\beta_{1,3,1}$ refers to the linear effect of $\eta_1$ to $\eta_3$.

Further, assume that the errors $\zeta_1, \zeta_2, \zeta_3$ have zero means, variances $\mathbb{E}\zeta_1^2 = \psi_{11}, \mathbb{E}\zeta_2^2 = \psi_{22}, \mathbb{E}\zeta_3^2 = \psi_{33}$, and are mutually independent to all other error terms and $\xi_1$. This implies that the error terms are also independent to the explanatory variables used in the equation where the error term is written. To see this, notice that the endogenous variables can sequentially be written in terms of $\xi_1$ and other error terms (first insert the equation for $\eta_1$ into the equation for $\eta_2$, then the equation for $\eta_2$ into the equation for $\eta_3$).

Let us calculate $H$ (with components $H_j, j = 1, 2, 3$) and $\tilde{H}_1, \tilde{H}_2, \tilde{H}_3$. We have $H_1(x_1) = \tilde{H}_1(x_1)$, because this equation does not depend on any of the endogenous variables. Since $\mathbb{E}[\eta_1|\xi_1] = \alpha_1 + \gamma_{1,1,1}\xi_1 + \mathbb{E}[\zeta_1|\xi_1] = \alpha_1 + \gamma_{1,1,1}\xi_1 + \mathbb{E}[\zeta_1] = \alpha_1 + \gamma_{1,1,1}\xi_1$ by the assumed independence properties for $\zeta_1$ and it being a residual with zero mean. Therefore, $H_1(x_1) = \tilde{H}_1(x_1) = \alpha_1 + \gamma_{1,1,1}x_1$.

For $\eta_2$, we have

$$\begin{aligned}\mathbb{E}[\eta_2|\xi_1] &= \mathbb{E}[\alpha_2 + \gamma_{1,2,1}\xi_1 + \beta_{1,2,1}\eta_1 + \zeta_2|\xi_1]\\ &= \alpha_2 + \gamma_{1,2,1}\xi_1 + \beta_{1,2,1}\mathbb{E}[\eta_1|\xi_1] + \mathbb{E}[\zeta_2|\xi_1].\end{aligned}$$

Now, we have $\mathbb{E}\left[\eta_1|\xi_1\right] = \alpha_1 + \gamma_{1,1,1}\xi_1$, and $\mathbb{E}\left[\zeta_2|\xi_1\right] = \mathbb{E}\left[\zeta_2\right] = 0$. Therefore,

$$\mathbb{E}[\eta_2|\xi_1] = \alpha_2 + \beta_{1,2,1}\alpha_1 + (\gamma_{1,2,1} + \beta_{1,2,1}\gamma_{1,1,1})\xi_1,$$

and, hence, $H_2(x_1) = \alpha_2 + \beta_{1,2,1}\alpha_1 + (\gamma_{1,2,1} + \beta_{1,2,1}\gamma_{1,1,1})x_1$. For $\tilde{H}_2$ we use Lemma 7 (p. A75) and get

$$\mathbb{E}[\eta_2|\xi_1, \eta_1] = \alpha_2 + \gamma_{1,2,1}\xi_1 + \beta_{1,2,1}\eta_1,$$

and, hence, $\tilde{H}_2(x_1, y_1) = \alpha_2 + \gamma_{1,2,1}x_1 + \beta_{1,2,1}y_1$.

Finally, for the reduced form relationship between $\xi_1$ and $\eta_3$, we have

$$\mathbb{E}[\eta_3|\xi_1] = \alpha_3 + \beta_{1,3,1}\mathbb{E}[\eta_1|\xi_1] + \beta_{1,3,2}\mathbb{E}[\eta_2|\xi_1] + \mathbb{E}[\zeta_3|\xi_1],$$

for which we have $\mathbb{E}[\zeta_3|\xi_1] = 0$, and, hence,

$$\mathbb{E}[\eta_3|\xi_1] = \alpha_3 + \beta_{1,3,1}\alpha_1 + \beta_{1,3,2}\alpha_2 + \beta_{1,3,2}\beta_{1,2,1}\alpha_1 + (\beta_{1,3,1}\gamma_{1,1,1} + +\beta_{1,3,2}\gamma_{1,2,1} + \beta_{1,3,2}\beta_{1,2,1}\gamma_{1,1,1})\xi_1.$$

Therefore, $H_3(x_1) = \alpha_3+\beta_{1,3,1}\alpha_1+\beta_{1,3,2}\alpha_2+\beta_{1,3,2}\beta_{1,2,1}\alpha_1+(\beta_{1,3,1}\gamma_{1,1,1}++\beta_{1,3,2}\gamma_{1,2,1}+\beta_{1,3,2}\beta_{1,2,1}\gamma_{1,1,1})x_1$. For $\tilde{H}_3$, we again use Lemma 7 (p. A75) and get

$$\mathbb{E}[\eta_3|\xi_1, \eta_1, \eta_2] = \alpha_3 + \beta_{1,3,1}\eta_1 + \beta_{1,3,2}\eta_2,$$

and, hence, $\tilde{H}_3(x_1, y_1, y_2) = \alpha_3 + \beta_{1,3,1}y_1 + \beta_{1,3,2}y_2$.

To summarize, we have that

$$\begin{aligned}
H_1(x_1) &= \alpha_1 + \gamma_{1,1,1}x_1,\\
H_2(x_1) &= \underbrace{\alpha_2 + \beta_{1,2,1}\alpha_1}_{\alpha_2^\star} + \underbrace{(\gamma_{1,1,1}\beta_{1,2,1} + \gamma_{1,2,1})}_{\gamma_{1,2,1}^\star} x_1,\\
H_3(x_1) &= \underbrace{\alpha_3 + \beta_{1,3,1}\alpha_1 + \beta_{1,3,2}\alpha_2 + \beta_{1,3,2}\beta_{1,2,1}\alpha_1}_{\alpha_3^\star} + \underbrace{(\gamma_{1,2,1}\beta_{1,3,2} + \gamma_{1,1,1}\beta_{1,2,1}\beta_{1,3,2} + \gamma_{1,1,1}\beta_{1,3,2})}_{\gamma_{1,3,1}^\star} x_1,
\end{aligned}$$

and, in contrast, for $\tilde{H}_j(j = 1, 2, 3)$ we get

$$\begin{aligned}
\tilde{H}_1(x_1) &= \alpha_1 + \gamma_{1,1,1}x_1,\\
\tilde{H}_2(x_1, y_1) &= \alpha_2 + \gamma_{1,2,1}x_1 + \beta_{1,2,1}y_1,\\
\tilde{H}_3(x_1, y_1, y_2) &= \alpha_3 + \beta_{1,3,1}y_1 + \beta_{1,3,2}y_2.
\end{aligned}$$

Translating this into mediation analysis framework (see for an overview, MacKinnon et al., 2007), $H_2$, for instance, refers to the total effect of $\xi_1$ onto $\eta_2$, while $\tilde{H}_2$ describes the effect of $\xi_1$ to $\eta_2$ above and beyond $\eta_1$ in a regression sense. Hence, $\gamma_{1,2,1}$ within $\tilde{H}_2$ is the unique linear relation between $\xi_1$ and $\eta_2$ above and beyond $\eta_1$, while $\gamma_{1,2,1}^\star$ is the total effect of $\xi_1$ to $\eta_2$, ignoring any relations *mediated* by $\eta_1$. □

**Example 2.** Consider the nonlinear SEM

$$\begin{aligned}
\eta_1 &= \alpha_1 + \gamma_{1,1,1}\xi_1 + \zeta_1,\\
\eta_2 &= \alpha_2 + \gamma_{1,2,1}\xi_1 + \beta_{1,2,1}\eta_1 + \beta_{2,2,1}\eta_1^2 + \zeta_2\\
\eta_3 &= \alpha_3 + \beta_{1,3,1}\eta_1 + \beta_{1,3,2}\eta_2 + \beta_{2,3,1}\eta_1^2 + \beta_{3,3,1}\eta_1^3 + \zeta_3,
\end{aligned}$$

as a nonlinear extension of the linear SEM of Example 1, again representing the (possible nonlinear) relations depicted in Figure 10. We use the same notation as in Example 1, i.e., $\beta_{3,3,1}$ is the effect of the cubic $\eta_1^3$ on $\eta_3$, and, again, assume that the errors $\zeta_1, \zeta_2, \zeta_3$ have zero means, variances $\mathbb{E}\zeta_1^2 = \psi_{11}, \mathbb{E}\zeta_2^2 = \psi_{22}, \mathbb{E}\zeta_3^2 = \psi_{33}$, and are mutually independent to all other error terms and $\xi_1$.

Let us (again) calculate $H$ (with components $H_j, j = 1, 2, 3$) and $\tilde{H}_1, \tilde{H}_2, \tilde{H}_3$.

Identically to Example 1, we have $H_1(x_1) = \tilde{H}_1(x_1) = \alpha_1 + \gamma_{1,1,1}x_1$.

For $\eta_2$, we have

$$\begin{aligned}
\mathbb{E}[\eta_2|\xi_1] &= \mathbb{E}[\alpha_2 + \gamma_{1,2,1}\xi_1 + \beta_{1,2,1}\eta_1 + \beta_{2,2,1}\eta_1^2 + \zeta_2|\xi_1]\\
&= \alpha_2 + \gamma_{1,2,1}\xi_1 + \beta_{1,2,1}\mathbb{E}[\eta_1|\xi_1] + \beta_{2,2,1}\mathbb{E}[\eta_1^2|\xi_1] + \mathbb{E}[\zeta_2|\xi_1].
\end{aligned}$$

Now, we have $\mathbb{E}[\eta_1|\xi_1] = \alpha_1 + \gamma_{1,1,1}\xi_1$, and $\mathbb{E}[\zeta_2|\xi_1] = \mathbb{E}[\zeta_2] = 0$. For the expectation of $\eta_1^2$ conditioned on $\xi_1$ we get

$$\begin{aligned}
\mathbb{E}\left[\eta_1^2|\xi_1\right] &= \mathbb{E}\left[(\alpha_1 + \gamma_{1,1,1}\xi_1 + \zeta_1)^2|\xi_1\right]\\
&= \mathbb{E}\left[\alpha_1^2 + \gamma_{1,1,1}^2\xi_1^2 + \zeta_1^2 + 2\alpha_1\gamma_{1,1,1}\xi_1 + 2\alpha_1\zeta_1 + 2\gamma_{1,1,1}\xi_1\zeta_1|\xi_1\right]\\
&= \alpha_1^2 + \gamma_{1,1,1}^2\mathbb{E}\left[\xi_1^2|\xi_1\right] + \mathbb{E}\left[\zeta_1^2|\xi_1\right] + 2\alpha_1\gamma_{1,1,1}\mathbb{E}\left[\xi_1|\xi_1\right] + 2\alpha_1\mathbb{E}\left[\zeta_1|\xi_1\right] + 2\gamma_{1,1,1}\mathbb{E}\left[\xi_1\zeta_1|\xi_1\right]\\
&= \alpha_1^2 + \gamma_{1,1,1}^2\xi_1^2 + \mathbb{E}\left[\zeta_1^2\right] + 2\alpha_1\gamma_{1,1,1}\xi_1 + 2\alpha_1\cdot 0 + 2\gamma_{1,1,1}\xi_1\mathbb{E}\left[\zeta_1|\xi_1\right]\\
&= \alpha_1^2 + \gamma_{1,1,1}^2\xi_1^2 + \psi_{11} + 2\alpha_1\gamma_{1,1,1}\xi_1 + 2\gamma_{1,1,1}\xi_1\cdot 0\\
&= \alpha_1^2 + \psi_{11} + 2\alpha_1\gamma_{1,1,1}\xi_1 + \gamma_{1,1,1}^2\xi_1^2.
\end{aligned}$$

Hence, for $\mathbb{E}[\eta_2|\xi_1]$ we have

$$\begin{aligned}
\mathbb{E}[\eta_2|\xi_1] &= \alpha_2 + \gamma_{1,2,1}\xi_1 + \beta_{1,2,1}\left(\alpha_1 + \gamma_{1,1,1}\xi_1\right) + \beta_{2,2,1}\left(\alpha_1^2 + \psi_{11} + 2\alpha_1\gamma_{1,1,1}\xi_1 + \gamma_{1,1,1}^2\xi_1^2\right) \\
&= \alpha_2 + \beta_{1,2,1}\alpha_1 + \gamma_{1,2,1}\xi_1 + \beta_{1,2,1}\gamma_{1,1,1}\xi_1 + \beta_{2,2,1}\alpha_1^2 + \beta_{2,2,1}\psi_{11} + \beta_{2,2,1}2\alpha_1\gamma_{1,1,1}\xi_1 + \beta_{2,2,1}\gamma_{1,1,1}^2\xi_1^2 \\
&= \alpha_2 + \beta_{1,2,1}\alpha_1 + \beta_{2,2,1}\psi_{11} + \beta_{2,2,1}\alpha_1^2 + \gamma_{1,2,1}\xi_1 + \beta_{1,2,1}\gamma_{1,1,1}\xi_1 + 2\beta_{2,2,1}\alpha_1\gamma_{1,1,1}\xi_1 + \beta_{2,2,1}\gamma_{1,1,1}^2\xi_1^2 \\
&= \underbrace{\alpha_2 + \beta_{1,2,1}\alpha_1 + \beta_{2,2,1}\psi_{11} + \beta_{2,2,1}\alpha_1^2}_{=\alpha_2^\star} + \underbrace{\left(\gamma_{1,2,1} + \beta_{1,2,1}\gamma_{1,1,1} + 2\beta_{2,2,1}\alpha_1\gamma_{1,1,1}\right)}_{=\gamma_{1,2,1}^\star}\xi_1 + \underbrace{\beta_{2,2,1}\gamma_{1,1,1}^2}_{=\gamma_{2,2,1}^\star}\xi_1^2,
\end{aligned}$$

which is a quadratic form in $\xi_1$. We get $H_2(x_1) = \alpha_2^\star + \gamma_{1,2,1}^\star x_1 + \gamma_{2,2,1}^\star x_1^2$. In contrast, when conditioning on $\xi_1$ and $\eta_1$, we get

$$\mathbb{E}[\eta_2|\xi_1, \eta_1] = \alpha_2 + \gamma_{1,2,1}\xi_1 + \beta_{1,2,1}\eta_1 + \beta_{2,2,1}\eta_1^2,$$

so that $\tilde{H}_2(x_1, y_1) = \alpha_2 + \gamma_{1,2,1}x_1 + \beta_{1,2,1}y_1 + \beta_{2,2,1}y_1^2$.

Finally, for the reduced form relationship between $\xi_1$ and $\eta_3$, we have

$$\mathbb{E}[\eta_3|\xi_1] = \alpha_3 + \beta_{1,3,1}\mathbb{E}[\eta_1|\xi_1] + \beta_{1,3,2}\mathbb{E}[\eta_2|\xi_1] + \beta_{2,3,1}\mathbb{E}\left[\eta_1^2|\xi_1\right] + \beta_{3,3,1}\mathbb{E}\left[\eta_1^3|\xi_1\right] + \mathbb{E}[\zeta_3|\xi_1],$$

for which we have already derived $\mathbb{E}[\eta_1|\xi_1], \mathbb{E}[\eta_1^2|\xi_1]$, $\mathbb{E}[\eta_2|\xi_1]$, and have that $\mathbb{E}[\zeta_3|\xi_1] = \mathbb{E}[\zeta_3] = 0$ due to the independence of $\zeta_3$ to all other variables. Hence, we only have to calculate $\mathbb{E}[\eta_1^3|\xi_1]$:

$$\begin{aligned}
\mathbb{E}[\eta_1^3|\xi] &= \mathbb{E}\left[(\alpha_1 + \gamma_{1,1,1}\xi_1 + \zeta_1)^3|\xi_1\right] \\
&= \mathbb{E}\big[\alpha_1^3 + 3\alpha_1^2\gamma_{1,1,1}\xi_1 + 3\alpha_1^2\zeta_1 + 3\alpha_1(\gamma_{1,1,1}\xi_1)^2 + 6\alpha_1\gamma_{1,1,1}\xi_1\zeta_1 + 3\alpha_1\zeta_1^2 + \\
&\quad (\gamma_{1,1,1}\xi_1)^3 + 3(\gamma_{1,1,1}\xi_1)^2\zeta_1 + 3\gamma_{1,1,1}\xi_1\zeta_1^2 + \zeta_1^3|\xi_1\big] \\
&= \alpha_1^3 + 3\alpha_1^2\gamma_{1,1,1}\mathbb{E}\left[\xi_1|\xi_1\right] + 3\alpha_1^2\mathbb{E}\left[\zeta_1|\xi_1\right] + 3\alpha_1\gamma_{1,1,1}^2\mathbb{E}\left[\xi_1^2|\xi_1\right] + 6\alpha_1\gamma_{1,1,1}\mathbb{E}\left[\xi_1\zeta_1|\xi_1\right] + 3\alpha_1\mathbb{E}\left[\zeta_1^2|\xi_1\right] + \\
&\quad \gamma_{1,1,1}^3\mathbb{E}\left[\xi_1^3|\xi_1\right] + 3\gamma_{1,1,1}^2\mathbb{E}\left[\xi_1^2\zeta_1|\xi_1\right] + 3\gamma_{1,1,1}\mathbb{E}\left[\xi_1\zeta_1^2|\xi_1\right] + \mathbb{E}\left[\zeta_1^3|\xi_1\right] \\
&= \alpha_1^3 + 3\alpha_1^2\gamma_{1,1,1}\xi_1 + 3\alpha_1^2\cdot 0 + 3\alpha_1\gamma_{1,1,1}^2\xi_1^2 + 6\alpha_1\gamma_{1,1,1}\xi_1\mathbb{E}\left[\zeta_1|\xi_1\right] + 3\alpha_1\mathbb{E}\left[\zeta_1^2\right] + \\
&\quad \gamma_{1,1,1}^3\xi_1^3 + 3\gamma_{1,1,1}^2\xi_1^2\mathbb{E}\left[\zeta_1|\xi_1\right] + 3\gamma_{1,1,1}\xi_1\mathbb{E}\left[\zeta_1^2|\xi_1\right] + \mathbb{E}\left[\zeta_1^3\right] \\
&= \alpha_1^3 + 3\alpha_1^2\gamma_{1,1,1}\xi_1 + 3\alpha_1\gamma_{1,1,1}^2\xi_1^2 + 6\alpha_1\gamma_{1,1,1}\xi_1\cdot 0 + 3\alpha_1\psi_{11} + \\
&\quad \gamma_{1,1,1}^3\xi_1^3 + 3\gamma_{1,1,1}^2\xi_1^2\cdot 0 + 3\gamma_{1,1,1}\xi_1\mathbb{E}\left[\zeta_1^2\right] + \mathbb{E}\left[\zeta_1^3\right] \\
&= \alpha_1^3 + 3\alpha_1\psi_{11} + \mathbb{E}\left[\zeta_1^3\right] + \left(3\alpha_1^2\gamma_{1,1,1} \;\; + 3\gamma_{1,1,1}\psi_{11}\right)\xi_1 + \alpha_1\gamma_{1,1,1}^2\xi_1^2 + \gamma_{1,1,1}^3\xi_1^3.
\end{aligned}$$

Hence,

$$
\begin{aligned}
\mathbb{E}[\eta_3|\xi_1] &= \alpha_3 + \beta_{1,3,1}\left(\alpha_1 + \gamma_{1,1,1}\xi_1\right) + \beta_{1,3,2}\left(\alpha_2^\star + \gamma_{1,2,1}^\star\xi_1 + \gamma_{2,2,1}^\star\xi_1^2\right) + \\
&\quad \beta_{2,3,1}\left(\alpha_1^2 + \psi_{11} + 2\alpha_1\gamma_{1,1,1}\xi_1 + \gamma_{1,1,1}^2\xi_1^2\right) + \\
&\quad \beta_{3,3,1}\left(\alpha_1^3 + 3\alpha_1\psi_{11} + \mathbb{E}\left[\zeta_1^3\right] + \left(3\alpha_1^2\gamma_{1,1,1} + 3\gamma_{1,1,1}\psi_{11}\right)\xi_1 + \alpha_1\gamma_{1,1,1}^2\xi_1^2 + \gamma_{1,1,1}^3\xi_1^3\right) \\
&= \alpha_3 + \beta_{1,3,1}\alpha_1 + \beta_{1,3,1}\gamma_{1,1,1}\xi_1 + \beta_{1,3,2}\alpha_2^\star + \beta_{1,3,2}\gamma_{1,2,1}^\star\xi_1 + \beta_{1,3,2}\gamma_{2,2,1}^\star\xi_1^2 + \\
&\quad \beta_{2,3,1}\alpha_1^2 + \beta_{2,3,1}\psi_{11} + 2\beta_{2,3,1}\alpha_1\gamma_{1,1,1}\xi_1 + \beta_{2,3,1}\gamma_{1,1,1}^2\xi_1^2 + \beta_{3,3,1}\alpha_1^3 + 3\beta_{3,3,1}\alpha_1\psi_{11} + \\
&\quad \beta_{3,3,1}\mathbb{E}\left[\zeta_1^3\right] + \beta_{3,3,1}\left(3\alpha_1^2\gamma_{1,1,1} + 3\gamma_{1,1,1}\psi_{11}\right)\xi_1 + \beta_{3,3,1}\alpha_1\gamma_{1,1,1}^2\xi_1^2 + \beta_{3,3,1}\gamma_{1,1,1}^3\xi_1^3 \\
&= \underbrace{\alpha_3 + \beta_{1,3,1}\alpha_1 + \beta_{1,3,2}\alpha_2^\star + \beta_{2,3,1}\alpha_1^2 + \beta_{2,3,1}\psi_{11} + \beta_{3,3,1}\alpha_1^3 + 3\beta_{3,3,1}\alpha_1\psi_{11} + \beta_{3,3,1}\mathbb{E}\left[\zeta_1^3\right]}_{=\alpha_3^\star} + \\
&\quad \underbrace{\left(\beta_{1,3,1}\gamma_{1,1,1} + \beta_{1,3,2}\gamma_{1,2,1}^\star + 2\beta_{2,3,1}\alpha_1\gamma_{1,1,1} + \beta_{3,3,1}\left(3\alpha_1^2\gamma_{1,1,1} + 3\gamma_{1,1,1}\psi_{11}\right)\right)}_{=\gamma_{1,3,1}^\star}\xi_1 + \\
&\quad \underbrace{\left(\beta_{1,3,2}\gamma_{2,2,1}^\star + \beta_{2,3,1}\gamma_{1,1,1}^2 + \beta_{3,3,1}\alpha_1\gamma_{1,1,1}^2\right)}_{=\gamma_{2,3,1}^\star}\xi_1^2 + \underbrace{\beta_{3,3,1}\gamma_{1,1,1}^3}_{=\gamma_{3,3,1}^\star}\xi_1^3,
\end{aligned}
$$

where we get a constant that depends on the skewness of $\zeta_1$, i.e., the third order moment of $\zeta_1$.

Therefore, the reduced form of $\eta_3$ given $\xi_1$ is a third order polynomial in $\xi_1$ with the form

$$H_3(x_1) = \alpha_3^\star + \gamma_{1,3,1}^\star x_1 + \gamma_{2,3,1}^\star x_1^2 + \gamma_{3,3,1}^\star x_1^3.$$

This reduced form third order polynomial stands in direct conflict with the conditional expectation given $\xi_1, \eta_1$, and $\eta_2$, for which we immediately have that

$$\mathbb{E}[\eta_3|\xi_1, \eta_1, \eta_2] = \alpha_3 + \beta_{1,3,1}\eta_1 + \beta_{1,3,2}\eta_2 + \beta_{2,3,1}\eta_1^2 + \beta_{3,3,1}\eta_1^3,$$

which does not depend on the values of $\xi_1$ directly, but only indirectly through the values of $\eta_1$ and $\eta_2$.

Consequently, $\tilde{H}_3(x_1, y_1, y_2) = \alpha_3 + \beta_{1,3,1}y_1 + \beta_{1,3,2}y_2 + \beta_{2,3,1}y_1^2 + \beta_{3,3,1}y_1^3$.

To summarize, we have that

$$
\begin{aligned}
H_1(x_1) &= \alpha_1 + \gamma_{1,1,1}x_1, \\
H_2(x_1) &= \alpha_2^\star + \gamma_{1,2,1}^\star x_1 + \gamma_{2,2,1}^\star x_1^2, \\
H_3(x_1) &= \alpha_3^\star + \gamma_{1,3,1}^\star x_1 + \gamma_{2,3,1}^\star x_1^2 + \gamma_{3,3,1}^\star x_1^3,
\end{aligned}
$$

and, in contrast, for $\tilde{H}_j (j = 1, 2, 3)$ we get

$$
\begin{aligned}
\tilde{H}_1(x_1) &= \alpha_1 + \gamma_{1,1,1}x_1, \\
\tilde{H}_2(x_1, y_1) &= \alpha_2 + \gamma_{1,2,1}x_1 + \beta_{1,2,1}y_1 + \beta_{2,2,1}y_1^2, \\
\tilde{H}_3(x_1, y_1, y_2) &= \alpha_3 + \beta_{1,3,1}y_1 + \beta_{1,3,2}y_2 + \beta_{2,3,1}y_1^2 + \beta_{3,3,1}y_1^3.
\end{aligned}
$$

In conclusion, we emphasize that, for instance, $H_3$ representing the total effect of $\xi_1$ onto $\eta_3$ is a third order polynomial in $\xi_1$, while $\tilde{H}_3$ does not directly depend on $\xi_1$. Further, the total effect of $\xi_1$ onto $\eta_2$ as represented by $H_2$ is a quadratic form in $\xi_1$, while the direct effect of $\xi_1$ onto $\eta_2$ is linear in the full system, denoted by the function $\tilde{H}_2$. The reduced form representation, therefore, does not give any insights on the directness of the effects of any explanatory variables onto the endogenous variables, further, the functional form may vary drastically. □

B.1. **Considerations under Structural Misspecifications.** Standard covariance based goodness of fit tests can consistently (i.e., having power approaching one asymptotically) detect model misspecification if the misspecification is linear and the degrees of freedom is at least one. In the case of non-linear misspecifications, this need not be the case (Mooijaart & Satorra, 2009) when using classical goodness of fit tests (SEM lacking quadratic and interaction terms can be detected using the methods of e.g., Büchner & Klein, 2020; Nestler, 2015). This was also illustrated in the simple simulation example in Section A. An important application class for non-parametric trend estimates is therefore to detect such non-linear structural misspecification when a linear model is considered. We here consider some elementary illustrations of this issue.

**Example 3.** In Mooijaart and Satorra (2009), the three latent variables $\eta_1, \xi_1, \xi_2$ were considered. The data-generating mechanism of the structural part in their notation was

$$\eta_1 = \bar{\beta}_0 + \bar{\beta}_1\xi_1 + \bar{\beta}_2\xi_2 + \bar{\beta}_{12}\xi_1\xi_2 + \zeta,$$

where it was assumed that $\zeta$ was zero mean and independent to $\xi_1, \xi_2$, which means that $\mathbb{E}[\zeta|\xi_1, \xi_2] = 0$. Therefore, this is the same error term as the one generated from the conditional expectation argument, as this is (a.s.) unique.

Since $\mathbb{E}[\eta_1|\xi_1, \xi_2] = \bar{\beta}_0 + \bar{\beta}_1\xi_1 + \bar{\beta}_2\xi_2 + \bar{\beta}_{12}\xi_1\xi_2$. The non-parametric trend estimators would in this case consistently estimate the function

$$H(x_1, x_2) = \bar{\beta}_0 + \bar{\beta}_1 x_1 + \bar{\beta}_2 x_2 + \bar{\beta}_{12} x_1 x_2.$$

Therefore, the misspecification would be (asymptotically) detectable using the non-parametric approach. □

When applying non-parametric trend estimates component-wise to a full SEM, we run the risk of being influenced by structural misspecification. In terms of the non-parametric methods, this would mean that we approximate the conditional expectation of an endogenous variable, but that we condition on the right variables compared to if we had knowledge of the correct structural model. Because these conditional expectation functions always exists, it will be as far as we know impossible with presently available tools to separate model misspecification or functional misspecification, and we believe such separation techniques will require further assumptions than considered in the present paper. A full discussion of the practical implications of this is outside the scope of the present paper. We only consider the following example of this issue.

**Example 4.** The data generating mechanism of the example is

$$\eta_1 = \alpha_1 + \gamma_{1,1,1}\xi_1 + \zeta_1, \tag{7}$$

$$\eta_2 = \alpha_2 + \gamma_{1,2,1}\xi_1 + \beta_{1,2,1}\eta_1 + \beta_{2,2,1}\eta_1^2 + \zeta_2 \tag{8}$$

where the error terms $\zeta_1, \zeta_2$ have zero mean, $\mathbb{E}\zeta_1^2 = \psi_{11}, \mathbb{E}\zeta_2^2 = \psi_{22}$, and are independent to each other and to $\xi_1$.

Suppose now that we use a model that is incorrect, and omits the connection from $\eta_1$ to $\eta_2$. In the model, we would therefore suppose

$$\eta_1 = \alpha_1 + \gamma_{1,1,1}\xi_1 + \zeta_1$$

$$\eta_2 = \tilde{\alpha}_2 + \tilde{\gamma}_{1,2,1}\xi_1 + \tilde{\zeta}_2.$$

In the misspecified model, $\eta_2$ is linear in $\xi_1$, and lacks not only a linear influence from $\eta_1$ but also the quadratic influence from $\eta_1$. The $\sim$ indicates that these parameters will not, in general, be the true parameters of the original system and $\tilde{\zeta}_2$ is not the correct residual.

When non-parametrically estimating the structural specification of this system using the component-wise approach, we would first study the first equation, which here is correctly specified. Then the next step would consider $\mathbb{E}[\eta_2|\xi_1]$. If we had knowledge of the correct structural model, we would instead have considered $\mathbb{E}[\eta_2|\xi_1, \eta_1]$. But because of the misspecification, we do not condition on $\eta_1$. We would instead approximate $\mathbb{E}[\eta_2|\xi_1]$. We now calculate this conditional expectation.

This calculation is identical to earlier calculations in Example 2, and we get

$$\mathbb{E}[\eta_2|\xi_1] = \underbrace{\alpha_2 + \beta_{1,2,1}\alpha_1 + \beta_{2,2,1}\psi_{11} + \beta_{2,2,1}\alpha_1^2}_{=\alpha_2^\star} + \underbrace{(\gamma_{1,2,1} + \beta_{1,2,1}\gamma_{1,1,1} + 2\beta_{2,2,1}\alpha_1\gamma_{1,1,1})}_{=\gamma_{1,2,1}^\star}\xi_1 + \underbrace{\beta_{2,2,1}\gamma_{1,1,1}^2}_{=\gamma_{2,2,1}^\star}\xi_1^2,$$

which is a quadratic in $\xi_1$ instead of the linear function which would be expected if the structural model was correctly specified.

Based on non-parametric estimates of $\mathbb{E}[\eta_2|\xi_1 = x]$, the psychometrician would therefore know that there was a model misspecification, and that this model specification induced a square term in this conditional expectation. With substantive knowledge, this might lead the psychometrician to identify the correct model.

**Example 5.** Let us continue the previous example. Suppose now that the psychometrician does update the model, but that based on plots of approximations of $\mathbb{E}[\eta_2|\xi_1 = x]$ the update does not reach the correct model, but instead the model

$$\begin{aligned} \eta_1 &= \alpha_1 + \gamma_{1,1,1}\xi_1 + \zeta_1 \\ \eta_2 &= \alpha_2^\star + \gamma_{1,2,1}^\star\xi_1 + \gamma_{2,2,1}^\star\xi_1^2 + \zeta_2^\star. \end{aligned}$$

This model is still misspecified, but the detection of this misspecification is a more subtle issue, as the equation for $\eta_2$ is now compatible with the trend observed in approximations to $\mathbb{E}[\eta_2|\xi_1 = x]$

While equations of the updated model are similar to the trend in the data generating mechanism, they are different, as the psychometrician has not included the direct effect from $\eta_1$ to $\eta_2$. Let us consider this difference a bit closer: Recall that the equation system that generates the data is given in eq. (7) and (8). In these equations, we insert the expression from $\eta_1$ into $\eta_2$, which gives

$$\begin{aligned} \eta_1 &= \alpha_1 + \gamma_{1,1,1}\xi_1 + \zeta_1, \\ \eta_2 &= \alpha_2 + \beta_{1,2,1}\alpha_1 + \gamma_{1,2,1}\xi_1 + \beta_{1,2,1}\gamma_{1,1,1}\xi_1 + \beta_{1,2,1}\zeta_1 + \\ &\qquad \beta_{2,2,1}(\alpha_1^2 + \gamma_{1,1,1}^2\xi_1^2 + \zeta_1^2 + 2\alpha_1\gamma_{1,1,1}\xi_1 + 2\alpha_1\zeta_1 + 2\gamma_{1,1,1}\xi_1\zeta_1) + \zeta_2 \end{aligned}$$

We see that the updated model is in fact the reduced form equations. From this equation we also deduce that

$$\begin{aligned} \alpha_2^\star &= \alpha_2 + \beta_{1,2,1}\alpha_1 + \beta_{2,2,1}\alpha_1^2, \\ \gamma_{1,2,1}^\star &= \gamma_{1,2,1} + \beta_{1,2,1}\gamma_{1,1,1} + \beta_{2,2,1}2\alpha_1\gamma_{1,1,1}, \\ \gamma_{2,2,1}^\star &= \beta_{2,2,1}\gamma_{1,1,1}^2, \\ \zeta_2^\star &= \eta_2 - \mathbb{E}[\eta_2|\xi_1] = \beta_{2,2,1}(\zeta_1^2 - \psi_{11}) + (\beta_{1,2,1} + 2\beta_{2,2,1}\alpha_1 + 2\beta_{2,2,1}\gamma_{1,1,1}\xi_1)\zeta_1 + \zeta_2. \end{aligned}$$

We notice that $\zeta_2^\star$ is not equal to $\zeta_2$ in general, and is substantially different from $\zeta_2$. For example, $\zeta_2^\star$ includes the term $2\beta_{2,2,1}\gamma_{1,1,1}\xi_1\zeta_1$ which induces a heteroskedasticity into the error, and $\zeta_2^\star$ also includes a linear contribution from $\zeta_1$.

We started out with independent error terms $\zeta_1, \zeta_2$, and in the reduced form expression we have

$$\begin{aligned}
\mathrm{Cov}(\zeta_1, \zeta_2^\star) &= \mathbb{E}\zeta_1\zeta_2^\star \\
&= \mathbb{E}\left[\zeta_1\left(\beta_{2,2,1}(\zeta_1^2 - \psi_{11}) + (\beta_{1,2,1} + 2\beta_{2,2,1}\alpha_1 + 2\beta_{2,2,1}\gamma_{1,1,1}\xi_1)\zeta_1 + \zeta_2\right)\right] \\
&= \mathbb{E}\beta_{2,2,1}(\zeta_1^3 - \psi_{11}\zeta_1) + (\beta_{1,2,1} + 2\beta_{2,2,1}\alpha_1 + 2\beta_{2,2,1}\gamma_{1,1,1}\mathbb{E}\xi_1)\mathbb{E}\zeta_1^2 + \mathbb{E}\zeta_1\zeta_2 \\
&= \mathbb{E}\beta_{2,2,1}\zeta_1^3 + (\beta_{1,2,1} + 2\beta_{2,2,1}\alpha_1 + 2\beta_{2,2,1}\gamma_{1,1,1}\mathbb{E}\xi_1)\psi_{11},
\end{aligned}$$

which is non-zero under most parameter configurations.

If independence between the error terms in the structural part of the model is considered part of the model, the correlation of the error terms $\zeta_1, \zeta_2^\star$ can be seen as an identifiable indication that the model is misspecified.

## Appendix C. A Literature review of NLSEM

Early contributions to nonlinear factor analysis are Gibson (1959), R. McDonald (1967) and Etezadi-Amoli and McDonald (1983), who focused on examining nonlinear relationships between measurements and latent variables. This literature formed the theoretical background for NLSEM, which started fully with Kenny and Judd (1984), who suggested a normal theory product indicator approach for interaction models. This approach was extended and enhanced by relaxing certain constraints on the latent structure in Kelava and Brandt (2009); Marsh et al. (2004); Wall and Amemiya (2001).

What may be termed distribution analytic approaches have been proposed, assuming multivariate normality of both the latent exogenous variables and residuals (LMS, Klein and Moosbrugger, 2000, QML, Klein and Muthén, 2007). To account for non-normal latent exogenous variables, the LMS approach has been extended using latent classes (Kelava, Nagengast, & Brandt, 2014). In applied research, simplified versions of LMS rely on a single indicator per latent variable was suggested (Cheung & Lau, 2017).

Product indicator approaches traditionally rely on the first two moments of (mixed) polynomials of the measurements. Mooijaart and Bentler (2010) extended this to third-order moments. Mooijaart and Satorra (2012) further extended this approach to test the significance of certain moments in interaction models.

Several Bayesian approaches have been proposed: Arminger and Muthén (1998), Lee et al. (2007), and Kelava and Nagengast (2012) have all introduced Bayesian methods in this context. The approach of Lee et al. (2007) can be viewed as a Bayesian counterpart to LMS, while the one of Kelava and Nagengast (2012) can be seen as a Bayesian version of Kelava et al. (2014). Additionally, a Bayesian lasso approach for NLSEM, designed to handle multicollinear latent exogenous variables, has been put forth by Brandt et al. (2018).

Semi-parametric Bayesian models have been suggested: A semi-parametric Bayesian framework with non-parametric estimates of measurement error distributions was suggested in Song et al. (2010). A Bayesian lasso-type framework for basis function expansions of the influence from $\xi$ to $\eta$ was suggested in Guo, Zhu, Chow, and Ibrahim (2012), which was expanded by employing a grouped lasso approach that enables model selection (Feng, Wang, Wang, & Song, 2015). Additionally, Song, Lu, Cai, and Ip (2013) proposed a penalized spline approach that extends a previously suggested spline method (Song & Lu, 2010) by incorporating penalties and by modeling continuous, dichotomous, and count data. It should be noted that these Bayesian methods, while very flexible in some parts of the model, often impose strong distributional assumptions. In most models, the latent exogenous variables $\xi$,

latent residuals $\zeta$, and measurement errors $\varepsilon$ are assumed to be normal, while the residuals are further assumed to be independent.

Moreover, further semi-parametric methods incorporate latent classes (Bauer, 2005; Kelava et al., 2014). These semiparametric methods support non-linear effects and non-normal distributional relations among the latent variables, but as far as we can tell, the space of possible non-linear trends and distributions spanned by these techniques are unknown. For example, in Bauer (2005), there is a fixed number of latent classes within which $(\eta, \xi)$ follow a standard linear and usually normal SEM. In Kelava et al. (2014), this is extended so that within each latent class $(\eta, \xi)$ follow a parametric non-linear and usually normal SEM. The space of possible models for each of these suggestions are likely quite large, and the space spanned by Kelava et al. (2014) likely larger than that of Bauer (2005), but as far as we know, there are no theoretical descriptions of these spaces, and they are not non-parametric in the sense that they are able to estimate any structural relationship without distributional restrictions, at least when using a finite number of latent classes.

Therefore, to the best of our knowledge, the approaches by Kohler et al. (2015) and Kelava et al. (2017) are the only available non-parametric methods which do not impose parametric distributional assumptions.

Two-stage estimation techniques constitute another category of NLSEM methods. These approaches estimate a given functional form, and typically involve using instruments or estimates for the latent variables in a first step, followed by estimating the structural part of the model in a second step. Bollen (1995, Bollen & Paxton, 1998) proposed a two-step instrumental variable approach. Ng and Chan (2020) introduced a simplified version of the (Skrondal & Laake, 2001) method by employing factor scores in the initial step, which are subsequently analyzed using a simple regression model. This simplification is derived from the more complex two-stage method of moments (2SMM) approach by Wall and Amemiya (2000, 2003) where the uncertainty in factor score estimation during parameter estimation and inference in the second step is accounted for. Holst and Budtz-Jørgensen (2020) proposed a semi-parametric approach where $H$ is non-parametrically estimated, but which assumes that the predictors follow a normal distribution. This normality assumption is in contrast to the previously two-step approaches which have minimal or no distributional assumptions.

Finally, extensions to non-continuous data have been proposed in parametric estimation of NLSEM using maximum likelihood (Song & Lee, 2005), marginal maximum likelihood (Jin, Vegelius, & Yang-Wallentin, 2020) or Bayesian techniques (Lee, Song, & Cai, 2010; Song et al., 2013) by the use of link functions. We consider non-continuous data outside the scope of this article.

## Appendix D. Additional Information on the Simulation

D.1. **Data Generating Mechanisms.** Here, we describe the data generating processes used in the simulation study Sections 4.2, 4.3, and 4.4, in more detail. For some derivations of the population values of the trends and model coefficients, we used numeric integration or symbol derivations in `Maple` (Maplesoft, a division of Waterloo Maple Inc.., 2019). The `Maple` version was 2019.2. The `Matlab` (The MathWorks Inc., 2023) version was R2023a.

D.1.1. *Population Models for* $d_\xi = 1$. The model parametrization of the true trends is given in Table 2. We chose $\xi$ to be either standard normally distributed ($\xi \sim \mathcal{N}(0,1)$) or standardized uniform distribution ($\xi \sim \text{unif}\left(-\sqrt{3}, \sqrt{3}\right)$). The residual $\zeta$ of the structural part of the model was chosen to have the same distribution as $\xi$ with its variance being chosen in a way so that $\eta$ has a variance of 1, since $\eta = \mathbb{E}[\eta|\xi] + \zeta$. For the quadratic trend we choose the shape of $\mathbb{E}[\eta|\xi]$ to be identical, which

resulted in differing residual variances, while for all other trends we kept the residual variance $\mathrm{Var}[\zeta]$ to be (almost) identical across the different distributions of $\xi$. Still, we note that the different multivariate distributions of $f = (\xi, \eta)'$ are not directly comparable across different distributions of $\xi$. The trends themselves only differed by a scaling factor (see Table 2). A visualization of the trends for normal $\xi$ is also given in Figure 11 (as depicted by the dashed black line).

The measurement part of the model was chosen to represent data with rather low reliability for the model to yield considerable residual variance. See Table 2 for scale reliabilities and variances of $r$ for the different simulation conditions. The item-wise reliabilities (e.g., for measurement $i$ of $\xi$ it is computed by $(\Lambda_x)_{i1}^2 \mathrm{Var}[\xi] / \left[(\Lambda_x)_{i1}^2 \mathrm{Var}[\xi] + (\Psi_x)_{ii}\right])$ were chosen to be equidistant between .64 and .25 depending on the number of items used. The first factor loadings per latent variable were fixed to 1. Hence, the factor loadings matrix $\Lambda$ and the residual covariance matrix $\Psi$ were chosen as

$$\Psi = \begin{pmatrix} \Psi_x & \mathbf{0}_{d_x, d_y} \\ \mathbf{0}_{d_y, d_x} & \Psi_y \end{pmatrix}, \quad \Lambda = \begin{pmatrix} \Lambda_x & \mathbf{0}_{d_x, d_\eta} \\ \mathbf{0}_{d_y, d_\xi} & \Lambda_y \end{pmatrix}$$

for $d_x = 3, 6, 9$ and $d_y = 3$. For $d_x = 3$, $\Lambda_x$ and $\Psi_x$ where chosen as:

$$\Lambda_x = (1, .65, .5)', \quad \Psi_x = \mathrm{diag}(.5625, .5775, .75).$$

For $d_x = 6$, $\Lambda_x$ and $\Psi_x$ where chosen as:

$$\Lambda_x = (1, .74, .68, .62, .56, .5)', \quad \Psi_x = \mathrm{diag}(.5625, .4524, .5376, .6156, .6864, .75).$$

TABLE 2. Overview of the Parametrization used in the Simulation Study for $d_\xi = 1$

| Trend | $\xi$ | $\mathbb{E}[\eta\|\xi]$ | $\zeta$ | $\mathrm{Var}[\zeta]$ | $\mathbb{E}[\eta]$ | $\mathrm{Var}[\eta]$ |
|---|---|---|---|---|---|---|
| quadratic | norm | $-.5 + .4\xi + .5\xi^2$ | norm | .34 | 0 | 1 |
| quadratic | unif | $-.5 + .4\xi + .5\xi^2$ | unif | .64 | 0 | 1 |
| cubic | norm | $-.128 + 3.2(.4\xi - .4)(.2\xi + .3)\xi$ | norm | .427 | 0 | 1 |
| cubic | unif | $-.4 + 10(.4\xi - .4)(.2\xi + .3)\xi$ | unif | .419 | 0 | 1 |
| logit | norm | $1.776 \exp(2 + 5\xi) / [1 + \exp(2 + 5\xi)] - .647$ | norm | .5 | 0 | 1 |
| logit | unif | $1.671 \exp(2 + 5\xi) / [1 + \exp(2 + 5\xi)] - .615$ | unif | .5 | 0 | 1 |
| piecewise linear | norm | $2.784\,[PL(\xi) - .035]$ | norm | .3 | 0 | 1 |
| piecewise linear | unif | $2.745\,[PL(\xi) - .026]$ | unif | .3 | 0 | 1 |

*Note.* $\xi$ = distribution of $\xi$, $\mathbb{E}[\eta|\xi]$ = parametrization of the conditional expectation of $\eta$ given $\xi$ for $d_\eta = d_\xi = 1$, $\zeta$ = distribution of the residual $\zeta$ for $\eta = \mathbb{E}[\eta|\xi] + \zeta$, $\mathrm{Var}[\zeta]$ = variance of $\zeta$ chosen so that $\mathrm{Var}[\eta] = 1$, with $PL(\xi)$ being the piecewise linear function of $\xi$ given by:

$$PL(\xi) := \begin{cases} .5 + .5\xi, & \text{for } -1 \le \xi < 0, \\ .5 - \xi, & \text{for } 0 \le \xi < 1, \\ -.6 + .1\xi, & \text{for } 1 \le \xi, \\ 0, & \text{else.} \end{cases}$$

All displayed coefficients are rounded to three decimals if more than three decimals are needed; all decimals are given in the code accompanying the simulation study.

Table 3. Measurement Information for $d_\xi = 1$

| $d_x$ | $\mathbb{V}ar[r_\xi]$ | $\mathbb{V}ar[r_\eta]$ | $R^2_\xi$ | $R^2_\eta$ | $\omega_\xi$ | $\omega_\eta$ |
|---|---|---|---|---|---|---|
| 3 | 0.352 | 0.352 | 0.740 | 0.740 | 0.710 | 0.710 |
| 6 | 0.190 | 0.352 | 0.840 | 0.740 | 0.823 | 0.710 |
| 9 | 0.129 | 0.352 | 0.886 | 0.740 | 0.873 | 0.710 |

*Note.* $d_x$ = number of measurements for $\xi$, $\mathbb{V}ar[r_\xi]$ = model implied variance of $r_\xi$, $\mathbb{V}ar[r_\eta]$ = model implied variance of $r_\eta$, $R^2_\xi$ = amount of explained variance of $\ddot{\xi}$ by $\xi$, $R^2_\eta$ = amount of explained variance of $\ddot{\eta}$ by $\eta$, $\omega_\xi$ = McDonald's coefficient of reliability for measuring $\xi$, $\omega_\eta$ = McDonald's coefficient of reliability for measuring $\eta$.

For $d_x = 9$, $\Lambda_x$ and $\Psi_x$ where chosen as:

$$\Lambda_x = (1, .7625, .725, .6875, .65, .6125, .575, .5375, .5)',$$
$$\Psi_x = \text{diag}(.5625, .4185937, .474375, .5273438, .5775, .6248437, .669375, .7110938, .75).$$

$d_y$ was held constant, hence, $\Lambda_y$ and $\Psi_y$ where chosen as for all conditions as:

$$\Lambda_y = (1, .65, .5)', \quad \Psi_y = \text{diag}(.5625, .5775, .75).$$

The measurement errors with covariance matrix $\Psi$ were either independently normal, uniform, or scaled gamma distributed. We did not differentiate between the exogenous and the endogenous parts of the model.

D.1.2. *Population Models for $d_\xi = 2$.* The model parametrization of the true trends is given in Table 4. We extended the univariate simulation conditions by a second exogenous variable so that $\xi = (\xi_1, \xi_2)'$. We chose a normal copula with normal or uniform marginals. As the uniform marginal case with normal copula is not a straight forward object, we used numerical approximations for the variance estimation of $\xi$. Hence, the variance of $\eta$ in that condition is not exactly 1, but close to 1. The chosen trends are rather complex compared to simple linear trends, however, much more complex trends are possible. Hence, this simulation study is limited.

Similarly to the $d_\xi = 1$ case, the measurement part of the model was chosen to represent data with rather low reliability for the model to yield considerable residual variance. See Table 5 for scale reliabilities, McDonald's $\omega$ (R. P. McDonald, 1999), or Bollen's $\omega$ (Bollen, 1980), and variances of $r$ for the different simulation conditions. The aim was to extend the univariate case by a second latent exogenous variable with and without cross relations among the latent exogenous variables. The factor loadings matrix $\Lambda$ and the residual covariance matrix $\Psi$ were chosen as

$$\Psi = \begin{pmatrix} \Psi_x & \mathbf{0}_{d_x,d_y} \\ \mathbf{0}_{d_y,d_x} & \Psi_y \end{pmatrix}, \quad \Lambda = \begin{pmatrix} \Lambda_x & \mathbf{0}_{d_x,d_\eta} \\ \mathbf{0}_{d_y,d_\xi} & \Lambda_y \end{pmatrix}$$

for $d_x = d_{x_1} + d_{x_2}$ with $d_{x_1} = d_{x_2} = 3, 6, 9$ and $d_y = 3$. For models without cross loadings and without residual covariances $\Lambda_x$ simplifies to

$$\Lambda_x = \begin{pmatrix} \Lambda_{x_1} & \mathbf{0}_{d_{x_1},1} \\ \mathbf{0}_{d_{x_2},1} & \Lambda_{x_2} \end{pmatrix}, \qquad .\Psi_x = \begin{pmatrix} \Psi_{x_1} & \mathbf{0}_{d_{x_1},d_{x_2}} \\ \mathbf{0}_{d_{x_2},d_{x_1}} & \Psi_{x_2} \end{pmatrix}$$

Hence, we state $\Lambda_{x_1}, \Lambda_{x_2}, \Psi_{x_1}$, and $\Psi_{x_2}$ in the following. For $d_{x_1} = d_{x_2} = 3$, we have

$$\Lambda_{x_1} = \Lambda_{x_2} = (1, .65, .5)', \quad \Psi_{x_1} = \Psi_{x_2} = \text{diag}(.5625, .5775, .75).$$

For $d_{x_1} = d_{x_2} = 6$, we have

$$\Lambda_{x_1} = \Lambda_{x_2} = (1, .74, .68, .62, .56, .5)', \quad \Psi_{x_1} = \Psi_{x_2} = \text{diag}(.5625, .4524, .5376, .6156, .6864, .75).$$

For $d_{x_1} = d_{x_2} = 9$, we have

$$\Lambda_{x_1} = \Lambda_{x_2} = (1, .7625, .725, .6875, .65, .6125, .575, .5375, .5)',$$
$$\Psi_{x_1} = \Psi_{x_2} = \text{diag}(.5625, .4185937, .474375, .5273438, .5775, .6248437, .669375, .7110938, .75).$$

$d_y$, again, was held constant, hence, $\Lambda_y$ and $\Psi_y$ where chosen as for all conditions as:

$$\Lambda_y = (1, .65, .5)', \quad \Psi_y = \text{diag}(.5625, .5775, .75).$$

For models with cross loadings and cross correlations, we need to adapt the given matrices. Hence, we state the elements of $\Lambda$ and $\Psi$ that needed to change for the corresponding $d_{x_j}$, $j = 1, 2$.

For $d_{x_1} = d_{x_2} = 3$, we changed the following elements in $\Lambda$ to

$$\Lambda_{5,1} = .195,$$

and in $\Psi_x$ to

$$(\Psi_x)_{6,3} = (\Psi_x)_{3,6} = .3.$$

For $d_{x_1} = d_{x_2} = 6$, we changed the following elements in $\Lambda$ to

$$\Lambda_{8,1} = .222, \quad \Lambda_{11,1} = .168,$$

Table 4. Overview of the Parametrization used in the Simulation Study for $d_\xi = 2$

| Trend | $\xi$ | $\mathbb{E}[\eta\|\xi_1, \xi_2]$ | $\zeta$ | Var $[\zeta]$ | $\mathbb{E}[\eta]$ | Var $[\eta]$ |
|---|---|---|---|---|---|---|
| quadratic | norm | $.15 + .45\xi_1 + .32\xi_2 + .3\xi_1\xi_2 - .2\xi_1^2 - .1\xi_2^2$ | norm | .499 | 0 | 1 |
| quadratic | unif | $.15 + .45\xi_1 + .32\xi_2 + .3\xi_1\xi_2 - .2\xi_1^2 - .1\xi_2^2$ | unif | .499 | 0 | 1 |
| cubic | norm | $c(\xi_1, \xi_2)$ | norm | .507 | 0 | 1 |
| cubic | unif | $c(\xi_1, \xi_2)$ | unif | .605 | 0 | .987 |

*Note.* $\xi$ = standardized marginal distributions of $\xi_1$ and $\xi_2$ with normal copula with covariance Cov $[\xi_1, \xi_2] = .5$, $\mathbb{E}[\eta|\xi_1, \xi_2]$ = parametrization of the conditional expectation of $\eta$ given $\xi = (\xi_1, \xi_2)'$ for $d_\eta = 1$, $d_\xi = 2$, $\zeta$ = distribution of the residual $\zeta$ for $\eta = \mathbb{E}[\eta|\xi] + \zeta$, Var $[\zeta]$ = variance of $\zeta$ chosen so that Var $[\eta] = 1$. $c(\xi_1, \xi_2) = .15 + .3\xi_1 + .2\xi_2 + .3\xi_1\xi_2 - .2\xi_1^2 - .1\xi_2^2 + .02\xi_1^3 + .02\xi_2^3 + .06\xi_1\xi_2^2$. The variance of $\eta$ is only an approximation for the uniform marginal $\xi$ case. All displayed coefficients are rounded to three decimals if more than three decimals are needed; all decimals are given in the code accompanying the simulation study.

TABLE 5. Measurement Information for $d_\xi = 2$

| $d_{x_j}$ | cross | $\mathbb{V}ar[r_{\xi_1}]$ | $\mathbb{V}ar[r_{\xi_2}]$ | $\mathbb{C}ov_{12}$ | $\mathbb{C}or_{12}$ | $\mathbb{V}ar[r_\eta]$ | $R^2_{\xi_1}$ | $R^2_{\xi_2}$ | $R^2_\eta$ | $\omega_{\xi_1}$ | $\omega_{\xi_2}$ | $\omega_\eta$ |
|---|---|---|---|---|---|---|---|---|---|---|---|---|
| 3 | no | 0.352 | 0.352 | 0.000 | 0.000 | 0.352 | 0.740 | 0.740 | 0.740 | 0.710 | 0.710 | 0.710 |
| 6 | no | 0.190 | 0.190 | 0.000 | 0.000 | 0.352 | 0.840 | 0.840 | 0.740 | 0.823 | 0.823 | 0.710 |
| 9 | no | 0.129 | 0.129 | 0.000 | 0.000 | 0.352 | 0.886 | 0.886 | 0.740 | 0.873 | 0.873 | 0.710 |
| 3 | yes | 0.334 | 0.313 | -0.016 | -0.048 | 0.388 | 0.749 | 0.761 | 0.720 | 0.710 | 0.728 | 0.657 |
| 6 | yes | 0.174 | 0.150 | -0.007 | -0.046 | 0.388 | 0.852 | 0.869 | 0.720 | 0.823 | 0.837 | 0.657 |
| 9 | yes | 0.117 | 0.099 | -0.005 | -0.042 | 0.388 | 0.895 | 0.910 | 0.720 | 0.873 | 0.884 | 0.657 |

*Note.* $d_{x_j}$ = number of measurements for $\xi_j$, cross = indicator whether cross-loadings or cross-correlations are present, $\mathbb{V}ar[r_{\xi_j}]$ = model implied variance of $r_{\xi_j}$, $\mathbb{C}ov_{12}$ = model implied covariance of $r_\xi$, $\mathbb{C}or_{12}$ = model implied correlation of $r_\xi$, $\mathbb{V}ar[r_\eta]$ = model implied variance of $r_\eta$, $R^2_{\xi_j}$ = amount of explained variance of $\ddot{\xi}_j$ by $\xi_j$, $R^2_\eta$ = amount of explained variance of $\ddot{\eta}$ by $\eta$, $\omega_{\xi_j}$ = McDonald's coefficient or Bollen's coefficient of reliability for measuring $\xi_j$, $\omega_\eta$ = McDonald's coefficient or Bollen's coefficient of reliability for measuring $\eta$; for $j = 1, 2$.

and in $\Psi_x$ to

$$(\Psi_x)_{9,3} = (\Psi_x)_{3,9} = .21504, \quad (\Psi_x)_{12,6} = (\Psi_x)_{6,12} = .3.$$

For $d_{x_1} = d_{x_2} = 9$, we changed the following elements in $\Lambda$ to

$$\Lambda_{11,1} = .22875, \quad \Lambda_{14,1} = .195, \quad \Lambda_{17,1} = .16125,$$

and in $\Psi_x$ to

$$(\Psi_x)_{11,11} = .3662672, \quad (\Psi_x)_{14,14} = .539475, \quad (\Psi_x)_{17,17} = .3662672,$$
$$(\Psi_x)_{12,3} = (\Psi_x)_{3,12} = .18975, \quad (\Psi_x)_{15,6} = (\Psi_x)_{6,15} = .2499375, \quad (\Psi_x)_{18,9} = (\Psi_x)_{9,18} = .3.$$

We further introduced a cross correlation in $\Psi_y$ so that the we changed

$$(\Psi_y)_{2,3} = (\Psi_y)_{3,2} = .2632489.$$

The given cross-loadings in $\Lambda_x$ equal the standardized cross-loadings in value, hence, standardized cross-loadings vary between .229 and .161. The residual covariances in $\Psi$ are chosen in a way so that they result in residual correlations of .4. These are significant but not substantial.

From Table 4 it is evident that by introducing cross relations (i.e., cross-loadings and cross-correlations) the resulting correlation among $r_\xi$ is not large, although the cross relations are not negligible. Further, for increasing $d_{x_j}$ the correlation in $r_\xi$ decreases slightly. It is evident that including cross relation does have an influence on the scale reliability, computed via the extension of McDonald's $\omega$ (R. P. McDonald, 1999) that includes cross-correlations, also called Bollen's $\omega$ (see Bollen, 1980).

The measurement errors with covariance matrix $\Psi$ were either multivariate normal, or they were affine linear transformations of independent uniform or independent scaled gamma variables. We used the singular value decomposition of $\Psi$ in order to correlate the measurement errors with cross-correlations: For $d_z$ i.i.d. standardized measurement errors $\tilde{\varepsilon}$ (e.g., standardized uniform or standardized gamma(1,1)), we computed $\Psi^{\frac{1}{2}}$ via the singular value decomposition $\Psi^{\frac{1}{2}} = VD_\Psi^{\frac{1}{2}}U^{-1}$, where $\Psi = VD_\Psi U^{-1}$ is the singular value decomposition of $\Psi$, $D_\Psi$ is the diagonal matrix containing the singular values (eigenvalues) of $\Psi$ and $V$ is the orthonormal eigenvector matrix that corresponds to the

eigenvalues. Further $U^{-1} = V'$ for positive definite matrices and $D_{\Psi}^{\frac{1}{2}}$ is the matrix that contains the element wise square roots of the eigenvalues in $D_{\Psi}$. Then for

$$\varepsilon := \Psi^{\frac{1}{2}}\tilde{\varepsilon}$$

we have

$$\mathrm{Cov}\left[\varepsilon\right] = \Psi.$$

The marginal distributions may differ between $\varepsilon$ and $\tilde{\varepsilon}$ apart from scaling. For instance, for standardized uniform $\tilde{\varepsilon}$, $\varepsilon$ is no longer marginally uniformly distributed, but for correlated components shows distributions that tends towards the normal due to central limit theorem effects. The same holds true for gamma marginals in $\varepsilon$ compared to $\tilde{\varepsilon}$.

D.2. **Information on R-packages used in the simulation.** All empirical analyses were done in `R` (R Core Team, 2023). Data were generated using `R-base` and `stats` functions for univariate distributions and `mvtnorm` (Genz & Bretz, 2009) and `covsim` (Grønneberg, Foldnes, & Marcoulides, 2022) for specific multivariate distributions for which we wanted to control the marginal distributions and the copula (Nelsen, 2007). The Bartlett factor score and the corresponding CFAs were estimated using `lavaan` (Rosseel, 2012). The nonlinear factor scores proposed by Kelava et al. (2017) were estimated with a modified version of their `MATLAB` (The MathWorks Inc., 2023) scripts called from `R` including their used BSpline method. The HZ-method for local linear estimators for solving errors-in-variables problems including its simulation based cross-validation techniques for bandwidth selection is implemented in the `lpme` package (Huang & Zhou, 2017). We used a slightly modified version of the cross-validation technique by comparing its performance to a rule-of-thumb estimate for the bandwidth that was suggested by Wang and Wang (2011); for further descriptions see Appendix D.3. For the LOESS and the smoothed cubic spline function we used their widely used implementations `loess` and `smooth.spline` within the `stats` package (R Core Team, 2023).

For the examination of performance, we used integration techniques to compute mean integrated squared errors for the nonparametric trends which are further described in Section 4.3. For univariate integrals we used the `integrate` function of the `stats` package (R Core Team, 2023) and for multivariate integrals we used the `cubature` package (Narasimhan, Johnson, Hahn, Bouvier, & Kiêu, 2023). Additional packages for visualization and data handling are described in Appendix D.4. An overview of all package versions is given in Table 6 in the Appendix D.4. All code can be found the online supplementary material.

D.3. **Additional Information on the Estimation of Non-Parametric Trends Used in the Simulation Study.** We here briefly describe the HZ-method of Huang and Zhou (2017) in more detail. Translating their notation to ours, the proposed estimator is defined for the conditional expectation $\mathbb{E}[\ddot{\eta}|\xi = x] = H(x)$, where $\xi$ is measured with error $\ddot{\xi} = \xi + r_{\xi}$, where $\xi$ has density $f_{\xi}(x)$ and $r_{\xi}$ is independent to $(\xi, \ddot{\eta})'$ with known density $f_{r_{\xi}}(x)$. $\xi, r_{\xi}$, and $\ddot{\eta}$ are assumed to be continuous. Then $H^*(w)f_{\ddot{\xi}}(w) = (Hf_{\xi}) * f_{r_{\xi}}(w)$, where $(Hf_{\xi}) * f_{r_{\xi}}(w) = \int H(x)f_{\xi}(x)f_{r_{\xi}}(w-x)dx$ is the convolution (see Delaigle, 2014). Huang and Zhou (2017) then proposed to use the Fourier inverses on both sides, which results in $\phi_{H^*f_{\ddot{\xi}}}(t) = \phi_{Hf_{\xi}}(t)\phi_{r_{\xi}}(t)$, where $\phi_{H^*f_{\ddot{\xi}}}(t)$ is the Fourier transform of $H^*(w)f_{\ddot{\xi}}(w)$, $\phi_{Hf_{\xi}}$ is the Fourier transform of $Hf_{\xi} = H(x)f_{\xi}(x)$. Their local polynomial estimator of order $p$ for $H(x)$ is then given by

$$\hat{H}_{\mathrm{HZ}}(x) = \frac{1}{2\pi \hat{f}_\xi(x)} \int e^{-itx} \frac{\phi_{\hat{H}^* \hat{f}_{\ddot{\xi}}}(t)}{\phi_{r_\xi}(t)} dt, \tag{9}$$

where $\hat{f}_\xi(x)$ is the deconvolution kernel density estimator of $f_\xi(x)$ in Stefanski and Carroll (1990), $\phi_{\hat{H}^* \hat{f}_{\ddot{\xi}}}(t)$ is the Fourier transform of $\hat{H}^*(w)\hat{f}_{\ddot{\xi}}(w)$ in which $\hat{H}^*(w)$ is the $p$th order local polynomial estimator of $H^*(w)$, and $\hat{f}_{\ddot{\xi}}(w)$ is the regular kernel density estimator of $f_{\ddot{\xi}}(w)$ (see, e.g., Fan & Gijbels, 1996, Section 2.7.1). In order to estimate kernel densities a selection of a bandwidth is needed, which can be done using simulation based cross validation techniques for bandwidth selection as proposed by Delaigle and Hall (2008). Although the rationale of Huang and Zhou (2017) can be generalized to multivariate $\xi$, an implementation for the multivariate predictor case with measurement error is still lacking.

During preliminary analyses we noticed that the LOESS and the smoothed spline method produce numerically stable results, while the simulation based cross-validation technique necessary for bandwidth-selection of the HZ-estimator as described in Delaigle and Hall (2008) was rather unstable: here a $k$-fold cross validation sample is drawn, while the sample is refilled in each step to have a total sample size of $n$ (the original sample size) via simulation assuming the distribution of the residual to be valid, as $n$ interacts with the performance of a bandwidth. This process is done several times per cross-validation sample, over which it is then averaged. We choose a 5-fold cross-validation approach with 10 simulations, each. For more detail see the package documentation of the `lpme` package and Delaigle and Hall (2008). The cross-validation technique in the `lpme` package implemented approach (Huang & Zhou, 2017) sometimes produced bandwidth that were too small, which then resulted in strongly oscillating estimated trends. In applied research such a scenario would be noticed by the researchers simply by comparing the trend and the data. However, in a simulation study we needed data driven tools that examine whether a suggested bandwidth is useful without jeopardizing the interpretability of the simulation results. This is why we did not use the MISE as described in Section 4.3 to select a useful bandwidth as it cannot be computed in applied research due to the true trend being unknown. We, therefore, used an estimate for the residual variance in the prediction of the BFS for $\eta$ (namely $\ddot{\eta}$) using the HZ-estimator. As a comparison we used the rule-of-thumb bandwidth for nonparametric regression with measurement error as suggested in Wang and Wang (2011, see eq. (13)), that, translated to our notation and assuming normality for $r_\xi$ for $d_\xi = 1$, is given by

$$bw_{\mathrm{thumb}} := \sqrt{\frac{2\,\mathrm{Var}\left[r_\xi\right]}{\log(n)}}.$$

Although the HZ-estimator using the rule-of-thumb bandwidth is very quick compared to the cross-validation technique (see Table 7), we did not include a rule-of-thumb estimate of the HZ-estimator into our main simulation study as the estimate for $bw_{\mathrm{thumb}}$ has been criticized to not include the variance of the latent variable (the variance of $\ddot{\xi}$ in our notation, the true variance of the BFS) and, therefore, would give a biased estimate for the bandwidth (see for the Laplace case Delaigle, 2014). Probably due to the fact that we chose all latent variables to be standardized, this rule-of-thumb estimate for the bandwidth worked rather well. This is why we used the residual variance in prediction using the rule-of-thumb bandwidth as a comparison for the bandwidth suggested by the cross-validation. If the residual variance in prediction was more than 1.5 times higher for the cross-validation bandwidth compared to the rule-of-thumb bandwidth, we redid the cross-validation step. Hence, we only used the

cross-validation bandwidth $bw_{\text{cv}}$ if

$$\text{Var}\left[\hat{\ddot{\eta}} - \phi_{bw_{\text{cv}}}(\hat{\ddot{\xi}})\right] \leq \frac{3}{2}\,\text{Var}\left[\hat{\ddot{\eta}} - \phi_{bw_{\text{thumb}}}(\hat{\ddot{\xi}})\right],$$

where $\hat{\ddot{\eta}}$ and $\hat{\ddot{\xi}}$ are the empirically estimated BFS for $\eta$ and $\xi$, respectively, and where $\phi_{bw_{\text{cv}}}(\hat{\ddot{\xi}})$ and $\phi_{bw_{\text{thumb}}}(\hat{\ddot{\xi}})$ are the nonparametric estimates for the conditional expectation based on $bw_{\text{cv}}$ and $bw_{\text{thumb}}$, respectively. The difference to the MISE used in simulation studies is that the difference is taken towards an empirical estimate of $\ddot{\eta}$ and not the true conditional expectation $H(x) = \mathbb{E}[\eta|\xi = x]$. The re-initialization of the cross-validation step significantly increases the runtime for some replications within our simulation study, which further explains the large variation of runtimes in Table 7 for the HZCV method.

D.4. **Additional Graphics and Tables with Additional Comments on Simulation Results.** Here we display and comment additional plots and tables. Graphs were done using either `ggplot2` (Wickham, 2016) in combination with `scales` (Wickham & Seidel, 2022), or `rgl` (Murdoch & Adler, 2023) for 3D plots. We further utilized the packages `forcats` (Wickham, 2023) and `papaja` (Aust & Barth, 2022) for data handling and table generation and the `parallel` package (R Core Team, 2023) and the the `pbapply` package (Solymos & Zawadzki, 2023) for parallel computing. Table 6 lists all packages used (also implicitly loaded packages) and their version number. The `R` version was 4.2.2.

Table 6. `R` package versions used

| Package | Version |
|---|---|
| base | 4.2.2 |
| covsim | 1.0.0 |
| cubature | 2.0.4.6 |
| datasets | 4.2.2 |
| forcats | 0.5.2 |
| ggplot2 | 3.4.1 |
| graphics | 4.2.2 |
| grDevices | 4.2.2 |
| lavaan | 0.6.15 |
| lpme | 1.1.3 |
| methods | 4.2.2 |
| mvtnorm | 1.1.3 |
| parallel | 4.2.2 |
| pbapply | 1.7.0 |
| scales | 1.2.1 |
| stats | 4.2.2 |
| utils | 4.2.2 |

*Note.* Implicitly loaded packages are also displayed.

D.4.1. *Additionals for* $d_\xi = 1$. Figure 11 extends Figure 2 with point-wise 95% coverage intervals displaying the uncertainty of the average trends for the distributional condition with normal $\xi$ and gamma $\varepsilon$. It is evident that the linear SEM has the lowest uncertainty, but also approximates the true trend the worst as the true trend is nonlinear. The other methods show similar uncertainty, however, at the edges of the support the HZ methods using cross validation appears to have larger uncertainty compared to the other methods. Still, this difference is not large.

Table 7 shows the average runtime of each factor score based methods used within Figure 2 for a cubic trend. This bench marking was done on a 2019 16-inch MacBook Pro with an 2.6 GHz 6-Core

TABLE 7. Runtime for normal $\xi$ and gamma $\varepsilon$ with a cubic true trend for $d_\xi = 1$

| | | BFS | | HZ-estimator | | NLFS | | |
|---|---|---|---|---|---|---|---|---|
| | $d_x$ | LOESS | Spline | HZTH | HZCV | BSpline | LOESS | Spline |
| mean | 3 | 0.12 | 0.10 | 1.90 | 1,754.43 | 2,115.55 | 2,115.57 | 2,115.56 |
| mean | 9 | 0.11 | 0.08 | 1.73 | 1,493.18 | 2,826.04 | 2,826.06 | 2,826.04 |
| sd | 3 | 0.04 | 0.02 | 0.19 | 732.04 | 259.04 | 259.05 | 259.04 |
| sd | 9 | 0.01 | 0.01 | 0.15 | 623.55 | 210.07 | 210.08 | 210.08 |
| median | 3 | 0.11 | 0.10 | 1.82 | 1,444.00 | 2,192.29 | 2,192.30 | 2,192.29 |
| median | 9 | 0.11 | 0.08 | 1.75 | 1,379.59 | 2,868.18 | 2,868.19 | 2,868.19 |
| LB | 3 | 0.09 | 0.07 | 1.61 | 1,148.59 | 1,324.85 | 1,324.86 | 1,324.85 |
| LB | 9 | 0.09 | 0.07 | 1.42 | 1,121.86 | 2,521.00 | 2,521.01 | 2,521.00 |
| UB | 3 | 0.19 | 0.13 | 2.21 | 3,772.66 | 2,308.38 | 2,308.40 | 2,308.39 |
| UB | 9 | 0.12 | 0.10 | 1.96 | 2,976.04 | 2,972.71 | 2,972.73 | 2,972.71 |

*Note.* Time in seconds aggregated across 32 replications, BFS = Bartlett factor scores, NLFS = nonlinear factor scores, HZTH = HZ-estimator using rule-of-thumb band-width $bw_{\text{thumb}}$, HZCV = HZ-estimator using cross-validation for bandwidth selection $bw_{\text{cv}}$, BSpline = BSpline method for NLFS, LB = lower bound of 95% coverage interval, UB = upper bound of 95% coverage interval.

Intel Core i7 processor and 16 GB RAM. From Table 7 it is evident that the LOESS and spline method based on BFS are extremely quick compared to all other methods. Only the HZ-estimator using the rule-of-thumb bandwidth on average ran for less than 2 seconds. The HZ-estimator using simulation based cross-validated bandwidth took more than 24 minutes and the methods based on NLFS took more than 35 minutes on average. The runtime did not increase but rather decreased with increasing $d_x$ for methods based on BFS or the HZ-estimator but runtime did increase with $d_x$ for methods based on NLFS. Here, runtime was more than 33% longer for $d_x = 9$ compared to $d_x = 3$, on average. Due to the reinitialization of the adapted version of the cross-validation technique for the HZ-estimator as described in Appendix D.3, the HZCV showed the largest variation in runtime with a rather skewed distribution of runtime as suggested by the coverage intervals in Table 7.

Figure 12 emphasizes the relative improvement of MISE in comparison to the linear SEM approximation given that the true trend is nonlinear (see also Table 10 and 11). It is evident that methods based on NLFS showed an increase in MISE compared to the linear SEM in some conditions with only

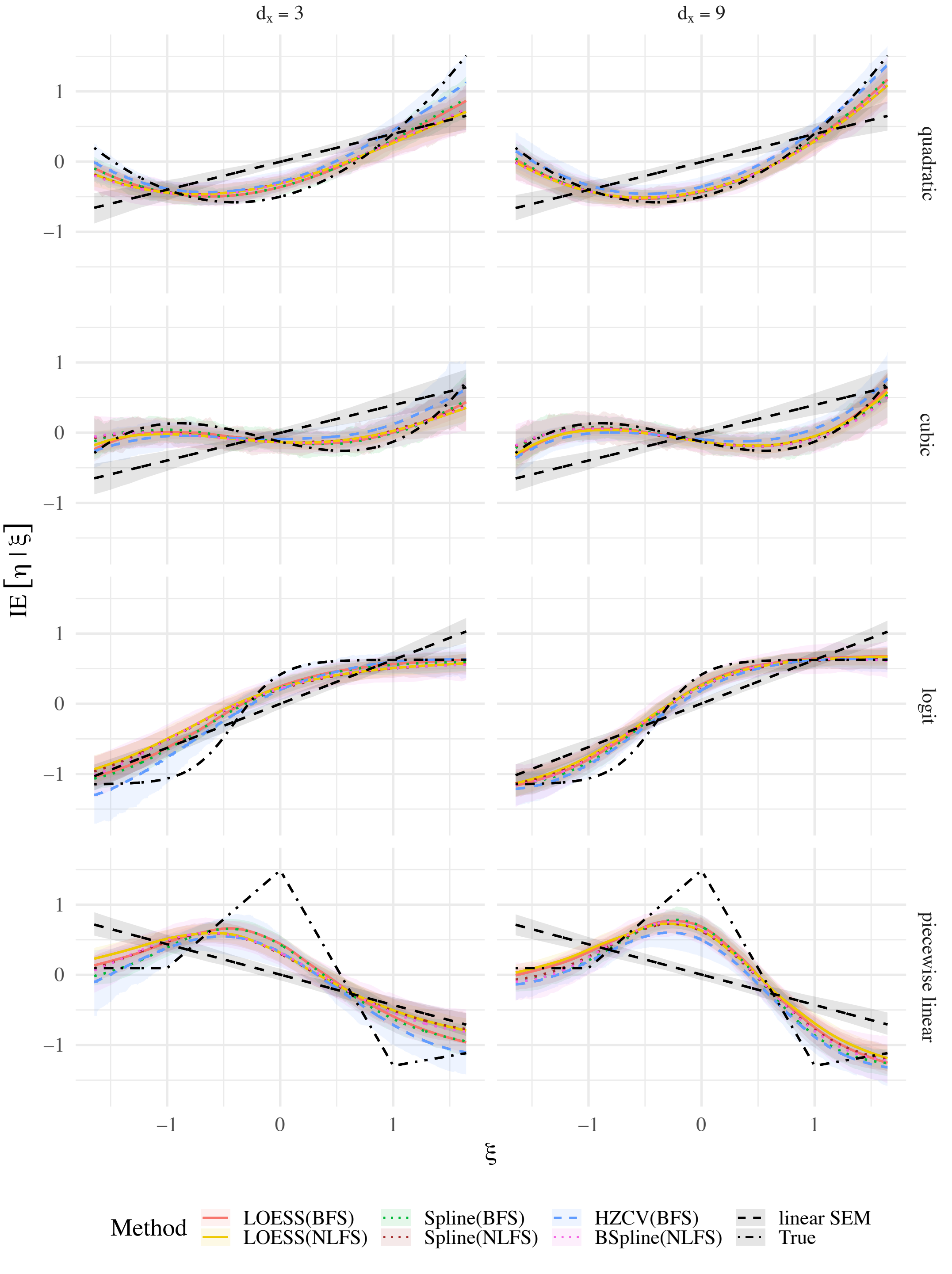


FIGURE 11. *A comparison of nonparametric estimation for* $\mathbb{E}[\eta|\xi]$ *averaged across 200 replications with* $n = 1000$ *for LOESS and smoothed spline based on BFS and the NLFS, the HZ-estimator, the BSpline estimator based on NLFS compared to the true trend and a linear SEM estimation with different true trends (quadratic, cubic, logit and piecewise linear) and dimensions* $d_x$ *with normal* $\xi$ *and gamma distributed errors* $\varepsilon$*. Shaded areas correspond to the 95% coverage interval computed point-wise across the 200 replications.*

Table 8. Average MISE

| Population | | | | LOESS | | | Spline | | | Other | | |
|---|---|---|---|---|---|---|---|---|---|---|---|---|
| Trend | $d_x$ | $\xi$ | $\varepsilon$ | f | BFS | NLFS | f | BFS | NLFS | HZCV | BSNLFS | SEM |
| quadratic | 3 | unif | gamma | 0.007 | 0.127 | 0.193 | 0.010 | 0.125 | 0.203 | 0.195 | 0.208 | 0.449 |
| quadratic | 3 | unif | unif | 0.007 | 0.154 | 0.239 | 0.010 | 0.165 | 0.257 | 0.193 | 0.245 | 0.446 |
| quadratic | 3 | unif | norm | 0.007 | 0.140 | 0.215 | 0.010 | 0.145 | 0.230 | 0.170 | 0.222 | 0.446 |
| quadratic | 3 | norm | gamma | 0.004 | 0.139 | 0.225 | 0.006 | 0.139 | 0.228 | 0.113 | 0.234 | 0.558 |
| quadratic | 3 | norm | unif | 0.004 | 0.190 | 0.308 | 0.006 | 0.195 | 0.312 | 0.119 | 0.330 | 0.560 |
| quadratic | 3 | norm | norm | 0.004 | 0.168 | 0.275 | 0.006 | 0.170 | 0.275 | 0.118 | 0.288 | 0.560 |
| quadratic | 6 | unif | gamma | 0.008 | 0.073 | 0.099 | 0.010 | 0.067 | 0.097 | 0.118 | 0.101 | 0.447 |
| quadratic | 6 | unif | unif | 0.007 | 0.077 | 0.107 | 0.009 | 0.078 | 0.111 | 0.117 | 0.115 | 0.448 |
| quadratic | 6 | unif | norm | 0.007 | 0.076 | 0.105 | 0.009 | 0.076 | 0.108 | 0.110 | 0.113 | 0.444 |
| quadratic | 6 | norm | gamma | 0.004 | 0.076 | 0.115 | 0.006 | 0.080 | 0.118 | 0.066 | 0.127 | 0.560 |
| quadratic | 6 | norm | unif | 0.004 | 0.081 | 0.125 | 0.005 | 0.086 | 0.127 | 0.059 | 0.137 | 0.556 |
| quadratic | 6 | norm | norm | 0.004 | 0.081 | 0.123 | 0.006 | 0.084 | 0.130 | 0.064 | 0.149 | 0.559 |
| quadratic | 9 | unif | gamma | 0.008 | 0.054 | 0.070 | 0.011 | 0.050 | 0.068 | 0.083 | 0.075 | 0.446 |
| quadratic | 9 | unif | unif | 0.008 | 0.054 | 0.072 | 0.010 | 0.052 | 0.073 | 0.082 | 0.071 | 0.449 |
| quadratic | 9 | unif | norm | 0.007 | 0.052 | 0.069 | 0.009 | 0.050 | 0.070 | 0.084 | 0.076 | 0.448 |
| quadratic | 9 | norm | gamma | 0.004 | 0.054 | 0.079 | 0.006 | 0.057 | 0.083 | 0.053 | 0.096 | 0.557 |
| quadratic | 9 | norm | unif | 0.004 | 0.051 | 0.071 | 0.006 | 0.054 | 0.074 | 0.045 | 0.083 | 0.556 |
| quadratic | 9 | norm | norm | 0.004 | 0.050 | 0.074 | 0.006 | 0.053 | 0.075 | 0.047 | 0.089 | 0.557 |
| cubic | 3 | unif | gamma | 0.032 | 0.480 | 0.638 | 0.011 | 0.455 | 0.642 | 0.681 | 0.667 | 1.130 |
| cubic | 3 | unif | unif | 0.031 | 0.548 | 0.703 | 0.011 | 0.550 | 0.719 | 0.688 | 0.707 | 1.133 |
| cubic | 3 | unif | norm | 0.032 | 0.510 | 0.651 | 0.011 | 0.504 | 0.674 | 0.656 | 0.649 | 1.137 |
| cubic | 3 | norm | gamma | 0.022 | 0.046 | 0.068 | 0.011 | 0.066 | 0.087 | 0.123 | 0.090 | 0.541 |
| cubic | 3 | norm | unif | 0.019 | 0.056 | 0.085 | 0.011 | 0.083 | 0.109 | 0.097 | 0.110 | 0.522 |
| cubic | 3 | norm | norm | 0.019 | 0.051 | 0.077 | 0.011 | 0.078 | 0.101 | 0.100 | 0.095 | 0.515 |
| cubic | 6 | unif | gamma | 0.031 | 0.311 | 0.394 | 0.011 | 0.256 | 0.356 | 0.438 | 0.366 | 1.131 |
| cubic | 6 | unif | unif | 0.032 | 0.327 | 0.417 | 0.011 | 0.293 | 0.398 | 0.457 | 0.385 | 1.138 |
| cubic | 6 | unif | norm | 0.031 | 0.320 | 0.408 | 0.010 | 0.278 | 0.384 | 0.446 | 0.372 | 1.124 |
| cubic | 6 | norm | gamma | 0.020 | 0.029 | 0.040 | 0.011 | 0.050 | 0.060 | 0.078 | 0.058 | 0.509 |
| cubic | 6 | norm | unif | 0.021 | 0.032 | 0.042 | 0.011 | 0.053 | 0.068 | 0.083 | 0.065 | 0.531 |
| cubic | 6 | norm | norm | 0.020 | 0.030 | 0.042 | 0.011 | 0.050 | 0.069 | 0.076 | 0.065 | 0.508 |
| cubic | 9 | unif | gamma | 0.031 | 0.234 | 0.296 | 0.010 | 0.173 | 0.242 | 0.362 | 0.255 | 1.125 |
| cubic | 9 | unif | unif | 0.032 | 0.229 | 0.289 | 0.010 | 0.185 | 0.252 | 0.362 | 0.241 | 1.134 |
| cubic | 9 | unif | norm | 0.030 | 0.225 | 0.286 | 0.011 | 0.178 | 0.243 | 0.347 | 0.232 | 1.127 |
| cubic | 9 | norm | gamma | 0.021 | 0.026 | 0.031 | 0.011 | 0.042 | 0.050 | 0.072 | 0.051 | 0.542 |
| cubic | 9 | norm | unif | 0.020 | 0.025 | 0.032 | 0.011 | 0.039 | 0.052 | 0.069 | 0.053 | 0.511 |
| cubic | 9 | norm | norm | 0.019 | 0.025 | 0.028 | 0.010 | 0.040 | 0.047 | 0.066 | 0.052 | 0.513 |

*Note.* MISE to true trend averaged across 200 replications for n = 1000. $\xi$ = distribution of $\xi$, $\varepsilon$ = distribution of $\varepsilon$, $f$ = true latent variables, BFS = Bartlett factor scores, NLFS = nonlinear factor scores, HZCV = HZ-estimator, BSNLFS = BSpline method for NLFS, SEM = linear SEM.

Table 9. Average MISE

| Population | | | | LOESS | | | Spline | | | Other | | |
|---|---|---|---|---|---|---|---|---|---|---|---|---|
| Trend | $d_x$ | $\xi$ | $\varepsilon$ | f | BFS | NLFS | f | BFS | NLFS | HZCV | BSNLFS | SEM |
| logit | 3 | unif | gamma | 0.022 | 0.126 | 0.194 | 0.012 | 0.127 | 0.204 | 0.154 | 0.192 | 0.227 |
| logit | 3 | unif | unif | 0.022 | 0.149 | 0.249 | 0.011 | 0.160 | 0.270 | 0.181 | 0.261 | 0.231 |
| logit | 3 | unif | norm | 0.022 | 0.137 | 0.215 | 0.013 | 0.146 | 0.233 | 0.161 | 0.227 | 0.230 |
| logit | 3 | norm | gamma | 0.023 | 0.201 | 0.337 | 0.012 | 0.192 | 0.326 | 0.151 | 0.331 | 0.307 |
| logit | 3 | norm | unif | 0.024 | 0.244 | 0.402 | 0.013 | 0.253 | 0.422 | 0.177 | 0.402 | 0.306 |
| logit | 3 | norm | norm | 0.022 | 0.239 | 0.373 | 0.013 | 0.250 | 0.398 | 0.174 | 0.388 | 0.309 |
| logit | 6 | unif | gamma | 0.022 | 0.075 | 0.101 | 0.011 | 0.073 | 0.104 | 0.115 | 0.109 | 0.227 |
| logit | 6 | unif | unif | 0.021 | 0.079 | 0.107 | 0.012 | 0.081 | 0.114 | 0.119 | 0.118 | 0.227 |
| logit | 6 | unif | norm | 0.022 | 0.074 | 0.100 | 0.012 | 0.074 | 0.107 | 0.110 | 0.116 | 0.229 |
| logit | 6 | norm | gamma | 0.024 | 0.119 | 0.171 | 0.014 | 0.112 | 0.167 | 0.100 | 0.177 | 0.308 |
| logit | 6 | norm | unif | 0.024 | 0.124 | 0.177 | 0.013 | 0.124 | 0.186 | 0.105 | 0.188 | 0.305 |
| logit | 6 | norm | norm | 0.023 | 0.120 | 0.168 | 0.014 | 0.123 | 0.178 | 0.107 | 0.187 | 0.308 |
| logit | 9 | unif | gamma | 0.022 | 0.056 | 0.070 | 0.012 | 0.052 | 0.070 | 0.089 | 0.082 | 0.225 |
| logit | 9 | unif | unif | 0.021 | 0.057 | 0.074 | 0.011 | 0.055 | 0.076 | 0.095 | 0.087 | 0.226 |
| logit | 9 | unif | norm | 0.023 | 0.057 | 0.072 | 0.012 | 0.054 | 0.074 | 0.093 | 0.088 | 0.225 |
| logit | 9 | norm | gamma | 0.024 | 0.086 | 0.112 | 0.013 | 0.078 | 0.110 | 0.081 | 0.121 | 0.308 |
| logit | 9 | norm | unif | 0.023 | 0.088 | 0.113 | 0.013 | 0.086 | 0.116 | 0.087 | 0.125 | 0.308 |
| logit | 9 | norm | norm | 0.023 | 0.080 | 0.106 | 0.014 | 0.080 | 0.111 | 0.081 | 0.126 | 0.304 |
| piecewise linear | 3 | unif | gamma | 0.125 | 0.581 | 0.829 | 0.012 | 0.510 | 0.797 | 0.748 | 0.807 | 1.467 |
| piecewise linear | 3 | unif | unif | 0.125 | 0.706 | 0.986 | 0.012 | 0.670 | 1.005 | 0.816 | 0.988 | 1.467 |
| piecewise linear | 3 | unif | norm | 0.126 | 0.663 | 0.912 | 0.013 | 0.616 | 0.918 | 0.797 | 0.904 | 1.469 |
| piecewise linear | 3 | norm | gamma | 0.123 | 0.659 | 0.925 | 0.134 | 0.633 | 0.915 | 0.740 | 0.893 | 1.599 |
| piecewise linear | 3 | norm | unif | 0.125 | 0.812 | 1.119 | 0.138 | 0.783 | 1.109 | 0.787 | 1.103 | 1.603 |
| piecewise linear | 3 | norm | norm | 0.125 | 0.734 | 1.014 | 0.138 | 0.706 | 1.019 | 0.720 | 1.002 | 1.599 |
| piecewise linear | 6 | unif | gamma | 0.125 | 0.377 | 0.479 | 0.013 | 0.281 | 0.400 | 0.520 | 0.402 | 1.465 |
| piecewise linear | 6 | unif | unif | 0.126 | 0.426 | 0.552 | 0.012 | 0.336 | 0.489 | 0.566 | 0.483 | 1.466 |
| piecewise linear | 6 | unif | norm | 0.123 | 0.406 | 0.524 | 0.012 | 0.310 | 0.455 | 0.541 | 0.462 | 1.465 |
| piecewise linear | 6 | norm | gamma | 0.124 | 0.418 | 0.548 | 0.139 | 0.386 | 0.523 | 0.550 | 0.526 | 1.597 |
| piecewise linear | 6 | norm | unif | 0.127 | 0.446 | 0.580 | 0.142 | 0.408 | 0.551 | 0.558 | 0.555 | 1.600 |
| piecewise linear | 6 | norm | norm | 0.126 | 0.440 | 0.569 | 0.142 | 0.399 | 0.536 | 0.537 | 0.543 | 1.602 |
| piecewise linear | 9 | unif | gamma | 0.127 | 0.301 | 0.360 | 0.012 | 0.189 | 0.262 | 0.458 | 0.266 | 1.466 |
| piecewise linear | 9 | unif | unif | 0.125 | 0.316 | 0.385 | 0.012 | 0.210 | 0.294 | 0.486 | 0.294 | 1.467 |
| piecewise linear | 9 | unif | norm | 0.122 | 0.300 | 0.365 | 0.012 | 0.193 | 0.274 | 0.431 | 0.277 | 1.466 |
| piecewise linear | 9 | norm | gamma | 0.125 | 0.316 | 0.390 | 0.138 | 0.283 | 0.357 | 0.470 | 0.358 | 1.599 |
| piecewise linear | 9 | norm | unif | 0.126 | 0.327 | 0.398 | 0.141 | 0.291 | 0.366 | 0.499 | 0.381 | 1.598 |
| piecewise linear | 9 | norm | norm | 0.123 | 0.320 | 0.395 | 0.136 | 0.279 | 0.357 | 0.474 | 0.376 | 1.597 |

*Note.* MISE to true trend averaged across 200 replications for n = 1000. $\xi$ = distribution of $\xi$, $\varepsilon$ = distribution of $\varepsilon$, $f$ = true latent variables, BFS = Bartlett factor scores, NLFS = nonlinear factor scores, HZCV = HZ-estimator, BSNLFS = BSpline method for NLFS, SEM = linear SEM.

3 measurements. Further, the largest improvement in MISE occurred for a cubic trend with normal $\xi$, however, when $\xi$ was uniform the improvement in MISE for conditions with cubic trends was comparable to the quadratic trend or the piecewise linear trend. This indicates that the cubic trend is not the furthest from linearity in all conditions. The logit trend is evident to be closest to linearity in terms of showing the smallest improvement compared to the linear SEM approximation.

The boxplots in Figure 13 emphasizes the average MISE per trend and $d_x$ for each method aggregated across all distributional conditions. It, therefore, supplements Figure 3, by including information on the differences across distributional conditions and distinguishes the MISE across different trends. From Figure 13 it is evident that the methods show comparable variation in MISE, hence, comparable heterogeneity across different distributional conditions. This variation decreases with increasing $d_x$ and is comparable among the methods based on factor scores (i.e., LOESS or smoothed splines using BFS or NLFS, as well as the BSpline method using NLFS and the HZ-estimator based on BFS). The methods based on the true latent variables $f$ on average show the smallest variation, i.e., have the highest precision with regard to MISE. In almost all conditions either the spline or LOESS using BFS as inputs performed best aggregated across all distributional conditions. This difference is strongest for the logit or the piecewise linear trend. For the cubic trend differences were not as large. For the quadratic trend the HZCV method showed good performance, also. With regard to variation: the cubic trend showed the largest variation among the MISE across the distributional conditions, but the piecewise linear trend resulted in the largest average MISE.

D.4.2. *Additionals for* $d_\xi = 2$. Figure 14 aggregates Figure 5 of the main text for the cross-relations. Hence, the difference between performance of the LOESS based on BFS and methods based on NLFS are averaged across the two cross-relation conditions. This averaged result shows the benefit of the LOESS based on BFS, as within the computation of the BFS the specific structure of the model may be tested and the BFS may be computed to include all cross-relations among the measurements.

Figure 15 shows all relative average MISE across the 200 replications for all used conditions (see also Table 14 and 15 for numerical values) in comparison to the linear SEM. Hence this figure and these tables show the relative improvement compared to a linear trend given that the actual trend is nonlinear. It is evident that the trends based on NLFS result in larger MISE compared to the linear SEM for many conditions which included cross-relations. Although being slightly less affected, the $\text{BFS}_{uc}$ also showed similar problems. This emphasizes the importance of a correctly specified measurement model.

Figure 16 emphasizes that the LOESS based on BFS is much more homogeneous in the MISE and, hence, in the performance in approximating the true trend. Further, homogeneity increases with increasing numbers of measurements ($d_{x_j}$). The LOESS based on $BFS_{\text{uc}}$ was less heterogeneous across all conditions compared to the methods based on NLFS as highlighted by the whiskers of the Box-Whisker plots.

Figures 17 and 18 depict the three-dimensional true trend. It is evident that the third order effects are not large as the two trends do not differ strongly. However, especially at the borders of the support the third degree effects are visible. The blue and black lines highlight the marginal relation between either $\xi_1$ for given values of $\xi_2$ or vice versa. These marginal relationships are depicted in the following Figures to make a comparison between the non-parametric methods based on BFS or NLFS more evident.

Figures 19, 20, 21, and 22 show the marginal relation between either $\xi_1$ and $H$ for $\xi_2 = 0, -1.6$ or for $\xi_2$ and $H$ for $\xi_1 = 0, -1.6$. For the border condition, i.e., $\xi_1$ or $\xi_2$ being -1.6, all methods show

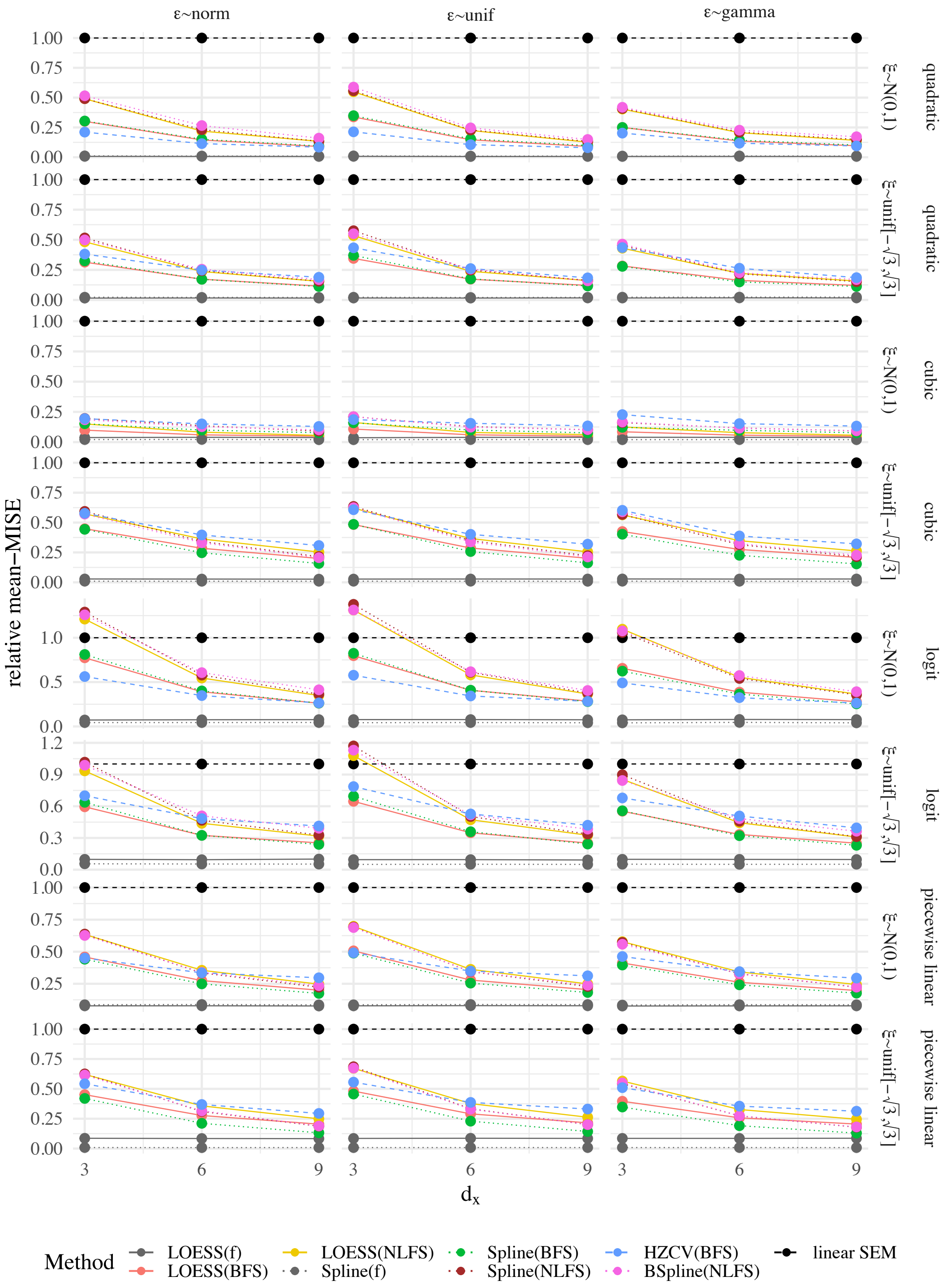


FIGURE 12. *A comparison of the relative averaged MISE in comparison to the linear SEM approximation across 200 replications with $n = 1000$ for different procedures [(B)Splines vs. LOESS vs. HZ/others] based on different inputs (BFS, NLFS, linear SEM, and true latent variables $f$ for comparison) for four models with different true trends (quadratic, cubic, logit and piecewise linear) and dimensions $d_x$. See Table 10 and 11 for numerical values.*

Table 10. Relative average MISE in comparison to linear SEM

| Population | | | | LOESS | | | Spline | | | Other | | |
|---|---|---|---|---|---|---|---|---|---|---|---|---|
| Trend | $d_x$ | $\xi$ | $\varepsilon$ | f | BFS | NLFS | f | BFS | NLFS | HZCV | BSNLFS | SEM |
| quadratic | 3 | unif | gamma | 0.016 | 0.282 | 0.430 | 0.023 | 0.279 | 0.452 | 0.435 | 0.464 | 1.000 |
| quadratic | 3 | unif | unif | 0.016 | 0.345 | 0.535 | 0.022 | 0.370 | 0.575 | 0.433 | 0.550 | 1.000 |
| quadratic | 3 | unif | norm | 0.015 | 0.314 | 0.483 | 0.023 | 0.325 | 0.515 | 0.381 | 0.498 | 1.000 |
| quadratic | 3 | norm | gamma | 0.007 | 0.250 | 0.404 | 0.010 | 0.250 | 0.409 | 0.202 | 0.419 | 1.000 |
| quadratic | 3 | norm | unif | 0.008 | 0.338 | 0.549 | 0.011 | 0.349 | 0.557 | 0.213 | 0.588 | 1.000 |
| quadratic | 3 | norm | norm | 0.008 | 0.301 | 0.491 | 0.012 | 0.304 | 0.491 | 0.211 | 0.515 | 1.000 |
| quadratic | 6 | unif | gamma | 0.017 | 0.163 | 0.221 | 0.023 | 0.150 | 0.217 | 0.263 | 0.226 | 1.000 |
| quadratic | 6 | unif | unif | 0.016 | 0.172 | 0.238 | 0.020 | 0.175 | 0.248 | 0.261 | 0.256 | 1.000 |
| quadratic | 6 | unif | norm | 0.016 | 0.172 | 0.236 | 0.021 | 0.171 | 0.243 | 0.248 | 0.255 | 1.000 |
| quadratic | 6 | norm | gamma | 0.007 | 0.136 | 0.206 | 0.011 | 0.143 | 0.211 | 0.118 | 0.227 | 1.000 |
| quadratic | 6 | norm | unif | 0.006 | 0.146 | 0.224 | 0.009 | 0.154 | 0.229 | 0.106 | 0.246 | 1.000 |
| quadratic | 6 | norm | norm | 0.007 | 0.145 | 0.219 | 0.010 | 0.151 | 0.232 | 0.114 | 0.266 | 1.000 |
| quadratic | 9 | unif | gamma | 0.017 | 0.120 | 0.157 | 0.024 | 0.112 | 0.152 | 0.185 | 0.169 | 1.000 |
| quadratic | 9 | unif | unif | 0.017 | 0.121 | 0.161 | 0.022 | 0.116 | 0.162 | 0.183 | 0.159 | 1.000 |
| quadratic | 9 | unif | norm | 0.016 | 0.115 | 0.155 | 0.021 | 0.112 | 0.156 | 0.188 | 0.169 | 1.000 |
| quadratic | 9 | norm | gamma | 0.007 | 0.096 | 0.141 | 0.010 | 0.102 | 0.149 | 0.095 | 0.172 | 1.000 |
| quadratic | 9 | norm | unif | 0.007 | 0.091 | 0.127 | 0.010 | 0.098 | 0.133 | 0.080 | 0.149 | 1.000 |
| quadratic | 9 | norm | norm | 0.007 | 0.090 | 0.133 | 0.010 | 0.095 | 0.134 | 0.085 | 0.160 | 1.000 |
| cubic | 3 | unif | gamma | 0.028 | 0.425 | 0.564 | 0.010 | 0.402 | 0.568 | 0.602 | 0.590 | 1.000 |
| cubic | 3 | unif | unif | 0.027 | 0.483 | 0.621 | 0.009 | 0.485 | 0.635 | 0.607 | 0.624 | 1.000 |
| cubic | 3 | unif | norm | 0.028 | 0.448 | 0.573 | 0.010 | 0.443 | 0.593 | 0.577 | 0.571 | 1.000 |
| cubic | 3 | norm | gamma | 0.040 | 0.085 | 0.125 | 0.020 | 0.122 | 0.161 | 0.227 | 0.166 | 1.000 |
| cubic | 3 | norm | unif | 0.036 | 0.108 | 0.162 | 0.020 | 0.160 | 0.210 | 0.186 | 0.210 | 1.000 |
| cubic | 3 | norm | norm | 0.038 | 0.099 | 0.150 | 0.021 | 0.152 | 0.196 | 0.194 | 0.185 | 1.000 |
| cubic | 6 | unif | gamma | 0.027 | 0.275 | 0.348 | 0.009 | 0.226 | 0.315 | 0.388 | 0.324 | 1.000 |
| cubic | 6 | unif | unif | 0.028 | 0.287 | 0.367 | 0.010 | 0.258 | 0.350 | 0.402 | 0.338 | 1.000 |
| cubic | 6 | unif | norm | 0.027 | 0.285 | 0.363 | 0.009 | 0.247 | 0.342 | 0.396 | 0.331 | 1.000 |
| cubic | 6 | norm | gamma | 0.039 | 0.057 | 0.078 | 0.021 | 0.098 | 0.117 | 0.153 | 0.115 | 1.000 |
| cubic | 6 | norm | unif | 0.039 | 0.060 | 0.080 | 0.021 | 0.100 | 0.129 | 0.156 | 0.123 | 1.000 |
| cubic | 6 | norm | norm | 0.040 | 0.060 | 0.082 | 0.022 | 0.099 | 0.136 | 0.151 | 0.127 | 1.000 |
| cubic | 9 | unif | gamma | 0.028 | 0.208 | 0.263 | 0.009 | 0.154 | 0.215 | 0.322 | 0.227 | 1.000 |
| cubic | 9 | unif | unif | 0.028 | 0.202 | 0.255 | 0.009 | 0.163 | 0.223 | 0.319 | 0.212 | 1.000 |
| cubic | 9 | unif | norm | 0.027 | 0.200 | 0.253 | 0.009 | 0.158 | 0.215 | 0.308 | 0.206 | 1.000 |
| cubic | 9 | norm | gamma | 0.039 | 0.048 | 0.057 | 0.020 | 0.077 | 0.092 | 0.133 | 0.093 | 1.000 |
| cubic | 9 | norm | unif | 0.040 | 0.049 | 0.062 | 0.021 | 0.076 | 0.102 | 0.134 | 0.103 | 1.000 |
| cubic | 9 | norm | norm | 0.037 | 0.049 | 0.055 | 0.020 | 0.077 | 0.091 | 0.130 | 0.101 | 1.000 |

*Note.* Relative MISE to true trend in comparison to linear SEM averaged across 200 replications for n = 1000. $\xi$ = distribution of $\xi$, $\varepsilon$ = distribution of $\varepsilon$, $f$ = true latent variables, BFS = Bartlett factor scores, NLFS = nonlinear factor scores, HZCV = HZ-estimator, BSNLFS = BSpline method for NLFS, SEM = linear SEM.

Table 11. Relative average MISE in comparison to linear SEM

| Population | | | | LOESS | | | Spline | | | Other | | |
|---|---|---|---|---|---|---|---|---|---|---|---|---|
| Trend | $d_x$ | $\xi$ | $\varepsilon$ | f | BFS | NLFS | f | BFS | NLFS | HZCV | BSNLFS | SEM |
| logit | 3 | unif | gamma | 0.098 | 0.553 | 0.854 | 0.052 | 0.557 | 0.898 | 0.678 | 0.844 | 1.000 |
| logit | 3 | unif | unif | 0.095 | 0.647 | 1.078 | 0.050 | 0.695 | 1.171 | 0.786 | 1.131 | 1.000 |
| logit | 3 | unif | norm | 0.098 | 0.595 | 0.935 | 0.056 | 0.637 | 1.017 | 0.699 | 0.988 | 1.000 |
| logit | 3 | norm | gamma | 0.074 | 0.656 | 1.097 | 0.040 | 0.623 | 1.060 | 0.491 | 1.077 | 1.000 |
| logit | 3 | norm | unif | 0.077 | 0.798 | 1.312 | 0.042 | 0.825 | 1.376 | 0.577 | 1.313 | 1.000 |
| logit | 3 | norm | norm | 0.072 | 0.774 | 1.210 | 0.044 | 0.811 | 1.289 | 0.562 | 1.258 | 1.000 |
| logit | 6 | unif | gamma | 0.098 | 0.333 | 0.444 | 0.051 | 0.322 | 0.458 | 0.508 | 0.483 | 1.000 |
| logit | 6 | unif | unif | 0.095 | 0.349 | 0.473 | 0.051 | 0.359 | 0.503 | 0.527 | 0.519 | 1.000 |
| logit | 6 | unif | norm | 0.094 | 0.325 | 0.439 | 0.054 | 0.324 | 0.468 | 0.483 | 0.509 | 1.000 |
| logit | 6 | norm | gamma | 0.079 | 0.386 | 0.554 | 0.046 | 0.365 | 0.541 | 0.325 | 0.574 | 1.000 |
| logit | 6 | norm | unif | 0.077 | 0.407 | 0.581 | 0.044 | 0.408 | 0.611 | 0.343 | 0.617 | 1.000 |
| logit | 6 | norm | norm | 0.075 | 0.391 | 0.546 | 0.044 | 0.400 | 0.579 | 0.348 | 0.607 | 1.000 |
| logit | 9 | unif | gamma | 0.096 | 0.250 | 0.309 | 0.051 | 0.230 | 0.312 | 0.395 | 0.364 | 1.000 |
| logit | 9 | unif | unif | 0.092 | 0.252 | 0.326 | 0.050 | 0.243 | 0.337 | 0.420 | 0.384 | 1.000 |
| logit | 9 | unif | norm | 0.100 | 0.253 | 0.318 | 0.053 | 0.239 | 0.328 | 0.413 | 0.392 | 1.000 |
| logit | 9 | norm | gamma | 0.077 | 0.278 | 0.365 | 0.041 | 0.255 | 0.356 | 0.263 | 0.392 | 1.000 |
| logit | 9 | norm | unif | 0.075 | 0.286 | 0.366 | 0.042 | 0.280 | 0.377 | 0.283 | 0.405 | 1.000 |
| logit | 9 | norm | norm | 0.077 | 0.262 | 0.349 | 0.045 | 0.262 | 0.364 | 0.265 | 0.413 | 1.000 |
| piecewise linear | 3 | unif | gamma | 0.085 | 0.396 | 0.565 | 0.008 | 0.348 | 0.543 | 0.510 | 0.550 | 1.000 |
| piecewise linear | 3 | unif | unif | 0.085 | 0.481 | 0.672 | 0.008 | 0.456 | 0.685 | 0.556 | 0.674 | 1.000 |
| piecewise linear | 3 | unif | norm | 0.086 | 0.451 | 0.621 | 0.009 | 0.420 | 0.625 | 0.542 | 0.615 | 1.000 |
| piecewise linear | 3 | norm | gamma | 0.077 | 0.412 | 0.578 | 0.084 | 0.396 | 0.572 | 0.462 | 0.558 | 1.000 |
| piecewise linear | 3 | norm | unif | 0.078 | 0.507 | 0.698 | 0.086 | 0.489 | 0.692 | 0.491 | 0.688 | 1.000 |
| piecewise linear | 3 | norm | norm | 0.078 | 0.459 | 0.635 | 0.087 | 0.441 | 0.637 | 0.450 | 0.627 | 1.000 |
| piecewise linear | 6 | unif | gamma | 0.085 | 0.257 | 0.327 | 0.009 | 0.192 | 0.273 | 0.355 | 0.274 | 1.000 |
| piecewise linear | 6 | unif | unif | 0.086 | 0.291 | 0.377 | 0.009 | 0.229 | 0.333 | 0.386 | 0.330 | 1.000 |
| piecewise linear | 6 | unif | norm | 0.084 | 0.277 | 0.358 | 0.008 | 0.211 | 0.310 | 0.369 | 0.315 | 1.000 |
| piecewise linear | 6 | norm | gamma | 0.078 | 0.262 | 0.343 | 0.087 | 0.242 | 0.327 | 0.344 | 0.329 | 1.000 |
| piecewise linear | 6 | norm | unif | 0.079 | 0.279 | 0.363 | 0.089 | 0.255 | 0.345 | 0.349 | 0.347 | 1.000 |
| piecewise linear | 6 | norm | norm | 0.078 | 0.275 | 0.355 | 0.089 | 0.249 | 0.334 | 0.335 | 0.339 | 1.000 |
| piecewise linear | 9 | unif | gamma | 0.086 | 0.205 | 0.246 | 0.008 | 0.129 | 0.179 | 0.313 | 0.181 | 1.000 |
| piecewise linear | 9 | unif | unif | 0.085 | 0.215 | 0.262 | 0.008 | 0.143 | 0.201 | 0.331 | 0.201 | 1.000 |
| piecewise linear | 9 | unif | norm | 0.083 | 0.204 | 0.249 | 0.008 | 0.132 | 0.187 | 0.294 | 0.189 | 1.000 |
| piecewise linear | 9 | norm | gamma | 0.078 | 0.198 | 0.244 | 0.086 | 0.177 | 0.224 | 0.294 | 0.224 | 1.000 |
| piecewise linear | 9 | norm | unif | 0.079 | 0.205 | 0.249 | 0.088 | 0.182 | 0.229 | 0.312 | 0.238 | 1.000 |
| piecewise linear | 9 | norm | norm | 0.077 | 0.200 | 0.247 | 0.085 | 0.175 | 0.223 | 0.297 | 0.235 | 1.000 |

*Note.* Relative MISE to true trend in comparison to linear SEM averaged across 200 replications for n = 1000. $\xi$ = distribution of $\xi$, $\varepsilon$ = distribution of $\varepsilon$, $f$ = true latent variables, BFS = Bartlett factor scores, NLFS = nonlinear factor scores, HZCV = HZ-estimator, BSNLFS = BSpline method for NLFS, SEM = linear SEM.

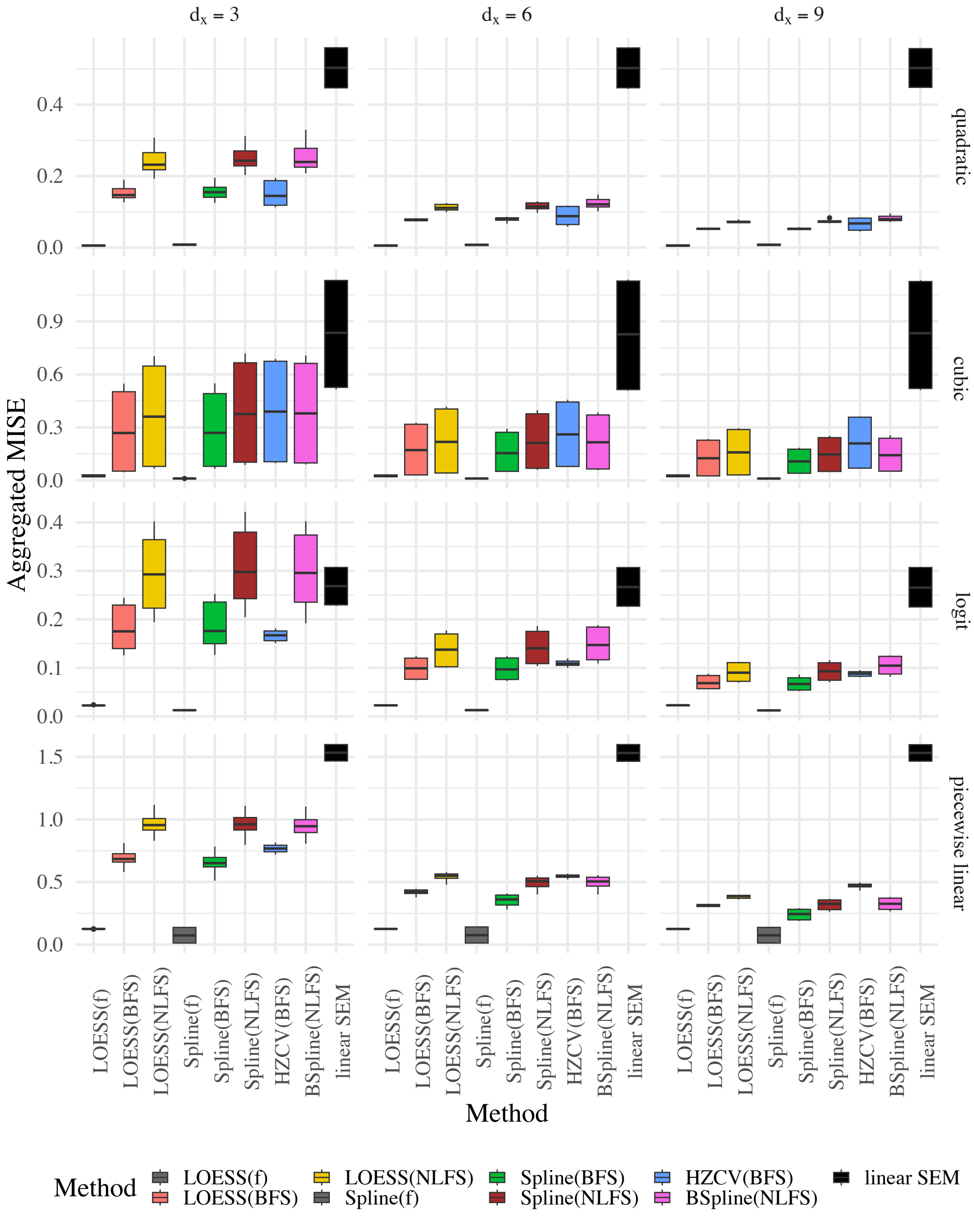


FIGURE 13. *A comparison of the averaged MISE across 200 replications with* $n = 1000$ *for different procedures [(B)Splines vs. LOESS vs. HZ/others] based on different inputs (BFS, NLFS, linear SEM, and true latent variables* $f$ *for comparison) for different dimensions* $d_x$ *aggregated across all distributions used in the simulation study described in Section 4.3.*

TABLE 12. Average MISE with $d_\xi = 2$ without cross-relations

| Population | | | | | LOESS | | | | Other | |
|---|---|---|---|---|---|---|---|---|---|---|
| Cross | Trend | $d_{x_j}$ | $\xi$ | $\varepsilon$ | f | BFS | $\text{BFS}_{\text{uc}}$ | NLFS | BSNLFS | SEM |
| uncrossed | quadratic | 3 | unif | gamma | 0.137 | 0.418 | 0.418 | 0.574 | 0.619 | 0.726 |
| uncrossed | quadratic | 3 | unif | unif | 0.134 | 0.471 | 0.471 | 0.612 | 0.665 | 0.722 |
| uncrossed | quadratic | 3 | unif | norm | 0.140 | 0.484 | 0.484 | 0.622 | 0.659 | 0.730 |
| uncrossed | quadratic | 3 | norm | gamma | 0.153 | 0.435 | 0.435 | 0.608 | 0.670 | 0.628 |
| uncrossed | quadratic | 3 | norm | unif | 0.154 | 0.502 | 0.502 | 0.706 | 0.778 | 0.621 |
| uncrossed | quadratic | 3 | norm | norm | 0.157 | 0.517 | 0.517 | 0.711 | 0.752 | 0.625 |
| uncrossed | quadratic | 6 | unif | gamma | 0.132 | 0.316 | 0.316 | 0.383 | 0.434 | 0.719 |
| uncrossed | quadratic | 6 | unif | unif | 0.131 | 0.353 | 0.353 | 0.420 | 0.476 | 0.720 |
| uncrossed | quadratic | 6 | unif | norm | 0.136 | 0.358 | 0.358 | 0.435 | 0.485 | 0.714 |
| uncrossed | quadratic | 6 | norm | gamma | 0.160 | 0.325 | 0.325 | 0.390 | 0.441 | 0.613 |
| uncrossed | quadratic | 6 | norm | unif | 0.152 | 0.345 | 0.345 | 0.434 | 0.495 | 0.620 |
| uncrossed | quadratic | 6 | norm | norm | 0.163 | 0.362 | 0.362 | 0.443 | 0.523 | 0.618 |
| uncrossed | quadratic | 9 | unif | gamma | 0.137 | 0.277 | 0.277 | 0.329 | 0.382 | 0.716 |
| uncrossed | quadratic | 9 | unif | unif | 0.142 | 0.296 | 0.296 | 0.341 | 0.392 | 0.712 |
| uncrossed | quadratic | 9 | unif | norm | 0.132 | 0.292 | 0.292 | 0.344 | 0.399 | 0.711 |
| uncrossed | quadratic | 9 | norm | gamma | 0.155 | 0.279 | 0.279 | 0.331 | 0.390 | 0.618 |
| uncrossed | quadratic | 9 | norm | unif | 0.154 | 0.296 | 0.296 | 0.343 | 0.418 | 0.617 |
| uncrossed | quadratic | 9 | norm | norm | 0.158 | 0.289 | 0.289 | 0.340 | 0.406 | 0.613 |
| uncrossed | cubic | 3 | unif | gamma | 0.141 | 0.412 | 0.412 | 0.535 | 0.588 | 0.752 |
| uncrossed | cubic | 3 | unif | unif | 0.146 | 0.459 | 0.459 | 0.592 | 0.649 | 0.747 |
| uncrossed | cubic | 3 | unif | norm | 0.144 | 0.478 | 0.478 | 0.596 | 0.641 | 0.752 |
| uncrossed | cubic | 3 | norm | gamma | 0.172 | 0.402 | 0.402 | 0.547 | 0.603 | 0.713 |
| uncrossed | cubic | 3 | norm | unif | 0.167 | 0.456 | 0.456 | 0.601 | 0.665 | 0.726 |
| uncrossed | cubic | 3 | norm | norm | 0.168 | 0.461 | 0.461 | 0.611 | 0.656 | 0.718 |
| uncrossed | cubic | 6 | unif | gamma | 0.144 | 0.328 | 0.328 | 0.390 | 0.440 | 0.744 |
| uncrossed | cubic | 6 | unif | unif | 0.147 | 0.357 | 0.357 | 0.426 | 0.487 | 0.740 |
| uncrossed | cubic | 6 | unif | norm | 0.145 | 0.350 | 0.350 | 0.421 | 0.474 | 0.737 |
| uncrossed | cubic | 6 | norm | gamma | 0.166 | 0.297 | 0.297 | 0.358 | 0.436 | 0.707 |
| uncrossed | cubic | 6 | norm | unif | 0.170 | 0.339 | 0.339 | 0.402 | 0.464 | 0.716 |
| uncrossed | cubic | 6 | norm | norm | 0.169 | 0.324 | 0.324 | 0.403 | 0.462 | 0.709 |
| uncrossed | cubic | 9 | unif | gamma | 0.145 | 0.279 | 0.279 | 0.336 | 0.405 | 0.735 |
| uncrossed | cubic | 9 | unif | unif | 0.144 | 0.290 | 0.290 | 0.344 | 0.401 | 0.732 |
| uncrossed | cubic | 9 | unif | norm | 0.140 | 0.292 | 0.292 | 0.342 | 0.406 | 0.733 |
| uncrossed | cubic | 9 | norm | gamma | 0.171 | 0.269 | 0.269 | 0.321 | 0.380 | 0.696 |
| uncrossed | cubic | 9 | norm | unif | 0.168 | 0.279 | 0.279 | 0.327 | 0.386 | 0.709 |
| uncrossed | cubic | 9 | norm | norm | 0.165 | 0.268 | 0.268 | 0.318 | 0.361 | 0.713 |

*Note.* MISE to true trend averaged across 200 replications for n = 1000. Cross = if crossed, then cross relations were present, $\xi$ = distribution of $\xi$, $\varepsilon$ = distribution of $\varepsilon$, $f$ = true latent variables, BFS = Bartlett factor scores, $\text{BFS}_{\text{uc}}$ = Bartlett factor scores without cross-relations in corresponding CFA, NLFS = nonlinear factor scores, BSNLFS = BSpline method for NLFS, SEM = linear SEM.

Table 13. Average MISE with $d_\xi = 2$ with cross-relations

| Population | | | | | LOESS | | | | Other | |
|---|---|---|---|---|---|---|---|---|---|---|
| Cross | Trend | $d_{x_j}$ | $\xi$ | $\varepsilon$ | f | BFS | $\text{BFS}_{\text{uc}}$ | NLFS | BSNLFS | SEM |
| crossed | quadratic | 3 | unif | gamma | 0.133 | 0.408 | 0.687 | 0.892 | 0.926 | 0.724 |
| crossed | quadratic | 3 | unif | unif | 0.134 | 0.467 | 0.780 | 1.001 | 1.048 | 0.722 |
| crossed | quadratic | 3 | unif | norm | 0.127 | 0.468 | 0.792 | 1.008 | 1.045 | 0.722 |
| crossed | quadratic | 3 | norm | gamma | 0.151 | 0.441 | 0.810 | 1.111 | 1.153 | 0.624 |
| crossed | quadratic | 3 | norm | unif | 0.156 | 0.500 | 0.944 | 1.275 | 1.324 | 0.625 |
| crossed | quadratic | 3 | norm | norm | 0.159 | 0.512 | 0.936 | 1.292 | 1.315 | 0.623 |
| crossed | quadratic | 6 | unif | gamma | 0.136 | 0.310 | 0.582 | 0.708 | 0.729 | 0.719 |
| crossed | quadratic | 6 | unif | unif | 0.138 | 0.334 | 0.622 | 0.758 | 0.786 | 0.716 |
| crossed | quadratic | 6 | unif | norm | 0.140 | 0.355 | 0.630 | 0.755 | 0.793 | 0.716 |
| crossed | quadratic | 6 | norm | gamma | 0.157 | 0.316 | 0.656 | 0.842 | 0.895 | 0.612 |
| crossed | quadratic | 6 | norm | unif | 0.155 | 0.335 | 0.705 | 0.886 | 0.929 | 0.618 |
| crossed | quadratic | 6 | norm | norm | 0.155 | 0.337 | 0.701 | 0.886 | 0.919 | 0.619 |
| crossed | quadratic | 9 | unif | gamma | 0.135 | 0.271 | 0.532 | 0.642 | 0.665 | 0.716 |
| crossed | quadratic | 9 | unif | unif | 0.134 | 0.278 | 0.538 | 0.654 | 0.694 | 0.713 |
| crossed | quadratic | 9 | unif | norm | 0.134 | 0.290 | 0.536 | 0.658 | 0.691 | 0.713 |
| crossed | quadratic | 9 | norm | gamma | 0.158 | 0.275 | 0.598 | 0.736 | 0.776 | 0.617 |
| crossed | quadratic | 9 | norm | unif | 0.165 | 0.289 | 0.620 | 0.766 | 0.809 | 0.619 |
| crossed | quadratic | 9 | norm | norm | 0.155 | 0.278 | 0.603 | 0.756 | 0.805 | 0.617 |
| crossed | cubic | 3 | unif | gamma | 0.138 | 0.414 | 0.641 | 0.724 | 0.764 | 0.745 |
| crossed | cubic | 3 | unif | unif | 0.146 | 0.456 | 0.685 | 0.813 | 0.844 | 0.748 |
| crossed | cubic | 3 | unif | norm | 0.143 | 0.475 | 0.709 | 0.814 | 0.849 | 0.747 |
| crossed | cubic | 3 | norm | gamma | 0.171 | 0.390 | 0.635 | 0.817 | 0.859 | 0.724 |
| crossed | cubic | 3 | norm | unif | 0.169 | 0.458 | 0.730 | 0.948 | 1.005 | 0.714 |
| crossed | cubic | 3 | norm | norm | 0.171 | 0.435 | 0.690 | 0.923 | 0.959 | 0.726 |
| crossed | cubic | 6 | unif | gamma | 0.143 | 0.321 | 0.534 | 0.577 | 0.608 | 0.745 |
| crossed | cubic | 6 | unif | unif | 0.145 | 0.341 | 0.564 | 0.609 | 0.640 | 0.743 |
| crossed | cubic | 6 | unif | norm | 0.141 | 0.336 | 0.563 | 0.615 | 0.635 | 0.739 |
| crossed | cubic | 6 | norm | gamma | 0.171 | 0.300 | 0.536 | 0.643 | 0.684 | 0.704 |
| crossed | cubic | 6 | norm | unif | 0.161 | 0.314 | 0.551 | 0.665 | 0.718 | 0.705 |
| crossed | cubic | 6 | norm | norm | 0.164 | 0.314 | 0.551 | 0.663 | 0.700 | 0.710 |
| crossed | cubic | 9 | unif | gamma | 0.140 | 0.281 | 0.486 | 0.525 | 0.561 | 0.741 |
| crossed | cubic | 9 | unif | unif | 0.145 | 0.287 | 0.502 | 0.537 | 0.587 | 0.734 |
| crossed | cubic | 9 | unif | norm | 0.145 | 0.286 | 0.503 | 0.536 | 0.569 | 0.738 |
| crossed | cubic | 9 | norm | gamma | 0.168 | 0.260 | 0.470 | 0.547 | 0.587 | 0.702 |
| crossed | cubic | 9 | norm | unif | 0.168 | 0.269 | 0.485 | 0.566 | 0.618 | 0.701 |
| crossed | cubic | 9 | norm | norm | 0.165 | 0.274 | 0.499 | 0.573 | 0.607 | 0.702 |

*Note.* MISE to true trend averaged across 200 replications for n = 1000. Cross = if crossed, then cross relations were present, $\xi$ = distribution of $\xi$, $\varepsilon$ = distribution of $\varepsilon$, $f$ = true latent variables, BFS = Bartlett factor scores, $\text{BFS}_{\text{uc}}$ = Bartlett factor scores without cross-relations in corresponding CFA, NLFS = nonlinear factor scores, BSNLFS = BSpline method for NLFS, SEM = linear SEM.

TABLE 14. Relative average MISE with $d_\xi = 2$ without cross-relations in comparison to linear SEM

| Population | | | | | LOESS | | | | Other | |
|---|---|---|---|---|---|---|---|---|---|---|
| Cross | Trend | $d_{x_j}$ | $\xi$ | $\varepsilon$ | f | BFS | $BFS_{uc}$ | NLFS | BSNLFS | SEM |
| uncrossed | quadratic | 3 | unif | gamma | 0.189 | 0.575 | 0.575 | 0.790 | 0.852 | 1.000 |
| uncrossed | quadratic | 3 | unif | unif | 0.186 | 0.653 | 0.653 | 0.848 | 0.922 | 1.000 |
| uncrossed | quadratic | 3 | unif | norm | 0.192 | 0.662 | 0.662 | 0.851 | 0.903 | 1.000 |
| uncrossed | quadratic | 3 | norm | gamma | 0.244 | 0.692 | 0.692 | 0.968 | 1.066 | 1.000 |
| uncrossed | quadratic | 3 | norm | unif | 0.248 | 0.808 | 0.808 | 1.136 | 1.252 | 1.000 |
| uncrossed | quadratic | 3 | norm | norm | 0.251 | 0.828 | 0.828 | 1.139 | 1.204 | 1.000 |
| uncrossed | quadratic | 6 | unif | gamma | 0.184 | 0.440 | 0.440 | 0.533 | 0.603 | 1.000 |
| uncrossed | quadratic | 6 | unif | unif | 0.181 | 0.490 | 0.490 | 0.584 | 0.660 | 1.000 |
| uncrossed | quadratic | 6 | unif | norm | 0.191 | 0.502 | 0.502 | 0.610 | 0.679 | 1.000 |
| uncrossed | quadratic | 6 | norm | gamma | 0.260 | 0.529 | 0.529 | 0.636 | 0.718 | 1.000 |
| uncrossed | quadratic | 6 | norm | unif | 0.245 | 0.557 | 0.557 | 0.699 | 0.797 | 1.000 |
| uncrossed | quadratic | 6 | norm | norm | 0.264 | 0.585 | 0.585 | 0.717 | 0.846 | 1.000 |
| uncrossed | quadratic | 9 | unif | gamma | 0.191 | 0.387 | 0.387 | 0.460 | 0.534 | 1.000 |
| uncrossed | quadratic | 9 | unif | unif | 0.200 | 0.416 | 0.416 | 0.479 | 0.550 | 1.000 |
| uncrossed | quadratic | 9 | unif | norm | 0.186 | 0.411 | 0.411 | 0.484 | 0.562 | 1.000 |
| uncrossed | quadratic | 9 | norm | gamma | 0.251 | 0.451 | 0.451 | 0.535 | 0.631 | 1.000 |
| uncrossed | quadratic | 9 | norm | unif | 0.250 | 0.480 | 0.480 | 0.556 | 0.678 | 1.000 |
| uncrossed | quadratic | 9 | norm | norm | 0.257 | 0.471 | 0.471 | 0.555 | 0.661 | 1.000 |
| uncrossed | cubic | 3 | unif | gamma | 0.187 | 0.548 | 0.548 | 0.711 | 0.782 | 1.000 |
| uncrossed | cubic | 3 | unif | unif | 0.195 | 0.614 | 0.614 | 0.793 | 0.868 | 1.000 |
| uncrossed | cubic | 3 | unif | norm | 0.191 | 0.635 | 0.635 | 0.793 | 0.852 | 1.000 |
| uncrossed | cubic | 3 | norm | gamma | 0.241 | 0.563 | 0.563 | 0.768 | 0.847 | 1.000 |
| uncrossed | cubic | 3 | norm | unif | 0.230 | 0.629 | 0.629 | 0.828 | 0.916 | 1.000 |
| uncrossed | cubic | 3 | norm | norm | 0.234 | 0.643 | 0.643 | 0.851 | 0.914 | 1.000 |
| uncrossed | cubic | 6 | unif | gamma | 0.194 | 0.441 | 0.441 | 0.524 | 0.591 | 1.000 |
| uncrossed | cubic | 6 | unif | unif | 0.199 | 0.483 | 0.483 | 0.575 | 0.658 | 1.000 |
| uncrossed | cubic | 6 | unif | norm | 0.197 | 0.475 | 0.475 | 0.571 | 0.643 | 1.000 |
| uncrossed | cubic | 6 | norm | gamma | 0.235 | 0.421 | 0.421 | 0.506 | 0.617 | 1.000 |
| uncrossed | cubic | 6 | norm | unif | 0.237 | 0.474 | 0.474 | 0.561 | 0.648 | 1.000 |
| uncrossed | cubic | 6 | norm | norm | 0.238 | 0.457 | 0.457 | 0.568 | 0.651 | 1.000 |
| uncrossed | cubic | 9 | unif | gamma | 0.197 | 0.379 | 0.379 | 0.457 | 0.551 | 1.000 |
| uncrossed | cubic | 9 | unif | unif | 0.196 | 0.396 | 0.396 | 0.470 | 0.548 | 1.000 |
| uncrossed | cubic | 9 | unif | norm | 0.191 | 0.398 | 0.398 | 0.467 | 0.554 | 1.000 |
| uncrossed | cubic | 9 | norm | gamma | 0.245 | 0.387 | 0.387 | 0.461 | 0.546 | 1.000 |
| uncrossed | cubic | 9 | norm | unif | 0.237 | 0.393 | 0.393 | 0.462 | 0.544 | 1.000 |
| uncrossed | cubic | 9 | norm | norm | 0.231 | 0.376 | 0.376 | 0.447 | 0.507 | 1.000 |

*Note.* Relative MISE to true trend in comparison to linear SEM averaged across 200 replications for n = 1000. Cross = if crossed, then cross relations were present, $\xi$ = distribution of $\xi$, $\varepsilon$ = distribution of $\varepsilon$, $f$ = true latent variables, BFS = Bartlett factor scores, $BFS_{uc}$ = Bartlett factor scores without cross-relations in corresponding CFA, NLFS = nonlinear factor scores, BSNLFS = BSpline method for NLFS, SEM = linear SEM.

TABLE 15. Relative average MISE with $d_\xi = 2$ with cross-relations in comparison to linear SEM

| Population | | | | | LOESS | | | | Other | |
|---|---|---|---|---|---|---|---|---|---|---|
| Cross | Trend | $d_{x_j}$ | $\xi$ | $\varepsilon$ | f | BFS | $BFS_{uc}$ | NLFS | BSNLFS | SEM |
| crossed | quadratic | 3 | unif | gamma | 0.184 | 0.564 | 0.949 | 1.233 | 1.280 | 1.000 |
| crossed | quadratic | 3 | unif | unif | 0.185 | 0.647 | 1.082 | 1.387 | 1.452 | 1.000 |
| crossed | quadratic | 3 | unif | norm | 0.176 | 0.648 | 1.096 | 1.396 | 1.447 | 1.000 |
| crossed | quadratic | 3 | norm | gamma | 0.243 | 0.707 | 1.298 | 1.780 | 1.847 | 1.000 |
| crossed | quadratic | 3 | norm | unif | 0.250 | 0.800 | 1.511 | 2.041 | 2.119 | 1.000 |
| crossed | quadratic | 3 | norm | norm | 0.255 | 0.821 | 1.501 | 2.073 | 2.109 | 1.000 |
| crossed | quadratic | 6 | unif | gamma | 0.189 | 0.431 | 0.810 | 0.985 | 1.015 | 1.000 |
| crossed | quadratic | 6 | unif | unif | 0.193 | 0.467 | 0.869 | 1.059 | 1.098 | 1.000 |
| crossed | quadratic | 6 | unif | norm | 0.195 | 0.496 | 0.880 | 1.054 | 1.108 | 1.000 |
| crossed | quadratic | 6 | norm | gamma | 0.256 | 0.516 | 1.071 | 1.374 | 1.461 | 1.000 |
| crossed | quadratic | 6 | norm | unif | 0.251 | 0.542 | 1.141 | 1.432 | 1.502 | 1.000 |
| crossed | quadratic | 6 | norm | norm | 0.251 | 0.544 | 1.133 | 1.432 | 1.486 | 1.000 |
| crossed | quadratic | 9 | unif | gamma | 0.188 | 0.379 | 0.742 | 0.896 | 0.928 | 1.000 |
| crossed | quadratic | 9 | unif | unif | 0.188 | 0.390 | 0.756 | 0.918 | 0.974 | 1.000 |
| crossed | quadratic | 9 | unif | norm | 0.188 | 0.407 | 0.752 | 0.923 | 0.969 | 1.000 |
| crossed | quadratic | 9 | norm | gamma | 0.256 | 0.446 | 0.969 | 1.193 | 1.258 | 1.000 |
| crossed | quadratic | 9 | norm | unif | 0.266 | 0.466 | 1.002 | 1.236 | 1.306 | 1.000 |
| crossed | quadratic | 9 | norm | norm | 0.252 | 0.450 | 0.976 | 1.225 | 1.304 | 1.000 |
| crossed | cubic | 3 | unif | gamma | 0.186 | 0.555 | 0.861 | 0.972 | 1.025 | 1.000 |
| crossed | cubic | 3 | unif | unif | 0.195 | 0.609 | 0.916 | 1.087 | 1.129 | 1.000 |
| crossed | cubic | 3 | unif | norm | 0.191 | 0.635 | 0.949 | 1.089 | 1.137 | 1.000 |
| crossed | cubic | 3 | norm | gamma | 0.237 | 0.538 | 0.877 | 1.128 | 1.186 | 1.000 |
| crossed | cubic | 3 | norm | unif | 0.237 | 0.641 | 1.023 | 1.328 | 1.407 | 1.000 |
| crossed | cubic | 3 | norm | norm | 0.235 | 0.599 | 0.951 | 1.272 | 1.322 | 1.000 |
| crossed | cubic | 6 | unif | gamma | 0.193 | 0.431 | 0.717 | 0.775 | 0.816 | 1.000 |
| crossed | cubic | 6 | unif | unif | 0.195 | 0.459 | 0.759 | 0.819 | 0.860 | 1.000 |
| crossed | cubic | 6 | unif | norm | 0.191 | 0.455 | 0.762 | 0.833 | 0.860 | 1.000 |
| crossed | cubic | 6 | norm | gamma | 0.243 | 0.426 | 0.761 | 0.913 | 0.970 | 1.000 |
| crossed | cubic | 6 | norm | unif | 0.228 | 0.445 | 0.782 | 0.945 | 1.019 | 1.000 |
| crossed | cubic | 6 | norm | norm | 0.231 | 0.442 | 0.776 | 0.934 | 0.987 | 1.000 |
| crossed | cubic | 9 | unif | gamma | 0.189 | 0.379 | 0.656 | 0.709 | 0.757 | 1.000 |
| crossed | cubic | 9 | unif | unif | 0.198 | 0.391 | 0.684 | 0.731 | 0.800 | 1.000 |
| crossed | cubic | 9 | unif | norm | 0.196 | 0.387 | 0.682 | 0.727 | 0.771 | 1.000 |
| crossed | cubic | 9 | norm | gamma | 0.239 | 0.371 | 0.669 | 0.779 | 0.836 | 1.000 |
| crossed | cubic | 9 | norm | unif | 0.239 | 0.383 | 0.691 | 0.807 | 0.881 | 1.000 |
| crossed | cubic | 9 | norm | norm | 0.235 | 0.389 | 0.711 | 0.816 | 0.864 | 1.000 |

*Note.* Relative MISE to true trend in comparison to linear SEM averaged across 200 replications for n = 1000. Cross = if crossed, then cross relations were present, $\xi$ = distribution of $\xi$, $\varepsilon$ = distribution of $\varepsilon$, $f$ = true latent variables, BFS = Bartlett factor scores, $BFS_{uc}$ = Bartlett factor scores without cross-relations in corresponding CFA, NLFS = nonlinear factor scores, BSNLFS = BSpline method for NLFS, SEM = linear SEM.

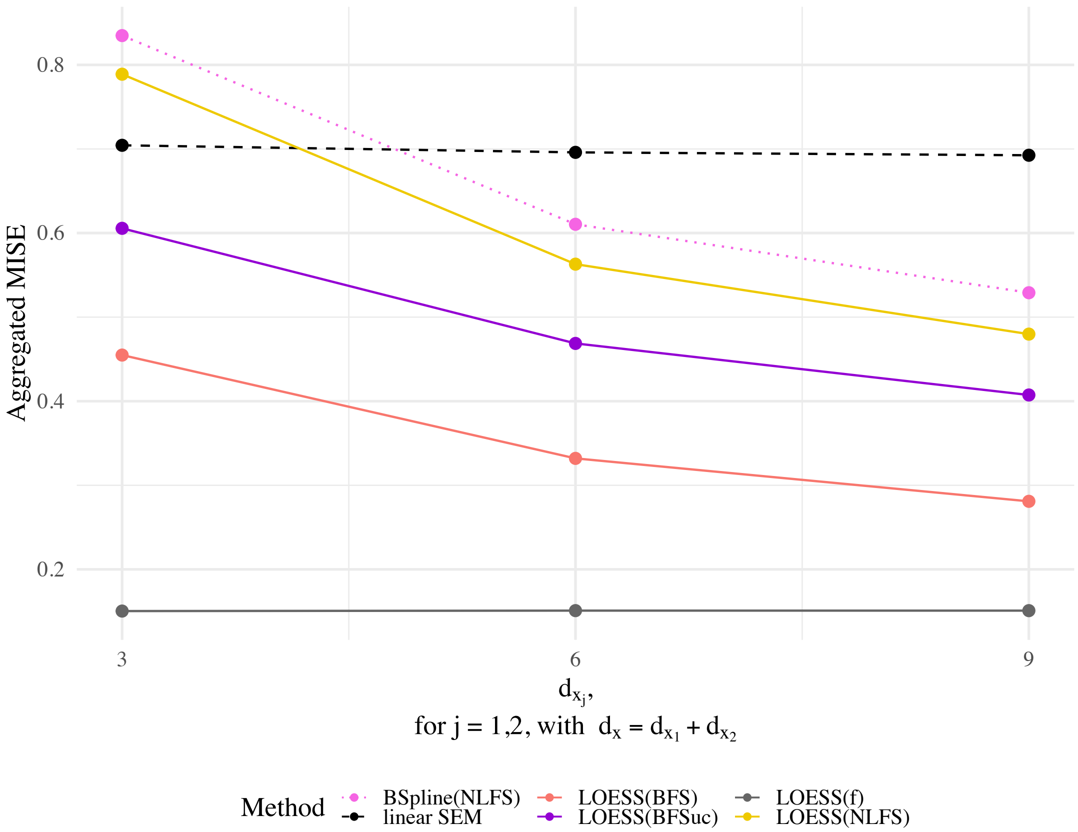


FIGURE 14. *A comparison of the averaged MISE across 200 replications with* $n = 1000$ *for different procedures [(B)Splines vs. LOESS vs. HZ/others] based on different inputs (BFS, NLFS, linear SEM, and true latent variables* $f$ *for comparison) for different dimensions* $d_{x_j}$ *aggregated across all distributions, trends and inclusion of cross-relations (cross-loadings and cross-correlations in* $\Lambda_x, \Psi_x,$ *and* $\Psi_y$*) used in the simulation study.*

poor performance in approximating the true trend. However, for $\xi_1$ or $\xi_2$ being 0, i.e., the center of the distribution, the LOESS based on BFS outperforms the other methods. This difference is larger in the conditions where cross-relations are present (see Figures 21, and 22), where also LOESS based on BFS$_{uc}$ differs from LOESS based on BFS using the true model. However, LOESS based on BFS$_{uc}$ still outperforms the methods based on NLFS on average. This suggests that for the presented scenarios even a misspecified Bartlett score results in a better non-parametric estimation of the trend compared to the methods based on NLFS. Further, the methods based on BFS show slightly less variation as highlighted by the confidence bands in Figures 19 and 20.

To summarize, similarly to the univariate case, the non-parametric methods approach the true trend for increasing numbers of measurements with LOESS based on BFS showing better approximations to the true trend as already suggested by Figure 5 of the main text. However, the difference to the true trend appears slightly larger than in the univariate case.

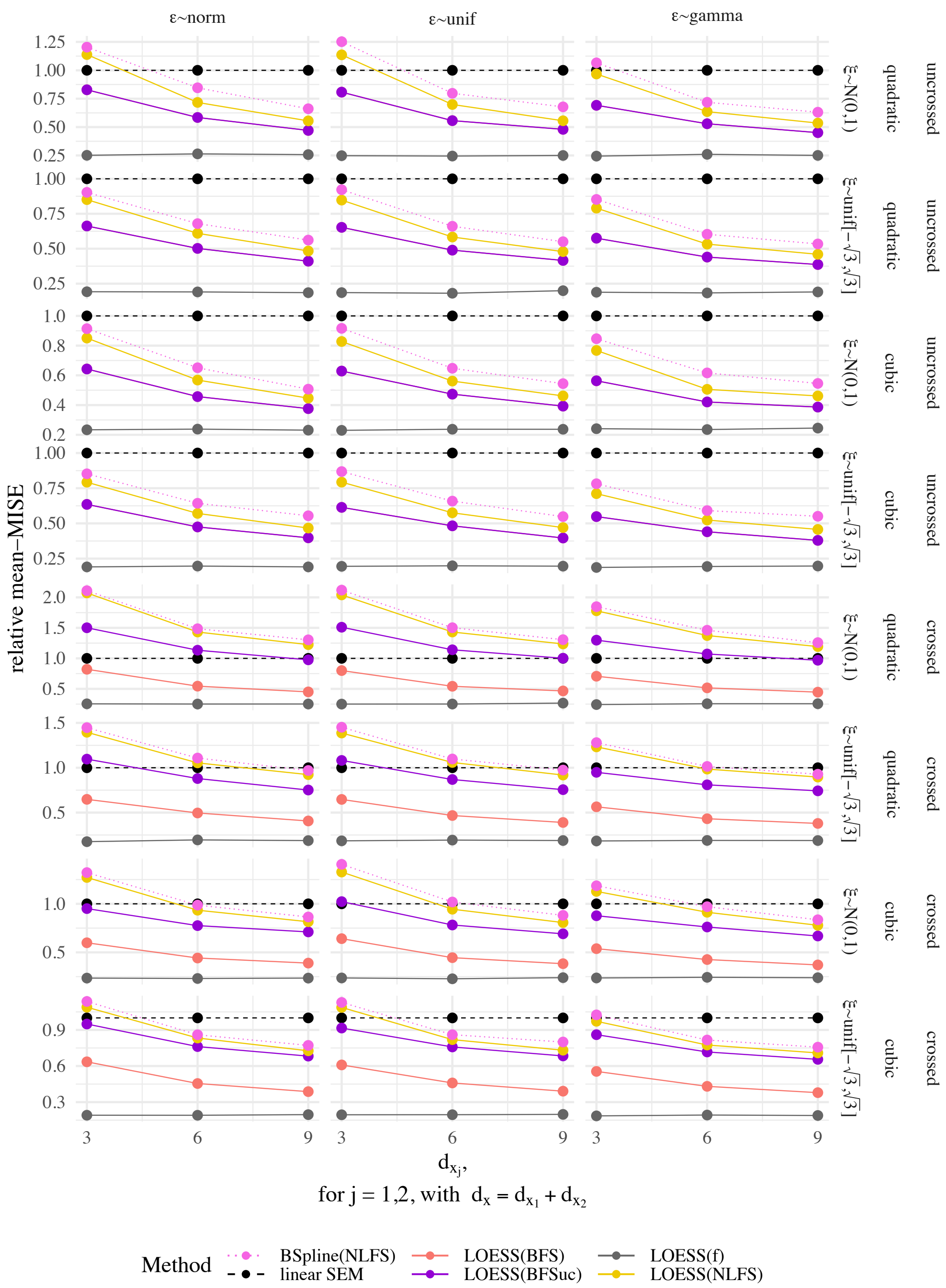


FIGURE 15. *A comparison of the relative averaged MISE compared to the linear SEM approximation across 200 replications with* $n = 1000$ *for different procedures [(B)Splines vs. LOESS vs. HZ/others] based on different inputs (BFS, NLFS, linear SEM, and true latent variables* $f$ *for comparison) for two models with different true trends (quadratic and cubic), dimensions* $d_{x_j}$*, and inclusion of cross-relations (cross-loadings and cross-correlations in* $\Lambda_x, \Psi_x$*, and* $\Psi_y$*) and distributions (row and column names refer to marginal distributions) used in the simulation study for* $d_\xi = 2$*. See Table 14 and 15 for numerical values.*

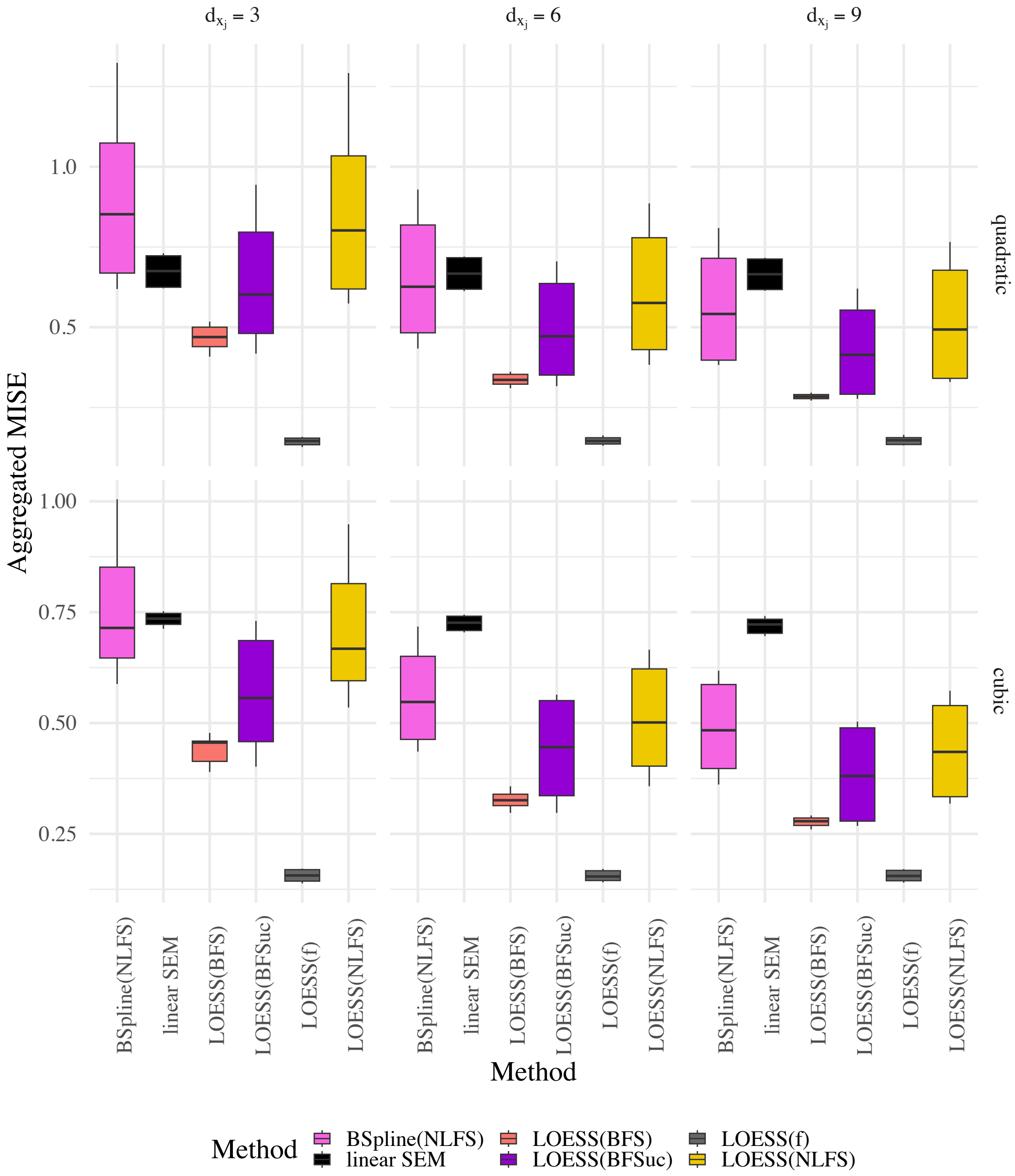


FIGURE 16. *A comparison of the averaged MISE across 200 replications with* $n = 1000$ *for different procedures [(B)Splines vs. LOESS vs. HZ/others] based on different inputs (BFS, NLFS, linear SEM, and true latent variables* $f$ *for comparison) for two models with different true trends (quadratic and cubic) and dimensions* $d_{x_j}$ *for* $d_\xi = 2$ *aggregated across all distributions and and inclusion of cross-relations (cross-loadings and cross-correlations in* $\Lambda_x, \Psi_x$*, and* $\Psi_y$*) used in the simulation study described in Section 4.4.*

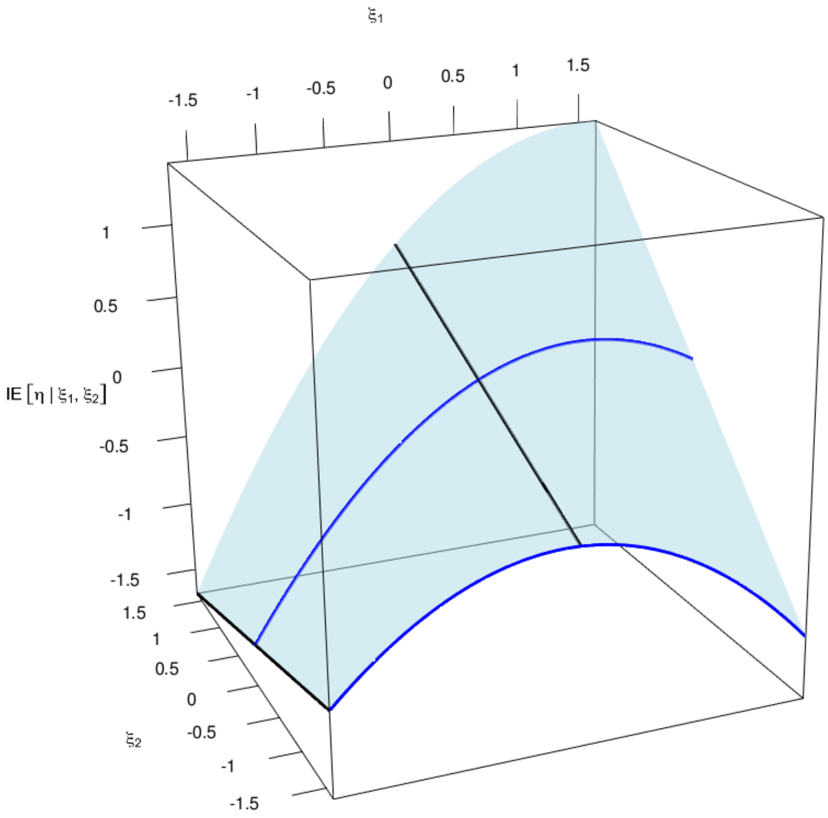


FIGURE 17. *True quadratic trend of used in the simulation study. black lines and blue lines indicate the specific marginal relationships between $H$ and $\xi$ further depicted in Figures 19, and 20.*

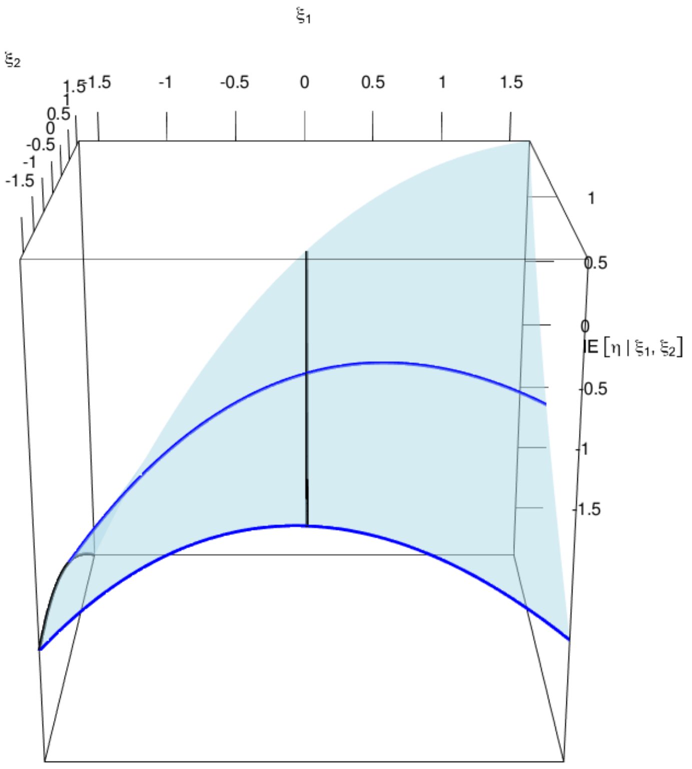


Figure 18. *True cubic trend of used in the simulation study. black lines and blue lines indicate the specific marginal relationships between $H$ and $\xi$ further depicted in Figures 19, and 20.*

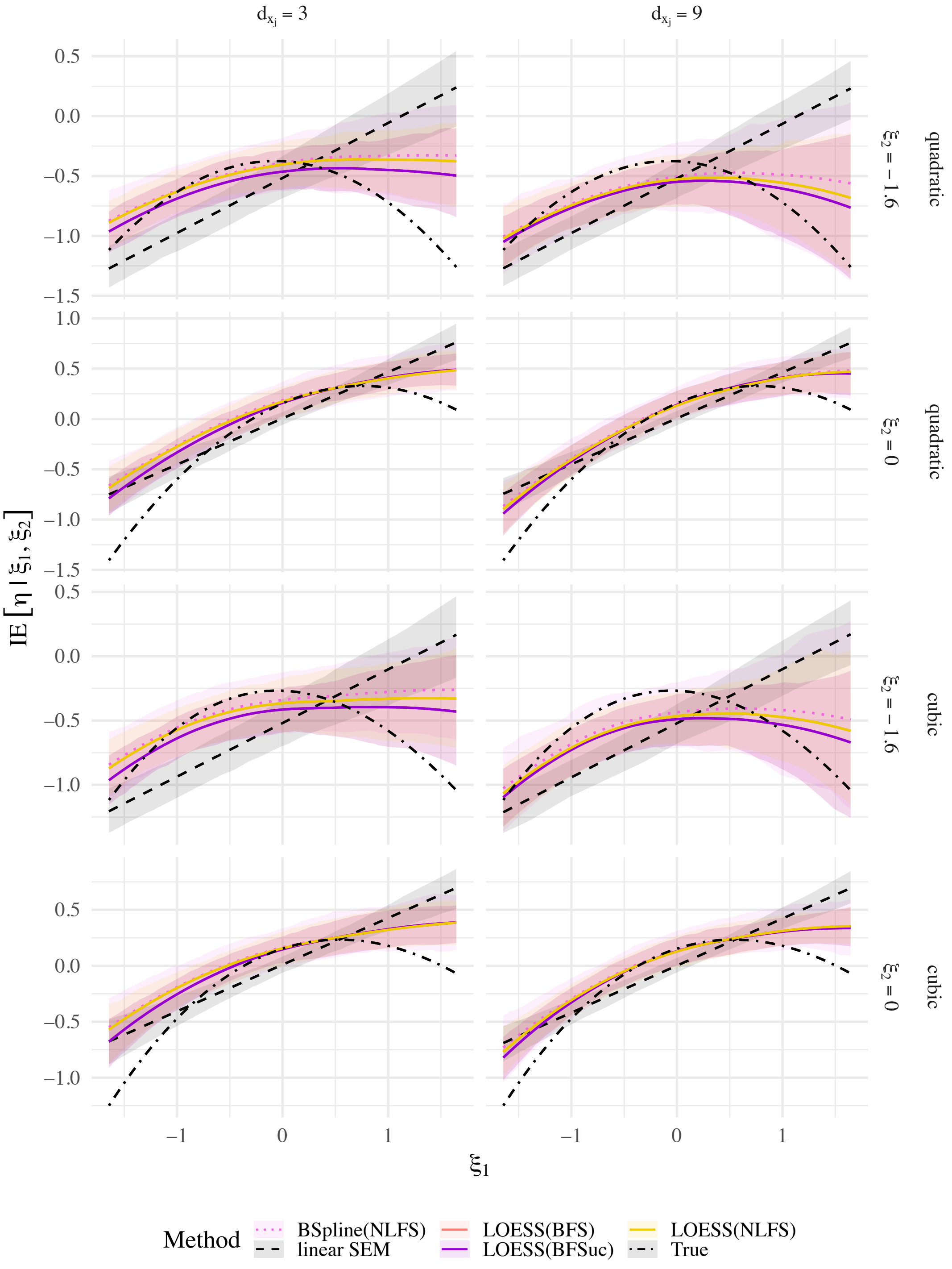


FIGURE 19. *A comparison of nonparametric estimation for* $\mathbb{E}[\eta|\xi]$ *averaged across 200 replications with* $n = 1000$ *for LOESS based on BFS and the NLFS, the BSpline estimator based on NLFS compared to the true trend and a linear SEM estimation with different true trends (quadratic, cubic) and dimensions* $d_{x_j}$ *with multivariate normal* $\xi$ *and gamma distributed errors* $\varepsilon$ *and measurements without cross-relations for specific values of* $\xi_2$*. Shaded areas correspond to the 95% coverage interval computed point-wise across the 200 replications.*

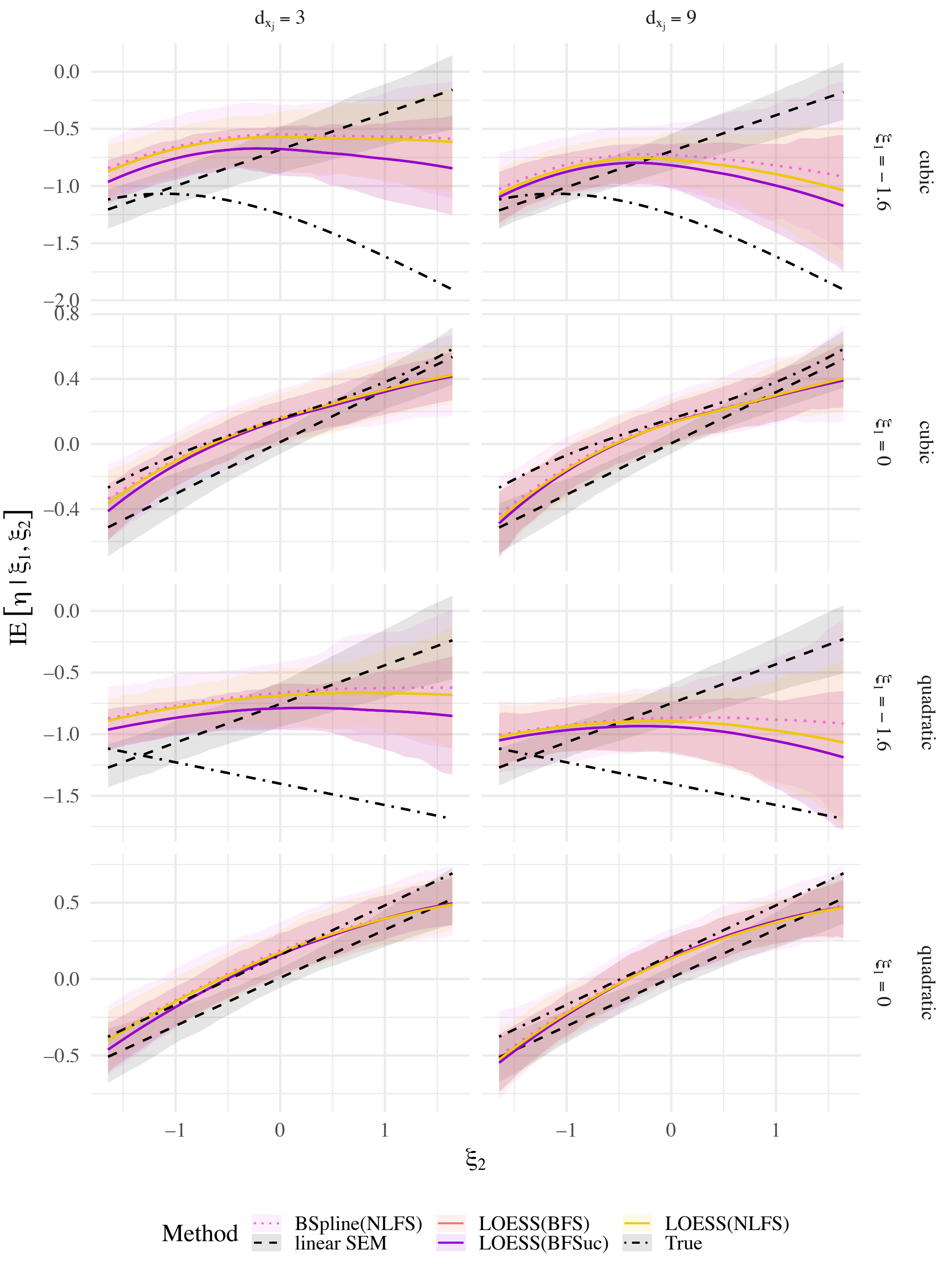


FIGURE 20. *A comparison of nonparametric estimation for* $\mathbb{E}[\eta|\xi]$ *averaged across 200 replications with* $n = 1000$ *for LOESS based on BFS and the NLFS, the BSpline estimator based on NLFS compared to the true trend and a linear SEM estimation with different true trends (quadratic, cubic) and dimensions* $d_{x_j}$ *with multivariate normal* $\xi$ *and gamma distributed errors* $\varepsilon$ *and measurements without cross-relations for specific values of* $\xi_1$. *Shaded areas correspond to the 95% coverage interval computed point-wise across the 200 replications.*

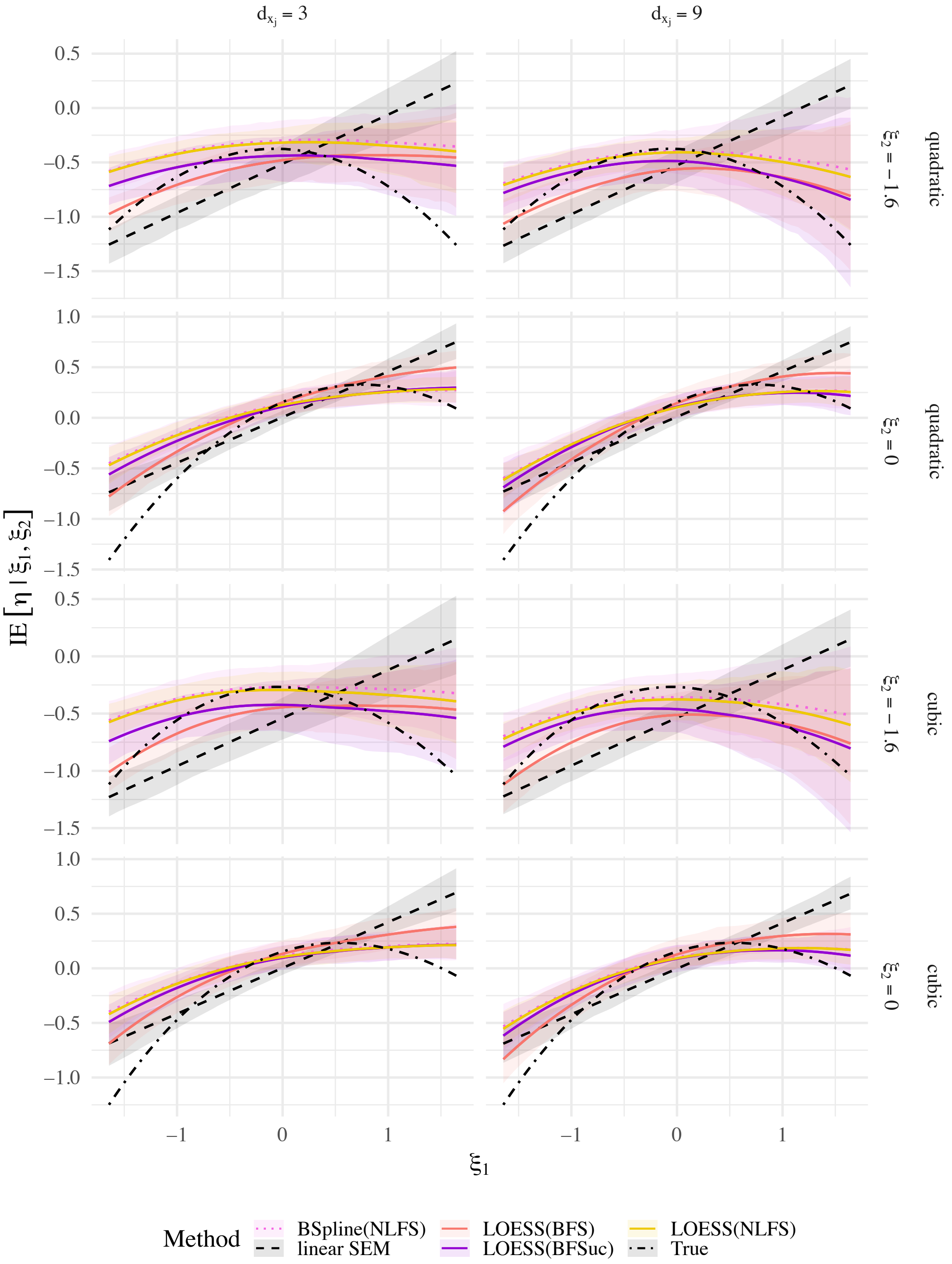


FIGURE 21. *A comparison of nonparametric estimation for $\mathbb{E}[\eta|\xi]$ averaged across 200 replications with $n = 1000$ for LOESS based on BFS and the NLFS, the BSpline estimator based on NLFS compared to the true trend and a linear SEM estimation with different true trends (quadratic, cubic) and dimensions $d_{x_j}$ with multivariate normal $\xi$ and gamma distributed errors $\varepsilon$ and measurements with cross-relations for specific values of $\xi_2$. Shaded areas correspond to the 95% coverage interval computed point-wise across the 200 replications.*

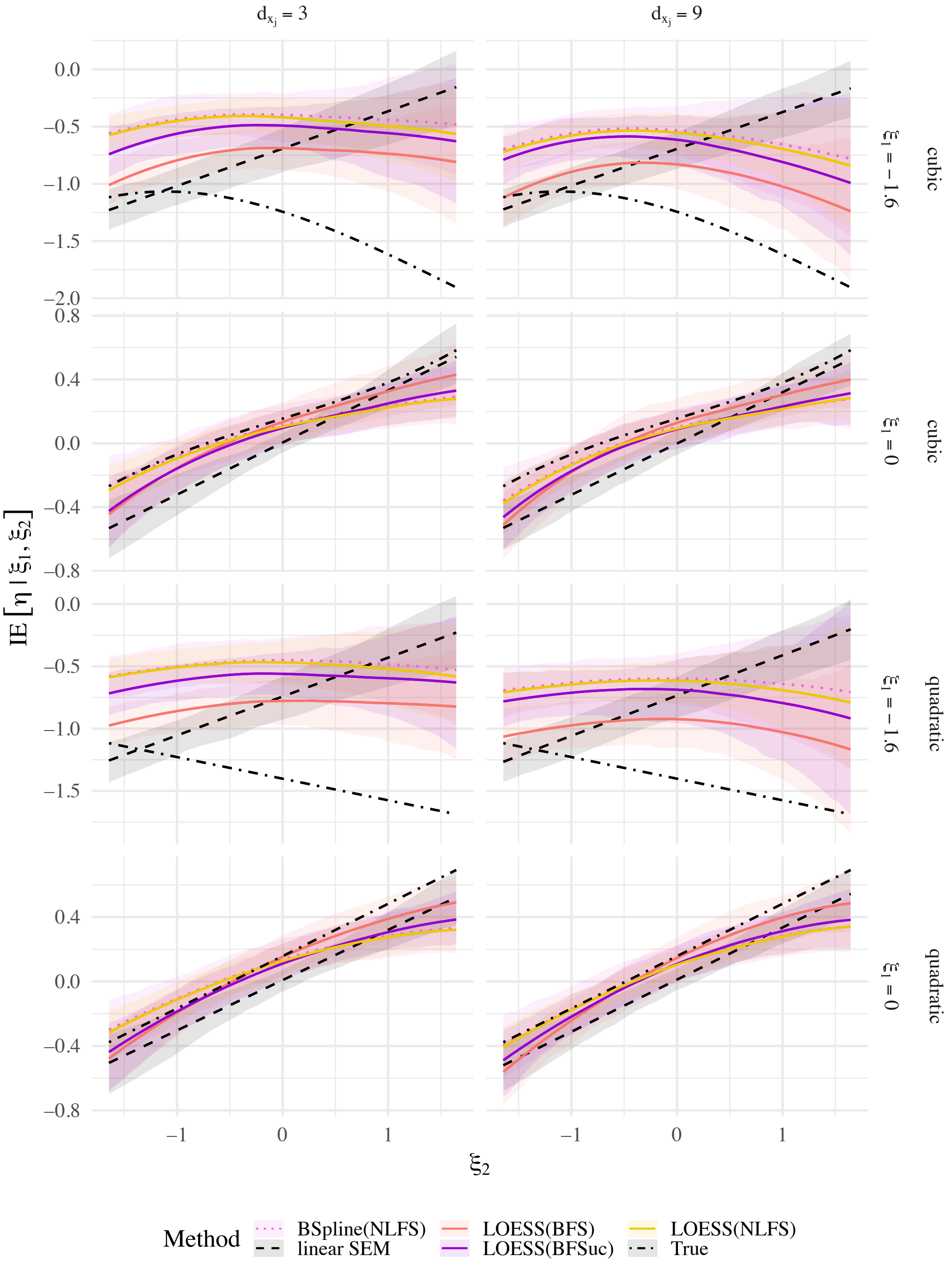


FIGURE 22. *A comparison of nonparametric estimation for* $\mathbb{E}[\eta|\xi]$ *averaged across 200 replications with* $n = 1000$ *for LOESS based on BFS and the NLFS, the BSpline estimator based on NLFS compared to the true trend and a linear SEM estimation with different true trends (quadratic, cubic) and dimensions* $d_{x_j}$ *with multivariate normal* $\xi$ *and gamma distributed errors* $\varepsilon$ *and measurements with cross-relations for specific values of* $\xi_1$*. Shaded areas correspond to the 95% coverage interval computed point-wise across the 200 replications.*

## Appendix E. Technical and Mathematical Appendix

E.1. **On Assumption 1.** The assumption of at least two finite moments firstly implies that $\mathbb{E}[\eta|\xi]$ exists, and secondly that the mentioned covariances are finite (by the Cauchy-Schwartz inequality).

Assumption 1 (1) is a minimum requirement for $\tilde{z}$ to be said to follow a factor model, as otherwise the covariance structure is misspecified. Let $\mathcal{G}(\Lambda)$ be the set of matrices which fulfill $A\Lambda = I_{d_f}$, i.e., the left inverses of $\Lambda$. Since a matrix has left inverses if and only if it has full column rank (Harville, 1997, Lemma 8.1.1), $\mathcal{G}(\Lambda)$ is non-empty if and only if $\Lambda$ has full column rank. Therefore, Assumption 1 (2) is foundational. Assumption 1 (3) means that no linear combinations of $f$ has zero variance, which would mean that the dimensionality of $f$ is misspecified. Assumption 1 (4) is also foundational. Suppose (4) does not hold. Since $\Psi$ is a covariance matrix, this means that some of its non-negative eigenvalues are zero. $\Psi$ is diagonalizable with $\Psi = PDP'$ for a diagonal matrix $D$ with real and ordered eigenvalues, and $P$ a $d_z \times d_z$ orthonormal matrix. Therefore, let $\tilde{\varepsilon} = P'\varepsilon$, whose last coordinates are zero, so that $\mathrm{Cov}\,(\tilde{\varepsilon}) = P'\Psi P = D$, and $P'\tilde{z} = P\Lambda f + \tilde{\varepsilon}$ follows a factor model whose last coordinates have no measurement error. Assumption 1 (4) disallows this, which under parameter identification would mean there is no need for factor scores.

E.2. **A Discussion on Assumption 7 (3) (a).** Recall that Assumption 7 (3) (a) is that $\sup_{x\in\mathcal{S}^\rho} |\mathbb{E}\omega(x, r_\xi)| \to 0$ as $d_x \to \infty$, where $\omega(x, h) = H(x - h) - H(x)$. We here verify this assumption in the simple class of functions $H$ that are univariate polynomials, assuming the strong assumptions as in Section 2.2. Extensions of this argument can be developed, but we consider this verification mainly an illustration.

Let us start getting familiar with this assumption in some special cases for real valued coefficients $a_i$, for $i \geq 0$. Suppose $H(x) = a_0 + a_1 x$ is linear. Then $H(x-r_\xi) - H(x) = -a_1 r_\xi$ and so $\mathbb{E}H(x-r_\xi) - H(x) = -a_1\mathbb{E}r_\xi = 0$. Suppose then that $H(x) = a_0 + a_1 x + a_2 x^2$ is a second degree polynomial. Then $H(x-r_\xi) - H(x) = -a_1 r_\xi + a_2[(x-r_\xi)^2 - x^2] = -a_1 r_\xi + a_2(-2xr_\xi + r_\xi^2)$, so that $\mathbb{E}\left[H(x - r_\xi) - H(x)\right] = a_2\left(-2x\mathbb{E}r_\xi + \mathbb{E}r_\xi^2\right) = a_2 \,\mathrm{Var}\, r_\xi$, which goes to zero e.g. under the conditions of Proposition 3.

For both of these cases, the convergence holds irrespective of the size of $\mathcal{S}^\rho$, which will not be the case in general. Indeed, let us consider a third order polynomial. Let

$$H_p(x) = \sum_{i=0}^{p} a_i x_i^p, \qquad \text{with } a_p \neq 0. \tag{10}$$

Then $H_3(x - r_\xi) - H_3(x) = H_2(x - r_\xi) - H_2(x) + a_3(x - r_\xi)^3 - a_3 x^3 = H_2(x - r_\xi) - H_2(x) + a_3\left(-3x^2 r_\xi + 3x r_\xi^2 - r_\xi^3\right)$ with expectation $(a_2 + a_3 3x)\,\mathrm{Var}\, r_\xi + a_3\mathbb{E}r_\xi^3$. Due to the inclusion of $x$, we cannot have that $\sup_{x\in\mathcal{S}^\rho} |\mathbb{E}\omega(x, r_\xi)| \to 0$ if $\mathcal{S}^\rho$ has infinite extension. For general functions, we will therefore assume that $\mathcal{S}^\rho$ has a finite extension, and we see from the third order case that this cannot be weakened.

Since $\eta, \xi$ has all practically relevant realizations within a region of finite extension, assuming that $\mathcal{S}^\rho$ has finite extension will not matter in practical applications, especially with finite sample settings. A different proof technique could give a requirement where this is not needed, and this is considered outside the scope of the present paper.

To finish the argument in the the third order polynomial case, if $s = |\sup\{x \in \mathcal{S}^\rho\}|$, then using the triangle inequality, we have $\sup_{x\in\mathcal{S}^\rho} |\mathbb{E}\omega(x, r_\xi)| = \sup_{x\in\mathcal{S}^\rho} |(a_2 + a_3 3x)\,\mathrm{Var}\, r_\xi + a_3\mathbb{E}r_\xi^3| \leq (|a_2| + |a_3|3s)\,\mathrm{Var}\, r_\xi + |a_3||\mathbb{E}r_\xi^3| \to 0$ where $\mathbb{E}r_\xi^3 \to 0$ follows by the upcoming Lemma 5.

We now consider the general polynomial case.

*Assumption* 10. Suppose

(1) $d_\eta = d_\xi = 1$.
(2) for a $p \geq 1$ we have

$$\sup_{j\geq 1} \mathbb{E}\frac{|\varepsilon_j|^p}{\sqrt{\psi_{jj}}} < \infty.$$

(3) Suppose $\mathcal{S}^\rho$ from Assumption 7 (3) has finite extension, that is there is a number $M_{\mathcal{S}^\rho} > 0$ such that $\mathcal{S}^\rho \subseteq [-M_{\mathcal{S}^\rho}, M_{\mathcal{S}^\rho}]$.
(4) Suppose $H$ is a polynomial of degree $p$, where $p$ is the constant from (2) above.

**Proposition 6.** *Suppose given Assumption 1, 8, 9 (2) and (3), and 10. Then* $\sup_{x\in\mathcal{S}^\rho} |\mathbb{E}\omega(x, r_\xi)| \to 0$ *as* $d_x \to \infty$.

*Proof.* We have $H = H_p$ as in eq. (10). Suppose $p \geq 2$, as $p = 1$ follows as above. We have

$$\begin{aligned}
\mathbb{E}H_p(x - r_\xi) - H_p(x) &= \mathbb{E}\sum_{i=0}^{p} a_i(x - r_\xi)^i - \sum_{i=0}^{p} a_i x^i \\
&= \mathbb{E}\sum_{i=0}^{p} a_i[(x - r_\xi)^i - x^i] \\
&\overset{(a)}{=} \sum_{i=2}^{p} a_i \mathbb{E}[(x - r_\xi)^i - x^i] \\
&= \sum_{i=2}^{p} a_i \mathbb{E}\left[\left(\sum_{j=0}^{i}\binom{i}{j} x^{i-j}(-1)^j \mathbb{E}r_\xi^j\right) - x^i\right] \\
&\overset{(b)}{=} \sum_{i=2}^{p} a_i \mathbb{E}\sum_{j=1}^{i}\binom{i}{j} x^{i-j}(-1)^j \mathbb{E}r_\xi^j \\
&\overset{(c)}{=} \sum_{i=2}^{p} a_i \sum_{j=2}^{i}\binom{i}{j} x^{i-j}(-1)^j \mathbb{E}r_\xi^j
\end{aligned}$$

*(a)* For $i = 0$ we have $(x - r_\xi)^i - x^i = 0$, so the $i = 0$ term vanishes. Also, for $i = 1$, we get $\mathbb{E}(x - r_\xi)^i - x^i = x - \mathbb{E}r_\xi - x = 0$. Therefore, only terms with $i \geq 2$ are relevant. *(b)* For $j = 0$ we have $\binom{i}{j}x^{i-j}(-1)^j \mathbb{E}r_\xi^j = x^i$, which cancels by the term $-x^i$. *(c)* If $j = 1$ then $\mathbb{E}r_\xi^j = 0$, and hence this term vanishes.

Therefore, by Assumption 10 (3) and the triangle inequality, we have

$$\begin{aligned}
\sup_{x\in\mathcal{S}^\rho} |\mathbb{E}H_p(x - r_\xi) - H_p(x)| &\leq \sup_{|x|\leq M_A} |\mathbb{E}H_p(x - r_\xi) - H_p(x)| \\
&= \sup_{|x|\leq M_A} \sum_{i=2}^{p} a_i \sum_{j=2}^{i}\binom{i}{j} |x^{i-j}||\mathbb{E}r_\xi^j| \\
&\leq \sum_{i=2}^{p} a_i \sum_{j=2}^{i}\binom{i}{j} M_A^{i-j}\mathbb{E}|r_\xi|^j
\end{aligned}$$

Since $\mathbb{E}|r_\xi|^i \to 0$ for $2 \leq i \leq p$ by the forthcoming Lemma 5, we get the desired convergence, as the number of terms in the sum is fixed as $d_x$ increase. $\square$

**Lemma 5.** *Suppose given Assumption 1, 8, 9 (2) and (3), and 10. Then* $\mathbb{E}|r_\xi|^q \to 0$ *for any integer* $2 \le q \le p$.

*Proof.* Notice that from Assumption 8 (1), $\varepsilon_x$ has independent components. This independence will be crucial for the result.

By the Lyapunov inequality, we have $\mathbb{E}|r_\xi|^q \le (\mathbb{E}|r_\xi|^p)^{q/p}$. Therefore, $\mathbb{E}|r_\xi|^q \to 0$ for $2 \le q \le p$ as long as $\mathbb{E}|r_\xi|^p \to 0$, which is what we show.

We use one of the several inequalities that carry the name "the Marczinkiewicz-Zygmund inequality", see (Révész, 1967, Theorem 2.1.3) and the more recent refinement in Ren and Liang (2001) which gives the soon to be stated bound for the soon mentioned constant $\mathcal{C}$. It says that for independent $X_1, \ldots$ with zero mean and $\sup_{i\ge1}\mathbb{E}|X_i|^p < \infty$ for $p \ge 2$ we have that that for a constant $\mathcal{C} \le (3\sqrt{2})^p p^{p/2}$ we have

$$\mathbb{E}\left|\sum_{i=1}^n X_i\right|^p \le \mathcal{C}n^{(p/2)-1}\sum_{i=1}^n \mathbb{E}|X_i|^p. \tag{11}$$

As in the proof of Proposition 5, see eq. (17) (p. A57), we get from Assumption 8 that

$$r_\xi = \frac{1}{n_{d_x}}\sum_{i=1}^{d_x}\sqrt{\alpha_j}u_j$$

where $u_j = \frac{\varepsilon_j}{\sqrt{\psi_{jj}}}$ $\alpha_j = \frac{\lambda_{ji}^2}{\psi_{jj}}$ and $n_{d_x} = \sum_{j=1}^{d_x}\alpha_j$. Notice $\alpha_j \ge 0$.

This gives

$$\begin{aligned}
\mathbb{E}|r_\xi|^p &= n_{d_x}^{-p}\mathbb{E}\left|\sum_{i=1}^{d_x}\sqrt{\alpha_j}u_j\right|^p \\
&\le \mathcal{C}n_{d_x}^{-p}d_x^{(p/2)-1}\sum_{i=1}^{d_x}\mathbb{E}|\sqrt{\alpha_j}u_j|^p \\
&\le \mathcal{C}n_{d_x}^{-p}d_x^{(p/2)-1}\sum_{i=1}^{d_x}\sqrt{\alpha_j}^p\left(\sup_{j\ge1}\mathbb{E}\frac{|\varepsilon_j|^p}{\sqrt{\psi_{jj}}}\right) \\
&= \left(\sup_{j\ge1}\mathbb{E}\frac{|\varepsilon_j|^p}{\sqrt{\psi_{jj}}}\right)\mathcal{C}n_{d_x}^{-p}d_x^{(p/2)-1}\sum_{i=1}^{d_x}\sqrt{\alpha_j}^p.
\end{aligned}$$

We now use Assumption 9 (2), i.e., that $\left(\frac{\lambda_{j1}^2}{\psi_{jj}}\right)_{1\le j\le d_x} \subset [m_{\lambda/\psi}, M_{\lambda/\psi}]$ for numbers $0 < m_{\lambda/\psi} \le M_{\lambda/\psi} < \infty$. This gives

$$n_{d_x} = \sum_{j=1}^{d_x}\alpha_j \ge \sum_{j=1}^{d_x} m_{\lambda/\psi} = d_x m_{\lambda/\psi}$$

and

$$\sum_{i=1}^{d_x}\sqrt{\alpha_j}^p \le \sum_{i=1}^{d_x}\sqrt{M_{\lambda/\psi}}^p = d_x\sqrt{M_{\lambda/\psi}}^p.$$

Inserting this in the series of inequalities from above gives

$$\begin{aligned}
\mathbb{E}|r_\xi|^q &\le \left(\sup_{j\ge1}\mathbb{E}\frac{|\varepsilon_j|^p}{\sqrt{\psi_{jj}}}\right)\mathcal{C}m_{\lambda/\psi}^{-p}d_x^{-p}d_x^{(p/2)-1}\sqrt{M_{\lambda/\psi}}^p d_x \\
&= \left(\sup_{j\ge1}\mathbb{E}\frac{|\varepsilon_j|^p}{\sqrt{\psi_{jj}}}\right)\mathcal{C}m_{\lambda/\psi}^{-p}\sqrt{M_{\lambda/\psi}}^p d_x^{-p/2}
\end{aligned}$$

Since $p > 0$ we have $-p/2 < 0$ and so the convergence is shown as $d_x^{-p/2} \to 0$ for $d_x \to \infty$, $\sup_{j\geq 1} \mathbb{E}\frac{|\varepsilon_j|^p}{\sqrt{\psi_{jj}}}$ was assumed to be finite, and the remaining are finite constants. □

E.3. **More Details on the Consequences of Asymptotic Normality of $r_\xi$ Following Proposition 5.** Using our notation as well as the the conclusion from Lemma 2 that $\mathbb{E}[\ddot{\eta}|\xi = x] = H(x)$, Huang and Zhou (2017) is based on the equality

$$H(x) = \frac{1}{2\pi f_{\ddot{\xi}}(x)} \int e^{-itx} \frac{\phi_{H_{d_x} f_{\ddot{\eta}}}(t)}{\phi_{r_\xi}(t)} dt$$

where $H_{d_x}(x) = \mathbb{E}[\ddot{\eta}|\ddot{\xi} = x]$, where $\phi_{H_{d_x} f_{\ddot{\eta}}}$ is the characteristic function of the convolution between $H_{d_x}$ and $f_{\ddot{\eta}}$, and where $\phi_{r_\xi}$ is the characteristic function of $r_\xi$. Except $\phi_{r_\xi}$, all quantities in the above display are identified. Proposition 5 motivates approximating $\phi_{r_\xi}$ by the characteristic function of a re-scaled normal random vector, which has a known formula. To simplify notation, consider the special case $d_\eta = d_\xi = 1$. Then the suggested approximation is

$$\breve{H}(x) = \frac{1}{2\pi f_{\ddot{\xi}}(x)} \int e^{-itx} \frac{\phi_{H_{d_x} f_{\ddot{\eta}}}(t)}{\phi_{Z/c_{d_x}}(t)} dt, \quad Z \sim N(0, 1),$$

which is the population version of the Huang and Zhou (2017) estimator when using it with the normality approximation from Proposition 5. However, replacing $r_\xi$ with zero is shown to yield uniformly consistent approximations of $H$ as $d_x$ increase in Proposition 4, and so merely getting this from the normal approximation does seem needed, as the asymptotic normality also implies that $r_\xi$ converges to zero in probability, thereby fulfilling Assumption 7 (4), which means that such a result would not give new insight. Therefore, the possible benefits of the normality approximation would be not in terms of asymptotic identification, but if $\breve{H}$ was better than $H_{d_x}$ of Proposition 4 as an approximation to $H$. This seems complex to investigate mathematically, especially since $Z/c_{d_x}$ goes to zero for $d_x$ increasing, and is considered outside the scope of the present paper.

E.4. **Mathematical Results and Proofs.**

E.4.1. *Proof of Lemma 1.*

*Proof of Lemma 1.* Statement 1: We have $\text{Cov}(f, r_A) = \text{Cov}(f, A\tilde{z} - f) = \text{Cov}(f, A\tilde{z}) - \text{Cov}(f) = \text{Cov}(f, A\Lambda f + A\varepsilon) - \text{Cov}(f) = A\Lambda\,\text{Cov}(f) - \text{Cov}(f) + A\,\text{Cov}(f, \varepsilon) = (A\Lambda - I_{d_f})\,\text{Cov}(f) = (A\Lambda - I_{d_f})\Phi$.

Suppose $A \in \mathcal{G}(\Lambda)$. Then $A\Lambda - I_{d_f} = 0$, so that $\text{Cov}(f, r_A) = 0\Phi = 0$. Suppose $\text{Cov}(f, r_A) = 0$. Then $0 = (A\Lambda - I_{d_f})\,\text{Cov}(f)$ so that right multiplying both sides of the equality by $\Phi^{-1}$ gives $0 = A\Lambda - I_{d_f}$, and so $A \in \mathcal{G}(\Lambda)$.

Statement 2: This follows from $\mathbb{E}[A\tilde{z}|f] = \mathbb{E}[A(\Lambda f + \varepsilon)|f] = A\Lambda\mathbb{E}[f|f] + A\mathbb{E}[\varepsilon|f] = A\Lambda f$, which equals $f$ if and only if A is a left inverse of $\Lambda$, i.e., $A \in \mathcal{G}(\Lambda)$.

Statement 3: We first show that $T$ exists. This is implied from that $\Sigma = \text{Cov}\ z = \Lambda\Phi\Lambda' + \Psi$ is invertible under Assumption 1 (3) and (4), as we now show. We will do this by showing that $\Sigma$ is positive definite. Let $x$ be a non-zero $d_z$ dimensional vector. Since $\Psi$ is positive definite by Assumption 1 (4), $x'\Psi x > 0$. We have $x'\Sigma x = x'\Lambda\Phi\Lambda' x + x'\Psi x = y'\Phi y + x'\Psi x$ where $y = \Lambda x$. If $y = 0$, then $x'\Sigma x = x'\Psi x > 0$. If $y \neq 0$, then also $y'\Phi y > 0$ since $\Phi$ is positive definite by Assumption 1 (3). Therefore, $\Sigma$ is positive definite and, hence, invertible.

Let $T = \text{Cov}(f)\Lambda'\,\text{Cov}(z)^{-1} = \Phi\Lambda'(\Lambda\Phi\Lambda' + \Psi)^{-1}$. Now we show that $T \notin \mathcal{G}(\Lambda)$ by contradiction: Assume that $T \in \mathcal{G}(\Lambda)$. That is, $T\Lambda = I_{d_f}$. By Lemma 13 (p. A77), we can also write $T = \left(\Phi^{-1} + \Lambda'\Psi^{-1}\Lambda\right)^{-1}\Lambda'\Psi^{-1}$. Now, we have by assumption that $T\Lambda = \left(\Phi^{-1} + \Lambda'\Psi^{-1}\Lambda\right)^{-1}\Lambda'\Psi^{-1}\Lambda = I_{d_f}$. Right multiplying on both sides with $\Phi^{-1} + \Lambda'\Psi^{-1}\Lambda$ gives $\Lambda'\Psi^{-1}\Lambda = \Phi^{-1} + \Lambda'\Psi^{-1}\Lambda$ which holds

if and only if $\Phi^{-1} = 0$, which is not the case, because $\Phi^{-1}$ is positive definite since $\Phi$ is positive definite by Assumption 1 (3). This is a contradiction, so it follows that $T \notin \mathcal{G}(\Lambda)$.

Statement 4: We first show that $\Delta$ exists, which holds if $\Lambda'\Psi^{-1}\Lambda$ is invertible. We show that $\Lambda'\Psi^{-1}\Lambda$ has the same rank as $\Psi^{-1}$. This implies that $\Lambda'\Psi^{-1}\Lambda$ is invertible, because it has the same dimensionality as $\Psi^{-1}$, which is an invertible matrix by Assumption 1 (4).

Recall (Harville, 1997, Lemma 8.3.2) that for two matrices $A, B$ of compatible dimensions, we have that $\text{rank}(AB) = \text{rank}(B)$ if $A$ has full column rank, and that $\text{rank}(AB) = \text{rank}(A)$ if $B$ has full row rank.

Since $\Psi$ has full rank, since it is positive definite, $\Psi^{-1}$ has full row and full column rank and this gives $\text{rank}(\Psi^{-1}\Lambda) = \text{rank}(\Lambda)$. Since $\Lambda$ has full column rank, $\Lambda'$ has full row rank. Therefore, $\text{rank}(\Lambda'\Psi^{-1}\Lambda) = \text{rank}(\Psi^{-1}\Lambda) = \text{rank}(\Lambda)$. The rank of $\Lambda$ is $d_f$ as it has full column rank and since $d_f < d_z$. Since $\Lambda'\Psi^{-1}\Lambda$ is a $d_f \times d_f$ matrix, it has full rank and, therefore, is invertible. Hence, the Bartlett matrix $\Delta$ exists.

We have that $\Delta$ is in $\mathcal{G}(\Lambda)$ because $\Delta\Lambda = (\Lambda'\Psi^{-1}\Lambda)^{-1}\Lambda'\Psi^{-1}\Lambda = I_{d_f}$. The optimality property follows from standard theory on GLS, see e.g. (Hansen, 2022, Chapter 4.6).

□

E.4.2. *Proof of Lemma 2.*

*Proof of Lemma 2.* By eq. (5), by the displayed above and the linearity of conditional expectations, we have $\mathbb{E}[\ddot{\eta}|\xi] = \mathbb{E}[\eta|\xi] + \mathbb{E}[r_\eta|\xi]$. Let $\mathbf{0}_{p,q}$ be the $p \times q$ matrix of zeros and $I_p$ be the $p \times p$ identity matrix, we have $r_\eta = (\mathbf{0}_{d_\eta,d_\xi}, I_{d_\eta})A\varepsilon$. Therefore, we have $\mathbb{E}[r_\eta|\xi] = (\mathbf{0}_{d_\eta,d_\xi}, I_{d_\eta})A\mathbb{E}[\varepsilon|\xi]$. By Assumption Assumption 1 (1) and Assumption 2, $\varepsilon$ has zero mean and is independent to $f = (\xi', \eta')'$. It is therefore also independent to $\xi$. Therefore, $\mathbb{E}[\varepsilon|\xi] = \mathbb{E}\varepsilon = \mathbf{0}$ and $\mathbb{E}[\ddot{\eta}|\xi] = \mathbb{E}[\eta|\xi]$. □

E.4.3. *Proof of Proposition 1.*

*Proof of Proposition 1.* For concreteness, let us choose to work with $A = \Delta$, the Bartlett factor matrix which under Assumption 1 exists using Lemma 1 (4), and form $\ddot{f} = (\ddot{\xi}', \ddot{\eta}')'$. Consider the characteristic function of $(\ddot{\xi}', \ddot{\eta}')'$, which we recall uniquely characterizes its joint distribution. For a vector $t = (t_\xi', t_\eta')'$ of dimension $d_f$ and component dimensions $d_\xi, d_\eta$, we have

$$\mathbb{E}e^{it'(\ddot{\xi}',\ddot{\eta}')'} = \mathbb{E}e^{it_\xi'\ddot{\xi}+it_\eta'\ddot{\eta}} = \mathbb{E}e^{it_\xi'(\xi+r_\xi)+it_\eta'\ddot{\eta}} = \mathbb{E}e^{it_\xi' r_\xi}e^{it_\xi'\xi+it_\eta'\ddot{\eta}}.$$

From Assumption 2, $\varepsilon$ is independent to $f$. Therefore, $r = A\varepsilon$ is also independent to $f$. Since $r_\xi$ is just the first $d_\xi$ coordinates of $r$, it too is independent to $f$, and hence to $\xi$ and $\eta$. By Assumption 4 (2), $r_\xi$ is also independent to $r_\eta$. Therefore, $r_\xi$ is independent to both $\xi$ and $\ddot{\eta} = \eta + r_\eta$ (since $\ddot{\eta}$ is a function of $\eta$ and $r_\eta$). Therefore, the expectation of the product in the above display factorizes to the product of expectations of the terms, and we get

$$\mathbb{E}e^{it_\xi'\xi+it_\eta'\ddot{\eta}} = \frac{\mathbb{E}e^{it(\ddot{\xi}',\ddot{\eta}')'}}{\mathbb{E}e^{it_\xi' r_\xi}}.$$

Since the distribution of $r_\xi$ is known by Assumption 4 (1), and $\ddot{f} = A\tilde{z}$ has a distribution given by $A$ and the distribution of $\tilde{z}$, which is identified by Assumption 3, this shows that the distribution of $(\xi, \ddot{\eta})$ is identified. From this distribution, we may compute $\mathbb{E}[\ddot{\eta}|\xi = x]$ which from Lemma 2 equals $\mathbb{E}[\eta|\xi = x] = H(x)$, which is therefore identified. □

E.4.4. *Proof of Lemma 3.*

*Proof of Lemma 3.* As in eq. (6), we have

$$A\tilde{z} = \begin{pmatrix} A_x\Lambda_x\xi + A_x\varepsilon_x \\ A_y\Lambda_y\eta + A_y\varepsilon_y \end{pmatrix} = \begin{pmatrix} \xi \\ \eta \end{pmatrix} + \begin{pmatrix} A_x\varepsilon_x \\ A_y\varepsilon_y \end{pmatrix} = f + \begin{pmatrix} r_\xi \\ r_\eta \end{pmatrix}$$

by the assumed $A_x \in \mathcal{G}(\Lambda_x)$ and $A_y \in \mathcal{G}(\Lambda_y)$ for $r_\xi = A_x\varepsilon_x$ and $r_\eta = A_y\varepsilon_y$. Since we assume that $\varepsilon_x$ and $\varepsilon_y$ are independent, we have that also $r_\xi$ and $r_\eta$ are independent, as they are functions of only $\varepsilon_x$ and $\varepsilon_y$, respectively. □

E.4.5. *Proof of Proposition 2.*

*Proof of Proposition 2.* Using partition matrix rules, we have

$$\Psi^{-1} = \begin{pmatrix} \Psi_x^{-1} & \mathbf{0}_{d_x,d_y} \\ \mathbf{0}_{d_y,d_x} & \Psi_y^{-1} \end{pmatrix}.$$

Since

$$\begin{pmatrix} \Lambda_x' & \mathbf{0}_{d_\xi,d_y} \end{pmatrix} \begin{pmatrix} \mathbf{0}_{d_x,d_y} \\ \Psi_y^{-1} \end{pmatrix} = \mathbf{0}_{d_\xi,d_y}, \quad \text{and} \quad \begin{pmatrix} \mathbf{0}_{d_\eta,d_x} & \Lambda_y' \end{pmatrix} \begin{pmatrix} \Psi_x^{-1} \\ \mathbf{0}_{d_y,d_x} \end{pmatrix} = \mathbf{0}_{d_\eta,d_x},$$

we get

$$\Lambda'\Psi^{-1} = \begin{pmatrix} \Lambda_x' & \mathbf{0}_{d_\xi,d_y} \\ \mathbf{0}_{d_\eta,d_x} & \Lambda_y' \end{pmatrix} \begin{pmatrix} \Psi_x^{-1} & \mathbf{0}_{d_x,d_y} \\ \mathbf{0}_{d_y,d_x} & \Psi_y^{-1} \end{pmatrix} = \begin{pmatrix} \Lambda_x'\Psi_x^{-1} & \mathbf{0}_{d_\xi,d_y} \\ \mathbf{0}_{d_\eta,d_x} & \Lambda_y'\Psi_y^{-1} \end{pmatrix}.$$

Since

$$\begin{pmatrix} \Lambda_x'\Psi_x^{-1} & \mathbf{0}_{d_\xi,d_y} \end{pmatrix} \begin{pmatrix} \mathbf{0}_{d_x,d_\eta} \\ \Lambda_y \end{pmatrix} = \mathbf{0}_{d_\xi,d_\eta}, \quad \text{and} \quad \begin{pmatrix} \mathbf{0}_{d_\eta,d_x} & \Lambda_y'\Psi_y^{-1} \end{pmatrix} \begin{pmatrix} \Lambda_x \\ \mathbf{0}_{d_y,d_\xi} \end{pmatrix} = \mathbf{0}_{d_\eta,d_\xi},$$

we get

$$\Lambda'\Psi^{-1}\Lambda = \left[\Lambda'\Psi^{-1}\right]\Lambda = \begin{pmatrix} \Lambda_x'\Psi_x^{-1} & \mathbf{0}_{d_\xi,d_y} \\ \mathbf{0}_{d_\eta,d_x} & \Lambda_y'\Psi_y^{-1} \end{pmatrix} \begin{pmatrix} \Lambda_x & \mathbf{0}_{d_x,d_\eta} \\ \mathbf{0}_{d_y,d_\xi} & \Lambda_y \end{pmatrix} = \begin{pmatrix} \Lambda_x'\Psi_x^{-1}\Lambda_x & \mathbf{0}_{d_\xi,d_\eta} \\ \mathbf{0}_{d_\eta,d_\xi} & \Lambda_y'\Psi_y^{-1}\Lambda_y \end{pmatrix}.$$

Since $\Lambda_x$ has full column rank from the last statement in Assumption 5, and $\Psi_x$ is positive definite being a principle sub-matrices of a positive definite matrix $\Psi$ (Horn & Johnson, 2013, Observation 7.1.2), the matrix $\Lambda_x'\Psi_x^{-1}\Lambda_x$ is invertible by the same argument as in the proof of Statement 4 in Lemma 1 (replacing $\Lambda, \Psi$ with $\Lambda_x, \Psi_x$ respectively). The same holds for $\Lambda_y'\Psi_y^{-1}\Lambda_y$. Hence, both $\Delta_x$ and $\Delta_y$ exist.

Also, since each non-zero partition is invertible, the partitioned diagonal matrix $\Lambda'\Psi^{-1}\Lambda$ can be inverted using the partition rules, giving

$$(12) \qquad \left(\Lambda'\Psi^{-1}\Lambda\right)^{-1} = \begin{pmatrix} \left(\Lambda_x'\Psi_x^{-1}\Lambda_x\right)^{-1} & \mathbf{0}_{d_\xi,d_\eta} \\ \mathbf{0}_{d_\eta,d_\xi} & \left(\Lambda_y'\Psi_y^{-1}\Lambda_y\right)^{-1} \end{pmatrix},$$

Therefore,

$$\begin{aligned} \Delta = (\Lambda'\Psi^{-1}\Lambda)^{-1}\Lambda'\Psi^{-1} &= \begin{pmatrix} \left(\Lambda_x'\Psi_x^{-1}\Lambda_x\right)^{-1} & \mathbf{0}_{d_\xi,d_\eta} \\ \mathbf{0}_{d_\eta,d_\xi} & \left(\Lambda_y'\Psi_y^{-1}\Lambda_y\right)^{-1} \end{pmatrix} \begin{pmatrix} \Lambda_x'\Psi_x^{-1} & \mathbf{0}_{d_\xi,d_y} \\ \mathbf{0}_{d_\eta,d_x} & \Lambda_y'\Psi_y^{-1} \end{pmatrix} \\ &= \begin{pmatrix} \left(\Lambda_x'\Psi_x^{-1}\Lambda_x\right)^{-1}\Lambda_x'\Psi_x^{-1} & \mathbf{0}_{d_\xi,d_y} \\ \mathbf{0}_{d_\eta,d_x} & \left(\Lambda_y'\Psi_y^{-1}\Lambda_y\right)^{-1}\Lambda_y'\Psi_y^{-1} \end{pmatrix} \\ &= \begin{pmatrix} \Delta_x & \mathbf{0}_{d_\xi,d_y} \\ \mathbf{0}_{d_\eta,d_x} & \Delta_y \end{pmatrix}. \end{aligned}$$

As for Cov $r$, we calculate in general that

$$\begin{aligned}
\text{Cov } r &= \text{Cov } [\Delta\varepsilon] = \Delta \,\text{Cov } [\varepsilon]\, \Delta' \\
&= \left[(\Lambda'\Psi^{-1}\Lambda)^{-1}\Lambda'\Psi^{-1}\right] \Psi \left[(\Lambda'\Psi^{-1}\Lambda)^{-1}\Lambda'\Psi^{-1}\right]' \\
&= (\Lambda'\Psi^{-1}\Lambda)^{-1}\Lambda' \underbrace{\Psi^{-1}\Psi}_{=I} \left[\left(\Psi^{-1}\right)' (\Lambda')' \left[(\Lambda'\Psi^{-1}\Lambda)^{-1}\right]'\right] \\
&= \underbrace{(\Lambda'\Psi^{-1}\Lambda)^{-1}\Lambda'\Psi^{-1}\Lambda}_{=I} \left[(\Lambda'\Psi^{-1}\Lambda)'\right]^{-1} \\
&= (\Lambda'\Psi^{-1}\Lambda)^{-1},
\end{aligned}$$

which together with eq. (12) gives the stated formula. This formula also shows that Cov $r$ is positive definite, because it is invertible as shown in the proof of Statement 4 in Lemma 1. The last statement, that $\left(\Lambda_x'\Psi_x^{-1}\Lambda_x\right)^{-1}$ and $\left(\Lambda_y'\Psi_y^{-1}\Lambda_y\right)^{-1}$ are positive definite, follows since they the inverse of positive definite matrices, as shown when we above showed that $\Delta_x, \Delta_y$ exists. □

E.4.6. *Proof of Proposition 3.*

*Proof of Proposition 3.* Statement (1): We will use the following property twice: For a symmetric positive definite $m \times m$ matrix $M$, we have that $\max_{1\le i,j\le m} |M_{i,j}| \le \lambda_{\max}(M)$. Since we have not found a reference for this likely well-known result with a complete proof, we provide a proof in Lemma 12 (p. A77).

From Proposition 2 we have that Cov $r_\xi = (\Lambda_x'\Psi_x^{-1}\Lambda_x)^{-1}$, and that it is a positive definite matrix. We therefore have that

$$\max_{1\le i,j\le d_\xi} |(\text{Cov } r_\xi)_{i,j}| \le \lambda_{\max}\left((\Lambda_x'\Psi_x^{-1}\Lambda_x)^{-1}\right) = \frac{1}{\lambda_{\min}(\Lambda_x'\Psi_x^{-1}\Lambda_x)} \tag{13}$$

where the last step follows from the spectral decomposition theorem, see e.g. Corollary A.6.4.1 in Mardia et al. (1979).

We now show that

$$\lambda_{\min}(\Lambda_x'\Psi_x^{-1}\Lambda_x) \ge \min_{1\le i\le d_\xi} N_i \left(\frac{m_{\Lambda_x}^2}{M_{\Psi_x}} - \frac{M_{\Lambda_x}^2}{m_{\Psi_x}}\frac{1}{N_i}\sum_{1\le j\le d_\xi, j\neq i} C_{i,j}\right),$$

which from eq. (13) implies the conclusion of the first statement.

Now $\Lambda_x'\Psi_x^{-1}\Lambda_x$ has a constant dimension of $d_\xi \times d_\xi$, and has entries of the form

$$(\Lambda_x'\Psi_x^{-1}\Lambda_x)_{i,j} = (\Lambda_x)_{\cdot,i}'\Psi_x^{-1}(\Lambda_x)_{\cdot,j}$$

where $(\Lambda_x)_{\cdot,j}$ is the $i$'th column of $\Lambda_x$.

We make use of the Greshgorin circle theorem (Horn & Johnson, 2013, Theorem 6.1.1), which states that each eigenvalue $\lambda_M$ of a $d_m \times d_m$ square matrix $M = (m_{i,j})_{i,j}$ is contained in the complex plane $D(m_{i,i}, R_i)$ of radius $R_i = \sum_{i\neq j, 1\le j\le d_m} |m_{i,j}|$. Now in our scenario the matrix $M$ is positive definite and we know all of the $d_m$ eigenvalues are real. Hence, all $d_m$ eigenvalues are contained in the intervals of the form $D_i = [m_{i,i} - R_i, m_{i,i} + R_i]$, so that all eigenvalues are in $D = \bigcup_{1\le i\le d_m} D_i$. Since the radius $R_i \ge 0$ for all $1 \le i \le d_m$, we need to consider the smallest point $G$ within $D$, i.e., $G = \min_{d\in D} = \min_{1\le i\le d_m}(m_{i,i} - R_i)$. If now $G \to \infty$, then the eigenvalues of $M$ diverge without bound and consequently, the eigenvalues of $M^{-1}$ converge to zero. Therefore, by translation to our

notation, the smallest eigenvalue of $\Lambda_x'\Psi_x^{-1}\Lambda_x$ is greater than

$$G = \min_{1\leq i\leq d_\xi}\left((\Lambda_x)'_{\cdot,i}\Psi_x^{-1}(\Lambda_x)_{\cdot,i} - \sum_{1\leq j\leq d_\xi, j\neq i}|(\Lambda_x)'_{\cdot,i}\Psi_x^{-1}(\Lambda_x)_{\cdot,j}|\right).$$

Since $\lambda_{\min}(\Psi_x^{-1}) = 1/\lambda_{\max}(\Psi_x)$, Assumption 6 (1) implies that $1/\lambda_{\max}(\Psi_x) > 1/M_{\Psi_x} > 0$. Since $(\Lambda_x)'_{\cdot,i}(\Lambda_x)_{\cdot,i} = \sum_{k=1}^{d_x}(\Lambda_x)_{k,i}^2 > 0$, we have

$$(\Lambda_x)'_{\cdot,i}\Psi_x^{-1}(\Lambda_x)_{\cdot,i} = ((\Lambda_x)'_{\cdot,i}(\Lambda_x)_{\cdot,i})\cdot\underbrace{[(\Lambda_x)'_{\cdot,i}\Psi_x^{-1}(\Lambda_x)_{\cdot,i}/(\Lambda_x)'_{\cdot,i}(\Lambda_x)_{\cdot,i}]}_{\geq M_{\Psi_x}^{-1}}$$

$$\geq M_{\Psi_x}^{-1}(\Lambda_x)'_{\cdot,i}(\Lambda_x)_{\cdot,i} = M_{\Psi_x}^{-1}\sum_{k=1}^{d_x}(\Lambda_x)_{k,i}^2.$$

By Assumption 6 (2) and (3), there are $N_i$ non-zero elements in this sum, and these are larger than $m_{\Lambda_x}^2 > 0$. Therefore,

$$\sum_{k=1}^{d_x}(\Lambda_x)_{k,i}^2 > N_i m_{\Lambda_x}^2,$$

which further implies

$$(\Lambda_x)'_{\cdot,i}\Psi_x^{-1}(\Lambda_x)_{\cdot,i} > M_{\Psi_x}^{-1}N_i m_{\Lambda_x}^2.$$

We now bound the negative term in $G$ from below, which means providing an upper bound for $\sum_{1\leq j\leq d_\xi, j\neq i}|(\Lambda_x)'_{\cdot,i}\Psi_x^{-1}(\Lambda_x)_{\cdot,j}|$. From the triangle inequality, we have that

$$|(\Lambda_x)'_{\cdot,i}\Psi_x^{-1}(\Lambda_x)_{\cdot,j}| = |\sum_{k=1}^{d_x}\sum_{l=1}^{d_x}(\Lambda_x)_{k,i}(\Lambda_x)_{l,j}(\Psi_x^{-1})_{k,l}| \leq \sum_{k=1}^{d_x}\sum_{l=1}^{d_x}|(\Lambda_x)_{k,i}(\Lambda_x)_{l,j}(\Psi_x^{-1})_{k,l}|.$$

Recalling that $C_{i,j}$ is the number of non-zero elements in the sum, we get that

$$\sum_{k=1}^{d_x}\sum_{l=1}^{d_x}|(\Lambda_x)_{k,i}(\Lambda_x)_{l,j}(\Psi_x^{-1})_{k,l}| \leq C_{i,j}\max_{1\leq i,j\leq d_x}|(\Lambda_x)_{k,i}(\Lambda_x)_{l,j}(\Psi_x^{-1})_{k,l}|.$$

By Assumption 6 (2), we have that $|(\Lambda_x)_{k,i}(\Lambda_x)_{l,j}| < M_{\Lambda_x}^2$ which is fixed for all $d_x$. Again, we use that for a symmetric positive definite $m\times m$ matrix $M$, we have that $\max_{1\leq i,j\leq m}|M_{i,j}| \leq \max_{|x|=1}x'Mx = \lambda_{\max}(M)$. Since $\Psi_x$ is a positive definite matrix, we therefore get from Lemma 12 that $\max_{1\leq i,j\leq d_x}|(\Psi_x^{-1})_{i,j}| \leq \lambda_{\max}(\Psi_x^{-1}) = 1/\lambda_{\min}(\Psi)$. Since $\lambda_{\min}(\Psi) > m_{\Psi_x} > 0$ we get $1/\lambda_{\min}(\Psi) < 1/m_{\Psi_x}$. Therefore, we get that $\max_{1\leq i,j\leq d_x}|(\Lambda_x)_{k,i}(\Lambda_x)_{l,j}(\Psi_x^{-1})_{k,l}| \leq M_{\Lambda_x}^2/m_{\Psi_x}$, which gives

$$\sum_{k=1}^{d_x}\sum_{l=1}^{d_x}|(\Lambda_x)_{k,i}(\Lambda_x)_{l,j}(\Psi_x^{-1})_{k,l}| \leq C_{i,j}M_{\Lambda_x}^2/m_{\Psi_x}.$$

We therefore get that

$$\begin{aligned} G &\geq \min_{1\leq i\leq d_\xi}\left(M_{\Psi_x}^{-1}N_i m_{\Lambda_x}^2 - \sum_{1\leq j\leq d_\xi, j\neq i}C_{i,j}\max_{1\leq i,j\leq d_x}|(\Lambda_x)_{k,i}(\Lambda_x)_{l,j}(\Psi_x^{-1})_{k,l}|\right) \\ &\geq \min_{1\leq i\leq d_\xi}\left(M_{\Psi_x}^{-1}N_i m_{\Lambda_x}^2 - \sum_{1\leq j\leq d_\xi, j\neq i}C_{i,j}M_{\Lambda_x}^2/m_{\Psi_x}\right) \\ &\geq \min_{1\leq i\leq d_\xi}N_i\left(\frac{m_{\Lambda_x}^2}{M_{\Psi_x}} - \frac{M_{\Lambda_x}^2}{m_{\Psi_x}}\frac{1}{N_i}\sum_{1\leq j\leq d_\xi, j\neq i}C_{i,j}\right). \end{aligned}$$

This shows the first statement by inversion of both sides.

Statement (2):

Let $\tilde{e} > 0$ be given. Suppose $\tilde{e}$ is so small that $M_{\Psi_x}^{-1} m_{\Lambda_x}^2 / (M_{\Lambda_x}^2 / m_{\Psi_x}) - \tilde{e}/(M_{\Lambda_x}^2 / m_{\Psi_x}) > 0$. This is possible because $M_{\Psi_x}^{-1} m_{\Lambda_x}^2 / (M_{\Lambda_x}^2 / m_{\Psi_x}) > 0$ and $(M_{\Lambda_x}^2 / m_{\Psi_x}) > 0$.

Recall that $N_i > 0$ and $C_{i,j} \geq 0$ and since $\lim_{d_x \to \infty} \frac{1}{N_i} \sum_{1 \leq j \leq d_\xi, j \neq i} C_{i,j} = 0$ for all $1 \leq i \leq d_\xi$ by Assumption 6 (4), we have that there exists a $D > 0$ so that for all $d_x > D$ we have $0 \leq \frac{1}{N_i} \sum_{1 \leq j \leq d_\xi, j \neq i} C_{i,j} < M_{\Psi_x}^{-1} m_{\Lambda_x}^2 / (M_{\Lambda_x}^2 / m_{\Psi_x}) - \tilde{e}/(M_{\Lambda_x}^2 / m_{\Psi_x})$ for all $1 \leq i \leq d_\xi$. Therefore, recalling that $M_{\Lambda_x}^2 / m_{\Psi_x} > 0$ for all such $d_x$ and any $1 \leq i \leq d_\xi$ we have

$$
\begin{aligned}
\frac{m_{\Lambda_x}^2}{M_{\Psi_x}} - \frac{M_{\Lambda_x}^2}{m_{\Psi_x}} \frac{1}{N_i} \sum_{1 \leq j \leq d_\xi, j \neq i} C_{i,j} &\geq \frac{m_{\Lambda_x}^2}{M_{\Psi_x}} - \frac{M_{\Lambda_x}^2}{m_{\Psi_x}} \left( M_{\Psi_x}^{-1} m_{\Lambda_x}^2 / (M_{\Lambda_x}^2 / m_{\Psi_x}) - \tilde{e}/(M_{\Lambda_x}^2 / m_{\Psi_x}) \right) \\
&= \frac{m_{\Lambda_x}^2}{M_{\Psi_x}} - M_{\Psi_x}^{-1} m_{\Lambda_x}^2 + \tilde{e} \\
&= \tilde{e}.
\end{aligned}
$$

Therefore, for all sufficiently large $d_x$, we have

$$
\begin{aligned}
G &\geq \min_{1 \leq i \leq d_\xi} N_i \left( M_{\Psi_x}^{-1} m_{\Lambda_x}^2 - M_{\Lambda_x}^2 / m_{\Psi_x} M_{\Psi_x}^{-1} m_{\Lambda_x}^2 / (M_{\Lambda_x}^2 / m_{\Psi_x}) - \tilde{e}/(M_{\Lambda_x}^2 / m_{\Psi_x}) \right) \\
&= \min_{1 \leq i \leq d_\xi} N_i \tilde{e}
\end{aligned}
$$

which by Assumption 6 (3) goes to infinity. Therefore, the smallest eigenvalue of $\Lambda_x' \Psi_x^{-1} \Lambda_x$ goes to infinity and, consequently, the largest eigenvalue of $\mathrm{Cov}\ r_\xi = \left( \Lambda_x' \Psi_x^{-1} \Lambda_x \right)^{-1}$ goes to zero, which further implies

$$
\lim_{d_x \to \infty} \max_{1 \leq i,j \leq d_\xi} \mathrm{Cov}\ r_\xi = 0.
$$

□

E.4.7. *Proof of Proposition 4.*

*Proof of Proposition 4.* All limits are with respect to $d_x \to \infty$.

Let $\tilde{H}_{d_x}(x) = \mathbb{E}[\eta | \ddot{\xi} = x]$. We start by showing that $H_{d_x}(x) = \tilde{H}_{d_x}(x)$. We have that $\mathbb{E}[\ddot{\eta} | \ddot{\xi}] = \mathbb{E}[\eta + r_\eta | \ddot{\xi}] = \mathbb{E}[\eta | \ddot{\xi}] + \mathbb{E}[r_\eta | \ddot{\xi}]$.

We have that $\ddot{\xi} = \xi + r_\xi$ is independent to $r_\eta$, because $\xi$ and $r_\xi$ is, which is seen as follows: By Assumption 4 (2), we have $r_\xi$ is independent to $r_\eta$. We now show that also $\xi$ is independent to $r_\eta$: Since $r_\eta$ is a function of $\varepsilon$, and $\xi$ is a function of $f$, $\xi$ is independent to $r_\eta$ by Assumption 2 (1) which says that $\varepsilon$ is independent to $f$.

Therefore, $\mathbb{E}[r_\eta | \ddot{\xi}] = \mathbb{E}[r_\eta] = \mathbb{E} \Delta_\eta \varepsilon_y = \Delta_\eta \mathbb{E} \varepsilon_y$, which is zero by Assumption 1 (1).

The desired conclusion therefore follows if we show that $\sup_{x \in A} |\tilde{H}_{d_x}(x) - H(x)| \to 0$.

From Assumption 7 (1), $f = (\xi', \eta')'$ and $r_\xi$ have densities. From Assumption 4 (1), $r_\xi$ is independent to $f$. Therefore, $(\eta', \ddot{\xi}') = (\eta', \xi' + r_\xi')'$ has a density given by the convolution formula

$$
f_{\ddot{\xi},\eta}(x, y) = f_{\xi + r_\xi, \eta}(x, y) = \mathbb{E} f_{\xi,\eta}(x - r_\xi, y). \tag{14}
$$

Recall that we without loss of generality assume $d_\eta = 1$. By Assumption 7 (1), $\eta, \xi$ have densities, and therefore the conditional expectation $\tilde{H}_{d_x}$ is given by the classical formula

$$
\tilde{H}_{d_x}(x) = \int_{-\infty}^{\infty} y \frac{f_{\ddot{\xi},\eta}(x, y)}{f_{\ddot{\xi}}(x)} \, dy.
$$

We notice that

$$\begin{aligned}\tilde{H}_{d_x}(x) &= \int_{-\infty}^{\infty} y \frac{f_{\ddot{\xi},\eta}(x,y)}{f_{\ddot{\xi}}(x)}\, dy \\ &= \frac{f_\xi(x)}{f_{\ddot{\xi}}(x)} \int_{-\infty}^{\infty} y \frac{f_{\ddot{\xi},\eta}(x,y)}{f_\xi(x)}\, dy\end{aligned}$$

Recalling eq. (14), we have

$$\begin{aligned}\int_{-\infty}^{\infty} y \frac{f_{\ddot{\xi},\eta}(x,y)}{f_\xi(x)}\, dy &= \int_{-\infty}^{\infty} y \frac{\mathbb{E} f_{\xi,\eta}(x - r_\xi, y)}{f_\xi(x)}\, dy \\ &= \mathbb{E} \int_{-\infty}^{\infty} y \frac{f_{\xi,\eta}(x - r_\xi, y)}{f_\xi(x)}\, dy \\ &= \mathbb{E} H(x - r_\xi),\end{aligned}$$

and so for $\omega(x,h) = \mathbb{E}H(x-h) - H(x)$

$$\begin{aligned}\tilde{H}_{d_x}(x) &= \frac{f_\xi(x)}{f_{\ddot{\xi}}(x)} \mathbb{E}H(x - r_\xi) \\ &= [\mathbb{E}H(x - r_\xi)] - \left(1 - \frac{f_\xi(x)}{f_{\ddot{\xi}}(x)}\right) [\mathbb{E}H(x - r_\xi)] \\ &= [\mathbb{E}H(x - r_\xi)] - \underbrace{\frac{f_{\ddot{\xi}}(x) - f_\xi(x)}{f_{\ddot{\xi}}(x) - f_\xi(x) + f_\xi(x)}}_{=R_{d_x}(x)} [\mathbb{E}H(x - r_\xi)] \\ &= H(x) + [\mathbb{E}\left(H(x - r_\xi)\right) - H(x)] - R_{d_x}(x) \left[\mathbb{E}\left(H(x - r_\xi)\right) - H(x)\right] - R_{d_x}(x) H(x) \\ &\overset{(a)}{=} H(x) + [\mathbb{E}\left(H(x - r_\xi) - H(x)\right)] - R_{d_x}(x) \left[\mathbb{E}\left(H(x - r_\xi) - H(x)\right)\right] - R_{d_x}(x) H(x) \\ &= H(x) + [\mathbb{E}\omega(x, r_\xi)] - R_{d_x}(x) \left[\mathbb{E}\omega(x, r_\xi)\right] - R_{d_x}(x) H(x) \qquad (15)\end{aligned}$$

*(a)* $H(x)$ is non-random, therefore $\mathbb{E}\left(H(x - r_\xi)\right) - H(x) = \mathbb{E}\left(H(x - r_\xi) - H(x)\right)$.

In a separate step below, we show that $\sup_{x\in\mathcal{S}^\rho} |R_{d_x}(x)| \to 0$. We now show that this leads to the required conclusion.

From eq. (15), the triangle inequality and that $\sup_x |a(x)b(x)| \leq (\sup_x |a(x)|)(\sup_x |b(x)|)$, we get

$$\begin{aligned}&\sup_{x\in\mathcal{S}^\rho} |\tilde{H}_{d_x}(x) - H(x)| \\ &= \sup_{x\in\mathcal{S}^\rho} \Big| [\mathbb{E}\omega(x, r_\xi)] - R_{d_x}(x) \left[\mathbb{E}\omega(x, r_\xi)\right] - R_{d_x}(x) H(x) \Big| \\ &\leq \sup_{x\in\mathcal{S}^\rho} |\mathbb{E}\omega(x, r_\xi)| + \sup_{x\in\mathcal{S}^\rho} |R_{d_x}(x)| \sup_{x\in\mathcal{S}^\rho} |\mathbb{E}\omega(x, r_\xi)| + \sup_{x\in\mathcal{S}^\rho} |R_{d_x}(x)| \sup_{x\in\mathcal{S}^\rho} |H(x)|.\end{aligned}$$

The conclusion now follows: Firstly we have $\sup_{x\in\mathcal{S}^\rho} |\mathbb{E}\omega(x, r_\xi)| \to 0$ by Assumption 7 (3) (a). Secondly, by the separate step proved below, we have $\sup_{x\in\mathcal{S}^\rho} |R_{d_x}(x)| \to 0$. Thirdly, from Assumption 7 (3) (b) we have $\sup_{x\in\mathcal{S}^\rho} |H(x)| < \infty$, so that also the last term above goes to zero.

Bounding of $R_{d_x}$, step 1: We first show that $\sup_{x\in\mathcal{S}^\rho} |f_\xi(x) - f_{\ddot{\xi}}(x)| \to 0$, and then use this to show that $\sup_{x\in\mathcal{S}^\rho} |R_{d_x}(x)| \to 0$.

Let $x \in \mathcal{S}^\rho$. The density $f_{\ddot{\xi}} = f_{\xi + r_\xi}$ is given by the convolution expression

$$f_{\xi + r_\xi}(x) = \mathbb{E} f_\xi(x - r_\xi).$$

Therefore, for the indicator function $I\{\|r_\xi\|_2 < \rho\}$, which is one iff $\|r_\xi\|_2 < \rho$ and zero else, we have

$$\begin{aligned}\sup_{x\in\mathcal{S}^\rho}|f_{\ddot{\xi}}(x)-f_\xi(x)| &= \sup_{x\in\mathcal{S}^\rho}|\mathbb{E}f_\xi(x-r_\xi)-f_\xi(x)| \\ &= \sup_{x\in\mathcal{S}^\rho}\big|\mathbb{E}I\{\|r_\xi\|_2<\rho\}\big[f_\xi(x-r_\xi)-f_\xi(x)\big]+\mathbb{E}I\{\|r_\xi\|_2\geq\rho\}\big[f_\xi(x-r_\xi)-f_\xi(x)\big]\big| \\ &\leq \sup_{x\in\mathcal{S}^\rho}\big|\mathbb{E}I\{\|r_\xi\|_2<\rho\}\big[f_\xi(x-r_\xi)-f_\xi(x)\big]\big|+ \\ &\qquad \sup_{x\in\mathcal{S}^\rho}\big|\mathbb{E}I\{\|r_\xi\|_2\geq\rho\}\big[f_\xi(x-r_\xi)-f_\xi(x)\big]\big|,\end{aligned}$$

by the triangle inequality.

We bound the two terms separately. Recall that $\|a\|_2$ is the Euclidean norm of a vector $a$.

First, suppose $\|r_\xi\|_2 < \rho$. We can then bound $|f_\xi(x) - f_\xi(x - r_\xi)|$ via the mean value theorem (e.g. Edwards, 1973, p 90, Theorem 3.4): Since $f_\xi$ is continuously differentiable in $\mathcal{S}^\rho$ by Assumption 7 (3) (d), we have for the (random, $d_x$-dependent) line segment $L(x, r_\xi) = \{x + \alpha(x - r_\xi) : \alpha \in [0, 1]\}$. Then $f_\xi(x) - f_\xi(x - r_\xi) = f'(c)r_\xi$ where $c \in L(x, r_\xi)$ and $f'(c)$ is the derivative row vector $f'(c) = (D_1 f(c), \ldots, D_{d_x} f(c))$ where $D_j$ is partial derivation with respect to the $j$'th coordinate. This gives

$$\begin{aligned}|f_\xi(x)-f_\xi(x-r_\xi)| &= |f'(c)r_\xi| \\ &\overset{(a)}{\leq} \|f'(c)\|_2\|r_\xi\|_2 \\ &\overset{(b)}{\leq} \sup_{z\in L(x,r_\xi)}\|f'_\xi(z)\|_2\|r_\xi|_2\end{aligned}$$

*(a)* Cauchy-Schwartz. *(b)* Since $c \in L(x, r_\xi)$.

Since $\|r_\xi\|_2 < \rho$, we have $L(x, r_\xi) \subseteq \mathcal{S}^\rho$ by the definition of $\mathcal{S}^\rho$, and so $\sup_{z\in L(x,r_\xi)} |f'_\xi(z)| \leq \sup_{z\in\mathcal{S}^\rho} \|f'_\xi(z)\|_2 < \infty$ by Assumption 7 (3) (d), and consequently

$$|f_\xi(x)-f_\xi(x-r_\xi)| \leq \sup_{z\in\mathcal{S}^\rho}\|f'_\xi(z)\|_2\|r_\xi\|_2. \tag{16}$$

This shows that

$$I\{\|r_\xi\|_2<\rho\}|f_\xi(x-r_\xi)-f_\xi(x)| \leq I\{\|r_\xi\|_2<\rho\}\|r_\xi\|_2\sup_{z\in\mathcal{S}^\rho}|f'_\xi(z)|,$$

because either $\|r_\xi\|_2 < \rho$, and then eq. (16) holds, or $\|r_\xi\|_2 \geq \rho$, and then the indicator functions both sizes of the inequality are zero, and equality is preserved.

This shows that

$$\begin{aligned}\sup_{x\in\mathcal{S}^\rho}|\mathbb{E}I\{\|r_\xi\|_2<\rho\}[f_\xi(x-r_\xi)-f_\xi(x)]| &\leq \sup_{x\in\mathcal{S}^\rho}\mathbb{E}I\{\|r_\xi\|_2<\rho\}|f_\xi(x-r_\xi)-f_\xi(x)| \\ &\leq \sup_{x\in\mathcal{S}^\rho}\mathbb{E}I\{\|r_\xi\|_2<\rho\}\|r_\xi\|_2\sup_{z\in\mathcal{S}^\rho}\|f'_\xi(z)\|_2 \\ &= \left(\sup_{y\in\mathcal{S}^\rho}\|f'_\xi(y)\|_2\right)\mathbb{E}I\{\|r_\xi\|_2<\rho\}\|r_\xi\|_2.\end{aligned}$$

Recall that $r_\xi$ converges in probability by Assumption 7 (4). Since the function $g(x) = I\{\|x\|_2 < \rho\}\|x\|_2$ is continuous, $g(r_\xi)$ converges in probability to $g(0) = 0$ by the continuous mapping theorem. Since $I\{\|r_\xi\|_2 < \rho\}\|r_\xi\|_2$ is bounded by $\rho$ and converges in probability to 0 as $d_x \to \infty$, the variable therefore also converges in expectation (e.g. Theorem 6.4 in Bierens, 2004). Therefore the above display goes to zero since $\sup_{y\in\mathcal{S}^\rho} \|f'_\xi(y)\|_2 < \infty$ by Assumption 7 (3) (d).

We now consider $\sup_{x\in\mathcal{S}^\rho}|\mathbb{E}I\{\|r_\xi\|_2\geq\rho\}[f_\xi(x-r_\xi)-f_\xi(x)]|$, which by the triangle inequality is bounded by

$$\begin{aligned}
&\sup_{x\in\mathcal{S}^\rho}|\mathbb{E}I\{\|r_\xi\|_2\geq\rho\}[|f_\xi(x-r_\xi)|+|f_\xi(x)|]|\\
&\leq\sup_{x\in\mathcal{S}^\rho}\mathbb{E}I\{\|r_\xi\|_2\geq\rho\}2\sup_{y\in\mathcal{S}^\rho}|f_\xi(y)|\\
&=\mathbb{E}I\{\|r_\xi\|_2\geq\rho\}2\sup_{y\in\mathcal{S}^\rho}|f_\xi(y)|\\
&=2\sup_{y\in\mathcal{S}^\rho}|f_\xi(y)|\mathbb{E}I\{\|r_\xi\|_2\geq\rho\}\\
&=P(\|r_\xi\|_2\geq\rho)2\sup_{y\in\mathcal{S}^\rho}|f_\xi(y)|,
\end{aligned}$$

which goes to zero as $d_x\to\infty$ by the definition of convergence in probability since $r_\xi=o_P(1)$ by Assumption 7 (4).

Bounding of $R_{d_x}$, step 2: We now return to $R_{d_x}(x)$ directly, using the bound from step 1. Now recall $0<\inf_{y\in\mathcal{S}^\rho}f_\xi(y)\leq f_\xi(x)$. By Step 1, we have that for any $\tilde{e}>0$, we have that for all sufficiently large $d_x$, we have $-\tilde{e}<f_{\ddot{\xi}}(x)-f_\xi(x)<\tilde{e}$ for all $x\in\mathcal{S}^\rho$. Let $0<\tilde{e}<\inf_{y\in\mathcal{S}^\rho}f_\xi(y)$, so that $-\tilde{e}+\inf_{y\in\mathcal{S}^\rho}f_\xi(y)>0$. Then

$$f_{\ddot{\xi}}(x)-f_\xi(x)+f_\xi(x)>-\tilde{e}+f_\xi(x)\geq-\tilde{e}+\inf_{y\in\mathcal{S}^\rho}f_\xi(y)>0$$

Therefore, $|f_{\ddot{\xi}}(x)-f_\xi(x)+f_\xi(x)|=f_{\ddot{\xi}}(x)-f_\xi(x)+f_\xi(x)$, and

$$\begin{aligned}
\left|\frac{f_{\ddot{\xi}}(x)-f_\xi(x)}{f_{\ddot{\xi}}(x)-f_\xi(x)+f_\xi(x)}\right|&=\frac{|f_{\ddot{\xi}}(x)-f_\xi(x)|}{|f_{\ddot{\xi}}(x)-f_\xi(x)+f_\xi(x)|}\\
&\leq\frac{\tilde{e}}{-\tilde{e}+\inf_{y\in\mathcal{S}^\rho}f_\xi(y)}.
\end{aligned}$$

Since $\inf_{y\in\mathcal{S}^\rho}f_\xi(y)>0$ by Assumption 7 (3) (c),

$$\lim_{\tilde{e}\to0^+}\frac{\tilde{e}}{-\tilde{e}+\inf_{y\in\mathcal{S}^\rho}f_\xi(y)}=\frac{0}{-0+\inf_{y\in\mathcal{S}^\rho}f_\xi(y)}=0$$

and the convergence occurs at a rate that is independent of $x$. Therefore,

$$\sup_{x\in\mathcal{S}^\rho}|R_{d_x}(x)|=\sup_{x\in\mathcal{S}^\rho}\left|\frac{f_{\ddot{\xi}}(x)-f_\xi(x)}{f_{\ddot{\xi}}(x)-f_\xi(x)+f_\xi(x)}\right|\to0.$$

□

E.4.8. *Proof of Lemma 4.* The following proof is done for the $d_x$ measurements of $\xi$, as it is needed specifically for the rationale. It can easily be extended for all $d_z$ measurements by simply replacing $d_x$ by $d_z=d_x+d_y$, $d_\xi$ by $d_f=d_\xi+d_\eta$, $\varepsilon_x$ by $\varepsilon=(\varepsilon_x',\varepsilon_y')'$ and by enlarging $\Lambda_x$ and $\Psi_x$ by the corresponding elements of regarding the measurements of $\eta$.

*Proof of Lemma 4.* From Assumption 8 we have that Cov $\varepsilon_x=\Psi_x$ is an invertible diagonal $d_x\times d_x$ matrix, which further implies $\Psi_x^{-1}$ is a diagonal matrix. We call $\psi_{ii}$ the residual variance of variable $i$, for $i=1,\dots,d_x$:

$$\Psi_x:=\operatorname{diag}(\psi_{11},\dots,\psi_{d_xd_x})=\begin{pmatrix}\psi_{11}&&\\0&\ddots&\\0&\dots\ 0&\psi_{d_xd_x}\end{pmatrix},\text{ and },\Psi_x^{-1}:=\operatorname{diag}\left(\frac{1}{\psi_{11}},\dots,\frac{1}{\psi_{d_xd_x}}\right),$$

where diag stacks the given vector into a diagonal matrix. Further, we call $\lambda_{ij}$ the $(i,j)$-entry of $\Lambda_x$.

From Assumption 8 (2) we have that $\Lambda_x$ only has one non-zero element per row, which implies that $\Lambda_x'\Psi_x^{-1}$ is a $d_\xi \times d_x$ matrix that has the identical non-zero elements as $\Lambda_x'$, the elements are

$$\Lambda_x'\Psi_x^{-1} := \left(\frac{\lambda_{ij}}{\psi_{ii}}\right)_{j,i,i=1,\dots,d_x,j=1,\dots,d_\xi}.$$

Hence, the element $(j,i)$ of $\Lambda_x'\Psi_x^{-1}$ is $\frac{\lambda_{ij}}{\psi_{ii}}$, where $\lambda_{ij}$ is either zero or non-zero. Post-multiplying with $\Lambda_x$ results in a diagonal $d_\xi \times d_\xi$ matrix:

$$\Lambda_x'\Psi_x^{-1}\Lambda_x = \operatorname{diag}\left(\left(\sum_{i=1}^{d_x}\frac{\lambda_{ij}^2}{\psi_{ii}}\right)_{j=1,\dots,d_\xi}\right).$$

The off-diagonal elements are zero, since the columns of $\Lambda_x$ are orthogonal, i.e., $(\Lambda_{\cdot,j_1})'\Lambda_{\cdot,j_2} = 0$, for $j_1 \neq j_2$, where $\Lambda_{\cdot,j_1}$ and $\Lambda_{\cdot,j_2}$ correspond to the $j_1$-th and $j_2$-th column of $\Lambda_x$, respectively. The $j$-th diagonal element of $\Lambda_x'\Psi_x\Lambda_x$ is the sum $\sum_{i=1}^{d_x}\frac{\lambda_{ij}^2}{\psi_{ii}}$, which is nonzero since $\Lambda_x$ has full column rank.

Now, since $\Lambda_x'\Psi_x^{-1}\Lambda_x$ is a diagonal matrix, we have for its inverse a $d_\xi \times d_\xi$ matrix:

$$(\Lambda_x'\Psi_x^{-1}\Lambda_x)^{-1} = \operatorname{diag}\left(\left(\frac{1}{\sum_{i=1}^{d_x}\frac{\lambda_{ij}^2}{\psi_{ii}}}\right)_{j=1,\dots,d_\xi}\right).$$

The derived entities are used in the following proofs for the specific subsections of Lemma 4.

Statement (1): Since $(\Lambda_x'\Psi_x^{-1}\Lambda_x)^{-1}$ is diagonal and $\Lambda_x'\Psi_x^{-1}$ has the identical non-zero elements as $\Lambda_x'$, we have that $(\Lambda_x'\Psi_x^{-1}\Lambda_x)^{-1}\Lambda_x'\Psi_x^{-1}$ also has the identical non-zero elements as $\Lambda_x'$. The elements result as

$$\Delta_x = (\Lambda_x'\Psi_x^{-1}\Lambda_x)^{-1}\Lambda_x'\Psi_x^{-1} = \left(\frac{\lambda_{ij}}{\psi_{ii}\sum_{k=1}^{d_x}\frac{\lambda_{kj}^2}{\psi_{kk}}}\right)_{j,i,i=1,\dots,d_x,j=1,\dots,d_\xi}.$$

The elements of $\Delta_x$ are nonzero if $\lambda_{ij}$ is nonzero.

Statement (2): Now, since $\Delta_x$ has the same non-zero elements as $\Lambda_x'$, this implies that $r_\xi = \Delta_x\varepsilon_x$ consists of elements that are independent sums. This is so since their elements are mutually independent and $\Lambda_x$ has only one non-zero element per row (and $\Lambda_x'$ only has one non-zero element per column). For $r_\xi := (r_1,\dots,r_{d_\xi})'$ we have for $j = 1,\dots,d_\xi$:

$$r_j := \sum_{i=1}^{d_x}\frac{\lambda_{ij}}{\psi_{ii}\sum_{k=1}^{d_x}\frac{\lambda_{kj}^2}{\psi_{kk}}}\varepsilon_i,$$

where $\varepsilon_i$ is the $i$-th element of $\varepsilon_x$, for $i = 1,\dots,d_x$, and $\lambda_{ij}$ is non-zero for the set of variables measuring the $j$-th latent variable denoted as $\mathcal{I}_j$ (the item set of the $j$-th latent variable) with $\cup_{j=1}^{d_\xi}\mathcal{I}_j = \{1,\dots,d_x\}$ and with $\mathcal{I}_{j_1}\cap\mathcal{I}_{j_2} = \emptyset$ for $j_1 \neq j_2$. Hence, we can write $r_j$ as

$$r_j := \sum_{i\in\mathcal{I}_j}\frac{\lambda_{ij}}{\psi_{ii}\sum_{k=1}^{d_x}\frac{\lambda_{kj}^2}{\psi_{kk}}}\varepsilon_i.$$

Now since the $\mathcal{I}_j$ are disjoint, it follows from Assumption 8 that the components of $r_\xi$ are independent.

Statement (3): We have that

$$\text{Cov } r_\xi = (\Lambda_x' \Psi_x^{-1} \Lambda_x)^{-1}.$$

from Proposition 2. We have already derived the specific shape of this object under Assumption 5. Hence,

$$\text{Cov } r_\xi := (\Lambda_x' \Psi_x^{-1} \Lambda_x)^{-1} = \text{diag}\left( \left( \frac{1}{\sum_{k=1}^{d_x} \frac{\lambda_{kj}^2}{\psi_{kk}}} \right)_{j=1,\ldots,d_\xi} \right).$$

The diagonal elements of Cov $r_\xi$ are, therefore, $d_j := \left( \frac{1}{\sum_{k=1}^{d_x} \frac{\lambda_{kj}^2}{\psi_{kk}}} \right)$ for $j = 1, \ldots, d_\xi$.

This completes the proof of Lemma 4.

□

E.4.9. *Proof of Proposition 5.*

*Proof of Proposition 5.* Since $r_\xi$ consists of independent elements from Lemma 4 (2), we may without loss of generality consider just one of the elements, say the first, as joint convergence in distribution of independent random variables is implied by their marginal convergence in distribution, e.g., by the convergence of their characteristic function which is the product of their marginal characteristic functions. To simplify notation, this argument is equivalent to $d_\xi = 1$, which we assume without loss of generality.

By Lemma 4, we have

$$r_{1,d_x} = \frac{1}{\sum_{k=1}^{d_x} \frac{\lambda_{k1}^2}{\psi_{kk}}} \sum_{j=1}^{d_x} \frac{\lambda_{j1}}{\psi_{jj}} \varepsilon_j = \frac{1}{\sum_{k=1}^{d_x} \frac{\lambda_{k1}^2}{\psi_{kk}}} \sum_{j=1}^{d_x} \frac{\lambda_{j1}}{\sqrt{\psi_{jj}}} \frac{\varepsilon_j}{\sqrt{\psi_{jj}}}.$$

Define the standardized errors

$$u_j := \frac{\varepsilon_j}{\sqrt{\psi_{jj}}}.$$

Also define

$$\alpha_j := \frac{\lambda_{j1}^2}{\psi_{jj}}$$

and notice that $(\alpha_j)_j$ is a sequence of positive numbers. Let us also write

$$n_{d_x} = \sum_{j=1}^{d_x} \alpha_j.$$

When $\alpha_j$ is constant and equal to $\alpha_0$, $n_{d_x} = d_x \alpha_0$, and so $n_{d_x}$ is similar to the sample size in non-weighted sums.

With this notation, we have

$$r_{1,d_x} = \frac{1}{n_{d_x}} \sum_{i=1}^{d_x} \sqrt{\alpha_j} u_j. \tag{17}$$

We apply the Lyapunov central limit theorem (Billingsley, 1995, Section 27), which says that for an independent sequence of variables $X_1, \ldots,$ we have

$$\frac{1}{s_{d_x}} \sum_{i=1}^{d_x} X_i \xrightarrow[d_x \to \infty]{d} N(0,1) \tag{18}$$

where $s_{d_x}^2 = \sum_{i=1}^{d_x} \operatorname{Var} X_i$, as long as the Lyapunov condition

$$\lim_{n\to\infty} \beta_{n,\delta} = 0, \quad \text{where } \beta_{n,\delta} = \frac{1}{s_{d_x}^{2+\delta}} \sum_{i=1}^{d_x} \mathbb{E}|X_i|^{2+\delta} \tag{19}$$

is fulfilled, for a $\delta > 0$.

Let $X_i = \sqrt{\alpha_i} u_i$. Since for $u_i = \varepsilon_i / \sqrt{\psi_{ii}}$, $(u_i)_i$ is a sequence of independent standardized random variables from Assumption 9, eq. (18) will follow as long as the Lyapunov condition is fulfilled for a $\delta > 0$.

We will now show that the Lyapunov condition is fulfilled with the $\delta$ from Assumption 9 (2). This will give the stated conclusion, because

$$s_{d_x}^2 = \sum_{i=1}^{d_x} \operatorname{Var} X_i = \sum_{i=1}^{d_x} \alpha_i = n_{d_x},$$

since $\operatorname{Var} u_j = \frac{1}{\psi_{jj}} \operatorname{Var} \varepsilon_j = 1$, and eq. (18) works with

$$\frac{1}{s_{d_x}} \sum_{i=1}^{d_x} X_i = \frac{1}{\sqrt{n_{d_x}}} \sum_{i=1}^{d_x} \sqrt{\alpha_i} u_i = \sqrt{n_{d_x}} \frac{1}{n_{d_x}} \sum_{i=1}^{d_x} \sqrt{\alpha_i} u_i = \sqrt{n_{d_x}} r_{1,d_x}.$$

We have

$$\mathbb{E}|X_i|^{2+\delta} = \mathbb{E}(\sqrt{\alpha_j}|u_j|)^{2+\delta} = \alpha_j^{1+\delta/2} \mathbb{E}|u_j|^{2+\delta} \tag{20}$$

and

$$s_{d_x}^{2+\delta} = (s_{d_x}^2)^{1+\delta/2} = \left( \sum_{i=1}^{d_x} \alpha_j \right)^{1+\delta/2} \tag{21}$$

By Assumption 9 (3), we have

$$c_\delta := \sup_j \mathbb{E}|u_j|^{2+\delta} < \infty$$

Therefore,

$$\begin{aligned} \beta_{n,\delta} = \frac{1}{s_{d_x}^{2+\delta}} \sum_{i=1}^{d_x} \mathbb{E}|X_i|^{2+\delta} &\overset{(a)}{=} \frac{1}{\left(\sum_{i=1}^{d_x} \alpha_j\right)^{1+\delta/2}} \sum_{i=1}^{d_x} \alpha_j^{1+\delta/2} \mathbb{E}|u_j|^{2+\delta} \\ &\overset{(b)}{\leq} c_\delta \frac{\sum_{i=1}^{d_x} \alpha_j^{1+\delta/2}}{\left(\sum_{i=1}^{d_x} \alpha_j\right)^{1+\delta/2}} \\ &= c_\delta \left( \sum_{i=1}^{d_x} \alpha_j^{1+\delta/2} \right) \frac{1}{\left(\sum_{i=1}^{d_x} \alpha_j\right)^{1+\delta/2}} \\ &\overset{(c)}{\leq} c_\delta d_x M_{\lambda/\psi}^{1+\delta/2} \frac{1}{{d_x}^{1+\delta/2} m_{\lambda/\psi}^{1+\delta/2}} \\ &= (c_\delta M_{\lambda/\psi}^{1+\delta/2} m_{\lambda/\psi}^{-1-\delta/2}) {d_x}^{-\delta/2} \\ &\overset{(d)}{\to} 0 \quad \text{as } d_x \to \infty. \end{aligned}$$

*(a)* Use eq. (20) and (21). *(b)* Since $(\alpha_j)_j$ is a sequence of positive numbers, all terms in the two sums are positive, and $\alpha_j^{1+\delta/2} \mathbb{E}|u_j|^{2+\delta} \leq \alpha_j^{1+\delta/2} c_\delta$. Then factorize out $c_\delta$. *(c)* From Assumption 9 (2), we know that each $\alpha_j$ is contained within a finite interval, $[m_{\lambda/\psi}, M_{\lambda/\psi}]$ with $m_{\lambda/\psi} > 0$. Therefore $\sum_{i=1}^{d_x} \alpha_j^{1+\delta/2} \leq \sum_{i=1}^{d_x} M_{\lambda/\psi}^{1+\delta/2} = d_x M_{\lambda/\psi}^{1+\delta/2}$ and $\sum_{i=1}^{d_x} \alpha_j \geq \sum_{i=1}^{d_x} m_{\lambda/\psi} = d_x m_{\lambda/\psi}$, so that

$\frac{1}{\left(\sum_{i=1}^{d_x} \alpha_j\right)^{1+\delta/2}} \leq \frac{1}{(d_x m_{\lambda/\psi})^{1+\delta/2}}$. *(d)* Since $\delta > 0$ we have $d_x{}^{-\delta/2} \to 0$. The constants are non-zero and finite. □

## Appendix F. On non-linear and misspecified measurement models

F.1. **Polynomial measurement models.** We here consider polynomial measurement models in the context of the present paper. Such measurement models have long history, see R. McDonald (1967) for an early monograph on the subject. For simplicity, we consider only a very restricted class of models, though our arguments can be extended in various directions. Keeping the linear measurement model of eq. (2), suppose without loss of generality that the two first coordinates of $\xi$ and $\eta$ are of the form

$$(\theta_{1,x}, \theta_{1,x}^{m_x})', \qquad (\theta_{1,y}, \theta_{1,y}^{m_y})' \qquad m_x, m_y > 1$$

respectively. That is, there may be deterministic though non-linear relations between the coordinates of $\xi$ and $\eta$. This is a special case of a polynomial measurement model understood as a linear measurement model with non-linear deterministic connections between the latent variables (This is an old observation, see Chapter 3 in R. McDonald, 1967).

While treating such a polynomial measurement model as if it was linear may have certain drawbacks as the deterministic relationships between the latent variables are not taken into account e.g. when forming factor scores, such non-linear measurement models may be be compatible with the assumptions of the present paper. A core assumption in the paper is Assumption 3, where parameter identification is assumed. The error in variables parametrization of Yalcin and Amemiya (2001) can be used to secure this. Yalcin and Amemiya (2001) also provides an estimation method. Both identification (to fulfill Assumption 3) and an available estimation method (to apply the method in a practical setting), are taken as given in the following, as well as the remaining relevant assumptions.

Suppose given Assumption 1 and 3. By Lemma 1, $\Delta$ exists and is a left inverse of $\Lambda$. Therefore, the key correspondence

$$\Delta(\tilde{x}', \tilde{y}')' = (\xi', \eta')' + (r_\xi', r_\eta')'$$

still holds. In the presence of deterministic relationships between the coordinates of $\xi$ and $\eta$, it is usually not of interest to compute the full $\mathbb{E}[\eta|\xi = x]$. We now review why. For simplicity, we assume that $\xi$ and $\eta$ are bivariate, have quadratic measurement models, and therefore only contain $(\theta_{1,x}, \theta_{1,x}^2)'$ and $(\theta_{1,y}, \theta_{1,y}^2)'$ respectively.

Since $\theta_{1,x}^2$ is a function of $\theta_{1,x}$ we have that $\sigma(\theta_{1,x}, \theta_{1,x}^2) = \sigma(\theta_{1,x})$ by Lemma 11 (p. A76) since $\varphi(x) = x^2$ is a Borel function. Therefore, $\mathbb{E}[\eta|\theta_{1,x}, \theta_{1,x}^2] = \mathbb{E}[\eta|\theta_{1,x}]$. Therefore, the non-uniqueness (up to probability one) of conditional expectations now enter in a detrimental manner: Recall that $\mathbb{E}[\eta|\theta_{1,x}, \theta_{1,x}^2]$ is a function $H$ of $\theta_{1,x}$ and $\theta_{1,x}^2$. However, since $\sigma(\theta_{1,x}, \theta_{1,x}^2) = \sigma(\theta_{1,x})$, and $H(\theta_{1,x}, \theta_{1,x}^2) = \mathbb{E}[\eta|\theta_{1,x}] = \varphi(\theta_{1,x})$, for some function $\varphi$, we have that the functional mapping $H$ is highly non-unique. Indeed, any function $H$ such that $H(x_1, x_2) = \varphi(x_1)$ fulfills the requirement. While all such variables agree with probability one when evaluated at $\theta_{1,x}, \theta_{1,x}^2$, the functional relationship within the mappings can vary: For example, $H(x_1, x_2) = \varphi(x_1)$ and $H(x_1, x_2) = \varphi(\sqrt{|x_2|}\,\text{sign}(x_1))$ are two members of this class. It is therefore of interest to approximate $\varphi$ and not $H$.

The degeneracy induced by the deterministic relationship between $\theta_{1,x}$ and $\theta_{1,x}^2$ is also incompatible with Assumption 7 used in Proposition 4 unless the set $\mathcal{S}^\rho$ is chosen in a manner which takes the deterministic relationship into account. For example, in Assumption 7 we assume that $f_\xi$ is continuously differentiable in $\mathcal{S}^\rho$. For simplicity, assume that $\theta_{1,x} > 0$. The joint cumulative distribution of $\theta_{1,x}$ and

$\theta_{1,x}^2$ is by definition

$$\mathbb{P}(\theta_{1,x} \leq t_1, \theta_{1,x}^2 \leq t_2) \overset{(a)}{=} \mathbb{P}(\theta_{1,x} \leq t_1, \theta_{1,x} \leq \sqrt{t_2}) \overset{(b)}{=} \mathbb{P}\left(\theta_{1,x} \leq \min(t_1, \sqrt{t_2})\right) \overset{(c)}{=} F_{\theta_{1,x}}\left(\min(t_1, \sqrt{t_2})\right)$$
$$\overset{(d)}{=} \begin{cases} F_{\theta_{1,x}}(t_1) & \text{if } t_1 \leq \sqrt{t_2} \\ F_{\theta_{1,x}}(\sqrt{t_2}) & \text{if } t_1 > \sqrt{t_2} \end{cases}$$

*(a)* Recall that we assume that $\theta_{1,x} > 0$. *(b)* Recall that the comma in the probability stands for intersection. Therefore, the event can only happen if $\theta_{1,x}$ is less than or equal the smallest of the two upper limits. *(c)* $F_{\theta_{1,x}}$ is the cumulative distribution function of $\theta_{1,x}$, defined as $F_{\theta_{1,x}}(z) = P(\theta_{1,x} \leq z)$. *(d)* We consider the two cases where we know the value of the minimum.

We therefore take the two partial derivatives of the above joint cumulative distribution function of $\theta_{1,x}, \theta_{1,x}^2$, and find that its density is given by

$$f_\xi(t_1, t_2) = \begin{cases} f_{\theta_{1,x}}(t_1) & \text{if } t_1 \leq \sqrt{t_2} \\ f_{\theta_{1,x}}(\sqrt{t_2}) & \text{if } t_1 > \sqrt{t_2} \end{cases} = f_{\theta_{1,x}}(t_1)I\{t_1 \leq \sqrt{t_2}\} + f_{\theta_{1,x}}(\sqrt{t_2})I\{t_1 > \sqrt{t_2}\},$$

whose partial derivatives are

$$(\partial/\partial t_1)f_\xi(t_1, t_2) = f'_{\theta_{1,x}}(t_1)I\{t_1 \leq \sqrt{t_2}\}, \qquad (\partial/\partial t_2)f_\xi(t_1, t_2) = \frac{1}{2}f'_{\theta_{1,x}}(\sqrt{t_2})t_2^{-1/2}I\{t_1 > \sqrt{t_2}\}$$

Since these partial derivatives have jumps except in sets $(t_1, t_2)$ where $t_1$ and $\sqrt{t_2}$ have a fixed order, $f_\xi$ is not continuously differentiable even when $f_{\theta_{1,x}}$ is.

Another issue is that $\theta_{1,y}^2$ is considered as part of $\eta$ only since we are considering a non-linear measurement model from a linear perspective. We are interested in how $\theta_{1,y}$ varies with $\theta_{1,x}$, where $\theta_{1,y}$ is measured via a quadratic measurement equation. We therefore want to approximate

$$\mathbb{E}[\theta_{1,y}|\theta_{1,x} = x] \qquad \text{and not} \qquad \begin{pmatrix} \mathbb{E}[\theta_{1,y}|\theta_{1,x} = x_1] \\ \mathbb{E}[\theta_{1,y}^2|\theta_{1,x} = x_1] \end{pmatrix}.$$

Both issues can be dealt with by a minor modification of the framework of the paper. This can also be done in practice because the non-linear measurement model is provided by the user. Consider a linear transformation $P$ such that

$$(\dddot{\xi}', \dddot{\eta}')' := P\Delta(\tilde{x}', \tilde{y}')' = P(\xi', \eta')' + P(r_\xi', r_\eta')' = (\xi_1, \eta_1)' + (r_{1,\xi}, r_{1,\eta})'$$

removes the redundant variables, i.e., $\theta_{1,x}^2, \theta_{1,y}^2$. The statistical behavior of $\dddot{\xi}, \dddot{\eta}$ can be treated as population Bartlett scores and inputted into non-parametric regression methods as described above. This will approximate $\tilde{H}$ and not $H$.

F.2. **On measurement model misspecification.** We here investigate what happens when the measurement model is misspecified, focusing on a general non-linear measurement framework. We show that such misspecifications will be mixed in with the non-parametric trend estimate for $H$, and without assumptions leading to non-linear and possibly non-parametric identification of the measurement model and the structural relations, it is impossible to disentangle where contributions to the estimate of $H$ comes from. Such identification results appear not to be available in the literature, and seems difficult to reach.

Suppose the data-generating mechanism is such that

$$x = \mathbf{G}_x(\xi, \lambda^x) + \varepsilon_x, \qquad y = \mathbf{G}_y(\eta, \lambda^y) + \varepsilon_y.$$

where $\mathbf{G}_x(\cdot, \lambda^x) = ((G_x(\cdot, \lambda_i^x))_{i=1}^{d_x})'$, and $\mathbf{G}_y(\cdot, \lambda^y) = ((G_y(\cdot, \lambda_i^y))_{i=1}^{d_y})'$ are functions of the latent variables with parameter vectors $\lambda^x, \lambda^y$. The other parts of the data generating model is kept as is.

While the model as stated will not be identified without more assumptions, we may still suppose the data-generating mechanism is contained within this class. Notice that this general case also includes the linear case with a misspecified dimensionality, and even the case when the measurement model considered in the paper is correct.

Suppose we apply the non-parametric regression methods based on Bartlett scores as described earlier. This procedure will then estimate $\tilde{H}(z) = \mathbb{E}[\tilde{\Delta}_y \tilde{y} | \tilde{\Delta}_x \tilde{x} = z]$ where $\tilde{\Delta}_y, \tilde{\Delta}_x$ are the Bartlett transformations for the endogenous and exogenous measurement models respectively, defined via the population limits of a given possibly inconsistent estimator.

Since $\varepsilon_x$ is mean zero and independent to $x$, we still have a conclusion similar to Lemma 2. Let $\tilde{\mathbf{G}}_x(\xi, \lambda^x) = \mathbf{G}_x(\xi, \lambda^x) - \mathbb{E}x$ and $\tilde{\mathbf{G}}_y(\eta, \lambda^y) = \mathbf{G}_y(\eta, \lambda^y) - \mathbb{E}y$. Then

$$\begin{aligned} \mathbb{E}\left[\tilde{\Delta}_y \tilde{y} | \tilde{\Delta}_x \tilde{x}\right] &= \mathbb{E}\left[\tilde{\Delta}_y \tilde{\mathbf{G}}_y(\eta, \lambda^y) \big| \tilde{\Delta}_x [\tilde{\mathbf{G}}_x(\xi, \lambda^x) + \varepsilon_x]\right] \\ &= \mathbb{E}\left[\tilde{\Delta}_y \tilde{\mathbf{G}}_y(H(\xi) + \zeta, \lambda^y) \big| \tilde{\Delta}_x [\tilde{\mathbf{G}}_x(\xi, \lambda^x) + \varepsilon_x]\right]. \end{aligned} \tag{22}$$

This cannot in general be simplified further, but we see that the non-parametric trends are mixed together, and cannot easily be separated without strong assumptions. Especially, we see that the relationship $\tilde{\mathbf{G}}_y(H(\xi) + \zeta, \lambda^y)$ implies that non-linearities in $\tilde{\mathbf{G}}_y$ and $H$ cannot be separated without further assumptions, as any function pair with the same function composition leads to the same values of $\mathbb{E}[\tilde{\Delta}_y y | \tilde{\Delta}_x x]$.

With more assumptions, $\mathbb{E}[\tilde{\Delta}_y y | \tilde{\Delta}_x x]$ can be further simplified. As an illustration, we consider the case of normality.

**Example 6.** Suppose $\xi, \zeta, \varepsilon_x$ are zero mean and jointly normal, and $G_x$ is linear, say $G_x(\xi, \lambda^x) = \tilde{\Lambda}_x \xi$. Then, in eq. (22), we condition on $\tilde{\Delta}_x[\tilde{\Lambda}_x \xi + \varepsilon_x]$, which is normal. Then $\mathcal{Z} := (\tilde{\Delta}_x[\tilde{\Lambda}_x \xi + \varepsilon_x], \xi + \zeta)$ is jointly normal. This joint normality implies that when conditioning $\xi + \zeta$ on $\tilde{\Delta}_x[\tilde{\Lambda}_x \xi + \varepsilon_x]$ is again normal. We now use Lemma 8 (p. A75) to find this distribution.

Since $\mathrm{Cov}\,(\tilde{\Delta}_x \tilde{\Lambda}_x \xi + \tilde{\Delta}_x \varepsilon_x) = \tilde{\Delta}_x \tilde{\Lambda}_x \Phi \tilde{\Lambda}_x' \tilde{\Delta}_x' + \tilde{\Lambda}_x \Psi_x \tilde{\Lambda}_x' = \tilde{\Delta}_x (\tilde{\Lambda}_x \Phi \tilde{\Lambda}_x' + \Psi_x) \tilde{\Delta}_x'$, and $\mathrm{Cov}\,(\xi + \zeta) = \Phi + \Psi_\zeta$, where $\Psi_\zeta = \mathrm{Cov}\,(\zeta)$. Hence, we have that $\Sigma_{Y,X} = \mathrm{Cov}\,(\xi + \zeta, \tilde{\Delta}_x \tilde{\Lambda}_x \xi + \tilde{\Delta}_x \varepsilon_x) = \mathrm{Cov}\,(\xi, \tilde{\Delta}_x \tilde{\Lambda}_x \xi) + \mathrm{Cov}\,(\xi, \tilde{\Delta}_x \varepsilon_x) + \mathrm{Cov}\,(\zeta, \tilde{\Delta}_x \tilde{\Lambda}_x \xi) + \mathrm{Cov}\,(\zeta, \tilde{\Delta}_x \varepsilon_x) = \Phi \tilde{\Lambda}_x' \tilde{\Delta}_x'$, and analogously, $\Sigma_{X,Y} = (\Phi \tilde{\Lambda}_x' \tilde{\Delta}_x')' = \tilde{\Delta}_x \tilde{\Lambda}_x \Phi$. Therefore, $\mathcal{Z}$ is zero mean with covariance matrix

$$\Sigma = \begin{pmatrix} \tilde{\Delta}_x (\tilde{\Lambda}_x \Phi \tilde{\Lambda}_x' + \Psi_x) \tilde{\Delta}_x', & \tilde{\Delta}_x \tilde{\Lambda}_x \Phi \\ \Phi \tilde{\Lambda}_x' \tilde{\Delta}_x', & \Phi + \Psi_\zeta \end{pmatrix}.$$

From Lemma 8, we have that $\xi + \zeta | \tilde{\Lambda}_x \xi + \varepsilon_x$ is normal with mean

$$\mu(\tilde{\Delta}_x[\tilde{\Lambda}_x \xi + \varepsilon_x]) = \Phi \tilde{\Lambda}_x' \tilde{\Delta}_x' [\tilde{\Delta}_x (\tilde{\Lambda}_x \Phi \tilde{\Lambda}_x' + \Psi_x) \tilde{\Delta}_x']^{-1} \tilde{\Delta}_x [\tilde{\Lambda}_x \xi + \varepsilon_x]$$

and covariance

$$\tilde{\Sigma} = \Phi + \Psi_\zeta - \Phi \tilde{\Lambda}_x' \tilde{\Delta}_x' [\tilde{\Delta}_x (\tilde{\Lambda}_x \Phi \tilde{\Lambda}_x' + \Psi_x) \tilde{\Delta}_x']^{-1} \tilde{\Delta}_x \tilde{\Lambda}_x \Phi$$

Let $Z \sim N_{d_\xi}(0, I)$ and independent to $\xi$. Then for $\tilde{\Sigma}^{1/2} \tilde{(}\Sigma^{1/2})' = \tilde{\Sigma}$

$$\tilde{\Sigma}^{1/2} Z + \mu(\tilde{\Delta}_x[\tilde{\Lambda}_x \xi + \varepsilon_x])$$

is a stochastic representation of $\xi + \zeta | \tilde{\Delta}_x[\tilde{\Lambda}_x \xi + \varepsilon_x]$. Therefore,

$$\mathbb{E}\left[\tilde{\Delta}_y y | \tilde{\Delta}_x x\right] = \mathbb{E}_Z \left[\tilde{\Delta}_y \tilde{\mathbf{G}}_y \left(H\left(\tilde{\Sigma}^{1/2} Z + \mu(\tilde{\Delta}_x[\tilde{\Lambda}_x \xi + \varepsilon_x])\right), \lambda^y\right)\right]$$

where $\mathbb{E}_Z$ is expectation with respect only to $Z$.

**Example 7.** We continue Example 6, and verify its correctness in the case when the linear measurement model is in fact correct, and that

$$H(x) = Bx.$$

We compute directly that

$$\mathbb{E}[\tilde{\Delta}_y \tilde{\Lambda}_y \eta | \tilde{\Delta}_x \tilde{x}] = \mathbb{E}[\eta | \xi + r_\xi] = \mathbb{E}[B\xi + \zeta | \xi + r_\xi] = B\mathbb{E}[\xi | \xi + \tilde{\Delta}_x \varepsilon_x].$$

We have $\operatorname{Cov}(\xi + \tilde{\Delta}_x \varepsilon_x) = \Phi + \tilde{\Delta}_x \Psi_x \tilde{\Delta}_x'$. We also have $\operatorname{Cov}(\xi) = \Phi$. Further, $\operatorname{Cov}(\xi, \xi + \tilde{\Delta}_x \varepsilon_x) = \operatorname{Cov}(\xi, \xi) + \operatorname{Cov}(\xi, \tilde{\Delta}_x \varepsilon_x) = \Phi$, and $\operatorname{Cov}(\xi + \tilde{\Delta}_x \varepsilon_x, \xi) = \Phi' = \Phi$. Therefore, $(\xi + \tilde{\Delta}_x \varepsilon_x, \xi)$ is normal with zero mean and covariance matrix

$$\begin{pmatrix} \Phi + \tilde{\Delta}_x \Psi_x \tilde{\Delta}_x', & \Phi \\ \Phi, & \Phi \end{pmatrix}.$$

We therefore have that $\xi | \xi + \tilde{\Delta}_x \varepsilon_x$ is normal, with mean

$$\mu^\circ(\xi + \tilde{\Delta}_x \varepsilon_x) = \Phi(\Phi + \tilde{\Delta}_x \Psi_x \tilde{\Delta}_x')^{-1}(\xi + \tilde{\Delta}_x \varepsilon_x).$$

$$\begin{aligned} \mathbb{E}[\tilde{\Delta}_y \tilde{\Lambda}_y \eta | \Delta_x x] &= B\mathbb{E}[\xi | \xi + \tilde{\Delta}_x \varepsilon_x] \\ &= B\Phi(\Phi + \tilde{\Delta}_x \Psi_x \tilde{\Delta}_x')^{-1}(\xi + \tilde{\Delta}_x \varepsilon_x). \end{aligned}$$

We now verify that the expression from Example 6 is the same as found directly above. We have that $\tilde{\Delta}_y \tilde{\mathbf{G}}_y$ and $\tilde{\Delta}_x \tilde{\mathbf{G}}_x$ becomes the identity by Lemma 1. Then, using that $H(x) = Bx$, we get that

$$\begin{aligned} \mathbb{E}[\tilde{\Delta}_y y | \tilde{\Delta}_x x] &= \mathbb{E}_Z[\tilde{\Delta}_y \tilde{\mathbf{G}}_y (H(\tilde{\Sigma}^{1/2} Z + \mu(\tilde{\Delta}_x[\tilde{\Lambda}_x \xi + \varepsilon_x]), \lambda^y)] \\ &= \mathbb{E}_Z[B(\tilde{\Sigma}^{1/2} Z + \mu(\tilde{\Delta}_x[\tilde{\Lambda}_x \xi + \varepsilon_x])] \\ &= B\mu(\tilde{\Delta}_x[\tilde{\Lambda}_x \xi + \varepsilon_x]) \\ &= B\Phi\tilde{\Lambda}_x' \tilde{\Delta}_x' [\tilde{\Delta}_x (\tilde{\Lambda}_x \Phi \tilde{\Lambda}_x' + \Psi_x) \tilde{\Delta}_x']^{-1} \tilde{\Delta}_x [\tilde{\Lambda}_x \xi + \varepsilon_x] \\ &= B\Phi[\Phi + \tilde{\Delta}_x \Psi_x \tilde{\Delta}_x']^{-1}[\xi + \tilde{\Delta}_x \varepsilon_x], \end{aligned}$$

The last equality follows as $\tilde{\Delta}_x$ is a left inverse of $\tilde{\Lambda}_x$, which also implies that $\tilde{\Lambda}_x' \tilde{\Delta}_x' = (\tilde{\Delta}_x \tilde{\Lambda}_x)' = I$.

We see that the expressions match with the earlier calculation.

Let us seize the occasion to verify the conclusion of Proposition 4 in this direct and simple case. Since $\tilde{\Delta}_x x = \ddot{\xi}$, we have

$$H_{d_x}(x) = \mathbb{E}[\ddot{\eta} | \ddot{\xi} = x] = \mathbb{E}[\eta | \ddot{\xi} = x] = B\Phi[\Phi + \tilde{\Delta}_x \Psi_x \tilde{\Delta}_x']^{-1} x.$$

Since $\mathbb{E}[\eta|\xi] = \mathbb{E}[B\xi + \zeta|\xi] = B\mathbb{E}[\xi|\xi] + \mathbb{E}[\zeta|\xi] = B\xi$ we have

$$H(x) = \mathbb{E}[\eta | \xi = x] = Bx.$$

Therefore, for any set $\mathcal{S}^\rho$, we have

$$\begin{aligned} \sup_{x \in \mathcal{S}^\rho} |H_{d_x}(x) - H(x)| &= \sup_{x \in \mathcal{S}^\rho} |B\Phi[\Phi + \tilde{\Delta}_x \Psi_x \tilde{\Delta}_x']^{-1} x - Bx| \\ &= \sup_{x \in \mathcal{S}^\rho} \left| B\left(\Phi[\Phi + \tilde{\Delta}_x \Psi_x \tilde{\Delta}_x']^{-1} - I\right) x \right|. \end{aligned}$$

By Proposition 2, we have $\tilde{\Delta}_x \Psi_x \tilde{\Delta}_x' = \operatorname{Cov} r_\xi = \left(\Lambda_x' \Psi_x^{-1} \Lambda_x'\right)^{-1}$, which goes to zero as $d_x$ increases under e.g. the assumptions of Proposition 3. Since matrix inversion is continuous, we see that $\Phi[\Phi + \tilde{\Delta}_x \Psi_x \tilde{\Delta}_x']^{-1} \to \Phi[\Phi]^{-1} = I$ and so

$$B\left(\Phi[\Phi + \tilde{\Delta}_x \Psi_x \tilde{\Delta}_x']^{-1} - I\right) \to B(I - I) = \mathbf{0}.$$

Therefore, as long as $\mathcal{S}^\rho$ has finite extension, $\sup_{x\in\mathcal{S}^\rho} |H_{d_x}(x) - H(x)|$ goes to zero. If $\mathcal{S}^\rho$ has infinite extension, the supremum is infinite for each $d_x$. This is interesting with respect to the conditions identified in Appendix E.2 (p.A44) where the required convergence concerning $\omega$ is shown under Assumption 10, which include an assumption of finite extension. We therefore see that even in this simplest of cases, finite extension is actually needed. □

When specifying $\mathbf{G}_y$, concrete expressions for $H_{d_x}$ can be reached, as we now illustrate.

**Example 8.** Suppose that

$$x_i = \lambda^x_{i,1}\xi + \varepsilon_{i,x}, \qquad i = 1, 2, \ldots, d_x,$$
$$y_j = \lambda^y_{j,1}\eta + \lambda^y_{j,2}\eta^2 + \varepsilon_{j,x}, \qquad j = 1, 2, \ldots, d_y,$$

where the structural trend is linear and given by

$$\eta = \xi + \zeta.$$

All error terms have zero mean, and are independent of each other as well as to $\xi$. Suppose $\xi$ and $\zeta$ are standardized. For computational tractability, we assume that all latent random variables are jointly normal.

Suppose we estimate a single-factor linear factor model. We will show that $\mathbb{E}[\ddot{\eta}|\ddot{\xi} = x]$ is a second degree polynomial in $x$. The missing non-linearity in the measurement model therefore shows up in the non-parametric trend estimates of the structural variables.

As earlier, the misspecified single-factor linear factor model without correlated errors is assumed estimated using a specific estimator, such as the normal theory ML or the GLS estimator. The asymptotic limit that this estimator converges to as the sample size increases will be denoted by $(\tilde{\lambda}^x_i), (\tilde{\lambda}^y_i)$, and similarly for error variances estimated by the misspecified model, denoted by $(\tilde{\psi}_{ii,x})_{i=1}^{d_x}, (\tilde{\psi}_{ii,y})_{i=1}^{d_y}$ for respectively error variances of the measurement error of $\xi$ and $\eta$.

Let $L_x, L_y$ be the linear operators defined by their application to sequences $c_x = (c_1, c_2, \ldots, c_{d_x})$ and $c_y = (c_1, c_2, \ldots, c_{d_y})$ through the operation

$$L_x c_x = \left(\sum_{k=1}^{d_x} \frac{(\tilde{\lambda}^x_k)^2}{\tilde{\psi}_{kk,x}}\right)^{-1} \sum_{i=1}^{d_x} \tilde{\lambda}^x_i (\tilde{\psi}_{ii,x})^{-1} c_i$$
$$L_y c_y = \left(\sum_{k=1}^{d_y} \frac{(\tilde{\lambda}^y_k)^2}{\tilde{\psi}_{kk,y}}\right)^{-1} \sum_{i=1}^{d_y} \tilde{\lambda}^y_i (\tilde{\psi}_{ii,y})^{-1} c_i.$$

Then, from Lemma 4, the Bartlett factor scores for $\xi$ and $\eta$ respectively, are

$$L_x\tilde{x}, \qquad \tilde{x}_i = x_i - \mathbb{E}x_i$$
$$L_y\tilde{y}, \qquad \tilde{x}_i = y_i - \mathbb{E}y_i.$$

Now $\mathbb{E}x_i = 0$ and $\mathbb{E}y_i = \mathbb{E}\lambda^y_{i,2}\eta^2 = \lambda^y_{i,2} \operatorname{Var} \eta = \lambda^y_{i,2} \operatorname{Var}(\xi + \zeta) = \lambda^y_{i,2}[\operatorname{Var}(\xi) + \operatorname{Var}(\zeta)] = 2\lambda^y_{i,2}$.

The linearity of $L_x$ implies that

$$L_x\tilde{x} = \underbrace{[L_x(\lambda^x_{i,1})_{i=1}^{d_x}]}_{=:\tilde{\lambda}_{S,x}}\xi + \underbrace{L_x\varepsilon_{i,x}}_{=:\varepsilon_{S,x}}$$
$$= \tilde{\lambda}_{S,x}\xi + \varepsilon_{S,x}.$$

Similarly,

$$\begin{aligned}
L_y\tilde{y} &= L_x(-2\lambda^y_{i,2} + \lambda^y_{i,1}\eta + \lambda^y_{i,2}\eta^2 + \varepsilon_{i,x})_{i=1}^{d_x} \\
&= \underbrace{L_x(-2\lambda^y_{i,2})_{i=1}^{d_x}}_{=:\tilde{\lambda}_{S,0,y}} + \underbrace{L_x(\lambda^y_{i,1})_{i=1}^{d_x}}_{=:\tilde{\lambda}_{S,1,y}}\eta + \underbrace{L_x(\lambda^y_{i,2})_{i=1}^{d_x}}_{=:\tilde{\lambda}_{S,2,y}}\eta^2 + \underbrace{L_x(\varepsilon_{i,x})_{i=1}^{d_x}}_{=:\varepsilon_{S,y}} \\
&= \tilde{\lambda}_{S,0,y} + \tilde{\lambda}_{S,1,y}\eta + \tilde{\lambda}_{S,2,y}\eta^2 + \varepsilon_{S,y}.
\end{aligned}$$

From our assumptions, $\varepsilon_{S,y}, \varepsilon_{S,x}$ have zero mean and are independent to each other and to $\xi$. The LOESS estimator based on the Bartlett scores from the misspecified model will therefore asymptotically reach

$$\begin{aligned}
\mathbb{E}[L_y\tilde{y}|L_x\tilde{x}] &= \tilde{\lambda}_{S,0,y} + \tilde{\lambda}_{S,1,y}\mathbb{E}[\eta|\tilde{\lambda}_{S,x}\xi + \varepsilon_{S,x}] + \tilde{\lambda}_{S,2,y}\mathbb{E}[\eta^2||\tilde{\lambda}_{S,x}\xi + \varepsilon_{S,x}] + \mathbb{E}[\varepsilon_{S,y}|\tilde{\lambda}_{S,x}\xi + \varepsilon_{S,x}] \\
&= \tilde{\lambda}_{S,0,y} + \tilde{\lambda}_{S,1,y}\mathbb{E}[\xi + \zeta|\tilde{\lambda}_{S,x}\xi + \varepsilon_{S,x}] + \tilde{\lambda}_{S,2,y}\mathbb{E}[\xi^2 + 2\xi\zeta + \zeta^2||\tilde{\lambda}_{S,x}\xi + \varepsilon_{S,x}] \\
&= \tilde{\lambda}_{S,0,y} + \tilde{\lambda}_{S,1,y}\mathbb{E}[\xi|\tilde{\lambda}_{S,x}\xi + \varepsilon_{S,x}] + \tilde{\lambda}_{S,2,y}\mathbb{E}[\xi^2|\tilde{\lambda}_{S,x}\xi + \varepsilon_{S,x}] + 2\mathbb{E}[\xi\zeta|\tilde{\lambda}_{S,x}\xi + \varepsilon_{S,x}] \\
&\quad + \mathbb{E}[\zeta^2|\tilde{\lambda}_{S,x}\xi + \varepsilon_{S,x}].
\end{aligned}$$

Since $\zeta^2$ is independent to $\tilde{\lambda}_{S,x}\xi + \varepsilon_{S,x}$, we use eq. (27) (p. A75) to get that $\mathbb{E}[\zeta^2|\tilde{\lambda}_{S,x}\xi + \varepsilon_{S,x}] = \mathbb{E}\zeta^2 = 1$.

Since $\tilde{\lambda}_{S,x}\xi + \varepsilon_{S,x}$ is a function of $\xi$ and $\varepsilon_{S,x}$, we have that $\sigma(\tilde{\lambda}_{S,x}\xi + \varepsilon_{S,x}) \subseteq \sigma(\xi, \varepsilon_{S,x})$. Therefore, we apply Theorem 4 (p. A74) and get that $\mathbb{E}[\xi\zeta|\tilde{\lambda}_{S,x}\xi + \varepsilon_{S,x}] = \mathbb{E}[\mathbb{E}[\xi\zeta|\xi, \varepsilon_{S,x}]|\tilde{\lambda}_{S,x}\xi + \varepsilon_{S,x}]$. Since $\xi$ is $\sigma(\xi, \varepsilon_{S,x})$-measurable, $\mathbb{E}[\xi\zeta|\xi, \varepsilon_{S,x}] = \xi\mathbb{E}[\zeta|\xi, \varepsilon_{S,x}]$ (use Theorem 3 on p. A74). Since $\zeta$ is independent to both $\xi, \varepsilon_{S,x}$, we use eq. (27) (p. A75) to get that $\mathbb{E}[\zeta|\xi, \varepsilon_{S,x}] = \mathbb{E}[\zeta] = 0$. Therefore, $\mathbb{E}[\xi\zeta|\tilde{\lambda}_{S,x}\xi + \varepsilon_{S,x}] = 0$.

Since $(\tilde{\lambda}_{S,x}\xi + \varepsilon_{S,x}, \xi)$ is jointly normal, we use Lemma 8 to see that

$$\mathbb{E}[\xi|\tilde{\lambda}_{S,x}\xi + \varepsilon_{S,x} = z] = \mathrm{Cov}\left(\xi, \tilde{\lambda}_{S,x}\xi + \varepsilon_{S,x}\right)\mathrm{Var}\left(\tilde{\lambda}_{S,x}\xi + \varepsilon_{S,x}\right)^{-1}x,$$

which is linear in $x$.

Finally, since

$$\mathrm{Var}\left[\xi|\tilde{\lambda}_{S,x}\xi + \varepsilon_{S,x}\right] = \mathbb{E}[\xi^2|\tilde{\lambda}_{S,x}\xi + \varepsilon_{S,x}] - (\mathbb{E}[\xi|\tilde{\lambda}_{S,x}\xi + \varepsilon_{S,x}])^2$$

we get that

$$\mathbb{E}[\xi^2|\tilde{\lambda}_{S,x}\xi + \varepsilon_{S,x} = z] = \mathrm{Var}\left[\xi|\tilde{\lambda}_{S,x}\xi + \varepsilon_{S,x} = z\right] + (\mathbb{E}[\xi|\tilde{\lambda}_{S,x}\xi + \varepsilon_{S,x} = z])^2.$$

Again, since $(\tilde{\lambda}_{S,x}\xi + \varepsilon_{S,x}, \xi)$ is jointly normal, and therefore $\mathrm{Var}\left[\xi|\tilde{\lambda}_{S,x}\xi + \varepsilon_{S,x}\right]$ is non-stochastic from Lemma 8, it will not vary with $x$. Since we have already shown $\mathbb{E}[\xi|\tilde{\lambda}_{S,x}\xi + \varepsilon_{S,x} = z]$ to be linear in $z$, we conclude that $\mathbb{E}[\xi^2|\tilde{\lambda}_{S,x}\xi + \varepsilon_{S,x} = z]$ is a second degree polynomial in $z$. In conclusion, this shows that also $\mathbb{E}[L_y\tilde{y}|L_x\tilde{x} = z]$ is a second degree polynomial in $z$, with coefficients deducible from the above argument. □

F.3. **Simulation illustrations with measurement model misspecifications.** In this section we provide numerical illustrations of the effect of having a nonlinear factor model, when assuming a linear measurement model in the estimation of $H$. We consider only two empirical estimators: the LOESS(BFS) method and the BSpline(NLFS) method.

For both estimators, the examples show that for low degrees of nonlinearity in the measurement model, the structural part of the model is adequately estimated, while for stronger degree of nonlinearity, larger influences on the structural part are vivid. The supplemental material includes complete computer code and all parameter values.

Example 9 shows the effect of a nonlinear effect in the measurement as well as the structural part of the model which result in an estimated linear trend. Example 10 and 11 show that a nonlinear misspecification in the measurement model can result in estimates of the structural trend that is erroneously nonlinear (the true trend is linear). This happens both for the LOESS(BFS) and the the nonlinear factor scores of Kelava et al. (2017) in the BSpline(NLFS), which both assume a correctly specified and linear measurement model.

**Example 9.** Consider the measurement models

$$x_i = \mu_{x,i} + \lambda_{x,i,1}\xi + \alpha\lambda_{x,i,2}\xi^2 + \varepsilon_{x,i},\ i = 1,2,3, \quad y_j = \mu_{y,j} + \lambda_{y,j,1}\eta + \varepsilon_{y,j,2},\ j = 1,2,3, \tag{23}$$

which is a nonlinear factor model for $x_i$, where $\alpha > 0$ controls the degree of nonlinearity. For $\alpha = 0$ this is a linear factor model. We assume a quadratic structural model

$$\eta = \alpha_\eta + \xi + \xi^2 + \zeta,$$

and simulate all variables to be normal with $\xi \sim \mathcal{N}(0, .5)$, $\zeta \sim \mathcal{N}(0, .3)$, $\alpha_\eta = -.5$ so that $\mathbb{E}\eta = 0, \mathrm{Var}\ \eta = 1.3$ and set the factor loadings as $\lambda_{x,1,1} = \lambda_{y,1,1} = 1$, $\lambda_{x,2,1} = \lambda_{y,2,1} = .8$, and $\lambda_{x,3,1} = \lambda_{y,3,1} = .7$. For the nonlinear part in $\xi_1^2$ we set $\lambda_{x,1,2} = 1.3$, $\lambda_{x,2,2} = 1$, and $\lambda_{x,3,2} = .4$. Further, we set $\mathrm{Var}\ \varepsilon_{x,i}$ and $\mathrm{Var}\ \varepsilon_{y,j}$ so that the reliabilities are constant across all values of $\alpha$ with $Rel[x_1] = Rel[y_1] = .81, Rel[x_2] = Rel[y_2] = .64, Rel[x_3] = Rel[y_3] = .49$. The reliability are computed as

$$Rel[x_i] = \frac{\lambda^2_{x,i,1}\ \mathrm{Var}\ \xi + 2\alpha^2\lambda^2_{x,i,2}(\mathrm{Var}\ \xi)^2}{\lambda^2_{x,i,1}\ \mathrm{Var}\ \xi + 2\alpha^2\lambda^2_{x,i,2}(\mathrm{Var}\ \xi)^2 + \mathrm{Var}\ \varepsilon_{x,i}} \text{ and } Rel[y_j] = \frac{\lambda^2_{y,i,1}\ \mathrm{Var}\ \eta}{\lambda^2_{y,i,1}\ \mathrm{Var}\ \eta + \mathrm{Var}\ \varepsilon_{y,i}}.$$

Further, $\mu_{x,i} = -.5\lambda_{x,i}$ so that $\mathbb{E}x_i = 0,\ i = 1,2,3$.

We used two methods of the original simulation study. We estimated the (partly wrongly specified) linear factor model

$$x_i = \mu_{x,i} + \tilde{\lambda}_{x,i}\tilde{\xi} + \tilde{\varepsilon}_{x,i},\ i = 1,2,3, \quad y_j = \mu_{y,j} + \lambda_{y,j}\tilde{\eta} + \tilde{\varepsilon}_{y,j},\ j = 1,2,3,$$

and used this linear measurement model to estimate $H$ non-parametrically using LOESS(BFS) based on the Bartlett (1937) factor scores. Further, we estimated BSpline(NLFS) using the nonlinear factor scores of Kelava et al. (2017). Figure 23 shows that for small $\alpha$ (i.e., small nonlinearity in the measurement part of the model), the estimates for $H$ clearly suggest a nonlinear (quadratic trend) for both methods. In contrast, for large $\alpha$, i.e., $\alpha = 1$, a trend close to linear is suggested. This happens for LOESS(BFS) as well as BSpline(NLFS).

□

**Example 10.** The second example is almost identical to Example 9, except, we assume a linear structural model

$$\eta = \sqrt{2}\xi + \zeta,$$

where, again, all parameters were chosen so that all reliabilities are identical across different values of $\alpha$ and $\mathbb{E}\eta = 0, \mathrm{Var}\ \eta = 1.3$. We (again) estimated the (partly wrongly specified) linear factor model

$$x_i = \mu_{x,i} + \tilde{\lambda}_{x,i}\tilde{\xi} + \tilde{\varepsilon}_{x,i},\ i = 1,2,3, \quad y_j = \mu_{y,j} + \lambda_{y,j}\tilde{\eta} + \tilde{\varepsilon}_{y,j},\ j = 1,2,3,$$

and used this linear measurement model to estimate $H$ non-parametrically using LOESS(BFS) based on the Bartlett (1937) factor scores. Further, we estimated BSpline(NLFS) using the nonlinear factor scores of Kelava et al. (2017). Figure 24 suggest a linear trend for small values of $\alpha$, i.e., small nonlinear effects in the measurement part of the model. For $\alpha = 1$ a clear nonlinear trend is evident, which has slower than linear growth.

□

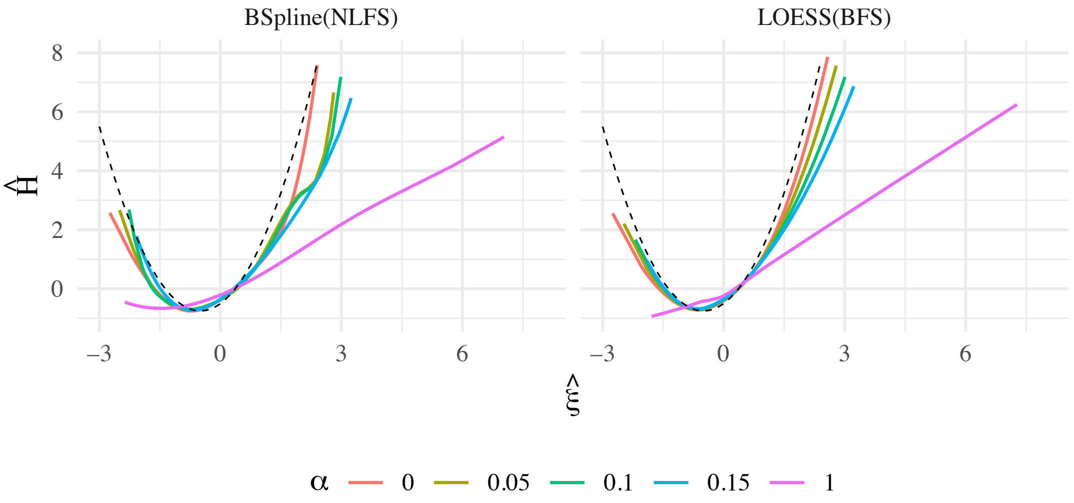


FIGURE 23. *Predicted trends for quadratic structural model using LOESS(BFS) and BSpline(NLFS) for different values of $\alpha$ representing different degree of nonlinearity (quadratic) in the factor model for $\xi$ for $n = 1000$.*

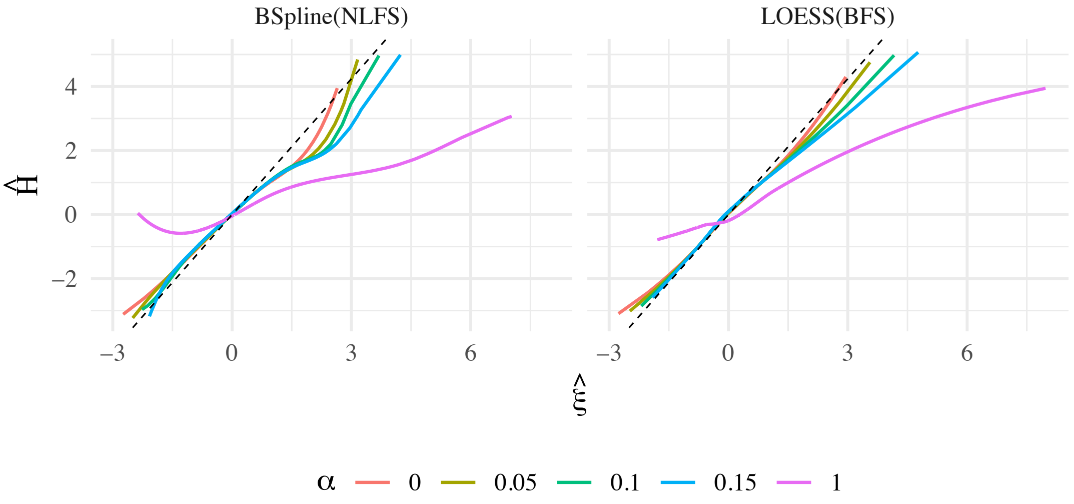


FIGURE 24. *Predicted trends for linear structural model using LOESS(BFS) and BSpline(NLFS) for different values of $\alpha$ representing different degree of nonlinearity (quadratic) in the factor model for $\xi$.*

**Example 11.** In this example we consider a linear measurement model for the exogenous part of the model, but a nonlinear one for the endogenous part of the model. Consider the measurement models

$$x_i = \mu_{x,i} + \lambda_{x,i,1}\xi + \varepsilon_{x,i}, \ i = 1, 2, 3, \quad y_j = \mu_{y,j} + \lambda_{y,j,1}\eta + \alpha\lambda_{y,j,3}\eta^3 + \varepsilon_{y,j}, \ j = 1, 2, 3, \tag{24}$$

which is a nonlinear factor model for $y_i$, where $\alpha > 0$ controls the degree of nonlinearity. For $\alpha = 0$ this is a linear factor model. We assume a linear structural model

$$\eta = \xi + \zeta,$$

and simulate all variables to be normal with $\xi \sim \mathcal{N}(0, .5)$, $\zeta \sim \mathcal{N}(0, .5)$, so that $\mathbb{E}\eta = 0, \text{Var } \eta = 1$ and set the factor loadings of the linear effects as in Example 9 and 10. For the nonlinear part in $\eta_1^3$ we set $\lambda_{y,1,3} = .2$, $\lambda_{y,2,3} = .15$, and $\lambda_{y,3,3} = .1$. Further, we set Var $\varepsilon_{x,i}$ and Var $\varepsilon_{y,j}$ so that the reliabilities are constant across all values of $\alpha$ with $Rel[x_1] = Rel[y_1] = .81, Rel[x_2] = Rel[y_2] = .64, Rel[x_3] = Rel[y_3] = .49$. The reliability are computed as

$$Rel[x_i] = \frac{\lambda_{y,i,1}^2 \text{ Var } \xi}{\lambda_{y,i,1}^2 \text{ Var } \xi + \text{Var } \varepsilon_{y,i}}$$

and

$$Rel[y_i] = \frac{\lambda_{y,i,1}^2 \text{ Var } \eta + \lambda_{y,i,3}^2 \alpha^2 \text{ Var } \eta^3 + 2\alpha\lambda_{y,i,1}\lambda_{y,i,3} \text{ Cov } [\eta, \eta^3]}{\lambda_{y,i,1}^2 \text{ Var } \eta + \lambda_{y,i,3}^2 \alpha^2 \text{ Var } \eta^3 + 2\alpha\lambda_{y,i,1}\lambda_{y,i,3} \text{ Cov } [\eta, \eta^3] + \text{Var } \varepsilon_{y,i}}.$$

Further, $\mu_{x,i} = \mu_{y,i} = 0$ so that $\mathbb{E}x_i = \mathbb{E}y_i = 0, \ i = 1, 2, 3$. Note that for a standardized normal $\eta$ we have Var $\eta^3 = \mathbb{E}\eta^6 = 15$ and Cov $[\eta, \eta_3] = \mathbb{E}\eta^4 = 3$.

We (again) estimated the (partly wrongly specified) linear factor model

$$x_i = \mu_{x,i} + \tilde{\lambda}_{x,i}\tilde{\xi} + \tilde{\varepsilon}_{x,i}, \ i = 1, 2, 3, \quad y_j = \mu_{y,j} + \lambda_{y,j}\tilde{\eta} + \tilde{\varepsilon}_{y,j}, \ j = 1, 2, 3,$$

and used this linear measurement model to estimate $H$ non-parametrically using LOESS(BFS) based on the Bartlett (1937) factor scores. Further, we estimated BSpline(NLFS) using the nonlinear factor scores of Kelava et al. (2017). Figure 25 suggests a linear trend for small values of $\alpha$, i.e., small nonlinear effects in the measurement part of the model. For $\alpha = 1$ a clear nonlinear trend is evident with growth quicker than linear.

For $\alpha \neq 0$, the estimates of $H$ are affected by the misspecified non-linear measurement model. For $\alpha = 1$, the estimated non-linear trend appears to be a third order polynomial. We conjecture that this is due to the same type of effect as shown analytically in Example 8 (p. A63).

□

## Appendix G. Independence between $\xi$ and $\varepsilon$ is incompatible with ordinal data

Suppose a factor model $X = \Lambda_x\xi + \varepsilon_x$, where $X$ has ordinal coordinates and $\xi$ continuous. Since then $\Lambda_x\xi$ is continuous, we can apply the following Lemma (Lemma 6 below) coordinate by coordinate to $X$ and see that the coordinates of $\varepsilon_x$ cannot be independent to $\sum_j \lambda_{k,j}^x \xi_j$, which implies that $\varepsilon_x$ is not independent to $\xi$.

This conclusion seems intuitively clear: Since $X$ can only take on a finite number of values, but $\xi$ can take on a continuum of possible values, $\varepsilon_x = X - \Lambda_x\xi$ has to compensate for the continuity of $\xi$ whose influence on $X$ is filtered in such a way that the result of $\Lambda_x\xi + \varepsilon_x$ only takes on a finite number of values. This compensation leads to dependence between $\varepsilon_x$ and $\xi$.

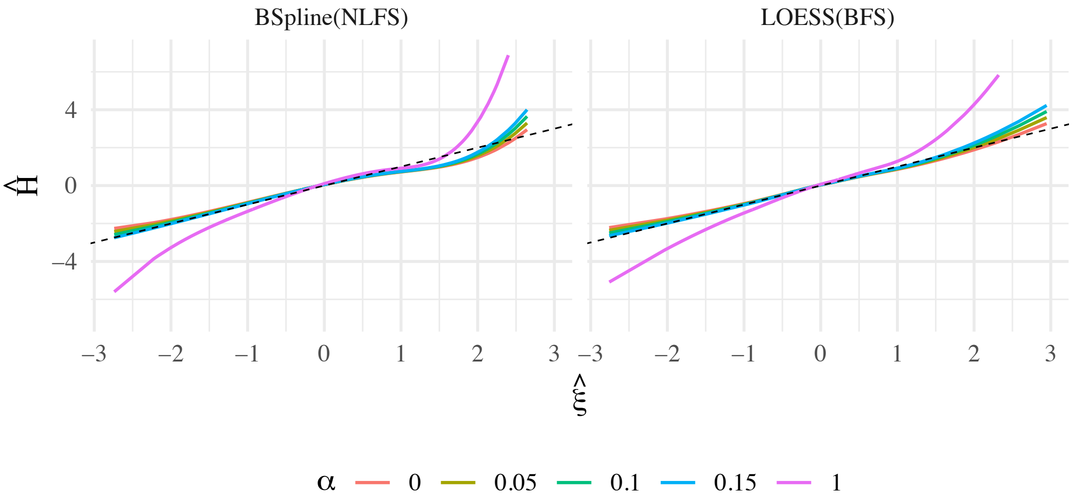


FIGURE 25. *Predicted trends for a linear structural model using LOESS(BFS) and BSpline(NLFS) for different values of $\alpha$ representing different degree of nonlinearity (cubic) in the factor model for $\xi$.*

G.1. **A simple illustration.** Let us look at this lack of independence in more detail using a prototypical factor model for univariate $x, \xi$, and $\varepsilon_x$, namely

$$x = \mu_x + \lambda_x \xi + \varepsilon_x.$$

Here, $\mu_x, \lambda_x$ are numbers, and $\xi$ is an arbitrary continuous random variable.

As an extreme though practically relevant case, we suppose $x$ is a binary variable. For concreteness, suppose $x$ fulfills the equations of an ordinal factor model

$$x = I\{\xi + U > \tau\},$$

where $\xi, U$ are independent. If the distributions of $\xi, U$ are chosen, we may use them to choose constants $\lambda_x, \mu_x$ so that the identifying restrictions $\mathrm{Cov}\,(\xi, \varepsilon_x) = 0$ and $\mathbb{E}\varepsilon_x = 0$ are fulfilled. To see this, notice that

$$0 = \mathrm{Cov}\,(\xi, \varepsilon_x) = \mathrm{Cov}\,(\xi, x - \lambda_x \xi) = \mathrm{Cov}\,(\xi, x) - \lambda_x \,\mathrm{Var}\,\xi$$

which gives

$$\lambda_x = \frac{\mathrm{Cov}\,(\xi, x)}{\mathrm{Var}\,\xi}$$

We then choose $\mu_x$ so that $\mathbb{E}\varepsilon_x = 0$, which is achieved by $\mu_x = \mathbb{E}x - \lambda_x \mathbb{E}\xi$.

Now consider the formula for $\varepsilon_x$, which is

$$\varepsilon = x - \mu_x - \lambda_x \xi = I\{\xi + U > \tau\} - \mu_x - \lambda_x \xi.$$

Simulated values when $\xi \sim N(0,1), U \sim N(0,1), \tau = 0$ are visualized in Figure 26, showing extreme negative dependence with a perfect locally linear trend $-\mu_x - \lambda_x x$ randomly distorted by adding 1 when $\xi + U \leq 0$. The Pearson correlation is zero by design.

While a general discussion of this topic is outside the scope of the present paper, we warn against using the above argument as a justification for treating ordinal data as continuous, among other reasons because the error terms from different ordinal variables will be correlated unless more restrictions are imposed. This implies that standard identification criteria for confirmatory factor models are not fulfilled. Therefore, the binary variables do not in fact follow a confirmatory factor model in a meaningful way, and the statistical properties of the binary variables will therefore not be derivable from general results on confirmatory factor models.

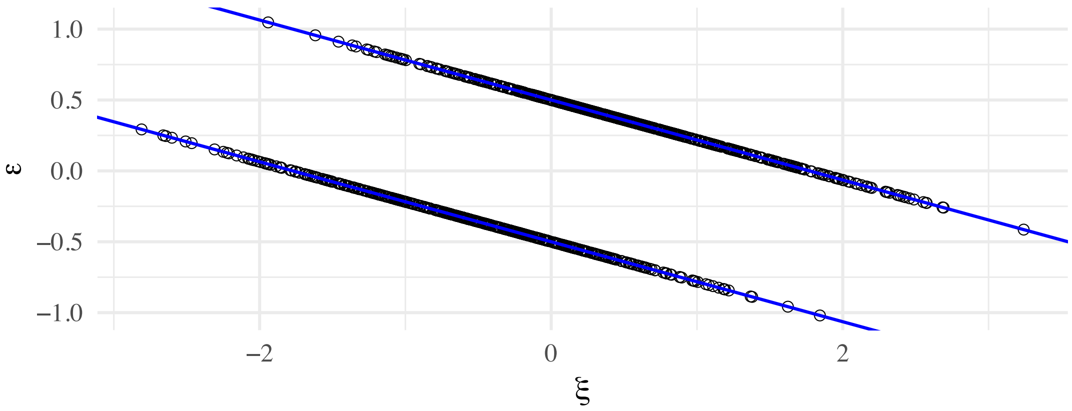


FIGURE 26. Scatterplot between $\varepsilon$ and $\xi$ in the illustrative binary case, with trend lines in blue.

G.2. **The general lemma.**

**Lemma 6.** *Suppose univariate $x$ attains only a countable number of values, and*

$$x = \xi + \varepsilon_x$$

*where $\xi$ is a continuous random variable and $\varepsilon_x$ is a random variable. Then $\xi$ and $\varepsilon_x$ cannot be independent.*

*Proof.* Let the unique attainable values of $x$ be $a_1, a_2, \ldots$. Suppose, to reach a contradiction, that $\xi$ and $\varepsilon_x$ are independent. Then, for $k = a_j$ for $j \geq 1$, we have by the assumed independence that

$$\begin{aligned} \mathbb{P}(x = k) &= \mathbb{P}(\xi + \varepsilon_x = k) = \mathbb{E}\mathbb{P}(\xi + \varepsilon_x = k | \xi) \\ &\overset{(a)}{=} \int_{\mathbb{R}} \mathbb{P}(z + \varepsilon_x = k) f_\xi(z) \, \mathrm{d}z \\ &= \int_{\mathbb{R}} \mathbb{P}(\varepsilon_x = k - z) f_\xi(z) \, \mathrm{d}z. \end{aligned}$$

*(a)* This is the step that follows by independence. It is justified e.g. by Lemma 4.11 in Kallenberg (2021).

Therefore,

$$1 = \sum_{j\geq 1} \mathbb{P}(x = a_j) = \int_{\mathbb{R}} \sum_{j\geq 1} \mathbb{P}(\varepsilon = a_j - z) f_\xi(z)\,\mathrm{d}z$$
$$= \int_{\mathbb{R}} \mathbb{P}(\cup_{j\geq 1}\{\varepsilon = a_j - z\}) f_\xi(z)\,\mathrm{d}z.$$

Since $0 \leq \mathbb{P}(\cup_{j\geq 1}\{\varepsilon = a_j - z\}) \leq 1$ and $\int_{\mathbb{R}} f_\xi(z)\,\mathrm{d}z = 1$ we must have $\mathbb{P}(\cup_{j\geq 1}\{\varepsilon = a_j - z\}) = 1$ for all $z$ such that $f_\xi(z) > 0$ except on a Lebesgue measure zero. To see this, notice that otherwise $1 = \int_{\mathbb{R}} \mathbb{P}(\cup_{j\geq 1}\{\varepsilon = a_j - z\}) f_\xi(z)\,\mathrm{d}z < \int_{\mathbb{R}} f_\xi(z)\,\mathrm{d}z = 1$ which is impossible.

Now the support $S = \{z : f_\xi(z) > 0\}$ of $f_\xi(z)$ must have positive Lebesgue measure, since otherwise it is impossible that $\int_{\mathbb{R}} f_\xi(z)\,\mathrm{d}z = 1$. We therefore conclude that $\mathbb{P}(\cup_{j=1}^{m}\{\varepsilon = a_j - z\}) = 1$ for all $z \in S \setminus M$ where $M$ has Lebesgue measure zero. Since the Lebesgue measure of $\tilde{S} := S \setminus M$ equals that of $S$ which is positive, also $\tilde{S}$ has positive Lebesgue measure. Choose two distinct values $z_1, z_2$ in $\tilde{S}$ that are not equal to any $a_j, j \geq 1$. This is possible because any set with a positive Lebesgue measure has an uncountable number of outcomes, and the list $a_j, j \geq 1$ is countable and therefore does not exhaust the values in $\tilde{S}$ in case there is overlap. Then $\cup_{j\geq 1}\{\varepsilon = a_j - z_1\}$ and $\cup_{j\geq 1}\{\varepsilon = a_j - z_2\}$ are disjoint events, and their probability equals their sum, which is 2, which is impossible, and, therefore, we reach a contradiction which proves that the assumed statement of independence is impossible. □

## Appendix H. How $H$ is influenced by Transformations of the Units of Measurements of $f$

By the well-known scaling problem in confirmatory factor analysis, the unit of measurement of $f$ is not identified from the measurement model in eq. (2), and an arbitrary scale is fixed in applications. Let us therefore consider the effect of going from one scale to another.

We here show that conditional expectations are well-behaved under scale changes. This is surely established in the literature earlier, and the lack of importance of scale transformations is also mentioned in Kelava et al. (2017), but we have failed to find a reference for this, nor the exact formulas for how the changes influence $H$, and we therefore include derivations on this issue here.

Since conditional expectations are defined coordinate wise, we may without loss of generality assume that $\eta$ is univariate.

A scale transformation of one coordinate $f_i$ of $f$ is of the form $af_i + b$ where $a > 0$. How does

$$H(x) = \mathbb{E}[\eta|\xi = x]$$

change under such transformations? The coordinate $f_i$ is either contained in $\eta$ or $\xi$. Scale changes in $\eta$ are dealt with from the linearity of conditional expectation, so that $\mathbb{E}[a\eta + b|\xi] = a\mathbb{E}[\eta|\xi] + b$. Let us therefore consider a scale transformation in a $\xi$.

First, let us consider a univariate and continuous $\xi$. We have

$$\breve{H}(z) = \mathbb{E}[\eta|a\xi + b = z] = \int_{\mathbb{R}} y f_{\eta|a\xi+b}(y|z)\,dy = \int_{\mathbb{R}} y \frac{f_{\eta,a\xi+b}(y,z)}{f_{a\xi+b}(z)}\,dy.$$

We have

$$f_{a\xi+b}(z) = \frac{\partial}{\partial z} P(a\xi + b \leq z) = \frac{\partial}{\partial z} P(\xi \leq (z-b)/a) = a^{-1} f_\xi((z-b)/a)$$

and similarly

$$\begin{aligned} f_{\eta,a\xi+b}(y,z) &= \frac{\partial^2}{\partial y \partial z} P(\eta \leq y, a\xi + b \leq z) \\ &= \frac{\partial^2}{\partial y \partial z} P(\eta \leq y, \xi \leq (z-b)/a) \\ &= a^{-1} f_{\eta,\xi}(y,(z-b)/a). \end{aligned}$$

Therefore

$$\begin{aligned} \mathbb{E}[\eta | a\xi + b = z] &= \int_{\mathbb{R}} y \frac{f_{\eta,\xi}(y,(z-b)/a)}{f_{\xi}((z-b)/a)}\, dy \\ &= \mathbb{E}[\eta | \xi = (z-b)/a)], \end{aligned}$$

since the $a^{-1}$ cancels. Therefore, $\breve{H}(z) = H((z-b)/a)$.

Similar calculations show that scale transformations of $\xi$ in general makes the function $H$ stay the same, except a scale and shift transformation in each of its inputs.

## Appendix I. The Problem of Empirically Approximating the Distribution of $r_\xi$

The theoretical basis for choosing between our suggested approximations for $H$ depend on the distribution of $r_\xi$. One way to approximate the distribution of $r_\xi = \Delta_x \varepsilon_x$ based on data would be to calculate a type of a residual, say $\hat{\varepsilon}_x$ and then inspect the empirical distribution of $\hat{r}_\xi = \hat{\Delta}\hat{\varepsilon}_x$. Unfortunately, this appears to be difficult.

Factor residuals have been studied in Bollen and Arminger (1991), who suggest defining residuals in the way $\hat{\varepsilon}_x = (x-\hat{\mu}_x) - \hat{\Lambda}_x \hat{\xi}$ where $\hat{\xi}$ is an affine factor score, such as the Bartlett factor score. If we use the Bartlett factor score and set $\hat{\xi} = \hat{\Delta}_x(x-\hat{\mu}_x)$, then $\hat{r}_\xi = \hat{\Delta}_x\hat{\varepsilon}_x = \hat{\Delta}_x(x-\hat{\mu}_x) - \hat{\Delta}_x\hat{\Lambda}_x\hat{\Delta}_x(x-\hat{\mu}_x) = \hat{\Delta}_x(x-\hat{\mu}_x) - \hat{\Delta}_x(x-\hat{\mu}_x) = 0$ using that $\hat{\Delta}_x$ is a left inverse of $\hat{\Lambda}_x$. Therefore, the resulting approximation does not work.

In general, for an affine factor score of the form $\hat{\xi} = \hat{A}_x(x-\hat{\mu}_x)$ we get $\hat{r}_\xi = \hat{\Delta}_x(I - \hat{\Lambda}A_x)(x-\hat{\mu}_x)$. Numerical experiments with using the Thurstone matrix $A_x = T_x$ (see Lemma 1 (3)) indicates that the shape of the distribution of $r_\xi$ is lost in this transformation likely due to a central limit effect induced by the summation involved in the matrix multiplication of $\hat{\Delta}_x(I-\hat{\Lambda}A_x)$: The empirical distribution of $\hat{r}_\xi$ is much too normal compared to the distribution of $r_\xi$, and, therefore, cannot be used for diagnostics. Numerical experiments show that this also happens when using the non-parametric factor scores of Kelava et al. (2017). Hence, the empirical approximation of the distribution of $r_\xi$ is an open problem.

## Appendix J. Non-additive noise

Since the methodology considered in this paper is centered around conditional expectation, which is related to averaging and therefore addition, it is most suitable when the relation between $\eta$ and $\xi$ is that of a trend with additive noise. We here provide a very simple illustration of modeling trends with non-additive noise from a conditional expectation framework. While this is a practically important topic, the same issue is met in standard regression modeling with observed variables, and this topic is discussed in text-books on non-linear regression modeling. We consider a full discussion of this issue outside the scope of the present paper.

Consider a non-linear SEM with a structural model where the error term enters in a multiplicative (and therefore non-additive) way through

$$\eta_1 = \exp(\beta_0 + \beta_1\xi_1 + u_1) = e^{\beta_0+\beta_1\xi_1} \cdot e^{u_1}, \tag{25}$$

where $\xi_1, u_1$ are zero mean and independent of each other.

In the non-parametric framework,

$$\eta_1 = H(\xi_1) + \zeta_1, \qquad H(x) = \mathbb{E}[\eta_1|\xi_1 = x]$$

which is additive, and the foundational property $\mathbb{E}[\zeta|\xi] = 0$ is gained by the tautological definition of $\zeta_1 := \eta_1 - H(\xi) = \eta_1 - \mathbb{E}[\eta_1|\xi]$ and basic properties of the conditional expectation.

In the example, we use the independence between $u_1, \xi_1$ to calculate

$$\mathbb{E}[\eta_1|\xi_1] = e^{\beta_0+\beta_1\xi_1}\mathbb{E}[e^{u_1}|\xi] = e^{\beta_0+\beta_1\xi_1}\mathbb{E}[e^{u_1}].$$

Therefore, $H(x) = e^{\beta_0+\beta_1 x}\mathbb{E}[e^{u_1}]$ is still an exponential trend, though with a different level than the description in eq. (25). If e.g. $u_1 \sim N(0,1)$, we have $\mathbb{E}e^{u_1} = e^{1/2}$. Then $H(x) = e^{\beta_0+\beta_1 x}e^{1/2} = e^{0.5+\beta_0+\beta_1 x}$.

Of course, $\zeta_1$ will not be $u_1$. The error term of eq. (25) $u_1$ is independent to $\xi_1$. And the independence between $\xi$ and $\zeta_1$ is not expected, and not assumed in the paper. This might be problematic for parametric estimation methods which assumes such an independence.

We here have

$$\zeta_1 = \eta_1 - \mathbb{E}[\eta_1|\xi_1] = e^{\beta_0+\beta_1\xi_1}\left(e^{u_1} - \mathbb{E}[e^{u_1}]\right)$$

While known from general theory, we confirm that

$$\mathbb{E}[\zeta_1|\xi] = e^{\beta_0+\beta_1\xi_1}\mathbb{E}[e^{u_1} - \mathbb{E}[e^{u_1}]|\xi_1] = \mathbb{E}[\zeta_1|\xi_1] = e^{\beta_0+\beta_1\xi_1}\mathbb{E}[e^{u_1} - \mathbb{E}[e^{u_1}]] = 0$$

where the next to last equality follows from the independence between $e^{u_1} - \mathbb{E}[e^{u_1}]$ and $\xi_1$, as implied by the independence between $\xi_1$ and $u_1$.

From general results we also get that $\zeta_1$ is uncorrelated with $\xi$ and has zero mean. But $\zeta_1$ is not independent to $\xi$, and in fact $\zeta_1$ may be highly dependent to $\xi$, as is the case in the present example. Since $\mathbb{E}[\zeta_1|\xi] = 0$, we have that

$$\begin{aligned}\operatorname{Var}[\zeta_1|\xi] &= \mathbb{E}[\zeta_1^2|\xi] = e^{2\beta_0+2\beta_1\xi_1}\mathbb{E}\left[(e^{u_1} - \mathbb{E}[e^{u_1}])^2|\xi\right] \\ &= e^{2\beta_0+2\beta_1\xi_1}\mathbb{E}\left[(e^{u_1} - \mathbb{E}[e^{u_1}])^2\right] = e^{2\beta_0+2\beta_1\xi_1}\operatorname{Var} e^{u_1}.\end{aligned}$$

As far as we can see, this is problematic for the presently available NLSEM estimators. The practitioner could therefore use factor score plots and trend estimates to detect signs of such dependence, such as conditional heteroskedasticity as seen in the above example, if a parametric model is to be fitted to a model using traditional methods. In the simple case of eq. (25), taking a log transform of $\eta_1$ would be a possibility, though we do not study the statistical implications of this. In econometrics, a large literature presents solutions to this problem (see e.g. Hayashi, 2011). It seems plausible that using these solutions using factor scores, can aid the problem, possibly with some modification. We consider a full analysis of this outside the scope of the present paper.

In the present example, a preferred method would be to identify that a non-additive noise model would be more appropriate. The estimation of $H$ as a trend estimate may be inappropriate to summarize the trend in the factor sore in such cases, but the factor scores themselves might still be of use in a more traditional manner to motivate non-linear models with additive noise. Also this is considered outside the scope of the present paper.

## Appendix K. A Review of Conditional Expectations, and Their Rules

In this section, we provide a short review of conditional expectation and their most important properties, properties used especially in Appendix B, F, G, and H.

Suppose given a probability space $(\Omega, \mathcal{F}, P)$. We consider mappings from $\Omega$ to $\mathbb{R}^d$, and equip $\mathbb{R}^d$ with the Borel $\sigma$-field $\mathcal{B}$. Recall that mappings $Z : \Omega \mapsto \mathbb{R}^d$ are called random vectors (or random variables if $d = 1$) if $Z^{-1}(U) := \{\omega \in \Omega : Z(\omega) \in U\} \in \mathcal{F}$ for any $U \in \mathcal{B}$. Also, a random variable $Z$ is said to be measurable with respect to a $\sigma$-field $\mathcal{H} \subseteq \mathcal{F}$ if $Z^{-1}(U) \in \mathcal{H}$ for any $U \in \mathcal{B}$. Here, measurable can be understood in terms of having information about the events in $\omega \in \Omega$ that result in specific outcomes of $Z(\omega)$. Therefore, if these events are known, the values of $Z$ are known, which is why all statements are made about subsets of $\Omega$.

Modern development of conditional expectations are based on conditioning with respect to a $\sigma$-field $\mathcal{H}$. Let $X$ be a random variable. Suppose $X$ is integrable, which means that $\mathbb{E}|X| < \infty$. The conditional expectation $\mathbb{E}[X|\mathcal{H}]$ of $X$ given $\mathcal{H}$ is a random variable that fulfills the following two properties (Billingsley, 1995, Section 34).

(1) $\mathbb{E}[X|\mathcal{H}]$ is $\mathcal{H}$-measurable and integrable.
(2) For all $G \in \mathcal{H}$, we have $\int_G \mathbb{E}[X|\mathcal{H}] \, \mathrm{d}P = \int_G X \, \mathrm{d}P$.

That such a variables always exists is proved in Billingsley (1995, Section 34). While the two requirements placed on $\mathbb{E}[X|\mathcal{H}]$ do not uniquely construct it, all random variables that fulfill these properties are with probability one equal (Billingsley, 1995). We will follow standard convention and talk about $\mathbb{E}[X|\mathcal{H}]$ in the singular, despite this lack of uniqueness.

The $\sigma$-field generated by $Z$ is $\sigma(Z)$, the smallest $\sigma$-field for which $Z$ is measurable. It is given concretely by $\sigma(Z) = \{Z^{-1}(B) : B \in \mathcal{B}\} = \{\{\omega \in \Omega : Z(\omega) \in B\} : B \in \mathcal{B}\}$ (Billingsley, 1995, Section 33, p. 433).

A Borel function $\varphi$ is a function such that if $B \in \mathcal{B}$, we have that $\varphi^{-1}(B) = \{z : \varphi(z) \in B\} \in \mathcal{B}$. Notice that if $Y = \varphi(Z)$ is a Borel function of $Z$, then for any $U \in \sigma(Z)$ we have that

$$Y^{-1}(U) = \{\omega \in \Omega : Y(\omega) \in U\} = \{\omega \in \Omega : \varphi(Z(\omega)) \in U\} = \{\omega \in \Omega : Z(\omega) \in \varphi^{-1}(U)\}$$

Since $\varphi$ is a Borel function, $\varphi^{-1}(U) \in \mathcal{B}$. Since $\sigma(Z)$ consists of all sets of the form $\{\omega \in \Omega : Z(\omega) \in B\}$ for $B \in \mathcal{B}$, we get that $Y^{-1}(U) \in \sigma(Z)$, and therefore $Y$ is $\sigma(Z)$ measurable.

Also the converse holds:

**Theorem 1** (Remark 5, p. 175 in Shiryaev (2016))**.** *Let $Z$ be a random vector. If a random variable $X$ is $\sigma(Z)$-measurable, there exists a Borel function $\varphi$ such that $X = \varphi(Z)$.*

By the definition of $\mathbb{E}[X|\sigma(Z)]$, it is $\sigma(Z)$ measurable. By Theorem 1, that means that $\mathbb{E}[X|\sigma(Z)]$ is a function of $Z$. We usually write $\mathbb{E}[X|Z]$ instead of $\mathbb{E}[X|\sigma(Z)]$. That is, there is a function $\varphi$ so that

$$\varphi(Z) = \mathbb{E}[X|Z].$$

Now for $Z = (Y_1', Y_2')'$ where $Y_1, Y_2$ are random vectors, we sometimes write $\mathbb{E}[X|Y_1, Y_2]$, which means $\mathbb{E}[X|Z]$. As in the case of expectations of random vectors, if $X$ is a random vector $X = (X_1, \dots, X_n)$ then we define

$$\mathbb{E}[X|Z] = (\mathbb{E}[X_1|Z], \dots, \mathbb{E}[X_n|Z])'.$$

Since $\mathbb{E}[X|Z]$ is a function of $Z$, there is a function $\varphi$ such that $\mathbb{E}[X|Z] = \varphi(Z)$. This function is sometimes denoted by $\varphi(z) = \mathbb{E}[X|Z = z]$, although it is not the case that the conditional expectation

is the expectation with respect to the probability measure conditioned on the event $Z = z$ (though in the discrete case the function does correspond to this when $P(Z = z) > 0$).

While the above description is very abstract, the function $\varphi$ fulfills a property which connects conditional expectations with non-parametric regression: For random a random vector $X$ and a random variable $Y$, let $\varphi(x) = \mathbb{E}[Y|X = x]$. Then $\varphi$ minimizes the squared distance to $Y$ for given $x$:

$$\mathbb{E}\left[(Y - \varphi(x))^2\right],$$

i.e., $\varphi(x)$ is the least squares estimate for $Y$ at $X = x$ (see e.g. Hayashi, 2011, Proposition 2.7). Using a linear function for $\varphi$ results in the definition of the linear regression least squares estimator, while modeling $\varphi$ non-parametrically highlights the connection of the conditional expectation to non-parametric regression analysis: The non-parametric regression estimate approximates the conditional expectation.

We now review the most important properties of conditional expectations that are used in this paper.

**Theorem 2** (Theorem 34.2 in Billingsley (1995)). *Suppose $X, Y$ are integrable (i.e., $\mathbb{E}|X| < \infty, \mathbb{E}|Y| < \infty$).*

*(1) If $X = a$ with probability 1, then $\mathbb{E}[X|Z] = a$.*
*(2) For constants $a, b$, we have $\mathbb{E}[aX + bY|Z] = a\mathbb{E}[X|Z] + b\mathbb{E}[Y|Z]$.*
*(3) If $X \leq Y$ with probability 1, then $\mathbb{E}[X|Z] \leq \mathbb{E}[Y|Z]$.*
*(4) $|\mathbb{E}[X|Z]| \leq \mathbb{E}[|X||Z]$.*

**Theorem 3** (Theorem 34.3 in Billingsley (1995)). *If $X$ is measurable with regard to a $\sigma$-field $\mathcal{H}$, and if $Y$ and $XY$ are integrable, then*

$$\mathbb{E}[XY|\mathcal{H}] = X\mathbb{E}[Y|\mathcal{H}], \text{ with probability 1.}$$

From this combined with Theorem 1, it follows that $\mathbb{E}[Z|Z] = Z$ and $\mathbb{E}[\varphi(Z)|Z] = \varphi(Z)$, for an integrable function $\varphi$.

**Theorem 4** (Law of Iterated Expectations, Theorem 34.4 in Billingsley (1995)). *If $X$ is integrable and the $\sigma$-field $\mathcal{G}_1$ and $\mathcal{G}_2$ satisfy $\mathcal{G}_1 \subseteq \mathcal{G}_2$ then*

$$\mathbb{E}\left[\mathbb{E}[X|\mathcal{G}_2]|\mathcal{G}_1\right] = \mathbb{E}\left[X|\mathcal{G}_1\right].$$

This can be used e.g. when $\mathcal{G}_1 = \sigma(Z_1) \subseteq \sigma(Z_1, Z_2) = \mathcal{G}_2$ (see the upcoming Section K.1), in which case we have $\mathbb{E}[\mathbb{E}[X|Z_1, Z_2]|Z_1] = \mathbb{E}[X|Z_1]$.

**Theorem 5** (Tower Property, see the discussion following Theorem 34.4 in Billingsley (1995)). *If $X$ is integrable then*

$$\mathbb{E}\left[\mathbb{E}[X|Z]\right] = \mathbb{E}\left[X\right].$$

From e.g. Problem 34.2 in (Billingsley, 1995, p. 455), we have that when $X, Y$ are continuous random variables with a joint density $f$ and $Y$ is integrable, then

$$\mathbb{E}[Y|X = x] = \frac{\int_{-\infty}^{\infty} y f(x, y) \, \mathrm{d}y}{\int_{-\infty}^{\infty} f(x, y) \, \mathrm{d}y}. \tag{26}$$

From e.g. Problem 34.3 in (Billingsley, 1995, p. 455) we have that if $X, Y$ are independent

$$\mathbb{E}[Y|X] = \mathbb{E}[Y]. \tag{27}$$

**Lemma 7.** *For a random variables $X$ and a random vector $Y$ we have that*

$$\mathbb{E}[X|X,Y] = X.$$

*Proof of Lemma 7.* Since $X = \varphi(X,Y)$ where $\varphi(x,y) = x$ is a Borel function, we have that $X$ is $\sigma(X,Y)$ measurable (see the comment just before Theorem 1). Therefore, from Theorem 3, we have $\mathbb{E}[X|X,Y] = X\mathbb{E}[1|X,Y] = X$. □

Lemma 7 implies e.g. that $\mathbb{E}[X|X,U,V] = X$ for $Y = (U,V)$.

**Lemma 8.** *(1) For two bivariately normal variables $A, B$, we have that*

$$\mathbb{E}[A|B] = \mu_A + \mathrm{Cov}\,(A,B)\,\mathrm{Var}\,(B)^{-1}(B - \mu_B)$$

*and*

$$\mathrm{Var}\,[A|B] = \mathrm{Var}\,(A) - \mathrm{Cov}\,(A,B)^2\,\mathrm{Var}\,(B)^{-1} \tag{28}$$

*(2) For a jointly normal random vector $(Y,X)$ with mean vector and covariance matrix*

$$\mu = \begin{pmatrix} \mu_X \\ \mu_Y \end{pmatrix}, \quad \Sigma = \begin{pmatrix} K_{X,X} & K_{X,Y} \\ K_{Y,X} & K_{Y,Y} \end{pmatrix}$$

*we have that*

$$Y|X \sim N(\mu_{Y|X}, K_{Y|X})$$

*where*

$$\mu_{Y|X} = \mu_Y + K_{YX}K_{XX}^{-1}(X - \mu_X)$$

*and*

$$K_{Y|X} = K_{Y,Y} - K_{Y,X}K_{X,X}^{-1}K_{X,Y}.$$

*Proof.* See (Mardia et al., 1979, Theorem 3.2.4). □

We conclude this section by showing the property of $\xi$ and $\zeta$ mentioned in the introduction.

**Lemma 9.** *If $\mathbb{E}[\zeta|\xi] = 0$ then $\mathbb{E}\zeta = 0$ and $\mathrm{Cov}\,(\varphi(\xi),\zeta) = 0$ for any $\varphi$ such that $\varphi(\xi)$ is integrable.*

*Proof.* We have $\mathbb{E}\zeta = \mathbb{E}\mathbb{E}[\zeta|\xi] = \mathbb{E}0 = 0$.

Therefore, $\mathrm{Cov}\,(\varphi(\xi),\zeta) = \mathbb{E}[\varphi(\xi)\zeta] - [\mathbb{E}\varphi(\xi)][\mathbb{E}\zeta] = \mathbb{E}\big[\mathbb{E}[\varphi(\xi)\zeta|\xi]\big] = \mathbb{E}\big[\varphi(\xi)\mathbb{E}[\zeta|\xi]\big] = 0.$ □

K.1. **Some stability results of $\sigma$-fields generated by random vectors.** We here gather two results we use in the paper, for which we did not find a reference. Especially the first property is well-known.

Let $\mathcal{B}(\mathbb{R}^d)$ be the Borel $\sigma$-field for the $d$-dimensional Euclidean space. Recall that Chapter 2.2.3 (p. 176) in Shiryaev (2016) that for two $\sigma$-fields $\mathcal{F}_1, \mathcal{F}_2$, the product $\sigma$-field $\mathcal{F} = \mathcal{F}_1 \otimes \mathcal{F}_2$ is the smallest $\sigma$-field containing all sets of the form $B_1 \times B_2$ where $B_1 \in \mathcal{F}_1, B_2 \in \mathcal{F}_2$.

It is the case that

$$\mathcal{B}(\mathbb{R}^{d_1}) \otimes \mathcal{B}(\mathbb{R}^{d_2}) = \mathcal{B}(\mathbb{R}^{d_1+d_2}),$$

as shown in e.g. Chapter 2.2.3 (p. 176) in Shiryaev (2016).

**Lemma 10.** *For two $d_1, d_2$ dimensional random vectors $Z_1, Z_2$, we have $\sigma(Z_1) \subseteq \sigma(Z_1, Z_2)$.*

*Proof.* As mentioned just before Theorem 1, we have

$$\begin{aligned}\sigma(Z_1) &= \{Z_1^{-1}(B_1) : B_1 \in \mathcal{B}(\mathbb{R}^{d_1})\} \\ &= \{\{\omega \in \Omega : Z_1(\omega) \in B_1\} : B_1 \in \mathcal{B}(\mathbb{R}^{d_1})\}.\end{aligned}$$

We also have

$$\begin{aligned}\sigma(Z_1, Z_2) &= \{(Z_1, Z_2)^{-1}(B) : B \in \mathcal{B}(\mathbb{R}^{d_1+d_2})\} \\ &= \{\{\omega \in \Omega : (Z_1(\omega), Z_2(\omega)) \in B\} : B \in \mathcal{B}(\mathbb{R}^{d_1+d_2})\}\end{aligned}$$

Since $\mathcal{B}(\mathbb{R}^{d_1+d_2}) = \mathcal{B}(\mathbb{R}^{d_1}) \otimes \mathcal{B}(\mathbb{R}^{d_2})$, we have that for all $B_1 \in \mathcal{B}(\mathbb{R}^{d_1}), B_2 \in \mathcal{B}(\mathbb{R}^{d_2})$ it is the case that $B_1 \times B_2 \in \mathcal{B}(\mathbb{R}^{d_1+d_2})$.

Using this and that $\mathcal{B}(\mathbb{R}^{d_2})$ is a $\sigma$-field so that $\mathbb{R}^{d_2} \in \mathcal{B}(\mathbb{R}^{d_2})$, shows that for any $B_1 \in \mathcal{B}(\mathbb{R}^{d_1})$ we have that $B_1 \times \mathbb{R}^{d_2} \in \mathcal{B}(\mathbb{R}^{d_1+d_2})$.

Therefore,

$$\begin{aligned}\sigma(Z_1) &= \{\{\omega \in \Omega : Z_1(\omega) \in B_1\} : B_1 \in \mathcal{B}(\mathbb{R}^{d_1})\} \\ &= \{\{\omega \in \Omega : Z_1(\omega) \in B_1, Z_2(\omega) \in \mathbb{R}^{d_2}\} : B_1 \in \mathcal{B}(\mathbb{R}^{d_1})\} \\ &= \{\{\omega \in \Omega : Z_1(\omega) \in B_1, Z_2(\omega) \in \mathbb{R}^{d_2}\} : B_1 \times \mathbb{R}^{d_2} \in \mathcal{B}(\mathbb{R}^{d_1+d_2})\} \\ &\subseteq \{\{\omega \in \Omega : (Z_1(\omega), Z_2(\omega)) \in B\} : B \in \mathcal{B}(\mathbb{R}^{d_1+d_2})\} \\ &= \sigma(Z_1, Z_2).\end{aligned}$$

□

For the next lemma, we recall Jacod and Protter (2004, Theorem 8.1), stated below. Before we state it we recall the following more general general concept from measure theory.

Let $(E, \mathcal{E})$ and $(F, \mathcal{F})$ be two measurable spaces. A function $X : E \mapsto F$ is measurable relative to $\mathcal{E}$ and $\mathcal{F}$ if $X^{-1}(\Xi) \in \mathcal{E}$ for all $\Xi \in \mathcal{F}$.

**Theorem 6** (Theorem 8.1 in Jacod and Protter (2004))**.** *Let $\mathcal{C}$ be a class of subsets of $\Omega$ such that $\sigma(\mathcal{C}) = \mathcal{F}$. Then $X : E \mapsto F$ is measurable (relative to $\mathcal{E}$ and $\mathcal{F}$) if and only if $X^{-1}(C) \in \mathcal{E}$ for all $C \in \mathcal{C}$.*

**Lemma 11.** *Let $X$ be a $d_1$ dimensional random variable and $\varphi : \mathbb{R}^{d_1} \mapsto \mathbb{R}^{d_2}$ a Borel function. Then $\sigma(X) = \sigma(X, \varphi(X))$.*

*Proof of Lemma 11.* Since $\varphi$ is a Borel function, $\varphi(X)$ is a random variable. We first show $\sigma(X) \subseteq \sigma(X, \varphi(X))$ and then that $\sigma(X, \varphi(X)) \subseteq \sigma(X)$, which implies that $\sigma(X) = \sigma(X, \varphi(X))$.

First, Lemma 10 implies that $\sigma(X) \subseteq \sigma(X, \varphi(X))$.

Second, we show that $\sigma(X, \varphi(X)) \subseteq \sigma(X)$. We do this by showing that $(X, \varphi(X))$ is $\sigma(X)$ measurable. Since $\sigma(X, \varphi(X))$ is the smallest $\sigma$-field such that $(X, \varphi(X))$ is measurable with respect to it, and $\sigma(X)$ is a $\sigma$-field.

To do this, we use Theorem 6. We have that $(X, \varphi(X)) : \Omega \to \mathbb{R}^{d_1+d_2}$, where $\mathbb{R}^{d_1+d_2}$ is equipped with the Borel $\sigma$-field $\mathcal{B}(\mathbb{R}^{d_1+d_2}) = \mathcal{B}(\mathbb{R}^{d_1}) \otimes \mathcal{B}(\mathbb{R}^{d_2})$ which as mentioned at the start of this sub-section is generated by the product sets of the form $B_1 \times B_2$ where $B_1 \in \mathcal{B}(\mathbb{R}^{d_1}), B_2 \in \mathcal{B}(\mathbb{R}^{d_2})$. Let $\mathcal{C} =$

$\{B_1 \times B_2 : B_1 \in \mathcal{B}(\mathbb{R}^{d_1}), B_2 \in \mathcal{B}(\mathbb{R}^{d_2})\}$. By Theorem 6, we need to show that $(X, \varphi(X))^{-1}(C) \in \sigma(X)$ for all $C \in \mathcal{C}$. Let $C \in \mathcal{C}$ so that $C = B_1 \times B_2$. We have

$$\begin{aligned}(X, \varphi(X))^{-1}(C) &= \{\omega \in \Omega : (X(\omega), \varphi(X(\omega))) \in B_1 \times B_2\} \\ &= \{\omega \in \Omega : X(\omega) \in B_1, \varphi(X(\omega)) \in B_2\} \\ &= \{\omega \in \Omega : X(\omega) \in B_1\} \cap \{\omega \in \Omega : \varphi(X(\omega)) \in B_2\} \\ &= \{\omega \in \Omega : X(\omega) \in B_1\} \cap \{\omega \in \Omega : X(\omega) \in \varphi^{-1}(B_2)\}\end{aligned}$$

For the last step, recall that $\varphi^{-1}(B_2) = \{z : \varphi(z) \in B_2\}$. Therefore, $\varphi(X(\omega)) \in B_2$ is equivalent to $X(\omega) \in \varphi^{-1}(B_2)$.

Since $\varphi$ is a Borel function, $\varphi^{-1}(B_2) \in \mathcal{B}(\mathbb{R}^{d_1})$. Therefore, the sets that are intersected are both of the form $\{\omega \in \Omega : X(\omega) \in B\} = X^{-1}(B)$ for a set $B \in \mathcal{B}(\mathbb{R}^{d_1})$, all of which are in

$$\sigma(X) = \{X^{-1}(B) : B \in \mathcal{B}(\mathbb{R}^{d_1})\}.$$

Since $\sigma$-fields are stable under finite intersections, $(X, \varphi(X))^{-1}(C) \in \sigma(X)$. □

## Appendix L. Miscellanea

Let $M$ be a square and symmetric matrix. It is a positive semidefinite matrix if its quadratic form is non-negative. If $M$ is positive definite, it is also positive semidefinite.

**Lemma 12.** *For a $m \times m$ matrix $M$ with elements $(m_{i,j})_{ij}$ that is symmetric and positive semidefinite, we have that $\max_{1 \le i,j \le m} |m_{i,j}| \le \lambda_{\max}(M)$.*

*Proof.* Since $M$ is a square symmetric positive semidefinite matrix, Theorem 4.2.8 in Golub and Van Loan (2013) shows that

$$\max_{1 \le i,j \le m} |m_{i,j}| = \max_{1 \le i \le m} m_{i,i}.$$

Recall that $\lambda_{\max}(M) = \max_{\|x\|_2 = 1} x' M x$ where $\|x\|_2 = \sqrt{\sum_{i=1}^m x_i^2}$. Choose $x = e_j$ be the $j$'th unit vector $e_j = (0, 0, \ldots, 0, 1, 0, \ldots, 0)'$, which is such that $\|x\|_2 = \sqrt{\sum_{i=1}^m x_i^2} = 1$ and $x' M x = m_{i,i}$. Therefore, for each $1 \le i \le m$ we have $m_{i,i} \le \max_{\|x\|_2 = 1} x' M x = \lambda_{\max}(M)$, and therefore $\max_{1 \le i,j \le m} |m_{i,j}| = \max_{1 \le i \le m} m_{i,i} \le \lambda_{\max}(M)$. □

The following lemma is well known in the literature, and is used e.g. in Rosseel and Loh (2022). While the result is given in Johnson and Wichern (2002, Exercise 9.6, p. 531) in the case when $\Phi$ is the identity matrix, and can therefore be considered standard, we have not found a reference with explicit statement and proof of the full result, and we for completeness include a proof for it using our Assumption 1.

**Lemma 13.** *Suppose given Assumption 1. Then the Thurstone matrix $T := \Phi \Lambda' \Sigma^{-1}$ used to derive the regression factor score is equivalent to $T_2 := \left(\Phi^{-1} + \Lambda' \Psi^{-1} \Lambda\right)^{-1} \Lambda' \Psi^{-1}$.*

*Proof of Lemma 13.* We begin with $T$ and notice that there are several alternative notations for the population covariance matrix $\Sigma_z := \Lambda \Phi \Lambda' + \Psi$. Let $L := \Lambda(\Phi^{\frac{1}{2}})'$, where $\Phi^{\frac{1}{2}}$ is part of the Cholesky decomposition (see, e.g., Horn & Johnson, 2013, p. 441, Corollary 7.2.9) of $\Phi = (\Phi^{\frac{1}{2}})' \Phi^{\frac{1}{2}}$. Here, $\Phi^{\frac{1}{2}}$ is an upper triangular matrix. Further, we note that for the Cholesky decomposition the following identity holds: $\Phi^{-1} = \left((\Phi^{\frac{1}{2}})' \Phi^{\frac{1}{2}}\right)^{-1} = \Phi^{-\frac{1}{2}} (\Phi^{-\frac{1}{2}})'$, where $\Phi^{-\frac{1}{2}}$ is the inverse of $\Phi^{\frac{1}{2}}$. For the proof,

we make use of three matrix properties which are sequentially proven. These are based on exercises in (Johnson & Wichern, 2002, see Exercise 9.6, p. 531):

(a) $(I_{d_f} + L'\Psi^{-1}L)^{-1}L'\Psi^{-1}L = I_{d_f} - (I_{d_f} + L'\Psi^{-1}L)^{-1}$
(b) $(LL' + \Psi)^{-1} = \Psi^{-1} - \Psi^{-1}L\left(I_{d_f} + L'\Psi^{-1}L\right)^{-1}L'\Psi^{-1}$
(c) $L'(LL' + \Psi)^{-1} = \left(I_{d_f} + L'\Psi^{-1}L\right)^{-1}L'\Psi^{-1}$

*Proof of (a).* We proof (a) by premultiplying both sides of (a) by $I_{d_f} + L'\Psi^{-1}L$:

$$\begin{aligned}
\left[I_{d_f} + L'\Psi^{-1}L\right](I_{d_f} + L'\Psi^{-1}L)^{-1}L'\Psi^{-1}L &= \left[I_{d_f} + L'\Psi^{-1}L\right]\left(I_{d_f} - (I_{d_f} + L'\Psi^{-1}L)^{-1}\right) \\
\iff L'\Psi^{-1}L &= \left[I_{d_f} + L'\Psi^{-1}L\right] - I_{d_f} \\
\iff L'\Psi^{-1}L &= L'\Psi^{-1}L.
\end{aligned}$$

□

*Proof of (b).* We provide proof for (b) by postmultiplying both sides of (b) by $LL' + \Psi$:

$$\begin{aligned}
(LL' + \Psi)^{-1}\left[LL' + \Psi\right] = \Big(\Psi^{-1} - \Psi^{-1}L\left(I_{d_f} + L'\Psi^{-1}L\right)^{-1}L'\Psi^{-1}\Big)\left[LL' + \Psi\right] \\
\iff I_{d_z} = \Psi^{-1}\left[LL' + \Psi\right] - \Psi^{-1}L\left(I_{d_f} + L'\Psi^{-1}L\right)^{-1}L'\Psi^{-1}\left[LL' + \Psi\right] \\
\iff I_{d_z} = \Psi^{-1}LL' + I_{d_z} - \Psi^{-1}L\left(I_{d_f} + L'\Psi^{-1}L\right)^{-1}L'\Psi^{-1}LL' - \\
\Psi^{-1}L\left(I_{d_f} + L'\Psi^{-1}L\right)^{-1}L'\Psi^{-1}\Psi \\
\overset{(a)}{\iff} I_{d_z} = \Psi^{-1}LL' + I_{d_z} - \Psi^{-1}L\left[I_{d_f} - (I_{d_f} + L'\Psi^{-1}L)^{-1}\right]L' - \\
\Psi^{-1}L\left(I_{d_f} + L'\Psi^{-1}L\right)^{-1}L' \\
\iff I_{d_z} = \Psi^{-1}LL' + I_{d_z} - \Psi^{-1}LL' + \Psi^{-1}L(I_{d_f} + L'\Psi^{-1}L)^{-1}L' - \\
\Psi^{-1}L\left(I_{d_f} + L'\Psi^{-1}L\right)^{-1}L' \\
\iff I_{d_z} = I_{d_z}.
\end{aligned}$$

□

*Proof of (c).* We provide proof for (c) and begin by postmultiplying (b) with $L$:

$$\begin{aligned}
(LL' + \Psi)^{-1}L &= \Big(\Psi^{-1} - \Psi^{-1}L\left(I_{d_f} + L'\Psi^{-1}L\right)^{-1}L'\Psi^{-1}\Big)L \\
\iff (LL' + \Psi)^{-1}L &= \Psi^{-1}L - \Psi^{-1}L\left(I_{d_f} + L'\Psi^{-1}L\right)^{-1}L'\Psi^{-1}L \\
\overset{(a)}{\iff} (LL' + \Psi)^{-1}L &= \Psi^{-1}L - \Psi^{-1}L\left[I_{d_f} - (I_{d_f} + L'\Psi^{-1}L)^{-1}\right] \\
\iff (LL' + \Psi)^{-1}L &= \Psi^{-1}L - \Psi^{-1}L + \Psi^{-1}L(I_{d_f} + L'\Psi^{-1}L)^{-1} \\
\iff (LL' + \Psi)^{-1}L &= \Psi^{-1}L(I_{d_f} + L'\Psi^{-1}L)^{-1}.
\end{aligned}$$

Now, we transpose both sides and use that $\Psi^{-1}$, $(LL' + \Psi)^{-1}$ and $(I_{d_f} + L'\Psi^{-1}L)^{-1}$ are symmetric and we have

$$L'(LL' + \Psi)^{-1} = \left(I_{d_f} + L'\Psi^{-1}L\right)^{-1}L'\Psi^{-1}.$$

□

Now, resubstitute $L := \Lambda(\Phi^{\frac{1}{2}})'$ in $L'\left(LL' + \Psi\right)^{-1} = \left(I_{d_f} + L'\Psi^{-1}L\right)^{-1} L'\Psi^{-1}$ and we have:

$$
\begin{aligned}
& L'\left(LL' + \Psi\right)^{-1} = \left(I_{d_f} + L'\Psi^{-1}L\right)^{-1} L'\Psi^{-1} \\
\iff\ & (\Lambda(\Phi^{\frac{1}{2}})')'\left(\Lambda(\Phi^{\frac{1}{2}})'(\Lambda(\Phi^{\frac{1}{2}})')' + \Psi\right)^{-1} = \left(I_{d_f} + (\Lambda(\Phi^{\frac{1}{2}})')'\Psi^{-1}\Lambda(\Phi^{\frac{1}{2}})'\right)^{-1} (\Lambda(\Phi^{\frac{1}{2}})')'\Psi^{-1} \\
\iff\ & \Phi^{\frac{1}{2}}\Lambda'\left(\Lambda(\Phi^{\frac{1}{2}})'\Phi^{\frac{1}{2}}\Lambda' + \Psi\right)^{-1} = \left(I_{d_f} + \Phi^{\frac{1}{2}}\Lambda'\Psi^{-1}\Lambda(\Phi^{\frac{1}{2}})'\right)^{-1} \Phi^{\frac{1}{2}}\Lambda'\Psi^{-1} \\
\overset{\Phi=(\Phi^{\frac{1}{2}})'\Phi^{\frac{1}{2}}}{\iff}\ & \Phi^{\frac{1}{2}}\Lambda'\left(\Lambda\Phi\Lambda' + \Psi\right)^{-1} = \left(\Phi^{\frac{1}{2}}\left[\Phi^{-\frac{1}{2}} I_{d_f}(\Phi^{-\frac{1}{2}})' + \Lambda'\Psi^{-1}\Lambda\right](\Phi^{\frac{1}{2}})'\right)^{-1} \Phi^{\frac{1}{2}}\Lambda'\Psi^{-1} \\
\overset{\Phi^{-1}=\Phi^{-\frac{1}{2}}(\Phi^{-\frac{1}{2}})'}{\iff}\ & \Phi^{\frac{1}{2}}\Lambda'\left(\Lambda\Phi\Lambda' + \Psi\right)^{-1} = (\Phi^{-\frac{1}{2}})'\left(\Phi^{-1} + \Lambda'\Psi^{-1}\Lambda\right)^{-1} \Phi^{-\frac{1}{2}}\Phi^{\frac{1}{2}}\Lambda'\Psi^{-1} \\
\iff\ & \Phi^{\frac{1}{2}}\Lambda'\left(\Lambda\Phi\Lambda' + \Psi\right)^{-1} = (\Phi^{-\frac{1}{2}})'\left(\Phi^{-1} + \Lambda'\Psi^{-1}\Lambda\right)^{-1} \Lambda'\Psi^{-1}.
\end{aligned}
$$

Premultiplying both sides with $(\Phi^{\frac{1}{2}})'$ results in

$$
\begin{aligned}
& (\Phi^{\frac{1}{2}})'\Phi^{\frac{1}{2}}\Lambda'\left(\Lambda\Phi\Lambda' + \Psi\right)^{-1} = (\Phi^{\frac{1}{2}})'(\Phi^{-\frac{1}{2}})'\left(\Phi^{-1} + \Lambda'\Psi^{-1}\Lambda\right)^{-1} \Lambda'\Psi^{-1} \\
\iff\ & \Phi\Lambda'\left(\Lambda\Phi\Lambda' + \Psi\right)^{-1} = \left(\Phi^{-1} + \Lambda'\Psi^{-1}\Lambda\right)^{-1} \Lambda'\Psi^{-1} \\
\iff\ & \Phi\Lambda'\Sigma_z^{-1} = \left(\Phi^{-1} + \Lambda'\Psi^{-1}\Lambda\right)^{-1} \Lambda'\Psi^{-1} \\
\iff\ & T = T_2.
\end{aligned}
$$

□

## References


Arminger, G., & Muthén, B. O. (1998). A bayesian approach to nonlinear latent variable models using the gibbs sampler and the metropolis-hastings algorithm. *Psychometrika*, *63*, 271–300.

Aust, F., & Barth, M. (2022). papaja: Prepare reproducible APA journal articles with R Markdown [Computer software manual]. Retrieved from `https://github.com/crsh/papaja` (R package version 0.1.1)

Bartlett, M. S. (1937). The statistical conception of mental factors. *British Journal of Psychology. General Section*, *28*(1), 97–104. https://doi.org/10.1111/j.2044-8295.1937.tb00863.x

Bauer, D. J. (2005). A semiparametric approach to modeling nonlinear relations among latent variables. *Structural Equation Modeling: A Multidisciplinary Journal*, *12*(4), 513–535.

Bierens, H. J. (2004). *Introduction to the mathematical and statistical foundations of econometrics.* Cambridge University Press.

Billingsley, P. (1995). *Probability and Measure.* New York: John Wiley & Sons. (3rd. Edition)

Bollen, K. A. (1980). Issues in the comparative measurement of political democracy. *American Sociological Review*, *45*(3), 370–390.

Bollen, K. A. (1995). Structural equation models that are nonlinear in latent variables: A least-squares estimator. *Sociological Methodology*, *25*, 223–251.

Bollen, K. A., & Arminger, G. (1991). Observational residuals in factor analysis and structural equation models. *Sociological Methodology*, 235–262.

Bollen, K. A., & Paxton, P. (1998). Interactions of latent variables in structural equation models. *Structural Equation Modeling: A Multidisciplinary Journal*, *5*(3), 267–293. https://doi.org/10.1080/10705519809540105

Brandt, H., Cambria, J., & Kelava, A. (2018). An adaptive bayesian lasso approach with spike-and-slab priors to identify multiple linear and nonlinear effects in structural equation models. *Structural Equation Modeling: A Multidisciplinary Journal*, *25*(6), 946–960. https://doi.org/10.1080/10705511.2018.1474114

Büchner, R. D., & Klein, A. G. (2020). A quasi-likelihood approach to assess model fit in quadratic and interaction SEM. *Multivariate Behavioral Research*, *55*(6), 855–872. https://doi.org/10.1080/00273171.2019.1689349

Cheung, G. W., & Lau, R. S. (2017). Accuracy of parameter estimates and confidence intervals in moderated mediation models: a comparison of regression and latent moderated structural equations. *Organizational Research Methods*, *20*(4), 746–769. https://doi.org/10.1177/1094428115595869

Cleveland, W. S. (1979). Robust locally weighted regression and smoothing scatterplots. *Journal of the American Statistical Association*, *74*(368), 829–836.

Cleveland, W. S. (1981). LOWESS: A program for smoothing scatterplots by robust locally weighted regression. *The American Statistician*, *35*(1), 54–54.

Cleveland, W. S., Grosse, E., & Shyu, W. M. (1992). Local regression models. In J. M. Chambers & T. J. Hastie (Eds.), *Statistical models in S* (pp. 309–379). Springer.

De Boor, C. (1978). *A practical guide to splines.* Springer-Verlag New York.

Delaigle, A. (2014). Nonparametric kernel methods with errors-in-variables: constructing estimators, computing them, and avoiding common mistakes. *Australian & New Zealand Journal of Statistics*, *56*(2), 105–124. https://doi.org/10.1111/anzs.12066

Delaigle, A., Fan, J., & Carroll, R. J. (2009). A design-adaptive local polynomial estimator for the errors-in-variables problem. *Journal of the American Statistical Association*, *104*(485), 348–359. https://doi.org/10.1198/jasa.2009.0114

Delaigle, A., & Hall, P. (2008). Using simex for smoothing-parameter choice in errors-in-variables problems. *Journal of the American Statistical Association*, *103*(481), 280–287. https://doi.org/10.1198/016214507000001355

Edwards, C. H. (1973). *Advanced calculus of several variables.* Academic Press.

Etezadi-Amoli, J., & McDonald, R. P. (1983). A second generation nonlinear factor analysis. *Psychometrika*, *48*(3), 315–342.

Fan, J., & Gijbels, I. (1996). *Local polynomial modelling and its applications: monographs on statistics and applied probability 66* (Vol. 66). CRC Press.

Feng, X.-N., Wang, G.-C., Wang, Y.-F., & Song, X.-Y. (2015). Structure detection of semiparametric structural equation models with bayesian adaptive group lasso. *Statistics in Medicine*, *34*(9), 1527–1547. https://doi.org/10.1002/sim.6410

Genz, A., & Bretz, F. (2009). *Computation of multivariate normal and t probabilities.* Heidelberg: Springer-Verlag.

Gibson, W. A. (1959). Three multivariate models: Factor analysis, latent structure analysis,

and latent profile analysis. *Psychometrika*, *24*(3), 229–252.

Golub, G. H., & Van Loan, C. F. (2013). *Matrix computations* (4th ed.). JHU press.

Grice, J. W. (2001). Computing and evaluating factor scores. *Psychological Methods*, *6*(4), 430-450. https://doi.org/10.1037/1082-989X.6.4.430

Grønneberg, S., Foldnes, N., & Marcoulides, K. M. (2022). covsim: An R package for simulating non-normal data for structural equation models using copulas. *Journal of Statistical Software*, *102*, 1–45.

Guo, R., Zhu, H., Chow, S.-M., & Ibrahim, J. G. (2012). Bayesian lasso for semiparametric structural equation models. *Biometrics*, *68*(2), 567-577. https://doi.org/10.1111/j.1541-0420.2012.01751.x

Hansen, B. (2022). *Econometrics.* Princeton University Press.

Harville, D. A. (1997). *Matrix algebra from a statistician's perspective.* Springer.

Hayashi, F. (2011). *Econometrics.* Princeton University Press.

Holst, K. K., & Budtz-Jørgensen, E. (2020). A two-stage estimation procedure for non-linear structural equation models. *Biostatistics*, *21*(4), 676–691. https://doi.org/10.1093/biostatistics/kxy082

Horn, R. A., & Johnson, C. R. (2013). *Matrix analysis* (2nd ed.). Cambridge University Press.

Huang, X., & Zhou, H. (2017). An alternative local polynomial estimator for the error-in-variables problem. *Journal of Nonparametric Statistics*, *29*(2), 301–325.

Jacod, J., & Protter, P. (2004). *Probability essentials.* Springer Science & Business Media.

Jin, S., Vegelius, J., & Yang-Wallentin, F. (2020). A marginal maximum likelihood approach for extended quadratic structural equation modeling with ordinal data. *Structural Equation Modeling: A Multidisciplinary Journal*, *27*(6), 864–873. https://doi.org/10.1080/10705511.2020.1712552

Johnson, R. A., & Wichern, D. W. (2002). *Applied multivariate statistical analysis* (6th ed.). Prentice hall Upper Saddle River, NJ.

Kallenberg, O. (2021). *Foundations of modern probability* (3rd ed.). Springer.

Kelava, A., & Brandt, H. (2009). Estimation of nonlinear latent structural equation models using the extended unconstrained approach. *Review of Psychology*, *16*(2), 123–132.

Kelava, A., Kohler, M., Krzyżak, A., & Schaffland, T. F. (2017). Nonparametric estimation of a latent variable model. *Journal of Multivariate Analysis*, *154*, 112–134.

Kelava, A., & Nagengast, B. (2012). A bayesian model for the estimation of latent interaction and quadratic effects when latent variables are non-normally distributed. *Multivariate Behavioral Research*, *47*(5), 717–742. https://doi.org/10.1080/00273171.2012.715560

Kelava, A., Nagengast, B., & Brandt, H. (2014). A nonlinear structural equation mixture modeling approach for nonnormally distributed latent predictor variables. *Structural Equation Modeling: A Multidisciplinary Journal*, *21*(3), 468–481.

Kenny, D. A., & Judd, C. M. (1984). Estimating the nonlinear and interactive effects of latent variables. *Psychological Bulletin*, *96*(1), 201–210. https://doi.org/10.1037/0033-2909.96.1.201

Klein, A. G., & Moosbrugger, H. (2000). Maximum likelihood estimation of latent interaction effects with the LMS method. *Psychometrika*, *65*(4), 457–474. https://doi.org/10.1007/BF02296338

Klein, A. G., & Muthén, B. O. (2007). Quasi-maximum likelihood estimation of structural equation models with multiple interaction and quadratic effects. *Multivariate Behavioral Research*, *42*(4), 647–673. https://doi.org/10.1080/00273170701710205

Kohler, M., Müller, F., & Walk, H. (2015). Estimation of a regression function corresponding to latent variables. *Journal of Statistical Planning and Inference*, *162*, 88–109.

Lee, S.-Y., Song, X.-Y., & Cai, J.-H. (2010). A bayesian approach for nonlinear structural equation models with dichotomous variables using logit and probit links. *Structural Equation Modeling: A Multidisciplinary Journal*, *17*(2), 280–302. https://doi.org/10.1080/10705511003659425

Lee, S.-Y., Song, X.-Y., & Tang, N.-S. (2007). Bayesian methods for analyzing structural equation models with covariates, interaction, and quadratic latent variables. *Structural Equation Modeling: A Multidisciplinary Journal*, *14*(3), 404–434. https://doi.org/10.1080/10705510701301511

MacKinnon, D. P., Fairchild, A. J., & Fritz, M. S. (2007). Mediation analysis. *Annual Review of Psychology*, *58*(1), 593-614. (PMID: 16968208) https://doi.org/10.1146/annurev.psych.58.110405.085542

Maplesoft, a division of Waterloo Maple Inc.. (2019). Maple [Computer software manual]. Waterloo, Ontario. Retrieved from `https://hadoop.apache.org`

Mardia, K. V., Kent, J. T., & Bibby, J. M. (1979). *Multivariate analysis*. Academic Press.

Marsh, H. W., Wen, Z., & Hau, K.-T. (2004). Structural equation models of latent interactions: Evaluation of alternative estimation strategies and indicator construction. *Psychological Methods*, *9*(3), 275–300. https://doi.org/10.1037/1082-989X.9.3.275

McDonald, R. (1967). *Nonlinear factor analysis* (No. 15). William Byrd Press.

McDonald, R. P. (1999). *Test theory: A unified treatment*. Mahwah, NJ: Lawrence Erlbaum.

Mooijaart, A., & Bentler, P. M. (2010). An alternative approach for nonlinear latent variable models. *Structural Equation Modeling: A Multidisciplinary Journal*, *17*(3), 357–373. https://doi.org/10.1080/10705511.2010.488997

Mooijaart, A., & Satorra, A. (2009). On insensitivity of the chi-square model test to nonlinear misspecification in structural equation models. *Psychometrika*, *74*(3), 443–455.

Mooijaart, A., & Satorra, A. (2012). Moment testing for interaction terms in structural equation modeling. *Psychometrika*, *77*(1), 65–84. https://doi.org/10.1007/s11336-011-9232-6

Murdoch, D., & Adler, D. (2023). rgl: 3D visualization using OpenGL [Computer software manual]. Retrieved from `https://CRAN.R-project.org/package=rgl` (R package version 1.1.3)

Narasimhan, B., Johnson, S. G., Hahn, T., Bouvier, A., & Kiêu, K. (2023). cubature: Adaptive multivariate integration over hypercubes [Computer software manual]. Retrieved from `https://CRAN.R-project.org/package=cubature` (R package version 2.0.4.6)

Nelsen, R. B. (2007). *An introduction to copulas*. Springer Science & Business Media.

Nestler, S. (2015). A specification error test that uses instrumental variables to detect latent quadratic and latent interaction effects. *Structural Equation Modeling: A Multidisciplinary Journal*, *22*(4), 542-551. https://doi.org/10.1080/10705511.2014.994744

Ng, J. C. K., & Chan, W. (2020). Latent moderation analysis: A factor score approach. *Structural Equation Modeling: A Multidisciplinary Journal*, *27*(4), 629–648. https://doi.org/10.1080/10705511.2019.1664304

R Core Team. (2023). R: A language and environment for statistical computing [Computer software manual]. Vienna, Austria. Retrieved from `https://www.R-project.org/`

Ren, Y.-F., & Liang, H.-Y. (2001). On the best constant in marcinkiewicz–zygmund inequality. *Statistics & Probability Letters*, *53*(3), 227–233.

Révész, P. (1967). *The laws of large numbers* (Vol. 4). Academic Press.

Rosseel, Y. (2012). lavaan: An R package for structural equation modeling. *Journal of Statistical Software*, *48*(2), 1–36.

Rosseel, Y., & Loh, W. W. (2022). A structural after measurement approach to structural equation modeling. *Psychological Methods*, Advance online publication.

Shiryaev, A. N. (2016). *Probability 1* (Vol. 95). Springer.

Skrondal, A., & Laake, P. (2001). Regression among factor scores. *Psychometrika*, *66*(4), 563–575.

Solymos, P., & Zawadzki, Z. (2023). pbapply: Adding progress bar to '*apply' functions [Computer software manual]. Retrieved from `https://CRAN.R-project.org/package=pbapply` (R package version 1.7-0)

Song, X.-Y., & Lee, S.-Y. (2005). Maximum likelihood analysis of nonlinear structural equation models with dichotomous variables. *Multivariate Behavioral Research*, *40*(2), 151–177. https://doi.org/10.1207/s15327906mbr4002_1

Song, X.-Y., & Lu, Z.-H. (2010). Semiparametric latent variable models with bayesian p-splines. *Journal of Computational and Graphical Statistics*, *19*(3), 590–608. https://doi.org/10.1198/jcgs.2010.09094

Song, X.-Y., Lu, Z.-H., Cai, J.-H., & Ip, E. H.-S. (2013). A bayesian modeling approach for generalized semiparametric structural equation models. *Psychometrika*, *78*(4), 624–647. https://doi.org/10.1007/s11336-013-9323-7

Song, X.-Y., Pan, J.-H., Kwok, T., Vandenput, L., Ohlsson, C., & Leung, P.-C. (2010). A semiparametric bayesian approach for structural equation models. *Biometrical Journal*(3), 314-332. https://doi.org/10.1002/bimj.200900135

Stefanski, L. A., & Carroll, R. J. (1990). Deconvolving kernel density estimators. *Statistics*, *21*(2), 169–184.

The MathWorks Inc. (2023). MATLAB version: 9.13.0 (R2023a) [Computer software manual]. Natick, Massachusetts, United States: Author Retrieved from `https://www.mathworks.com`

Wall, M. M., & Amemiya, Y. (2000). Estimation for polynomial structural equation models. *Journal of the American Statistical Association*, *95*(451), 929–940. https://doi.org/10.1080/01621459.2000.10474283

Wall, M. M., & Amemiya, Y. (2001). Generalized appended product indicator procedure for nonlinear structural equation analysis. *Journal of Educational and Behavioral Statistics*, *26*(1), 1–29. https://doi.org/10.3102/10769986026001001

Wall, M. M., & Amemiya, Y. (2003). A method of moments technique for fitting interaction effects in structural equation models. *British Journal of Mathematical and Statistical Psychology*, *56*(1), 47–63. https://doi.org/10.1348/000711003321645331

Wang, X.-F., & Wang, B. (2011). Deconvolution estimation in measurement error models: The R package decon. *Journal of Statistical Software*, *39*(10), 1–24. https://doi.org/10.18637/jss.v039.i10

Wickham, H. (2016). *ggplot2: Elegant graphics for data analysis.* Springer-Verlag New York. Retrieved from `https://ggplot2.tidyverse.org`

Wickham, H. (2023). forcats: Tools for working with categorical variables (factors) [Computer software manual]. Retrieved from `https://CRAN.R-project.org/package=forcats` (R package version 1.0.0)

Wickham, H., & Seidel, D. (2022). scales: Scale functions for visualization [Computer software manual]. Retrieved from `https://CRAN.R-project.org/package=scales` (R package version 1.2.1)

Yalcin, I., & Amemiya, Y. (2001). Nonlinear factor analysis as a statistical method. *Statistical Science*, *16*(3), 275–294.

Department of Economics, BI Norwegian Business School, Oslo, Norway 0484
*E-mail address*: `steffeng@gmail.com`

Department of Psychology, Goethe University Frankfurt, Frankfurt am Main, Germany
*E-mail address*: `jirmer@psych.uni-frankfurt.de`
